# Global, regional, and national burden of leukemia, 1990–2021: a systematic analysis of the global burden of disease in 2021

**DOI:** 10.3389/fmed.2025.1542317

**Published:** 2025-04-15

**Authors:** Jiaxi Chen, Wenyi Pang, Miao Deng, Rubin Zheng, Yanjin Chen, Ziyang Zhang, Zhouke Tan, Zhixun Bai

**Affiliations:** ^1^Clinical College, Zunyi Medical University, Zunyi, Guizhou, China; ^2^Department of Nephrology, People's Hospital of Qianxinan Prefecture, Xingyi, Guizhou, China; ^3^Department of Nephrology, The Affiliated Hospital of Zunyi Medical University, Zunyi, Guizhou, China; ^4^Organ Transplant Center, The Affiliated Hospital of Zunyi Medical University, Zunyi, Guizhou, China

**Keywords:** leukemia, incidence, mortality, DALYs, global burden of disease

## Abstract

**Background:**

Leukemia is a heterogeneous hematologic malignancy with varying incidence, mortality, and disability-adjusted life years (DALYs) worldwide. Although the burden of leukemia has declined over recent decades, substantial disparities remain across different regions, socioeconomic levels, age groups, and sexes. This study looks at trends in leukemia cases, deaths, and the impact on quality of life from 1990 to 2021, aiming to uncover inequalities and help design better interventions.

**Methods:**

This study extracted data from the Global Burden of Disease (GBD) database (1990–2021) for five types of leukemia. The age-standardized incidence, mortality, and DALY rates for leukemia were calculated for 204 countries and 27 super regions worldwide. The estimated annual percentage change (EAPC) was used to quantify the trends in the leukemia burden, and trends were quantified using estimated annual percentage changes (EAPCs). Decomposition analysis examined the contributions of population growth, aging, and epidemiological changes. Additionally, autoregressive integrated moving average (ARIMA) and age-period-cohort (APC) models were employed. Inequality in leukemia burden was assessed using the Slope Index of Inequality (SII) and Concentration Index (CI).

**Results:**

Globally, the incidence and mortality of leukemia increased with age and were consistently higher in males. While incidence and mortality are projected to rise, DALYs are expected to decline slightly by 2031. Acute lymphoid leukemia (ALL) and acute myeloid leukemia (AML) dominated incidence and mortality patterns, with higher ALL burdens in low-SDI regions and more pronounced AML burdens in higher-SDI regions. Decomposition analysis indicated that epidemiological changes and population growth drove trends in incidence and mortality, respectively. APC results showed declining period effects and lower risks in more recent birth cohorts. Inequality analyses revealed a growing disparity in incidence and mortality burdens in high-SDI countries, while DALYs inequality slightly narrowed.

**Conclusion:**

From 1990 to 2021, the global burden of leukemia has shifted, with persistent geographic, socioeconomic, and sex-related differences. Although overall improvements in prevention and treatment have reduced the DALYs burden, rising incidence and mortality in certain regions underscore the need for tailored public health strategies, early screening, and risk-based interventions to address persistent health inequalities and mitigate future leukemia burdens.

## Introduction

Leukemia is a type of hematologic malignancy caused by the malignant transformation of hematopoietic cells in the bone marrow (1), and it is commonly observed in children, accounting for 28% of pediatric cancer cases (2). Leukemia can be classified based on cell lineage into lymphoid and myeloid types, which affect lymphocytes and myeloid cells, respectively. It can also be categorized according to the rate of disease progression into acute and chronic leukemia. Acute leukemia typically progresses rapidly, with symptoms worsening in a short period, while chronic leukemia progresses more slowly. Therefore, leukemia can be specifically classified into acute lymphoid leukemia (ALL), acute myeloid leukemia (AML), chronic lymphocytic leukemia (CLL), and chronic myeloid leukemia (CML) (3, 4). Additionally, some types of leukemia that cannot be precisely classified are referred to as “other leukemia” in this article, representing other lymphoproliferative disorders. In recent years, the incidence and mortality rates of leukemia have decreased with advancements in prevention, early diagnosis, novel treatment strategies, and the use of targeted drugs (5–8). However, evidence suggests that the geographic distribution of different types of leukemia varies significantly worldwide, with differing incidence rates across populations of varying age, sex, and ethnicity (3, 7, 9, 10).

In the past few decades, the pathogenesis and treatment strategies of different types of leukemia have been extensively discussed (11–13). At the same time, we recognize that leukemia rates vary globally, influenced by factors such as age, sex, and economic conditions (14). Understanding the patterns of leukemia onset, as well as regional and temporal trends, can help identify and develop better prevention policies, increase the predictability of leukemia incidence, and thus promote precise prevention strategies for leukemia.

The Global Burden of Disease (GBD) study assessed the leukemia burden across 204 countries and regions, providing an opportunity to understand the current status of leukemia. A recent study described the burden of acute myeloid leukemia (AML) in various countries and regions worldwide (14), but did not address other types of leukemia. Therefore, this study retrieved detailed data on the incidence, mortality, and disability-adjusted life years (DALYs, which represent the years of life lost due to premature death and the years lived with disability due to the disease) for five specific types of leukemia from the Global Burden of Disease (GBD) database. By analyzing the temporal trends of incidence, mortality, and DALYs for these types of leukemia at the global, national, and regional levels from 1990 to 2021, this study further evaluates the disease burden of different types of leukemia. Additionally, this study looks at trends in leukemia cases, deaths, and the impact on quality of life, aiming to uncover inequalities and provide insights for designing better interventions. The findings of this study serve as an important extension and supplement to previous research on the leukemia burden (15, 16) and also contribute to the development of leukemia response strategies tailored to different countries and regions.

## Materials and methods

### Study data

The annual incidence of leukemia, age-standardized incidence rate (ASIR), number of deaths, age-standardized mortality rate (ASMR), disability-adjusted life years (DALYs), and age-standardized DALYs rate (DALYs rate) from 1990 to 2021 were collected using the Global Health Data Exchange (GHDx) query tool (http://ghdx.healthdata.org/gbd-results-tool). Data were categorized by sex, region, country, and leukemia type (Acute Lymphoid Leukemia, Acute Myeloid Leukemia, Chronic Lymphoid Leukemia, Chronic Myeloid Leukemia, and Other Leukemia). Data from 204 countries and regions were available and were classified into five social-demographic index (SDI) groups: low, low-middle, middle, high-middle, and high. Additionally, the world was divided into 21 regions geographically. Detailed descriptions of the GBD data have been provided in previous studies (17, 18), so they are not repeated here.

### Statistical analysis

This study used age-standardized incidence rate (ASIR), age-standardized mortality rate (ASMR), age-standardized DALYs rate (DALYs rate), and estimated annual percentage change (EAPC) to quantify the burden of leukemia and its different types. Standardization is essential when comparing populations with different age structures or when the age distribution of the same population changes over time. The ASR (per 100,000 population) in accordance with the direct method is calculated by summing up the products of the age-specific rates (a_i_, where I denotes the i^th^ age class) and the number of persons (or weight; w_i_) in the same age subgroup I of the chosen reference standard population, then dividing the sum of standard population weights, i.e.,


$$ASR=\frac{\sum_{\left\{i=1\right\}}^{A}a_{i}w_{i}}{\sum_{\left\{i=1\right\}}^{A}w_{i}}\times 100,\text{​}000$$

EAPC is the annual average percentage change in ASR over a specific time period. In short, EAPC helps us understand the trend in the burden of leukemia over time. The regression line fits the natural logarithm of the rate, expressed as y = α + βx + ε, where y = ln(ASR) and x = calendar year. The EAPC is calculated using the formula: 100 × (exp(β)−1), with its 95% confidence interval (CI) also derived from the linear regression model (19, 20). *R*^2^ and *p*-values were derived from Pearson correlation analysis and were used to assess the goodness of fit and statistical significance of the regression model. ASR represents the average age-standardized rate (ASR) for the period. CI is a statistical range that represents the uncertainty of the estimated results in the study. If both the EAPC estimate and the lower bound of its 95% CI are >0, an increasing trend in ASR is considered. If both the EAPC estimate and the upper bound of its 95% CI are <0, a decreasing trend in ASR is assumed. Otherwise, ASR is considered stable over time. To explore the factors influencing EAPC, this study assessed the relationship between EAPC and ASR at the national level. Decomposition analysis was used to visually display the roles of three factors—aging, population growth, and epidemiological changes—in driving the changes in leukemia incidence, mortality, and DALYs from 1990 to 2021. Epidemiological changes refer to the age- and population-adjusted mortality and incidence rates (21).

### Autoregressive integrated moving average model

The Autoregressive Integrated Moving Average (ARIMA) model can predict the future burden of leukemia based on its past burden. It consists of the autoregressive (AR) model and the moving average (MA) model. The basic assumption of the model is that the data sequence is a time-dependent random variable, and its autocorrelation can be characterized by the ARIMA model. The ARIMA equation is expressed as: Y_t_ = ϕ_1_Y_t − 1_ + ϕ_2_Y_t − 2_ + … + ϕ_p_Y_t − p_ + e_t_ – θ_1_e_t − 1_ - … - θ_q_e_t − q_, where (ϕ_1_Y_t − 1_ + ϕ_2_Y_t − 2_ + … + ϕ_p_Y_t − p_ + e_t_) represents the AR component, and (e_t_ – θ_1_e_t − 1_ - … - θ_q_e_t − q_) represents the MA component. Here, Y_t − 1_ refers to observations *t-p* periods ago, p and q denote AR and MA orders, and e_t_ is the random error for period *t* (22). The time series used in ARIMA modeling must be a stationary random sequence with a zero mean.

### Age-period-cohort model

The Age-Period-Cohort (APC) model examines the effects of age, period, and cohort on health outcomes. The age effect refers to the outcome risk at different age groups; the period effect refers to the impact of time changes on outcomes across age groups; and the cohort effect reflects the outcome changes within the same birth cohort. A logarithmic linear regression model is used to represent this: log(Y_i_) = μ + α^*^$age_{i}+\beta^{*}$period_i_ + γ^*^cohort_i_ + ε, where Y_i_ represents leukemia incidence, mortality, or DALYs rate, α, β, and γ are the coefficients for age, period, and cohort, μ is the intercept, and ε is the model residual. The intrinsic estimator (IE) method is combined with the APC model to obtain the net effects across the three dimensions (23).

### Cross-country inequalities analysis

The Slope Index of Inequality (SII) and the Concentration Index (CI) are standardized measures used to assess absolute and relative gradients of inequality. The SII is obtained through regression analysis, linking a country's ASIR, ASMR, or DALYs rate to its relative position in the Social-Demographic Index (SDI) distribution, with the midpoint of the population in the cumulative distribution ordered by SDI defining the reference point (24). A weighted regression model is used to test for heteroscedasticity. The Concentration Index is calculated by numerically integrating the area under the Lorenz curve, aligning the cumulative proportions of ASIR, ASMR, or DALYs rate with the cumulative population distribution ordered by SDI (25).

All the above statistics were performed using R 4.3.0, with a *p*-value < 0.05 considered statistically significant, to ensure that the research findings are reliable and meaningful.

## Result

### Global leukemia burden

**Figure 1:**
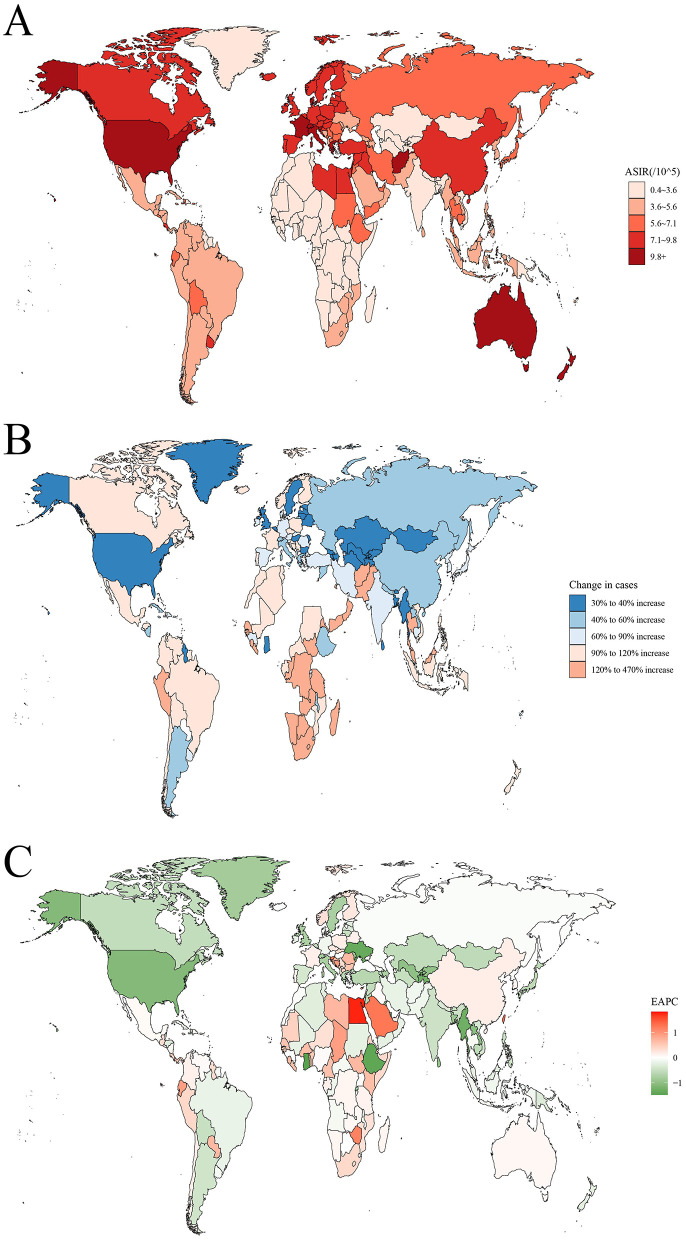
The global disease burden of leukemia for both sexes in 204 countries and territories. **(A)** The ASIR of leukemia in 2021; **(B)** The relative change in incident cases of leukemia between 1990 and 2021; **(C)** The EAPC of leukemia ASIR from 1990 to 2021. ASIR, age standardized incidence rate; EAPC, estimated annual percentage change.

The ASIR, ASMR, and DALYs rates for leukemia vary significantly across different regions of the world (Figure 1; Supplementary Figures S1, S2). In 2021, the highest ASIR was observed in the Principality of Monaco (25.1 per 100,000 population), followed by the Republic of San Marino (13 per 100,000) and the Hellenic Republic (11.5 per 100,000). In terms of absolute numbers, China had the highest number of leukemia cases in 2021 (105667.2 cases), followed by the United States (52060.4 cases) and India (34076.5 cases; Supplementary Table S1). In contrast, American Samoa had the highest ASMR (10 per 100,000), followed by Antigua and Barbuda (9.8 per 100,000) and the Arab Republic of Egypt (6.8 per 100,000). In terms of absolute numbers, China also had the highest number of leukemia deaths in 2021 (58903.5 deaths), followed by India (32,145 deaths) and the United States (29,786 deaths; Supplementary Table S2). Similarly, American Samoa had the highest DALYs rate (373.7 years per 100,000), followed by Antigua and Barbuda (323.5 years per 100,000) and the Arab Republic of Egypt (302.6 years per 100,000). In terms of absolute numbers, China had the highest DALYs (2205220.6 years), followed by India (1272622.9 years) and the United States (646396.1 years; Supplementary Table S3).

**Figure 2:**
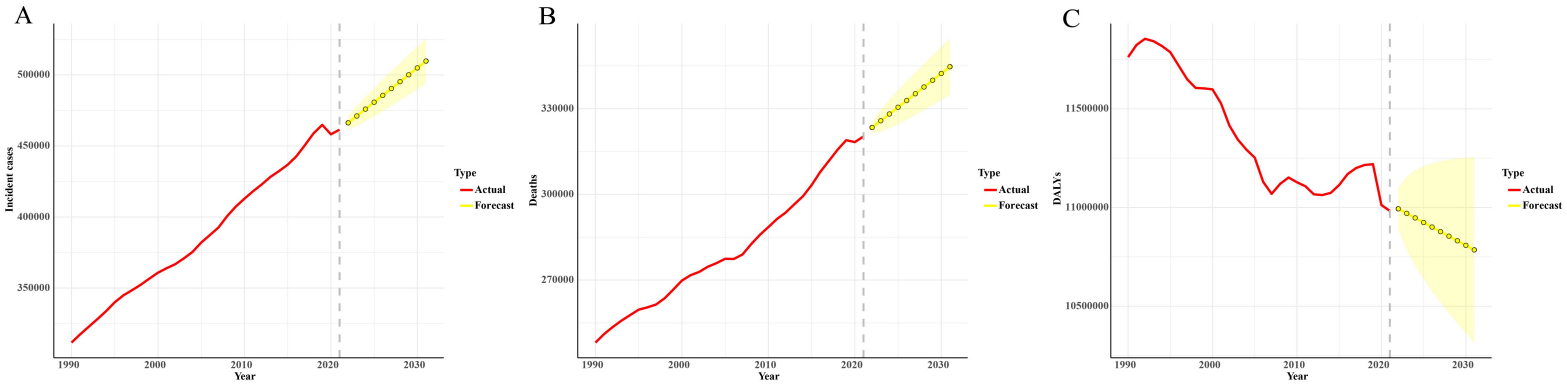
Predicted trends of leukemia incident cases **(A)**, deaths **(B)**, and DALYs **(C)** in the next decade (2021–2031). Red lines represent the true trend during 1990–2021; yellow dot lines and shaded regions represent the predicted trend and its 95% CI.

The ARIMA model results indicate that the number of leukemia cases is expected to increase from 461422.7 in 2021 to 509737.3 in 2031. The number of leukemia-related deaths is also projected to continue rising, from 320283.6 in 2021 to 344694.3 in 2031. In contrast, the DALYs for leukemia are expected to decrease, from 10982836.2 in 2021 to 10785356.1 in 2031 (Figure 2; Supplementary Table S4).

Decomposition analysis of age-standardized incidence number, age-standardized death number, and age-standardized DALYs number.

**Figure 3:**
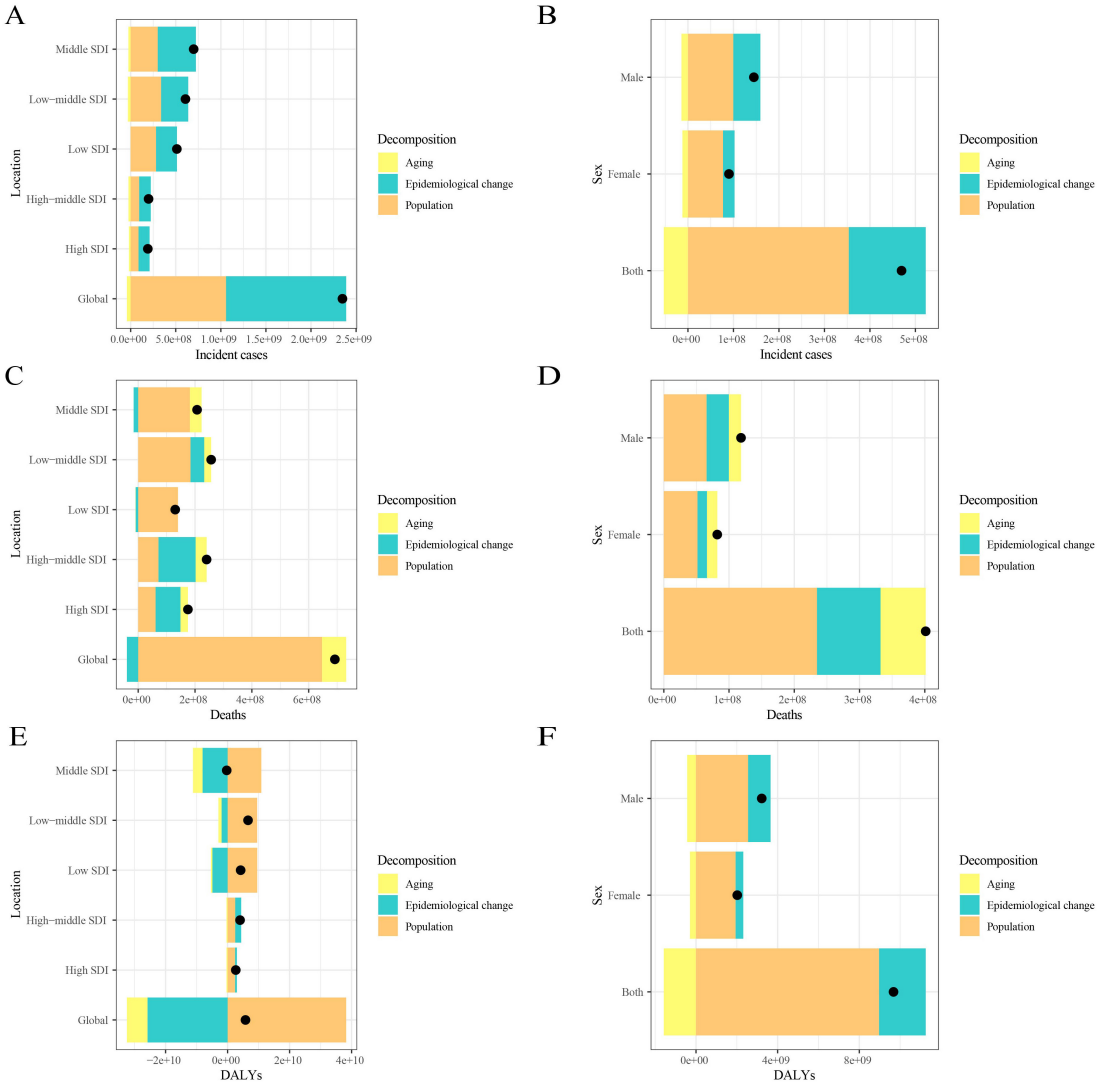
Changes in leukemia incidence, mortality, and DALYs from 1990 to 2021 according to population-level determinants, including population growth, aging, and epidemiological change, across different socio-demographic index (SDI) quintiles and by sex. Panels **(A, C, E)** show the decomposition of leukemia incidence, mortality, and DALYs by SDI quintile, while panels **(B, D, F)** show the decomposition by sex. The black dot represents the overall value of change contributed by all three components. Epidemiologic changes refer to the evolving patterns of CKD incidence and outcomes, independent of demographic shifts, as reflected in age- and population-standardized incidence and mortality. SDI, socio-demographic index.

From 1990 to 2021, the increase in global leukemia cases was primarily driven by epidemiological changes (56.70%), followed by population growth (45.06%). The greatest increase in leukemia cases was observed in middle SDI quintile regions, where epidemiological changes contributed the most (60.45%), followed by population growth (43.06%; Figure 3A; Supplementary Table S5). In contrast, in the gender stratification, population growth contributed the most (75.34%), while epidemiological changes contributed only 35.97% (Figure 3B; Supplementary Table S5). The increase in global leukemia-related deaths was predominantly influenced by population growth (93.42%), followed by aging (12.32%). The largest increase in leukemia deaths was observed in low to middle SDI quintile regions, where population growth contributed the most (71.82%) (Figure 3C; Supplementary Table S5). Similar results were observed in the gender stratification, where the increase in leukemia deaths was primarily driven by population growth (58.50%; Figure 3D; Supplementary Table S5). Notably, the global decrease in leukemia DALYs was mainly attributed to epidemiological changes (−447.36%), followed by aging (−115.68%), while the increase in DALYs was largely driven by population growth (663.04%). The largest decrease in leukemia DALYs occurred in middle SDI quintile regions, where it was solely influenced by population growth (−3815.72%; Figure 3E; Supplementary Table S5). In the gender stratification, the decrease in DALYs was solely influenced by aging (−16.29%; Figure 3F; Supplementary Table S5).

### The influential factor for EAPC

**Figure 4:**
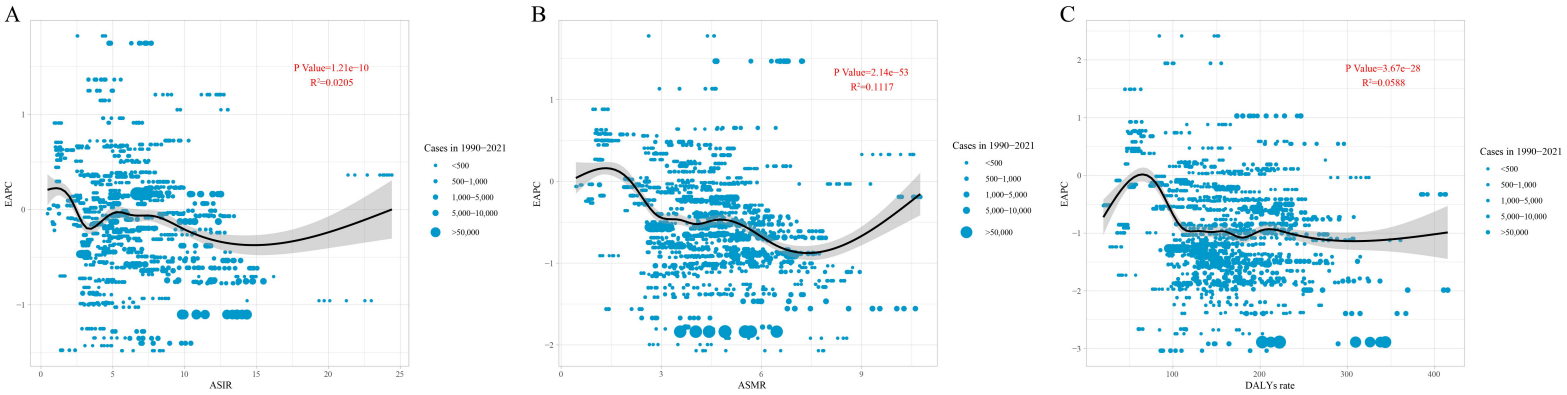
The correlation between EAPC and **(A)** ASIR, **(B)** ASMR, and **(C)** DALYs rate for leukemia. Circles represent leukemia cases from 1990 to 2021, with larger circles indicating higher cases. The *R*^2^ and *P*-values were derived from Pearson correlation analysis. EAPC, estimated annual percentage change; ASIR, age-standardized incidence rate; ASMR, age-standardized mortality rate; DALYs, disability-adjusted life years.

There is a significant association between EAPC and ASIR, ASMR, and DALYs rate (*P* < 0.05). When ASIR is below 5, the EAPC fluctuates significantly (from positive to negative values). As ASIR exceeds 15, the EAPC increases, showing a nonlinear trend (*P* = 1.21E-10, *R*^2^ = 0.0205). When ASMR is below 7, the EAPC generally shows a downward trend, whereas when ASMR exceeds 7, the EAPC increases (*P* = 2.14E-53, *R*^2^ = 0.1117). When the DALYs rate is below approximately 70, the EAPC shows an upward trend, then rapidly declines. When the DALYs rate exceeds around 120, the EAPC stabilizes (*P* = 3.67E-28, *R*^2^ = 0.0588; Figure 4).

### Age, period and cohort effects on leukemia incidence, mortality and DALYs

**Figure 5:**
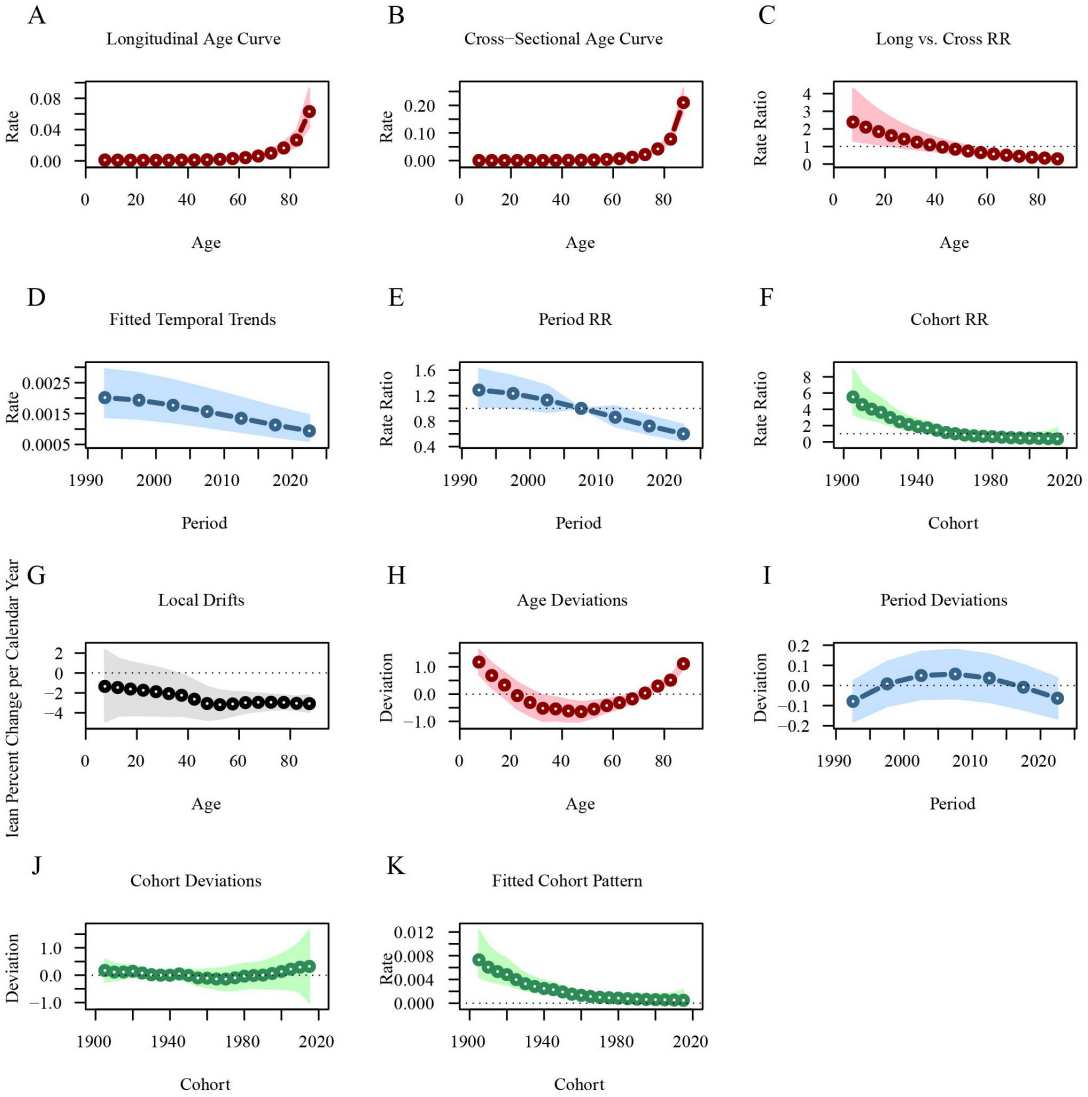
Age-period-cohort analysis of global leukemia incidence from 1990 to 2021. **(A)** Longitudinal age curve of leukemia incidence; **(B)** Cross-sectional age curve of leukemia incidence; **(C)** Comparison of longitudinal vs. cross-sectional rate ratios; **(D)** Fitted temporal trends in leukemia incidence; **(E)** Period rate ratios over time; **(F)** Cohort rate ratios by birth cohort; **(G)** Local drifts in leukemia incidence by age; **(H)** Age deviations from the fitted model; **(I)** Period deviations from the fitted model; **(J)** Cohort deviations from the fitted model; **(K)** Fitted cohort pattern of leukemia incidence.

Figure 5 illustrates the age-period-cohort effects on leukemia incidence. The longitudinal age curve shows that the leukemia incidence is lower in age groups under 60 years [RR_age(57.5)_ = 0.0028, 95%CI: 0.0019–0.0041], but it increases sharply in those aged 60 and above [RR_age(87.5)_ = 0.0630, 95%CI: 0.0428–0.0929]. The leukemia incidence across different periods has been steadily decreasing, with the RR value dropping from 1.2885 in 1992.5 to 0.601 in 2022.5. Similarly, the leukemia incidence is higher in early birth cohorts [RR_cohort(1905)_ = 5.5148, 95%CI: 3.3934–8.9625] and lower in recent birth cohorts [RR_cohort(2015)_ = 0.3693, 95%CI: 0.0805–1.6941; Figure 5; Supplementary material 1].

**Figure 6:**
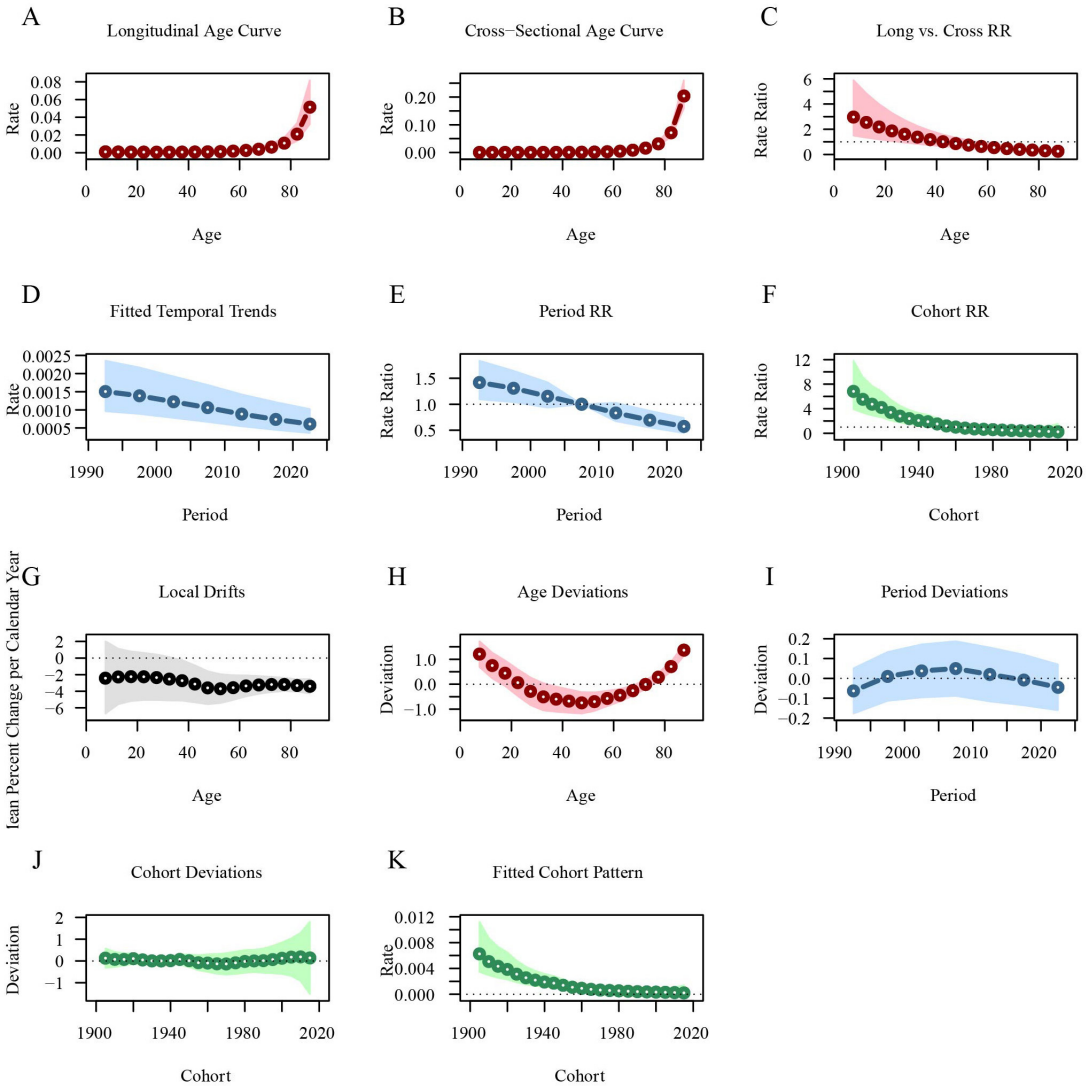
Age-period-cohort analysis of global leukemia mortality from 1990 to 2021. **(A)** Longitudinal age curve of leukemia mortality; **(B)** Cross-sectional age curve of leukemia mortality; **(C)** Comparison of longitudinal vs. cross-sectional rate ratios; **(D)** Fitted temporal trends in leukemia mortality; **(E)** Period rate ratios over time; **(F)** Cohort rate ratios by birth cohort; **(G)** Local drifts in leukemia mortality by age; **(H)** Age deviations from the fitted model; **(I)** Period deviations from the fitted model; **(J)** Cohort deviations from the fitted model; **(K)** Fitted cohort pattern of leukemia mortality.

Similarly, the longitudinal age curve shows that the leukemia mortality rate is lower in age groups under 60 years [${\text{RR}}_{\text{age}\left(57.5\right)}^{‵}$ = 0.0018, 95%CI: 0.0011–0.0028], but increases sharply in those aged 60 and above [${\text{RR}}_{\text{age}\left(87.5\right)}^{‵}$ = 0.0514, 95%CI: 0.0323–0.0818]. The leukemia mortality rate across different periods has been steadily decreasing, with the RR value dropping from 1.4184 in 1992.5 to 0.573 in 2022.5. Leukemia mortality is higher in early birth cohorts [${\text{RR}}_{\text{cohort}\left(1905\right)}^{‵}$ = 6.8197, 95%CI: 3.935–11.8192] and lower in recent birth cohorts [${\text{RR}}_{\text{cohort}\left(2015\right)}^{‵}$ = 0.2325, 95%CI: 0.0354–1.5279; Figure 6; Supplementary material 2].

**Figure 7:**
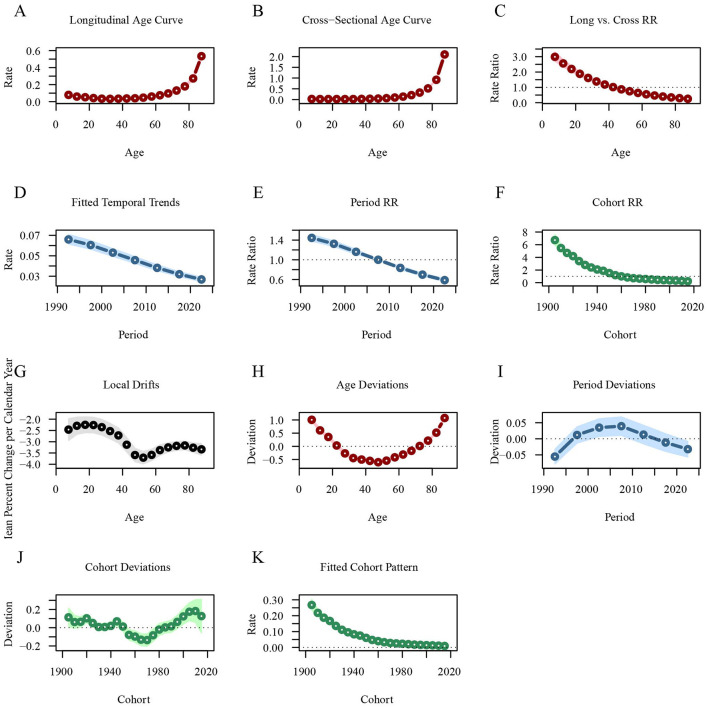
Age-period-cohort analysis of global leukemia DALYs from 1990 to 2021. **(A)** Longitudinal age curves of leukemia DALYs; **(B)** Cross-sectional age curve of leukemia DALYs; **(C)** Comparison of longitudinal vs. cross-sectional rate ratios; **(D)** Fitted temporal trends in leukemia DALYs; **(E)** Period rate ratios over time; **(F)** Cohort rate ratios by birth cohort; **(G)** Local drifts in leukemia DALYs by age; **(H)** Age deviations from the fitted model; **(I)** Period deviations from the fitted model; **(J)** Cohort deviations from the fitted model; **(K)** Fitted cohort pattern of leukemia DALYs. DALYs, disability-adjusted life years.

In contrast, the longitudinal age curve indicates that the rate of DALYs for leukemia decreases in individuals under 35 years, with the RR value dropping from 0.0796 at 7.5 years to 0.0331 at 32.5 years. After 35 years, the rate begins to increase gradually, particularly rising sharply after the age of 55 [${\text{RR}}_{\text{age}\left(87.5\right)}^{‵‵}$ = 0.5341, 95%CI: 0.4903–0.5817]. The rate of DALYs for leukemia has been steadily decreasing across different periods, with the RR value decreasing from 1.4435 in 1992.5 to 0.5866 in 2022.5. The rate of DALYs for leukemia is higher in early birth cohorts [${\text{RR}}_{\text{cohort}\left(1905\right)}^{‵‵}$ = 6.7176, 95%CI: 5.9372–7.6004] and lower in recent birth cohorts [${\text{RR}}_{\text{cohort}\left(2015\right)}^{‵‵}$ = 0.2302, 95%CI: 0.1856–0.2856; Figure 7; Supplementary material 3].

The distribution changes of leukemia subtypes across regions and periods.

**Figure 8:**
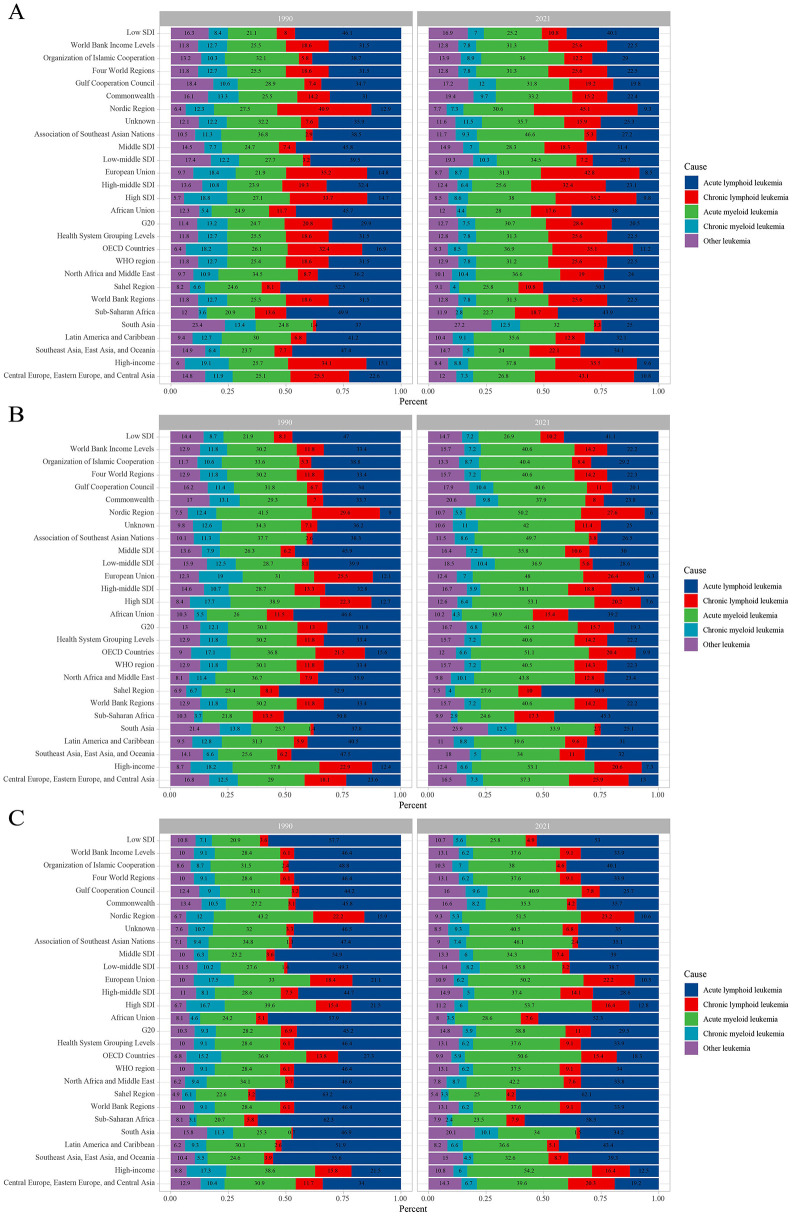
Contribution of leukemia subtypes incident cases **(A)**, deaths **(B)**, and DALYs **(C)**, both sexes and by region, in 1990 and 2021. DALYs, disability-adjusted life years.

The leukemia burden by type in different regions of the world in 1990 and 2021 is as follows. In all regions, the incidence of ALL decreased compared to 1990, while the incidence of CLL increased. Overall, the incidence of CML decreased. Notably, the incidence of ALL remained dominant in Sub-Saharan Africa, with little change (Figure 8A). Similarly, compared to 1990, the proportion of ALL deaths decreased in all regions, while the proportion of CLL deaths increased. It is noteworthy that the proportion of AML deaths increased in all regions, while the proportion of CML deaths decreased, with this change being more pronounced in regions with higher socio-economic levels (e.g., High SDI, Nordic region, ASEAN, and High-income countries). Overall, the proportion of deaths from other leukemias increased (Figure 8B). A similar trend was observed for leukemia DALYs. Compared to 1990, the proportion of DALYs from ALL decreased in all regions, while the proportion from CLL and AML increased. With the exception of the Gulf Cooperation Council region, the proportion of DALYs from CML decreased in all other regions. Overall, the proportion of DALYs from other leukemias increased. It is worth noting that the changes in DALYs for ALL and AML were more pronounced in countries with higher economic levels (e.g., High SDI and Nordic region; Figure 8C).

**Figure 9:**
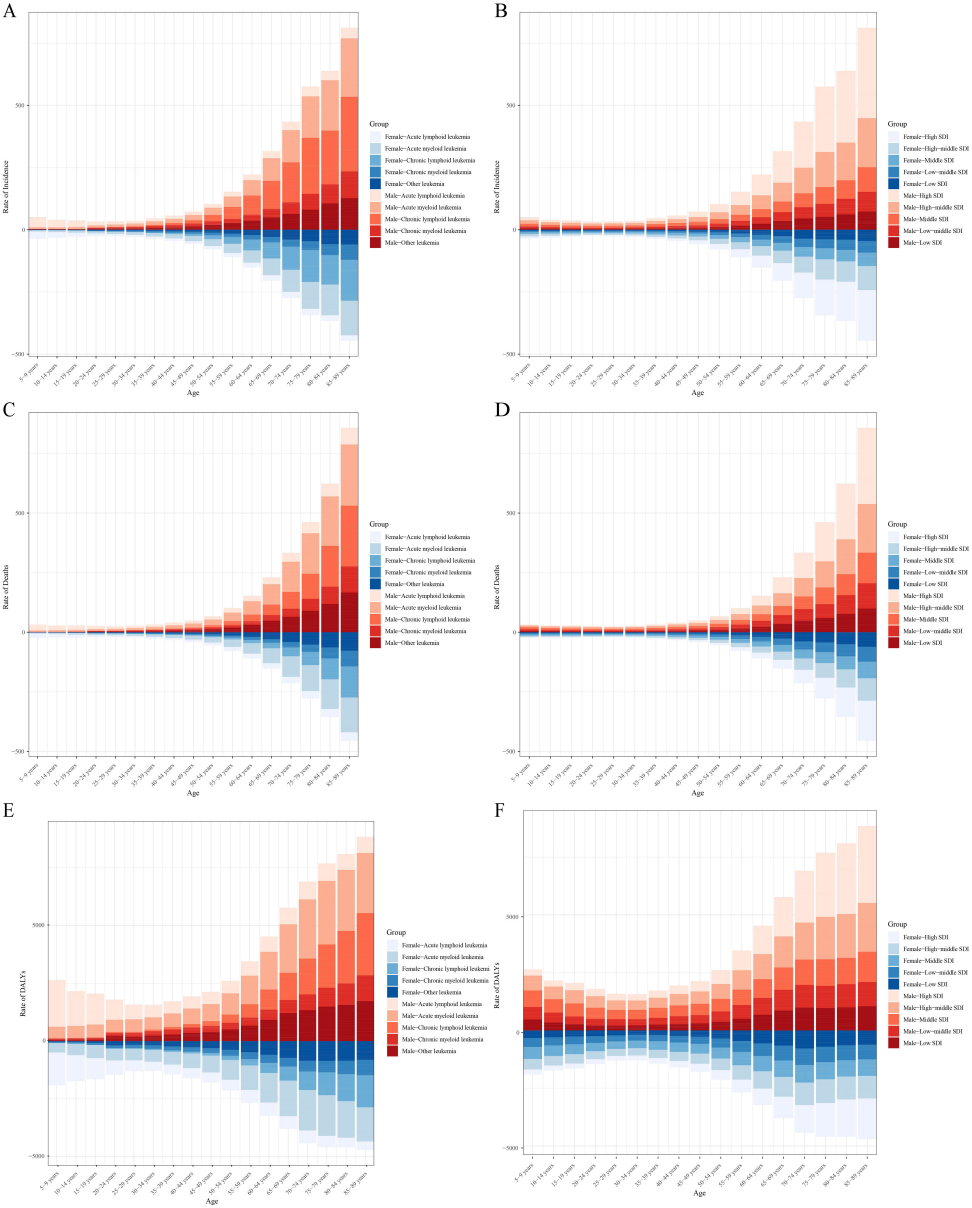
Age-specific incidence **(A, B)**, mortality **(C, D)**, and DALYs **(E, F)** of leukemia stratified by cause, sex, and SDI levels.

After stratifying by specific age groups and gender, it was observed that the leukemia incidence in individuals under 25 years is predominantly due to ALL, while in older age groups, leukemia is primarily AML and CLL. This distribution is not affected by gender. The leukemia incidence increases rapidly with age, and in all age groups, the incidence in males is higher than in females (Figure 9A). Overall, leukemia incidence across age groups is mainly concentrated in higher SDI regions, with males having a higher incidence than females (Figure 9B). Like incidence, leukemia mortality in individuals under 25 years is mainly due to ALL, while in older age groups, it is primarily due to AML and CLL. Mortality increases rapidly with age, and in all age groups, males have a higher mortality rate than females (Figure 9C). Overall, leukemia mortality across age groups is mainly concentrated in higher SDI regions, with males having a higher mortality rate than females (Figure 9D). Figure 9E shows that the DALYs burden in leukemia patients under 25 years is predominantly from ALL, while in older age groups, AML and CLL contribute more to the DALYs burden. Similarly, the DALYs burden from leukemia is heavier in higher SDI regions (Figure 9F).

### Global health inequality analysis of incidence, mortality and DALYs in leukemia from 1990 to 2021

**Figure 10:**
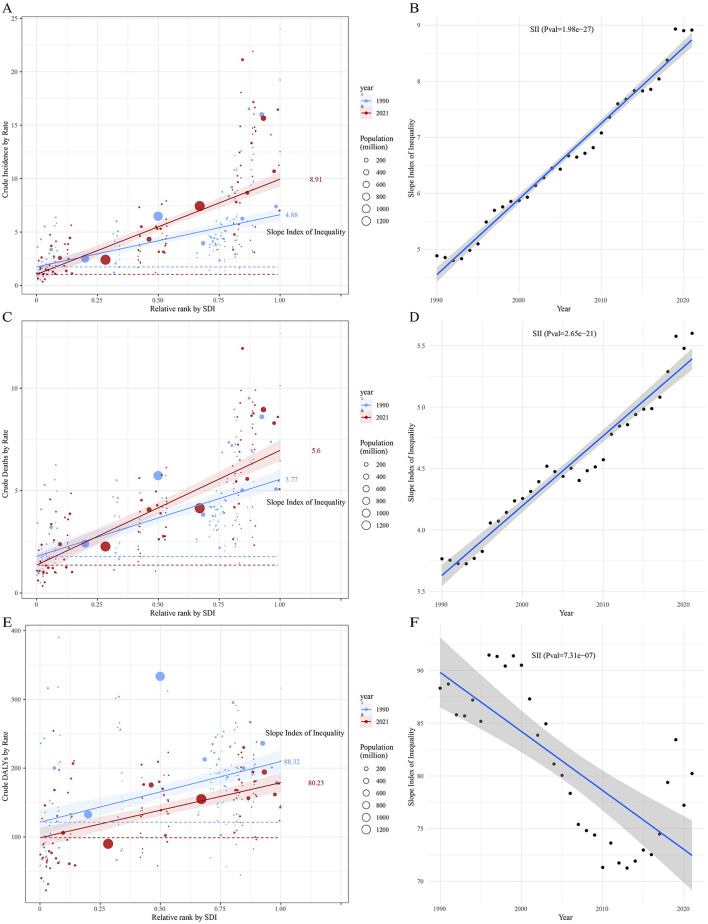
Trends in crude incidence, mortality, and DALYs rate of leukemia by SDI and SII from 1990 to 2021. Panels **(A, C, E)** show crude leukemia incidence, mortality, and DALYs rate by relative SDI rank, with the slope index of inequality (SII) for 1990 and 2021. Panels **(B, D, F)** display changes in SII over time for crude leukemia incidence, mortality, and DALYs rate. Circle sizes represent the population size for different countries and territories.

Compared to 1990, the inequality in leukemia incidence and mortality across countries and regions with different SDI levels increased in 2021. Overall, the incidence rate shows an upward trend with increasing SDI levels, and the Slope Index of Inequality (SII) for 2021 is significantly higher than that of 1990, with values of 8.91 and 4.88, respectively (Figure 10A; Supplementary Table S6). The SII also shows an increasing trend, with regression fitting results indicating highly significant statistical significance (*P* = 1.98e−27), suggesting that over the past few decades, the inequality between countries and regions with different levels of social development has continued to intensify, and the trend is very stable (Figure 10B). Similarly, the SII for leukemia mortality in 2021 is higher than in 1990, with SII values of 5.60 and 3.77, respectively (Figure 10C; Supplementary Table S6). The regression fitting results for the upward trend also show highly significant statistical significance (*P* = 2.65e−21), indicating that the inequality in leukemia mortality across countries and regions with different levels of social development has steadily increased over the past few decades (Figure 10D). In contrast, the inequality in leukemia DALYs has decreased, with the SII value dropping from 88.32 in 1990 to 80.23 in 2021 (Figure 10E; Supplementary Table S6). Overall, the SII shows a downward trend, indicating that the inequality in leukemia DALYs burden across countries and regions with different social development levels is narrowing (Figure 10F).

**Figure 11:**
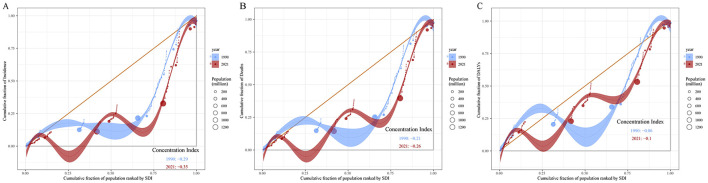
Concentration curves of leukemia incidence **(A)**, mortality **(B)**, and DALYs **(C)** by SDI in 1990 and 2021. The orange diagonal line represents perfect equality, where leukemia incidence, mortality and DALYs would be equally distributed across all SDI levels. Shaded areas represent the 95% confidence intervals of CI. DALYs, disability-adjusted life years; SDI, socio-demographic index; CI, concentration index.

As shown in Figure 11, the cumulative curves for 1990 and 2021 both deviate from the line of equality (orange diagonal line), indicating that leukemia incidence is more concentrated in regions with higher SDI. The concentration index (CI) for 1990 was −0.29, and for 2021 it was −0.35, suggesting an increase in the inequality of leukemia incidence based on socio-economic status (Figure 11A). Similarly, the CI for leukemia mortality decreased from −0.21 in 1990 to −0.26 in 2021, indicating that leukemia mortality in high SDI regions was higher in 2021, reflecting an increase in the inequality of mortality based on socio-economic status (Figure 11B). In contrast, the cumulative curve for leukemia DALYs is closer to the line of equality. The CI for 1990 was −0.06, and for 2021 it was −0.1, indicating a more equitable distribution of leukemia DALYs across regions with different SDI levels. However, the DALYs burden of leukemia is more concentrated in high SDI regions (Figure 11C).

## Discussion

This study comprehensively analyzed the temporal trends in incidence, mortality, and DALYs of five types of leukemia at global, national, and regional levels from 1990 to 2021. Overall, the burden of leukemia declined during this period. ALL and AML were the predominant subtypes, collectively accounting for more than 70% of leukemia cases in some regions, such as the Association of Southeast Asian Nations and the Sahel Region, though their individual proportions varied significantly across regions. Similar distributions were observed for leukemia mortality and DALYs. Furthermore, both leukemia incidence and mortality rates increased with age, while DALYs demonstrated a pattern of initial decline followed by an increase. Male patients had significantly higher leukemia incidence, mortality, and DALYs compared to females, consistent with previous studies (26–28). In both 1990 and 2021, the burden of leukemia incidence and mortality was primarily concentrated in middle- and high-SDI regions (CI <-0.2). Notably, health inequities in leukemia burden between countries and regions with varying levels of social development have persistently widened and remained stable, whereas the inequality in DALYs burden showed a decreasing trend.

Consistent with previous studies, ALL and AML are the two major subtypes of leukemia in terms of incidence and mortality (29, 30). From 1990 to 2021, the proportion of ALL decreased, while that of AML increased, with their combined proportion remaining stable. Among different types of leukemia, ALL incidence and mortality were more prevalent in low-SDI regions, such as Sub-Saharan Africa and the Sahel Region. Approximately 80% of ALL cases occur in children (31), which may explain the higher incidence of ALL in regions with high fertility rates. The incidence of AML is rising, and ASIR (age-standardized incidence rate) and ASMR (age-standardized mortality rate) in high-SDI regions are generally higher than those in low-SDI regions, consistent with previous findings (14). CLL is the most common leukemia among adults in Western countries but is relatively rare in Asia (32). Similarly, this study found that the highest proportion of CLL was observed in the Nordic Region and the European Union. Additionally, we found that CML had the lowest incidence among the four leukemia subtypes and was most observed in South Asia.

The ARIMA model results indicate that the number of leukemia cases is projected to rise from 461422.7 in 2021 to 509737.2 in 2031, while leukemia-related deaths are expected to increase from 320283.6 in 2021 to 344694.3 in 2031. In contrast, leukemia DALYs are predicted to decline from 10982836.2 in 2021 to 10785356.1 in 2031. One possible explanation for these trends is the increasing global population of elderly individuals (33), as leukemia predominantly affects older adults. Meanwhile, the Slope Index of Inequality (SII) for leukemia incidence and mortality has continued to rise steadily, and the Concentration Index (CI) has deviated further from the equality line over time, indicating a growing inequality in the leukemia burden across countries and regions with different levels of social development. The rise in leukemia cases and mortality will impact global health strategies, and this trend demands that public health policymakers in various regions adopt tailored interventions to address the increasing disease burden. This includes focusing more on elderly populations, strengthening early diagnosis, and providing more personalized and timely treatment options, especially in high-risk areas. At the same time, international cooperation is essential. Practical and feasible intervention measures should be designed for countries and regions with different socio-economic backgrounds. Decomposition analysis revealed that, from 1990 to 2021, the changes in leukemia incidence, mortality, and DALYs in higher SDI regions (High-Middle and High SDI) were primarily driven by epidemiological transitions. This may be associated with the increased prevalence of risk factors, such as unhealthy diets, physical inactivity, and air pollution, linked to modernization (34–36). Additionally, the study observed a persistent negative EAPC (Estimated Annual Percentage Change) with increasing ASIR, ASMR, and DALYs rates. A plausible explanation for this finding is that leukemia has been controlled to some extent through improved health awareness and policy interventions. Therefore, we recommend strengthening early leukemia screening and interventions for high-risk populations (e.g., individuals exposed to chemical materials and ionizing radiation) (37, 38), as well as promoting healthy lifestyles to minimize the burden of leukemia.

Age-period-cohort analysis revealed that leukemia incidence and mortality rates initially decrease with age, reaching their lowest point around 25 years, and then steadily increase thereafter. Studies have shown that age is a significant risk factor for leukemia, with varying incidence and prognosis across different age groups. The increased leukemia incidence in older individuals may be attributed to declining hematopoietic function and immunity (39–42). Additionally, genetic mutations may contribute to the occurrence of leukemia in children (43, 44). The period effect reflects the impact of specific time-related factors, such as medical advancements, diagnostic improvements, and socioeconomic and cultural changes, on the leukemia burden. In contrast, the cohort effect highlights the influence of early-life socioeconomic, behavioral, and environmental exposures, as well as risks associated with different birth cohorts. Our findings indicate that period effects contributed to a decline in leukemia incidence, mortality, and DALYs rates. Furthermore, earlier birth cohorts exhibited higher risks of leukemia incidence, mortality, and DALYs rates compared to recent birth cohorts. This trend may be associated with advancements in medical technologies, improvements in treatment modalities, the implementation of public health policies, and increased health awareness. Notably, across all SDI regions, years, and age groups, the leukemia burden was consistently higher in males than in females, aligning with previous studies (45–48). This observation underscores the need for further research into the underlying mechanisms of gender differences in leukemia burden. Such studies could provide scientific evidence to inform the development of more targeted prevention and treatment strategies, particularly for addressing the higher burden of leukemia among males.

Health inequality analysis reveals that there is an unequal burden of leukemia across countries and regions with different levels of social development, and this disparity is increasing. Notably, in high-SDI (Socio-Demographic Index) countries, despite improvements in treatment and preventive measures for leukemia, the inequality in incidence and mortality rates continues to widen. This suggests that there may still be unequal distribution of healthcare resources, disparities in health access among different populations, or that certain high-risk groups fail to receive timely early screening and treatment. Although high-SDI countries have relatively abundant resources for treatment and early screening, low-SDI regions continue to face a higher leukemia burden, particularly in acute lymphoblastic leukemia (ALL). The incidence of ALL is higher in low-income regions, such as sub-Saharan Africa, where medical resources and infrastructure are relatively scarce. This leads to delays in early diagnosis and treatment, thereby exacerbating the disease burden and mortality rate (49). Additionally, low-income countries and regions may lack high-quality data collection mechanisms, leading to an underestimation of the actual burden of leukemia, further exacerbating health inequalities. The disparities in leukemia burden are not only closely related to the economic development level of regions but also to the healthcare policies, accessibility of medical resources, and public health interventions in different countries and regions. In industrialized areas, particularly those with a concentration of chemical production, oil refining, and manufacturing industries, exposure to harmful chemicals such as benzene is considered a significant risk factor for leukemia (50). We recommend that future public health strategies focus more on addressing and mitigating these disparities. Health systems should improve the allocation of healthcare resources, particularly between high-income and low-income countries, to ensure that people in different socio-economic groups within these regions have access to timely leukemia screening, diagnosis, and treatment. Promoting the cross-border flow of resources through international cooperation and health aid, and reducing global healthcare gaps, will be one of the key directions for future efforts. Additionally, it is crucial to enhance the quality and transparency of health data collection in low-income countries and regions. The accuracy and comprehensiveness of data directly impact the scientific and effective nature of policy decisions. In the future, international organizations should promote the establishment of global health data-sharing platforms and encourage data standardization to better assess the leukemia burden in different regions and countries, and to develop corresponding intervention strategies. At the same time, stronger regulation of risk factors such as occupational exposure and environmental pollution, especially in industrialized areas, should be emphasized to reduce exposure to harmful substances like benzene and mitigate the resulting health inequalities.

This study has several limitations. First, the accuracy and robustness of the GBD estimates largely depend on the quality and quantity of data used in the modeling process. For example, underreporting of leukemia cases or a lack of medical information in certain impoverished regions may lead to an underestimation of specific leukemia subtypes. Second, variations in data collection methods, technologies, and tools vary across different countries and regions. Cross-national evidence may be confounded, making it difficult to distinguish between good and bad quality, which could affect the analysis of health inequalities. Finally, the age-period-cohort analysis reflects population-level results, which are inherently subject to ecological fallacy.

## Conclusion

From 1990 to 2021, the global burden of leukemia has shifted, with persistent geographic, socioeconomic, and sex-related differences. Although overall improvements in prevention and treatment have reduced the DALYs burden, rising incidence and mortality in certain regions underscore the need for tailored public health strategies, early screening, and risk-based interventions to address persistent health inequalities and mitigate future leukemia burdens.

## Data Availability

The raw data supporting the conclusions of this article will be made available by the authors, without undue reservation.
