# Supplementary material for: Global, regional, and national burden of leukemia, 1990–2021: a systematic analysis of the global burden of disease in 2021
Source: Front Med (Lausanne). 2025 Apr 15;12:1542317. doi: 10.3389/fmed.2025.1542317 (PMC12037485; doi:10.3389/fmed.2025.1542317)
Supplement: Supplementary file 4 [file Table_4.docx]

Table S1. The incident cases and age standardized incidence of leukemia caused by different reasons from 1990 to 2021.

| Cause | Location | 1990 | | 2021 | | 1990-2021 |
| --- | --- | --- | --- | --- | --- | --- |
|  |  | Incident cases  No. ×10^3^ (95% UI) | ASIR per 100,000  No. (95% UI) | Incident cases  No. ×10^3^ (95% UI) | ASIR per 100,000  No. (95% UI) | EAPC  No. (95% CI) |
| Leukemia | American Samoa | 1.2 (0.9-1.5) | 3.7 (2.9-4.5) | 1.8 (1.4-2.2) | 3.8 (2.9-4.8) | 0.28 (-0.53-1.1) |
| Leukemia | Antigua and Barbuda | 2.6 (2.4-2.8) | 4.7 (4.3-5) | 4.3 (4-4.6) | 4.5 (4.2-4.8) | -0.02 (-0.8-0.76) |
| Leukemia | Arab Republic of Egypt | 2007.7 (1668.2-2937.3) | 4.8 (3.9-7.5) | 5472.6 (3695.5-6963.4) | 7.2 (5.1-9.1) | 1.14 (0.33-1.95) |
| Leukemia | Argentine Republic | 2048.8 (1970.3-2127.2) | 6.3 (6.1-6.5) | 2671.9 (2465.1-2876.5) | 5.2 (4.8-5.6) | -0.36 (-1.12-0.4) |
| Leukemia | Australia | 1978.4 (1872.6-2087.3) | 10.6 (10-11.2) | 4429.1 (3962.2-4838.7) | 10.7 (9.8-11.7) | -0.18 (-1.19-0.84) |
| Leukemia | Barbados | 15.3 (14.4-16.2) | 5.5 (5.2-5.8) | 24.4 (19.1-30.3) | 5.6 (4.4-7) | 0.15 (-0.64-0.95) |
| Leukemia | Belize | 5.3 (4.9-5.8) | 3.2 (3-3.5) | 11.7 (10.5-13.2) | 3.3 (2.9-3.7) | 0.09 (-0.54-0.71) |
| Leukemia | Bermuda | 4 (3.7-4.2) | 6.5 (6-7) | 5.7 (4.8-6.9) | 5.5 (4.5-6.8) | -0.15 (-0.92-0.62) |
| Leukemia | Bolivarian Republic of Venezuela | 739.8 (712.2-766.3) | 4.7 (4.5-4.9) | 1343.3 (999.2-1760.6) | 4.9 (3.6-6.4) | -0.04 (-0.78-0.72) |
| Leukemia | Bosnia and Herzegovina | 168.9 (140-207.2) | 4.1 (3.4-5) | 295.4 (210-381) | 5.2 (3.8-6.7) | 0.51 (-0.29-1.31) |
| Leukemia | Brunei Darussalam | 10.9 (8.9-13.7) | 6.2 (5.1-7.7) | 19.1 (14-23.1) | 4.8 (3.5-5.8) | -0.24 (-1.06-0.59) |
| Leukemia | Burkina Faso | 99 (72.5-130.3) | 1.1 (0.9-1.4) | 260 (125.6-362.4) | 1.4 (0.7-1.9) | 0.29 (-0.1-0.67) |
| Leukemia | Canada | 3844.3 (3627-4042.4) | 12.7 (11.9-13.4) | 6164.8 (5589-6724.3) | 9.8 (9-10.6) | -0.44 (-1.56-0.68) |
| Leukemia | Central African Republic | 47.8 (32.9-67.1) | 2.5 (1.8-3.2) | 84.6 (53-122.4) | 2.3 (1.5-3.2) | -0.05 (-0.64-0.54) |
| Leukemia | Commonwealth of Dominica | 3.6 (3-4.3) | 5.5 (4.6-6.6) | 4.5 (3.4-5.7) | 6.2 (4.6-7.9) | 0.22 (-0.54-0.98) |
| Leukemia | Commonwealth of the Bahamas | 8.4 (7.7-9) | 4.2 (3.8-4.5) | 16.3 (13.1-20.1) | 4.1 (3.3-5) | -0.01 (-0.67-0.66) |
| Leukemia | Cook Islands | 0.3 (0.2-0.3) | 1.8 (1.3-2.3) | 0.3 (0.2-0.4) | 1.3 (0.9-1.7) | -0.33 (-0.79-0.14) |
| Leukemia | Czech Republic | 1074.6 (1016.8-1142.7) | 8.4 (7.9-8.9) | 1793.6 (1520.5-2086.9) | 8.9 (7.5-10.4) | -0.06 (-0.98-0.87) |
| Leukemia | Democratic People's Republic of Korea | 1009.6 (707.4-1371.5) | 5.1 (3.6-6.9) | 1414.1 (1019.5-1974.1) | 4.9 (3.5-6.8) | -0.03 (-0.83-0.78) |
| Leukemia | Democratic Republic of Sao Tome and Principe | 1 (0.7-1.3) | 0.8 (0.6-1) | 1.2 (0.8-1.8) | 0.8 (0.5-1) | -0.05 (-0.34-0.24) |
| Leukemia | Democratic Republic of the Congo | 528.5 (388.2-721.6) | 2 (1.4-2.6) | 1096.4 (687.2-1570.3) | 2 (1.2-3) | 0.05 (-0.47-0.57) |
| Leukemia | Democratic Republic of Timor-Leste | 30.9 (17.8-45.1) | 5.2 (3.5-7) | 50.2 (37.6-65.7) | 4.4 (3.3-5.8) | -0.24 (-1.02-0.54) |
| Leukemia | Democratic Socialist Republic of Sri Lanka | 732.8 (599.5-880.9) | 5.3 (4.4-6.4) | 895.4 (590-1254.2) | 3.7 (2.5-5.1) | -0.53 (-1.32-0.27) |
| Leukemia | Dominican Republic | 240 (194.4-289) | 3.8 (3.1-4.9) | 362.1 (272.1-471.8) | 3.5 (2.6-4.5) | 0.02 (-0.63-0.67) |
| Leukemia | Eastern Republic of Uruguay | 258.9 (244.4-274.4) | 7.2 (6.8-7.6) | 362.5 (330.6-393) | 7.4 (6.8-8) | -0.12 (-0.95-0.72) |
| Leukemia | Federal Democratic Republic of Ethiopia | 3683.7 (1900-5563) | 10.4 (6.9-14.2) | 5045 (3638.8-7358.1) | 7.1 (5-11) | -1 (-1.91--0.09) |
| Leukemia | Federal Democratic Republic of Nepal | 554.1 (362.5-749.2) | 3.5 (2.5-4.6) | 765.8 (563.1-1049.4) | 2.9 (2.2-4) | -0.19 (-0.81-0.43) |
| Leukemia | Federal Republic of Germany | 10268.4 (9616.4-10777.4) | 9.3 (8.8-9.8) | 15824.5 (14131.1-17291.5) | 9.7 (8.9-10.5) | -0.13 (-1.11-0.86) |
| Leukemia | Federal Republic of Nigeria | 943.3 (666.6-1326.6) | 1.1 (0.8-1.5) | 2109 (1123.1-2908.7) | 1.1 (0.6-1.5) | 0.01 (-0.35-0.38) |
| Leukemia | Federal Republic of Somalia | 141.7 (89-209.4) | 2.7 (1.9-3.7) | 363.5 (232.5-525.3) | 2.9 (1.9-4.1) | 0.2 (-0.37-0.78) |
| Leukemia | Federated States of Micronesia | 3.5 (2.5-4.8) | 4.9 (3.5-6.7) | 3.7 (2.5-5.1) | 4.4 (3-6) | -0.23 (-1.09-0.63) |
| Leukemia | Federative Republic of Brazil | 5436.5 (5259.5-5633.3) | 4.6 (4.5-4.8) | 10131.9 (9600.5-10562.5) | 4.3 (4.1-4.5) | -0.16 (-0.89-0.57) |
| Leukemia | French Republic | 7728.7 (7266.6-8179.1) | 10.5 (9.8-11.1) | 12605 (11038.6-14121.8) | 10.4 (9.2-11.5) | 0.07 (-0.86-1) |
| Leukemia | Gabonese Republic | 15.6 (11.8-19.7) | 2.2 (1.6-2.8) | 29.5 (17.8-43.9) | 2.3 (1.4-3.3) | 0.06 (-0.52-0.65) |
| Leukemia | Georgia | 341.5 (314.1-370.1) | 5.9 (5.4-6.4) | 278.5 (249.5-312.1) | 5.6 (5-6.2) | -0.11 (-0.84-0.63) |
| Leukemia | Grand Duchy of Luxembourg | 57.7 (54.3-61.2) | 12 (11.3-12.7) | 97.2 (87.1-108.2) | 10.2 (9.1-11.5) | -0.52 (-1.55-0.53) |
| Leukemia | Greenland | 1.6 (1.2-1.9) | 4.1 (3.2-4.9) | 1.8 (1.3-2.4) | 2.9 (2.1-3.8) | -0.45 (-1.1-0.2) |
| Leukemia | Grenada | 4.1 (3.7-4.5) | 5.1 (4.6-5.6) | 5.7 (5-6.5) | 5.3 (4.6-5.9) | 0.11 (-0.57-0.79) |
| Leukemia | Guam | 4.1 (3.3-5) | 4.1 (3.4-5.1) | 5.8 (4.6-7) | 3.2 (2.5-3.8) | -0.18 (-0.97-0.61) |
| Leukemia | Hashemite Kingdom of Jordan | 183.4 (147.6-225.9) | 8.1 (6.4-10.1) | 570.8 (408.3-749.2) | 6.4 (4.6-8.3) | -0.28 (-1.31-0.75) |
| Leukemia | Hellenic Republic | 1659.2 (1566.6-1743.5) | 12.8 (12-13.7) | 2299.9 (2096.1-2470.4) | 11.5 (10.7-12.3) | -0.59 (-1.44-0.28) |
| Leukemia | Hungary | 1123.6 (1051.3-1195) | 8.3 (7.7-8.8) | 1429 (1205.1-1689.6) | 8.3 (6.9-9.9) | -0.25 (-1.18-0.69) |
| Leukemia | Independent State of Papua New Guinea | 106.5 (53.9-150.7) | 3.5 (1.8-5.3) | 254.1 (145.6-365.4) | 3.2 (1.8-4.9) | -0.2 (-0.93-0.53) |
| Leukemia | Independent State of Samoa | 6.1 (4.8-8.2) | 5.5 (4.3-7.2) | 8.4 (6.4-11.5) | 5.2 (3.9-7) | -0.18 (-1.09-0.74) |
| Leukemia | Ireland | 390.1 (367.1-414.8) | 9.8 (9.2-10.4) | 555.7 (504.6-612.9) | 7.9 (7.2-8.7) | -0.25 (-1.25-0.76) |
| Leukemia | Islamic Republic of Afghanistan | 861 (446.6-1317.2) | 10.5 (5.7-15.8) | 1819.9 (1067.4-2624.6) | 10 (5.9-14.6) | -0.13 (-1.06-0.81) |
| Leukemia | Islamic Republic of Iran | 3451.4 (2114-4425.7) | 8 (4.9-9.9) | 5317.8 (3258.6-6371.1) | 6.8 (4.2-8.2) | -0.14 (-0.95-0.68) |
| Leukemia | Islamic Republic of Mauritania | 18 (13.8-22.9) | 1.1 (0.8-1.3) | 42.8 (22-64.7) | 1.3 (0.7-1.8) | 0.19 (-0.17-0.56) |
| Leukemia | Islamic Republic of Pakistan | 3323.5 (2340.6-4217.5) | 3.7 (2.8-4.6) | 6697.2 (5037.1-8993.3) | 3.6 (2.8-4.9) | -0.1 (-0.81-0.61) |
| Leukemia | Jamaica | 79.4 (73.4-86.8) | 3.7 (3.4-4) | 122.7 (95.6-158.4) | 4.2 (3.2-5.4) | 0.23 (-0.5-0.96) |
| Leukemia | Japan | 9300 (8914.6-9673.6) | 6.8 (6.5-7.3) | 13647.1 (12049.6-14607.2) | 5.7 (5.3-6) | -0.42 (-1.32-0.48) |
| Leukemia | Kingdom of Bahrain | 19.9 (15-23.9) | 8.2 (6-9.9) | 55.5 (39.3-74.5) | 5.6 (4.1-7.3) | -0.93 (-1.92-0.06) |
| Leukemia | Kingdom of Belgium | 1456.5 (1344.5-1561) | 11.1 (10.2-12) | 1888.9 (1634.4-2105.4) | 9.7 (8.6-10.8) | -0.5 (-1.41-0.41) |
| Leukemia | Kingdom of Bhutan | 16.1 (9.6-22.2) | 3.5 (2.3-4.8) | 19.4 (12.7-29.8) | 3 (2-4.5) | -0.28 (-0.94-0.38) |
| Leukemia | Kingdom of Cambodia | 548.6 (344.8-799.9) | 7.1 (4.9-9.7) | 860.2 (631-1133.2) | 6 (4.4-7.8) | -0.4 (-1.26-0.47) |
| Leukemia | Kingdom of Denmark | 822.7 (773.5-865.7) | 11.4 (10.7-12) | 1153.9 (1029.2-1272.8) | 11 (9.9-12) | -0.1 (-1.08-0.9) |
| Leukemia | Kingdom of Eswatini | 14.7 (11.1-21) | 3.4 (2.6-4.6) | 29.8 (19.6-44.6) | 4.1 (2.8-6) | 0.38 (-0.47-1.24) |
| Leukemia | Kingdom of Lesotho | 26.3 (19.2-35.1) | 2.6 (1.9-3.4) | 56.1 (40.3-75.9) | 4.4 (3.2-5.8) | 0.96 (0.16-1.75) |
| Leukemia | Kingdom of Morocco | 259.4 (192-331.4) | 1.3 (1-1.6) | 427 (291.9-550.9) | 1.2 (0.9-1.6) | -0.02 (-0.4-0.35) |
| Leukemia | Kingdom of Norway | 444.1 (422-462.5) | 7.6 (7.2-7.9) | 718.6 (648-771.4) | 8.1 (7.5-8.7) | 0.14 (-0.8-1.08) |
| Leukemia | Kingdom of Saudi Arabia | 336.7 (236.1-532.8) | 3.2 (2.2-5.6) | 1287.5 (898.4-2062.6) | 4.8 (3.5-7.3) | 0.78 (0.15-1.41) |
| Leukemia | Kingdom of Spain | 4663.2 (4387.8-4918.3) | 10.3 (9.6-11) | 7021.6 (6142.9-7696.5) | 9.3 (8.3-10.2) | -0.38 (-1.32-0.58) |
| Leukemia | Kingdom of Sweden | 1265.4 (1176.4-1342.2) | 10.5 (9.7-11.4) | 1532.4 (1346.2-1721.5) | 8.3 (7.3-9.2) | -0.5 (-1.52-0.54) |
| Leukemia | Kingdom of Thailand | 2622.8 (1996.1-3115.9) | 6 (4.4-7.1) | 5224.1 (3149-6883.2) | 6.1 (3.7-7.9) | -0.2 (-1.05-0.66) |
| Leukemia | Kingdom of the Netherlands | 1818.7 (1702.4-1925.9) | 10.3 (9.6-11) | 2380.2 (2120.6-2579) | 7.9 (7.1-8.6) | -0.46 (-1.44-0.52) |
| Leukemia | Kingdom of Tonga | 2.1 (1.5-2.8) | 3 (2.2-4.1) | 2.7 (1.9-3.6) | 3 (2.1-4) | -0.1 (-0.83-0.65) |
| Leukemia | Kyrgyz Republic | 172.7 (154.7-189.9) | 4.2 (3.8-4.6) | 178.7 (147.6-210.1) | 2.9 (2.4-3.4) | -0.61 (-1.2--0.02) |
| Leukemia | Lao People's Democratic Republic | 232.6 (125.9-358.9) | 7.1 (4.3-10.3) | 305 (220.3-403.7) | 5.1 (3.7-6.7) | -0.71 (-1.56-0.15) |
| Leukemia | Lebanese Republic | 159.8 (108.5-236.9) | 6.8 (4.7-9.9) | 412.4 (307.2-530.3) | 7 (5.1-9) | 0.23 (-0.62-1.08) |
| Leukemia | Malaysia | 762.9 (596.1-900.5) | 5.5 (4.5-6.6) | 1515.2 (1267.6-1933.6) | 5.1 (4.3-6.6) | -0.14 (-0.97-0.7) |
| Leukemia | Mongolia | 61.3 (47.8-79) | 3.3 (2.6-4.1) | 79.5 (59.1-100.4) | 2.7 (2-3.3) | -0.33 (-1.01-0.37) |
| Leukemia | Montenegro | 37.9 (30.1-46.2) | 6.2 (4.9-7.5) | 62.2 (46.8-84.7) | 6.9 (5.2-9.2) | 0.28 (-0.62-1.2) |
| Leukemia | New Zealand | 419.4 (392.8-442.3) | 11 (10.3-11.6) | 765.5 (699.3-826.4) | 10 (9.2-10.7) | -0.17 (-1.15-0.82) |
| Leukemia | North Macedonia | 102.6 (85-127.7) | 5.5 (4.6-6.9) | 187.6 (136.2-245.2) | 6.2 (4.6-8) | 0.23 (-0.91-1.38) |
| Leukemia | Northern Mariana Islands | 1.3 (0.9-1.8) | 4.8 (3.2-6.3) | 1.9 (1.4-2.2) | 3.8 (2.9-4.5) | -0.43 (-1.2-0.34) |
| Leukemia | Palestine | 119.5 (91-158) | 8.9 (6.7-11.6) | 240.5 (192.3-313.3) | 7.1 (5.7-9) | -0.45 (-1.39-0.5) |
| Leukemia | People's Democratic Republic of Algeria | 673.9 (497.8-831) | 3.5 (2.6-4.2) | 1088.9 (798.3-1432) | 2.9 (2.1-3.8) | -0.14 (-0.8-0.53) |
| Leukemia | People's Republic of Bangladesh | 3675.6 (2353.7-5263.2) | 4.1 (2.9-5.3) | 4235.6 (2963.3-5766.4) | 2.9 (2-3.9) | -0.5 (-1.16-0.15) |
| Leukemia | People's Republic of China | 76203.8 (58311.8-90958) | 7.1 (5.5-8.6) | 105667.2 (75275.7-132236.9) | 7.2 (4.9-9.1) | 0.02 (-0.91-0.96) |
| Leukemia | Plurinational State of Bolivia | 423.4 (289.8-583) | 7.8 (5.5-10.4) | 683.2 (475.7-919.2) | 6.6 (4.6-8.8) | -0.32 (-1.21-0.57) |
| Leukemia | Portuguese Republic | 926.5 (880.2-972) | 7.8 (7.3-8.2) | 1581.5 (1378.2-1734.8) | 8.1 (7.2-8.9) | 0 (-0.92-0.93) |
| Leukemia | Principality of Andorra | 7.5 (5.1-11) | 14.6 (10-21.9) | 13.6 (8.8-19.9) | 11 (7.3-15.6) | -0.58 (-1.69-0.54) |
| Leukemia | Principality of Monaco | 9.3 (6.8-12.5) | 20.6 (14.9-28) | 16.5 (12.2-21.3) | 25.1 (18.2-32.8) | 0.43 (-0.8-1.67) |
| Leukemia | Puerto Rico | 230.6 (218.3-244.4) | 6.4 (6.1-6.8) | 336.1 (273.4-397) | 5.9 (4.9-6.9) | -0.23 (-1.03-0.58) |
| Leukemia | Republic of Albania | 110.2 (90-139.7) | 4.3 (3.4-5.6) | 155.1 (108.7-239.5) | 4.3 (3-6.3) | 0.18 (-0.57-0.94) |
| Leukemia | Republic of Angola | 166.4 (108.3-241) | 2.2 (1.6-2.9) | 422.9 (267.6-607.5) | 2.1 (1.2-3.2) | -0.04 (-0.59-0.51) |
| Leukemia | Republic of Armenia | 206.2 (197.1-216.6) | 6.5 (6.2-6.8) | 185.9 (163.7-211.9) | 4.9 (4.4-5.5) | -0.36 (-1.1-0.38) |
| Leukemia | Republic of Austria | 1002.7 (943.8-1062.5) | 9.7 (9.1-10.3) | 1495.8 (1309-1654.6) | 9.3 (8.2-10.2) | -0.08 (-1.07-0.91) |
| Leukemia | Republic of Azerbaijan | 340.9 (278.6-413.5) | 5 (4.1-6) | 412.2 (283.7-596.3) | 4 (2.8-5.7) | -0.47 (-1.22-0.3) |
| Leukemia | Republic of Belarus | 914.7 (856.2-968.3) | 7.7 (7.2-8.2) | 1161.2 (949-1414.4) | 8.1 (6.6-9.8) | -0.11 (-0.97-0.76) |
| Leukemia | Republic of Benin | 51.2 (38.2-68) | 1.1 (0.9-1.4) | 146.9 (70-209.1) | 1.3 (0.7-1.9) | 0.23 (-0.15-0.62) |
| Leukemia | Republic of Botswana | 23.2 (16.4-31.5) | 3.1 (2.1-4.2) | 50.3 (33.4-72.1) | 3 (2-4.2) | 0 (-0.74-0.73) |
| Leukemia | Republic of Bulgaria | 550.9 (504.4-601.3) | 5.3 (4.9-5.8) | 696.4 (567.8-831.6) | 5.8 (4.7-7) | 0.23 (-0.51-0.98) |
| Leukemia | Republic of Burundi | 123 (90.2-167) | 2.7 (2-3.6) | 219.2 (121.1-340.3) | 2.5 (1.5-3.7) | -0.1 (-0.65-0.45) |
| Leukemia | Republic of Cabo Verde | 8 (6.3-11.1) | 2.4 (1.9-3.4) | 16.6 (12.3-21.2) | 3.5 (2.6-4.4) | 0.35 (-0.28-0.99) |
| Leukemia | Republic of Cameroon | 105.7 (78.9-138.4) | 1.2 (0.9-1.5) | 348.9 (166.2-508.1) | 1.5 (0.7-2.2) | 0.27 (-0.14-0.68) |
| Leukemia | Republic of Chad | 53.4 (38.8-72.5) | 0.9 (0.7-1.2) | 201.3 (105.1-297) | 1.4 (0.7-2) | 0.4 (0.03-0.78) |
| Leukemia | Republic of Chile | 581.9 (554.8-606) | 5.1 (4.8-5.3) | 1077 (986.3-1163.4) | 4.8 (4.4-5.3) | 0.06 (-0.67-0.79) |
| Leukemia | Republic of Colombia | 1462.1 (1380.2-1544) | 5.5 (5.2-5.7) | 2700.7 (2268.3-3216.4) | 5.4 (4.5-6.4) | -0.05 (-0.85-0.76) |
| Leukemia | Republic of Costa Rica | 145 (136.2-152.8) | 6.1 (5.8-6.5) | 375.4 (335.1-416.5) | 7.5 (6.6-8.3) | 0.26 (-0.56-1.08) |
| Leukemia | République de Côte d’Ivoire | 80.8 (55.7-104.4) | 0.9 (0.6-1.1) | 176.8 (99.3-268.1) | 0.9 (0.5-1.2) | 0.02 (-0.28-0.32) |
| Leukemia | Republic of Croatia | 432.6 (381.8-489.2) | 7.6 (6.7-8.6) | 759.9 (650.4-878.5) | 9.4 (8-11.1) | 0.2 (-0.77-1.17) |
| Leukemia | Republic of Cuba | 617.9 (589.6-644.5) | 5.9 (5.6-6.2) | 856.6 (741.4-983.4) | 5.3 (4.6-6.1) | -0.16 (-0.92-0.61) |
| Leukemia | Republic of Cyprus | 68.1 (54.4-94.7) | 9.4 (7.4-13.4) | 192.3 (129-239.4) | 10.5 (7-13) | 0.65 (-0.53-1.84) |
| Leukemia | Republic of Djibouti | 6.6 (4.3-9.8) | 2.2 (1.5-3.3) | 23.5 (13.1-39) | 2.7 (1.6-4.2) | 0.27 (-0.3-0.83) |
| Leukemia | Republic of Ecuador | 385.2 (363.3-409) | 4.8 (4.5-5.1) | 997.9 (816-1207.6) | 5.8 (4.7-7) | 0.5 (-0.36-1.37) |
| Leukemia | Republic of El Salvador | 245.8 (213.2-306.1) | 5.2 (4.5-6.5) | 363.7 (261.3-452.2) | 5.7 (4.1-7.1) | 0.24 (-0.59-1.07) |
| Leukemia | Republic of Equatorial Guinea | 6.9 (4.8-9.5) | 2.2 (1.6-2.8) | 18.5 (9.3-30.4) | 2.1 (1.1-3.5) | -0.06 (-0.6-0.49) |
| Leukemia | Republic of Estonia | 191 (178.2-203.1) | 10.2 (9.5-10.9) | 210.2 (175.7-243.9) | 8.8 (7.5-10.2) | -0.4 (-1.4-0.61) |
| Leukemia | Republic of Fiji | 30 (16.7-39.9) | 5.6 (3.1-7.5) | 39.7 (22.2-54.9) | 5 (2.8-6.8) | -0.31 (-1.42-0.81) |
| Leukemia | Republic of Finland | 484.9 (457.1-508.8) | 7.4 (7-7.8) | 801.7 (709.5-877.3) | 7.7 (7.1-8.5) | 0.09 (-0.78-0.98) |
| Leukemia | Republic of Ghana | 305.5 (159.4-416.7) | 2.4 (1.3-3.2) | 386 (265.5-559.8) | 1.4 (1-2.1) | -0.72 (-1.21--0.23) |
| Leukemia | Republic of Guatemala | 272.6 (255.8-288) | 3.7 (3.6-3.9) | 610.9 (526.7-711) | 4.3 (3.7-5) | 0.11 (-0.67-0.89) |
| Leukemia | Republic of Guinea | 30.8 (21.2-40.9) | 0.5 (0.3-0.6) | 48.2 (25.5-82.9) | 0.4 (0.3-0.6) | -0.02 (-0.2-0.17) |
| Leukemia | Republic of Guinea-Bissau | 12.1 (8.2-17.5) | 1.4 (1-1.9) | 23.1 (12.9-31) | 1.6 (0.9-2.2) | 0.26 (-0.18-0.7) |
| Leukemia | Republic of Guyana | 20.3 (17.7-22.7) | 3.5 (3.1-3.9) | 24.4 (18.7-31.6) | 3.5 (2.7-4.4) | 0.19 (-0.46-0.84) |
| Leukemia | Republic of Haiti | 406.4 (210.8-637) | 7.3 (4.6-10.5) | 610.5 (379.5-913.9) | 5.9 (3.8-8.4) | -0.31 (-1.05-0.44) |
| Leukemia | Republic of Honduras | 227.8 (186.2-278) | 5.5 (4.4-6.7) | 390.7 (279.6-545.3) | 5 (3.6-6.7) | -0.11 (-0.91-0.69) |
| Leukemia | Republic of Iceland | 24.8 (22.8-26.9) | 9.1 (8.4-9.9) | 43.8 (38.5-49.2) | 8.8 (7.8-9.8) | -0.09 (-1.04-0.86) |
| Leukemia | Republic of India | 21395.3 (16737.7-26136.3) | 3.2 (2.5-3.8) | 34076.5 (27487.5-41218.8) | 2.7 (2.2-3.3) | -0.26 (-0.86-0.35) |
| Leukemia | Republic of Indonesia | 7302.3 (5467-9433.6) | 5 (3.8-6.3) | 12045.3 (9663.4-15524.4) | 4.8 (3.9-6.2) | -0.1 (-0.93-0.74) |
| Leukemia | Republic of Iraq | 932.5 (719.1-1281.1) | 7.1 (5.6-9.9) | 2187.5 (1633.1-2861.3) | 7.3 (5.5-9.3) | 0.05 (-0.81-0.92) |
| Leukemia | Republic of Italy | 9384.4 (8908.3-9778.4) | 13.2 (12.4-14) | 12636 (11295.8-13625.1) | 11 (10.2-11.8) | -0.71 (-1.74-0.32) |
| Leukemia | Republic of Kazakhstan | 711.5 (667.2-754.8) | 4.7 (4.4-5) | 635.7 (558.2-725.8) | 3.4 (3-3.9) | -0.33 (-1-0.35) |
| Leukemia | Republic of Kenya | 278.9 (207.2-363.2) | 1.8 (1.3-2.7) | 684 (490.6-929.1) | 2.2 (1.6-2.9) | 0.32 (-0.15-0.79) |
| Leukemia | Republic of Kiribati | 1.8 (1.2-2.3) | 3.1 (2-3.8) | 3 (1.7-4.2) | 3.1 (1.8-4.1) | -0.04 (-0.75-0.68) |
| Leukemia | Republic of Korea | 1987.3 (1627.5-2403.2) | 5.1 (4.4-6.5) | 3028.2 (1947.8-3706.7) | 4.8 (2.9-6.2) | -0.01 (-0.86-0.85) |
| Leukemia | Republic of Latvia | 291.8 (270.5-312.3) | 9 (8.3-9.6) | 275.4 (231.8-323.6) | 8 (6.8-9.4) | -0.47 (-1.42-0.49) |
| Leukemia | Republic of Liberia | 26.4 (17.9-38.4) | 1.1 (0.8-1.4) | 55.3 (25.5-81.2) | 1.4 (0.7-2) | 0.28 (-0.09-0.66) |
| Leukemia | Republic of Lithuania | 396 (370.3-421.9) | 9.4 (8.8-10.1) | 393.2 (336-448.9) | 7.9 (6.7-8.9) | -0.45 (-1.37-0.48) |
| Leukemia | Republic of Madagascar | 207.2 (162.1-274.3) | 2.1 (1.7-2.7) | 420.9 (277.5-582.4) | 2.1 (1.4-2.9) | 0.05 (-0.44-0.54) |
| Leukemia | Republic of Malawi | 84.9 (57.2-109.5) | 0.9 (0.6-1.1) | 112.8 (59.2-189.9) | 0.8 (0.5-1.2) | -0.06 (-0.36-0.23) |
| Leukemia | Republic of Maldives | 8 (4.4-12.1) | 5 (3.1-7.2) | 11.5 (8.1-15.5) | 2.9 (2.1-3.8) | -0.85 (-1.59--0.1) |
| Leukemia | Republic of Mali | 85.8 (58-113.3) | 1 (0.7-1.2) | 157.1 (94.2-252.1) | 0.8 (0.5-1.1) | -0.09 (-0.41-0.23) |
| Leukemia | Republic of Malta | 29.6 (27.2-32.2) | 7.4 (6.8-8.1) | 55.7 (47.8-63.4) | 7.9 (6.8-9.1) | -0.05 (-0.94-0.85) |
| Leukemia | Republic of Mauritius | 41.8 (39.9-43.6) | 4.8 (4.6-5) | 59.1 (54.9-62.3) | 3.7 (3.5-3.9) | -0.66 (-1.44-0.13) |
| Leukemia | Republic of Moldova | 252.4 (239.9-266.3) | 5.7 (5.4-6) | 185 (166-208.6) | 3.7 (3.3-4.3) | -0.54 (-1.25-0.18) |
| Leukemia | Republic of Mozambique | 482.9 (362.5-635.9) | 3.9 (3.1-4.7) | 814.6 (493.6-1318) | 3.8 (2.7-5.2) | 0.18 (-0.48-0.84) |
| Leukemia | Republic of Namibia | 20.1 (15.2-24.9) | 2.2 (1.8-2.8) | 41.1 (28.5-57.3) | 2.4 (1.7-3.3) | 0.04 (-0.59-0.67) |
| Leukemia | Republic of Nauru | 0.4 (0.3-0.5) | 5.5 (4-7.3) | 0.4 (0.3-0.6) | 5.2 (3.4-7.5) | -0.17 (-1.1-0.77) |
| Leukemia | Republic of Nicaragua | 160 (134.9-199.8) | 4.3 (3.7-5.4) | 223.2 (159.9-273.4) | 3.8 (2.8-4.6) | -0.02 (-0.79-0.74) |
| Leukemia | Republic of Niue | 0.1 (0.1-0.1) | 3.8 (2.6-5) | 0.1 (0.1-0.1) | 5.7 (4.2-7.1) | 0.05 (-0.76-0.86) |
| Leukemia | Republic of Palau | 0.3 (0.2-0.4) | 2.8 (2-3.7) | 0.5 (0.4-0.6) | 2.6 (1.9-3.2) | -0.06 (-0.58-0.46) |
| Leukemia | Republic of Panama | 92.4 (86.1-99.3) | 4.4 (4.1-4.7) | 238.5 (197-286.6) | 5.6 (4.6-6.7) | 0.32 (-0.48-1.12) |
| Leukemia | Republic of Paraguay | 144.3 (115.3-173.3) | 4.4 (3.5-5.3) | 329.4 (240.6-424.1) | 5.3 (3.9-6.8) | 0.42 (-0.32-1.16) |
| Leukemia | Republic of Peru | 955.2 (782.9-1239.8) | 5.1 (4.2-6.5) | 1928.6 (1217.5-2524.5) | 5.6 (3.5-7.3) | 0.24 (-0.58-1.06) |
| Leukemia | Republic of Poland | 3185.8 (2974-3389.8) | 7.6 (7.1-8) | 5098 (4622.4-5612.3) | 7.6 (7-8.4) | -0.2 (-1.1-0.71) |
| Leukemia | Republic of Rwanda | 177.5 (129.9-239.7) | 3.1 (2.4-4.1) | 249.5 (155.1-380.8) | 2.7 (1.7-4) | -0.42 (-1-0.16) |
| Leukemia | Republic of San Marino | 5.7 (4.6-7.7) | 22.5 (17.6-30.1) | 6.3 (4-9.1) | 13 (8.2-18.5) | -0.71 (-1.9-0.49) |
| Leukemia | Republic of Senegal | 79.9 (61.6-101.8) | 1.1 (0.8-1.4) | 165.6 (81.8-253.2) | 1.4 (0.7-2) | 0.26 (-0.13-0.65) |
| Leukemia | Republic of Serbia | 596.3 (479.2-708.8) | 5.9 (4.8-7.1) | 1001.5 (757-1252.9) | 6.5 (4.9-8.2) | 0.05 (-0.8-0.91) |
| Leukemia | Republic of Seychelles | 4.3 (3.6-5.3) | 6.8 (5.9-8.6) | 6.2 (4.9-7.5) | 5.4 (4.4-6.6) | -0.24 (-0.96-0.5) |
| Leukemia | Republic of Sierra Leone | 43.6 (31.5-63) | 1 (0.8-1.3) | 94.4 (45.1-134.9) | 1.3 (0.6-1.9) | 0.29 (-0.08-0.67) |
| Leukemia | Republic of Singapore | 140.8 (132.4-149.6) | 5.5 (5.2-5.9) | 313.3 (287.8-338.7) | 5.1 (4.6-5.6) | -0.07 (-0.99-0.85) |
| Leukemia | Republic of Slovenia | 185.6 (171.7-198.8) | 8 (7.4-8.5) | 453.3 (376.4-535.7) | 11.1 (9.2-13.1) | 0.57 (-0.5-1.65) |
| Leukemia | Republic of South Africa | 869.6 (654.3-1028.2) | 3.3 (2.3-4.1) | 1786.9 (1261.2-2063.3) | 3.7 (2.6-4.3) | 0.18 (-0.61-0.97) |
| Leukemia | Republic of South Sudan | 116.8 (79.6-174.9) | 2.5 (1.8-3.5) | 224.1 (147.4-309.7) | 3.2 (2.1-4.3) | 0.36 (-0.2-0.92) |
| Leukemia | Republic of Sudan | 1105.3 (654.3-1656) | 6.9 (4.5-9.7) | 1931.8 (1204.4-2761.7) | 6.2 (3.8-8.9) | -0.17 (-1-0.66) |
| Leukemia | Republic of Suriname | 12.5 (9.3-14.6) | 3.9 (2.9-4.5) | 21.5 (16-27.7) | 3.6 (2.7-4.6) | -0.07 (-0.69-0.55) |
| Leukemia | Republic of Tajikistan | 205.3 (152.5-253) | 4.1 (3-5.1) | 254.7 (163.7-390.8) | 2.7 (1.8-4) | -0.74 (-1.39--0.08) |
| Leukemia | Republic of the Congo | 38.3 (28.5-51.4) | 2.4 (1.8-3) | 82.5 (53-115.7) | 2.3 (1.4-3.2) | -0.09 (-0.67-0.5) |
| Leukemia | Republic of the Gambia | 4.9 (3.2-6.5) | 0.6 (0.4-0.7) | 10.6 (7.1-14.8) | 0.6 (0.4-0.8) | 0 (-0.22-0.23) |
| Leukemia | Republic of the Marshall Islands | 1.1 (0.8-1.4) | 4.1 (2.9-5.3) | 1.8 (1.2-2.6) | 4.1 (2.7-5.7) | -0.02 (-0.85-0.81) |
| Leukemia | Republic of the Niger | 93.1 (61.3-131.8) | 1.1 (0.9-1.5) | 237.1 (107-372.9) | 1.2 (0.6-2) | 0.14 (-0.23-0.51) |
| Leukemia | Republic of the Philippines | 2588.5 (2114.6-3131.9) | 5.2 (4.1-6) | 4293.8 (3559.4-5105.4) | 4.4 (3.7-5.2) | -0.29 (-1.12-0.54) |
| Leukemia | Republic of the Union of Myanmar | 2438.8 (1405.2-3620.7) | 7.3 (4.6-10.4) | 2592.1 (1960.3-3367.5) | 5 (3.8-6.5) | -0.88 (-1.75-0.01) |
| Leukemia | Republic of Trinidad and Tobago | 49.6 (47.2-52) | 4.9 (4.7-5.2) | 73.4 (56.3-92.9) | 4.4 (3.4-5.5) | -0.25 (-0.94-0.46) |
| Leukemia | Republic of Tunisia | 252.6 (190-309.8) | 3.8 (2.8-4.8) | 442.3 (299.6-606.7) | 3.6 (2.4-4.9) | -0.16 (-0.83-0.51) |
| Leukemia | Republic of Turkey | 4240 (3123.4-5236.1) | 9.3 (7-11.2) | 6362.3 (4800.8-7849.6) | 7.3 (5.5-9) | -0.44 (-1.35-0.48) |
| Leukemia | Republic of Uganda | 258.4 (180.5-337.1) | 1.8 (1.3-2.3) | 622.9 (350.8-952.2) | 2 (1.3-2.8) | 0.02 (-0.45-0.5) |
| Leukemia | Republic of Uzbekistan | 870.7 (790.3-965.6) | 4.8 (4.4-5.3) | 1078.9 (887.2-1310.3) | 3.3 (2.7-4) | -0.6 (-1.27-0.07) |
| Leukemia | Republic of Vanuatu | 3.4 (2.1-4.7) | 3.6 (2.1-4.9) | 8.1 (5.3-10.5) | 3.5 (2.3-4.6) | -0.11 (-0.88-0.66) |
| Leukemia | Republic of Yemen | 589.1 (350-936.3) | 6.4 (3.9-9.5) | 1247.2 (689.4-1815) | 5.9 (3.2-8.7) | -0.19 (-0.96-0.59) |
| Leukemia | Republic of Zambia | 172.2 (128.8-236.5) | 2.7 (2.1-3.5) | 358.8 (219.1-512.3) | 2.8 (1.8-3.8) | 0.02 (-0.56-0.61) |
| Leukemia | Republic of Zimbabwe | 175.8 (129-218.7) | 3.1 (2.4-3.9) | 424.5 (298.2-561.5) | 4.3 (3.1-5.5) | 0.62 (-0.2-1.46) |
| Leukemia | Romania | 1185.3 (1130.8-1241.3) | 4.7 (4.5-5) | 1832.2 (1588.8-2071.5) | 5.8 (5-6.5) | 0.35 (-0.39-1.1) |
| Leukemia | Russian Federation | 9442.9 (9220.1-9670.8) | 5.7 (5.5-5.8) | 12559.8 (11629.6-13507.3) | 6 (5.6-6.4) | -0.07 (-0.79-0.65) |
| Leukemia | Saint Kitts and Nevis | 2 (1.9-2.2) | 5.3 (4.9-5.6) | 2.5 (2-2.9) | 4 (3.4-4.7) | -0.21 (-0.92-0.51) |
| Leukemia | Saint Lucia | 5.5 (5.1-5.9) | 5.2 (4.9-5.5) | 8.7 (7.1-10.5) | 4.1 (3.4-5) | -0.51 (-1.14-0.13) |
| Leukemia | Saint Vincent and the Grenadines | 5 (4.6-5.5) | 5.6 (5.2-6.1) | 6.3 (5.5-7.2) | 4.9 (4.3-5.6) | -0.2 (-0.88-0.48) |
| Leukemia | Slovak Republic | 408 (341.2-500.7) | 7 (5.9-8.6) | 646 (466.8-818.9) | 7.4 (5.4-9.5) | 0.05 (-0.91-1.01) |
| Leukemia | Socialist Republic of Viet Nam | 1728.2 (1353.3-2227) | 3.3 (2.6-4.2) | 2862.8 (2156.7-3853.9) | 2.9 (2.2-3.9) | -0.2 (-0.9-0.51) |
| Leukemia | Solomon Islands | 8.9 (4.4-13.2) | 4.1 (2-6.1) | 20.6 (12.7-28.1) | 4.1 (2.6-5.7) | -0.04 (-0.85-0.77) |
| Leukemia | State of Eritrea | 61.2 (42.6-88.6) | 2.5 (1.9-3.4) | 129.4 (79.3-185.5) | 2.9 (1.8-3.9) | 0.21 (-0.35-0.78) |
| Leukemia | State of Israel | 521.6 (482.8-559.1) | 10.7 (9.8-11.4) | 1177.4 (1053.8-1289.2) | 9.8 (8.9-10.8) | -0.15 (-1.27-0.99) |
| Leukemia | State of Kuwait | 66.4 (59.3-74.1) | 6.2 (5.6-6.8) | 146 (119.4-173.5) | 4.5 (3.7-5.4) | -0.59 (-1.35-0.18) |
| Leukemia | State of Libya | 198.9 (150.2-253.7) | 7.1 (5-9) | 444.8 (320-591.6) | 7.9 (5.6-10.5) | 0.47 (-0.43-1.38) |
| Leukemia | State of Qatar | 14.8 (9.9-18.8) | 8.2 (5.7-10.4) | 84.8 (52.5-118.8) | 6.2 (4.1-8.3) | -0.67 (-1.49-0.15) |
| Leukemia | Sultanate of Oman | 52.1 (37.2-70.3) | 4.6 (3.2-6.3) | 101.5 (74.2-133.2) | 3.9 (2.9-5.1) | -0.14 (-0.92-0.65) |
| Leukemia | Swiss Confederation | 1129.9 (1060.5-1192.1) | 13.4 (12.5-14.3) | 1520 (1325.3-1711.9) | 10.3 (9-11.5) | -0.65 (-1.67-0.39) |
| Leukemia | Syrian Arab Republic | 762.9 (581.6-966.5) | 8.8 (6.4-11) | 1027.3 (727.9-1401.3) | 7.8 (5.6-10.6) | -0.4 (-1.27-0.48) |
| Leukemia | Taiwan (Province of China) | 594.8 (567.2-623.1) | 3.3 (3.1-3.5) | 1655.2 (1496-1798.8) | 5.1 (4.7-5.6) | 0.82 (0-1.65) |
| Leukemia | Togolese Republic | 31.4 (23.2-40.2) | 1 (0.8-1.3) | 85 (40.3-124.3) | 1.4 (0.7-2) | 0.32 (-0.06-0.7) |
| Leukemia | Tokelau | 0.1 (0-0.1) | 4.1 (2.7-5.4) | 0.1 (0.1-0.1) | 6.3 (3.9-9) | -0.05 (-0.85-0.76) |
| Leukemia | Turkmenistan | 125.2 (115.3-135.8) | 3.8 (3.5-4.1) | 156.8 (125.7-196.6) | 3.2 (2.6-4) | -0.31 (-0.95-0.33) |
| Leukemia | Tuvalu | 0.4 (0.3-0.5) | 4.6 (3.3-6.1) | 0.4 (0.3-0.5) | 3.8 (2.6-4.8) | -0.3 (-1.12-0.52) |
| Leukemia | Ukraine | 4850.4 (4574.8-5138.5) | 8.1 (7.6-8.6) | 3360.3 (2479.5-4412.3) | 5.3 (4-6.8) | -0.96 (-1.7--0.22) |
| Leukemia | Union of the Comoros | 9.1 (6.3-12.5) | 2.5 (1.8-3.4) | 16.4 (11-23.2) | 2.7 (1.9-3.9) | 0.07 (-0.48-0.63) |
| Leukemia | United Arab Emirates | 68 (48.6-90.4) | 8.8 (6.1-12) | 298.5 (208.4-409.1) | 6.5 (4.9-8.5) | 0.09 (-0.8-0.98) |
| Leukemia | United Kingdom of Great Britain and Northern Ireland | 8571 (8235.1-8755.6) | 11 (10.7-11.3) | 9887.4 (9121.4-10334.9) | 8.8 (8.3-9.1) | -0.54 (-1.58-0.5) |
| Leukemia | United Mexican States | 3729 (3595.8-3882.1) | 5 (4.9-5.2) | 6560.3 (5920-7217.7) | 5.2 (4.7-5.7) | -0.05 (-0.9-0.82) |
| Leukemia | United Republic of Tanzania | 572.7 (455.1-730.8) | 2.6 (2.1-3.3) | 1169 (739.7-1717.1) | 2.7 (1.7-3.8) | 0.06 (-0.5-0.62) |
| Leukemia | United States of America | 40629.1 (38600.5-41740.2) | 13.5 (12.9-13.8) | 52060.4 (47500.1-54384.9) | 9.8 (9.1-10.2) | -0.85 (-1.95-0.26) |
| Leukemia | United States Virgin Islands | 4.5 (3.5-5.6) | 4.9 (3.7-6) | 4.8 (3.5-6.5) | 3.6 (2.6-4.7) | -0.35 (-1.16-0.46) |
| Leukemia | African Union | 14927.99 (11615.16-18924.99) | 3.07 (2.5-3.76) | 29015.06 (19700.36-35619.98) | 3.06 (2.13-3.73) | 0.11 (0.06-0.16) |
| Leukemia | Association of Southeast Asian Nations | 18376.45 (14551.22-23019.33) | 5.18 (4.19-6.37) | 30030.9 (24414.19-35677.71) | 4.66 (3.82-5.55) | -0.41 (-0.49--0.34) |
| Leukemia | Central Europe, Eastern Europe, and Central Asia | 28685.68 (28037.9-29477.35) | 6.37 (6.22-6.55) | 36029.07 (33655.22-38231.03) | 6.17 (5.77-6.55) | -0.13 (-0.19--0.06) |
| Leukemia | Commonwealth | 49746.97 (42152.01-57458.71) | 4.57 (4.01-5.1) | 78879.94 (66351.42-90046.86) | 3.67 (3.12-4.17) | -0.77 (-0.84--0.7) |
| Leukemia | European Union | 51051.85 (49078.05-52509.9) | 9.7 (9.38-9.98) | 75709.64 (68952.67-80924.07) | 9.31 (8.66-9.91) | -0.16 (-0.36-0.05) |
| Leukemia | Four World Regions | 311095.44 (278093.6-342605.79) | 6.89 (6.22-7.49) | 460600.93 (396848.73-503455.75) | 5.63 (4.83-6.17) | -0.65 (-0.71--0.59) |
| Leukemia | G20 | 248366.8 (225244.2-270263.88) | 7.62 (6.97-8.22) | 358076.11 (311579.46-391222.41) | 6.27 (5.44-6.93) | -0.63 (-0.69--0.57) |
| Leukemia | Gulf Cooperation Council | 557.94 (434.21-792.37) | 4.02 (3.09-6.01) | 1973.72 (1491.39-2835.16) | 4.81 (3.79-6.62) | 0.87 (0.62-1.12) |
| Leukemia | Health System Grouping Levels | 311341.14 (278334.46-342850.9) | 6.89 (6.22-7.49) | 460956.98 (397135.88-503892.37) | 5.63 (4.83-6.17) | -0.65 (-0.71--0.59) |
| Leukemia | High SDI | 102667.83 (98376.66-105202.07) | 10.09 (9.69-10.32) | 149127.61 (136650.81-157085.8) | 8.35 (7.72-8.81) | -0.63 (-0.79--0.48) |
| Leukemia | High-income | 113986.52 (109298.72-116779.87) | 10.54 (10.15-10.79) | 160114.31 (145531.61-169043.06) | 8.56 (7.99-8.98) | -0.73 (-0.9--0.55) |
| Leukemia | High-middle SDI | 77976.15 (68769.63-84291.03) | 7.75 (6.85-8.39) | 114756.89 (94868.14-129167.3) | 7.44 (5.88-8.62) | -0.03 (-0.08-0.02) |
| Leukemia | Latin America and Caribbean | 16196.19 (15541.67-17058.87) | 5.03 (4.83-5.24) | 29467.53 (26990.92-32195.32) | 4.92 (4.49-5.39) | -0.01 (-0.08-0.06) |
| Leukemia | Low SDI | 14164.64 (9791.41-19250.3) | 3.6 (2.71-4.53) | 23965.77 (17074.79-29871.21) | 3.05 (2.19-3.83) | -0.64 (-0.7--0.57) |
| Leukemia | Low-middle SDI | 34725.22 (27377.88-43155.84) | 3.68 (3.03-4.47) | 55187.48 (45809.43-65526.59) | 3.37 (2.8-4.03) | -0.27 (-0.3--0.24) |
| Leukemia | Middle SDI | 81807.29 (67136.72-95796.75) | 5.52 (4.61-6.39) | 117919.23 (94052.62-135713.05) | 4.72 (3.73-5.39) | -0.46 (-0.52--0.4) |
| Leukemia | Nordic Region | 3043.52 (2897.38-3159.34) | 9.53 (9.06-9.93) | 4252.25 (3859.38-4562.25) | 8.69 (7.99-9.34) | -0.28 (-0.4--0.15) |
| Leukemia | North Africa and Middle East | 16364.29 (12689.5-19966.29) | 6.46 (5.04-7.69) | 30995.66 (22754.41-36461.19) | 6.12 (4.51-7.17) | 0.04 (-0.06-0.14) |
| Leukemia | OECD Countries | 127793.34 (122820.6-130788.23) | 10.21 (9.81-10.45) | 182691.23 (167107.44-192265.13) | 8.39 (7.83-8.81) | -0.68 (-0.83--0.52) |
| Leukemia | Organization of Islamic Cooperation | 36655.13 (29308.74-44690.05) | 4.5 (3.65-5.38) | 64385.53 (50164.21-74349.1) | 4.18 (3.26-4.87) | -0.13 (-0.17--0.09) |
| Leukemia | Sahel Region | 2785.96 (1914.6-3858.67) | 2.23 (1.62-2.9) | 5983.81 (3694.42-7485.14) | 2.14 (1.33-2.67) | -0.07 (-0.11--0.04) |
| Leukemia | South Asia | 28964.59 (22480.77-35597.7) | 3.33 (2.66-3.99) | 45794.44 (37417.49-56236.82) | 2.85 (2.32-3.51) | -0.59 (-0.66--0.53) |
| Leukemia | Southeast Asia, East Asia, and Oceania | 97061.56 (76592.03-116144.78) | 6.44 (5.14-7.71) | 139870.12 (104013.57-169977.07) | 6.06 (4.44-7.26) | -0.06 (-0.15-0.03) |
| Leukemia | Sub-Saharan Africa | 10388.79 (7774.7-13670.66) | 2.72 (2.15-3.3) | 19151.6 (12646.3-24697.3) | 2.49 (1.7-3.17) | -0.31 (-0.33--0.29) |
| Leukemia | WHO region | 310498.91 (277485.34-342006.04) | 6.9 (6.23-7.5) | 458943.82 (395167.95-501728.75) | 5.63 (4.83-6.17) | -0.65 (-0.71--0.6) |
| Leukemia | World Bank Income Levels | 311340.74 (278334.18-342850.45) | 6.89 (6.22-7.49) | 460956.51 (397135.55-503891.79) | 5.63 (4.83-6.17) | -0.65 (-0.71--0.59) |
| Leukemia | World Bank Regions | 311238.1 (278250.88-342725.91) | 6.89 (6.22-7.49) | 460768.94 (396999.72-503661.81) | 5.63 (4.83-6.17) | -0.65 (-0.71--0.59) |
| Acute lymphoid leukemia | American Samoa | 0.1 (0-0.1) | 0.2 (0.1-0.3) | 0.1 (0-0.1) | 0.2 (0.1-0.3) | 0.28 (-0.53-1.1) |
| Acute lymphoid leukemia | Antigua and Barbuda | 0.5 (0.4-0.5) | 0.8 (0.7-0.9) | 0.6 (0.5-0.6) | 0.8 (0.6-0.9) | -0.02 (-0.8-0.76) |
| Acute lymphoid leukemia | Arab Republic of Egypt | 749.3 (492.6-1056.2) | 1.3 (0.8-1.8) | 1429.3 (616.6-2037.7) | 1.4 (0.6-2) | 1.14 (0.33-1.95) |
| Acute lymphoid leukemia | Argentine Republic | 495.3 (448.3-542.2) | 1.5 (1.3-1.6) | 579.3 (520.1-644.9) | 1.3 (1.2-1.5) | -0.36 (-1.12-0.4) |
| Acute lymphoid leukemia | Australia | 250.2 (227.8-277.5) | 1.6 (1.5-1.8) | 315 (269.7-361) | 1.5 (1.3-1.8) | -0.18 (-1.19-0.84) |
| Acute lymphoid leukemia | Barbados | 1.5 (1.3-1.7) | 0.6 (0.5-0.7) | 1.1 (0.9-1.5) | 0.4 (0.3-0.6) | 0.15 (-0.64-0.95) |
| Acute lymphoid leukemia | Belize | 2.7 (2.4-3) | 1.1 (1-1.2) | 3.3 (2.9-3.7) | 0.8 (0.7-0.9) | 0.09 (-0.54-0.71) |
| Acute lymphoid leukemia | Bermuda | 0.3 (0.2-0.4) | 0.5 (0.3-0.7) | 0.3 (0.2-0.5) | 0.9 (0.5-1.5) | -0.15 (-0.92-0.62) |
| Acute lymphoid leukemia | Bolivarian Republic of Venezuela | 334.2 (313.6-352.5) | 1.6 (1.5-1.7) | 476.5 (350.2-630.4) | 1.9 (1.4-2.5) | -0.04 (-0.78-0.72) |
| Acute lymphoid leukemia | Bosnia and Herzegovina | 38.2 (25.5-50.5) | 0.9 (0.6-1.2) | 24.8 (14.7-40.3) | 0.7 (0.4-1.1) | 0.51 (-0.29-1.31) |
| Acute lymphoid leukemia | Brunei Darussalam | 2.5 (1.6-3.6) | 1 (0.7-1.5) | 2.8 (1.6-3.7) | 0.7 (0.4-0.9) | -0.24 (-1.06-0.59) |
| Acute lymphoid leukemia | Burkina Faso | 57.3 (30.8-90.5) | 0.4 (0.3-0.6) | 140.8 (52.3-217) | 0.5 (0.2-0.7) | 0.29 (-0.1-0.67) |
| Acute lymphoid leukemia | Canada | 588.3 (491.5-698.8) | 2.6 (2.1-3.2) | 536.7 (471.8-616.6) | 1.9 (1.6-2.3) | -0.44 (-1.56-0.68) |
| Acute lymphoid leukemia | Central African Republic | 23.6 (12.8-41.3) | 0.8 (0.5-1.3) | 39.9 (20.7-65.1) | 0.7 (0.4-1.1) | -0.05 (-0.64-0.54) |
| Acute lymphoid leukemia | Commonwealth of Dominica | 1.2 (0.9-1.6) | 1.6 (1.2-2.2) | 1.3 (0.9-1.9) | 2.1 (1.5-2.9) | 0.22 (-0.54-0.98) |
| Acute lymphoid leukemia | Commonwealth of the Bahamas | 1.7 (1.5-1.9) | 0.6 (0.6-0.7) | 2 (1.6-2.4) | 0.5 (0.4-0.7) | -0.01 (-0.67-0.66) |
| Acute lymphoid leukemia | Cook Islands | 0 (0-0) | 0.2 (0.1-0.2) | 0 (0-0) | 0.1 (0.1-0.3) | -0.33 (-0.79-0.14) |
| Acute lymphoid leukemia | Czech Republic | 207.9 (184.7-233.8) | 1.9 (1.7-2.2) | 111.7 (89.2-138.8) | 1.1 (0.8-1.4) | -0.06 (-0.98-0.87) |
| Acute lymphoid leukemia | Democratic People's Republic of Korea | 430.5 (234.4-610.9) | 2.1 (1.1-3) | 518 (262.4-760) | 2 (1-2.9) | -0.03 (-0.83-0.78) |
| Acute lymphoid leukemia | Democratic Republic of Sao Tome and Principe | 0.5 (0.3-0.8) | 0.3 (0.2-0.4) | 0.4 (0.2-0.9) | 0.2 (0.1-0.4) | -0.05 (-0.34-0.24) |
| Acute lymphoid leukemia | Democratic Republic of the Congo | 260.3 (156.6-434.7) | 0.6 (0.4-0.9) | 472.9 (257.6-681.9) | 0.6 (0.3-0.9) | 0.05 (-0.47-0.57) |
| Acute lymphoid leukemia | Democratic Republic of Timor-Leste | 15.3 (6.9-27.3) | 1.8 (0.9-3) | 19.1 (11.3-27.2) | 1.4 (0.8-1.9) | -0.24 (-1.02-0.54) |
| Acute lymphoid leukemia | Democratic Socialist Republic of Sri Lanka | 271.5 (184.2-378.9) | 1.7 (1.2-2.4) | 236.9 (131.5-372.9) | 1.1 (0.6-1.7) | -0.53 (-1.32-0.27) |
| Acute lymphoid leukemia | Dominican Republic | 99.3 (68.9-135.2) | 1.2 (0.8-1.6) | 91.5 (56.8-132.5) | 0.9 (0.5-1.3) | 0.02 (-0.63-0.67) |
| Acute lymphoid leukemia | Eastern Republic of Uruguay | 34.3 (31.1-37.3) | 1.1 (1-1.2) | 37.4 (33-42.4) | 1.1 (1-1.3) | -0.12 (-0.95-0.72) |
| Acute lymphoid leukemia | Federal Democratic Republic of Ethiopia | 1844.3 (724.1-3419.1) | 3.5 (1.8-6.2) | 2234.8 (1441.8-3198.1) | 2 (1.3-2.9) | -1 (-1.91--0.09) |
| Acute lymphoid leukemia | Federal Democratic Republic of Nepal | 211.7 (113.9-364.1) | 1 (0.6-1.6) | 200.8 (131.5-297.6) | 0.7 (0.4-1) | -0.19 (-0.81-0.43) |
| Acute lymphoid leukemia | Federal Republic of Germany | 1155.9 (1013.6-1313.3) | 1.8 (1.5-2.2) | 1245.1 (1100.9-1405.2) | 2.2 (1.8-2.5) | -0.13 (-1.11-0.86) |
| Acute lymphoid leukemia | Federal Republic of Nigeria | 545.3 (280.3-905.5) | 0.4 (0.2-0.7) | 1252.7 (493.8-1827.3) | 0.4 (0.2-0.6) | 0.01 (-0.35-0.38) |
| Acute lymphoid leukemia | Federal Republic of Somalia | 75.4 (42.5-129.3) | 0.8 (0.5-1.3) | 175.8 (100.3-276.3) | 0.8 (0.5-1.3) | 0.2 (-0.37-0.78) |
| Acute lymphoid leukemia | Federated States of Micronesia | 0.5 (0.3-0.7) | 0.5 (0.3-0.7) | 0.3 (0.2-0.5) | 0.4 (0.2-0.5) | -0.23 (-1.09-0.63) |
| Acute lymphoid leukemia | Federative Republic of Brazil | 1597 (1476.2-1713.3) | 1 (1-1.1) | 1942.8 (1807-2098.5) | 0.9 (0.9-1) | -0.16 (-0.89-0.57) |
| Acute lymphoid leukemia | French Republic | 1031.7 (903-1204.3) | 2.1 (1.8-2.5) | 1126.5 (985-1283.7) | 2.3 (1.9-2.6) | 0.07 (-0.86-1) |
| Acute lymphoid leukemia | Gabonese Republic | 5.4 (3.4-7.7) | 0.6 (0.3-0.7) | 9.1 (4.8-13.9) | 0.5 (0.3-0.8) | 0.06 (-0.52-0.65) |
| Acute lymphoid leukemia | Georgia | 82.6 (70.7-94.8) | 1.6 (1.4-1.8) | 25.9 (22.5-29.6) | 0.7 (0.6-0.8) | -0.11 (-0.84-0.63) |
| Acute lymphoid leukemia | Grand Duchy of Luxembourg | 6.7 (5.9-7.6) | 2.2 (1.9-2.6) | 7.2 (6-8.7) | 1.6 (1.3-2) | -0.52 (-1.55-0.53) |
| Acute lymphoid leukemia | Greenland | 0.4 (0.2-0.6) | 0.9 (0.5-1.1) | 0.2 (0.1-0.4) | 0.4 (0.3-0.7) | -0.45 (-1.1-0.2) |
| Acute lymphoid leukemia | Grenada | 0.8 (0.6-0.9) | 0.8 (0.6-0.9) | 0.5 (0.5-0.6) | 0.6 (0.5-0.7) | 0.11 (-0.57-0.79) |
| Acute lymphoid leukemia | Guam | 0.8 (0.4-1.1) | 0.6 (0.3-0.8) | 0.5 (0.3-0.8) | 0.3 (0.2-0.5) | -0.18 (-0.97-0.61) |
| Acute lymphoid leukemia | Hashemite Kingdom of Jordan | 40.1 (24.6-54.2) | 1 (0.6-1.4) | 83.9 (45.7-124) | 0.7 (0.4-1.1) | -0.28 (-1.31-0.75) |
| Acute lymphoid leukemia | Hellenic Republic | 248.9 (213.6-284.9) | 3.1 (2.6-3.8) | 170.2 (150.2-191.4) | 2.5 (2-2.9) | -0.59 (-1.44-0.28) |
| Acute lymphoid leukemia | Hungary | 146.2 (129.7-164.3) | 1.4 (1.3-1.6) | 90.7 (70.3-118.2) | 1 (0.8-1.4) | -0.25 (-1.18-0.69) |
| Acute lymphoid leukemia | Independent State of Papua New Guinea | 19 (8.3-37.1) | 0.4 (0.2-0.8) | 42.8 (20.4-78.1) | 0.4 (0.2-0.7) | -0.2 (-0.93-0.53) |
| Acute lymphoid leukemia | Independent State of Samoa | 0.7 (0.4-1.1) | 0.4 (0.3-0.7) | 0.8 (0.4-1.1) | 0.4 (0.2-0.5) | -0.18 (-1.09-0.74) |
| Acute lymphoid leukemia | Ireland | 70.2 (60.7-82) | 2 (1.7-2.4) | 63.9 (54.5-75.3) | 1.6 (1.3-1.9) | -0.25 (-1.25-0.76) |
| Acute lymphoid leukemia | Islamic Republic of Afghanistan | 330 (132.7-724.8) | 3.4 (1.3-7.7) | 776.9 (369.8-1463) | 2.9 (1.2-6.1) | -0.13 (-1.06-0.81) |
| Acute lymphoid leukemia | Islamic Republic of Iran | 1440.5 (870.9-2020) | 2.2 (1.3-3.1) | 1161.9 (634.8-1502.6) | 1.5 (0.8-2) | -0.14 (-0.95-0.68) |
| Acute lymphoid leukemia | Islamic Republic of Mauritania | 9.2 (5.8-12.8) | 0.4 (0.2-0.5) | 19.3 (7.6-32.8) | 0.4 (0.2-0.6) | 0.19 (-0.17-0.56) |
| Acute lymphoid leukemia | Islamic Republic of Pakistan | 1205 (734.7-1784.6) | 1 (0.6-1.4) | 2244.2 (1536-3318.4) | 0.9 (0.6-1.4) | -0.1 (-0.81-0.61) |
| Acute lymphoid leukemia | Jamaica | 26.1 (22.4-30.5) | 1 (0.8-1.1) | 18.3 (14.2-23.6) | 0.7 (0.6-0.9) | 0.23 (-0.5-0.96) |
| Acute lymphoid leukemia | Japan | 2273 (2044-2571.8) | 2.4 (2-2.8) | 1694.9 (1573.2-1830.7) | 2 (1.8-2.2) | -0.42 (-1.32-0.48) |
| Acute lymphoid leukemia | Kingdom of Bahrain | 3.8 (2.3-5) | 0.9 (0.5-1.2) | 9.2 (5.3-14.4) | 0.7 (0.4-1.1) | -0.93 (-1.92-0.06) |
| Acute lymphoid leukemia | Kingdom of Belgium | 208 (174.8-245.3) | 2.7 (2.2-3.3) | 186.3 (158.1-221.1) | 2.3 (1.8-2.8) | -0.5 (-1.41-0.41) |
| Acute lymphoid leukemia | Kingdom of Bhutan | 5.9 (2.8-9.5) | 0.9 (0.5-1.4) | 5.2 (3.2-8.7) | 0.8 (0.5-1.2) | -0.28 (-0.94-0.38) |
| Acute lymphoid leukemia | Kingdom of Cambodia | 236.3 (114.3-430.7) | 2.2 (1.1-3.8) | 257.8 (145.1-375.4) | 1.6 (0.9-2.3) | -0.4 (-1.26-0.47) |
| Acute lymphoid leukemia | Kingdom of Denmark | 66 (59.8-73.7) | 1.5 (1.3-1.8) | 85.3 (73.1-100.4) | 2 (1.6-2.4) | -0.1 (-1.08-0.9) |
| Acute lymphoid leukemia | Kingdom of Eswatini | 5.9 (4.1-9.4) | 0.8 (0.6-1.3) | 9.9 (5.9-15) | 1 (0.6-1.5) | 0.38 (-0.47-1.24) |
| Acute lymphoid leukemia | Kingdom of Lesotho | 9.2 (5.5-13.7) | 0.7 (0.4-1) | 18.1 (12-26.1) | 1.1 (0.7-1.6) | 0.96 (0.16-1.75) |
| Acute lymphoid leukemia | Kingdom of Morocco | 93.1 (47.5-148.5) | 0.3 (0.2-0.6) | 90.1 (39.7-133.6) | 0.3 (0.1-0.4) | -0.02 (-0.4-0.35) |
| Acute lymphoid leukemia | Kingdom of Norway | 57.3 (51.1-64.1) | 1.6 (1.4-1.9) | 70.4 (62.7-78.1) | 1.7 (1.5-2) | 0.14 (-0.8-1.08) |
| Acute lymphoid leukemia | Kingdom of Saudi Arabia | 137.8 (93-211.6) | 0.8 (0.6-1.3) | 278.6 (182.2-454.4) | 0.9 (0.5-1.3) | 0.78 (0.15-1.41) |
| Acute lymphoid leukemia | Kingdom of Spain | 952.8 (839.4-1092) | 3.1 (2.6-3.8) | 823.9 (711.6-929.5) | 2.9 (2.4-3.4) | -0.38 (-1.32-0.58) |
| Acute lymphoid leukemia | Kingdom of Sweden | 205.6 (171.6-247.5) | 3.2 (2.6-4) | 153.1 (135.2-177.5) | 2 (1.7-2.4) | -0.5 (-1.52-0.54) |
| Acute lymphoid leukemia | Kingdom of Thailand | 722.9 (423-1026.6) | 1.4 (0.8-2) | 696.7 (365-945.2) | 1.3 (0.7-1.7) | -0.2 (-1.05-0.66) |
| Acute lymphoid leukemia | Kingdom of the Netherlands | 287.3 (242.3-339) | 2.5 (2-3) | 200.8 (176.6-230.8) | 1.5 (1.3-1.8) | -0.46 (-1.44-0.52) |
| Acute lymphoid leukemia | Kingdom of Tonga | 0.2 (0.1-0.3) | 0.2 (0.1-0.3) | 0.2 (0.1-0.3) | 0.2 (0.1-0.3) | -0.1 (-0.83-0.65) |
| Acute lymphoid leukemia | Kyrgyz Republic | 63.1 (54.8-71.3) | 1.3 (1.1-1.4) | 47 (38.5-56.3) | 0.7 (0.6-0.8) | -0.61 (-1.2--0.02) |
| Acute lymphoid leukemia | Lao People's Democratic Republic | 98 (42.6-184.6) | 2.2 (1.1-4.1) | 100.4 (59.3-142.7) | 1.4 (0.8-2.1) | -0.71 (-1.56-0.15) |
| Acute lymphoid leukemia | Lebanese Republic | 36.1 (23.3-56.3) | 1.2 (0.8-1.9) | 61.4 (36.9-95.1) | 1.2 (0.7-1.9) | 0.23 (-0.62-1.08) |
| Acute lymphoid leukemia | Malaysia | 307.6 (181.9-415.9) | 1.8 (1.1-2.3) | 422.5 (239.6-544.2) | 1.4 (0.8-1.8) | -0.14 (-0.97-0.7) |
| Acute lymphoid leukemia | Mongolia | 28.9 (20-41.8) | 1.2 (0.8-1.7) | 24.1 (15.2-33.2) | 0.7 (0.5-1) | -0.33 (-1.01-0.37) |
| Acute lymphoid leukemia | Montenegro | 8.7 (5.9-11.9) | 1.5 (1-2) | 5.1 (3.2-8.5) | 0.9 (0.5-1.4) | 0.28 (-0.62-1.2) |
| Acute lymphoid leukemia | New Zealand | 49.6 (44.5-55.6) | 1.6 (1.4-1.8) | 57.5 (50.2-65.5) | 1.4 (1.2-1.7) | -0.17 (-1.15-0.82) |
| Acute lymphoid leukemia | North Macedonia | 26.5 (18.4-34.7) | 1.4 (1-1.9) | 19.6 (11.1-30.8) | 0.9 (0.5-1.4) | 0.23 (-0.91-1.38) |
| Acute lymphoid leukemia | Northern Mariana Islands | 0.3 (0.1-0.4) | 0.8 (0.4-1.1) | 0.3 (0.1-0.3) | 0.6 (0.3-0.7) | -0.43 (-1.2-0.34) |
| Acute lymphoid leukemia | Palestine | 38.5 (25.8-55.8) | 1.6 (1.1-2.2) | 65.3 (44-94.8) | 1.2 (0.8-1.7) | -0.45 (-1.39-0.5) |
| Acute lymphoid leukemia | People's Democratic Republic of Algeria | 144.5 (87.7-207.6) | 0.5 (0.3-0.7) | 154.2 (87.3-226.1) | 0.4 (0.2-0.5) | -0.14 (-0.8-0.53) |
| Acute lymphoid leukemia | People's Republic of Bangladesh | 1503.6 (775.2-2628.2) | 1.2 (0.7-2) | 1249 (826.6-1792.1) | 0.8 (0.5-1.1) | -0.5 (-1.16-0.15) |
| Acute lymphoid leukemia | People's Republic of China | 38025.3 (26760.4-50354.2) | 3.4 (2.4-4.5) | 38570.9 (21149.3-50762.3) | 3.6 (2-5.1) | 0.02 (-0.91-0.96) |
| Acute lymphoid leukemia | Plurinational State of Bolivia | 217 (141-333.1) | 3.1 (2-4.7) | 264.9 (154.3-365.3) | 2.3 (1.4-3.2) | -0.32 (-1.21-0.57) |
| Acute lymphoid leukemia | Portuguese Republic | 168.5 (152.3-187.8) | 2 (1.7-2.3) | 141.7 (121.2-166.6) | 2.1 (1.7-2.6) | 0 (-0.92-0.93) |
| Acute lymphoid leukemia | Principality of Andorra | 1.6 (0.9-2.8) | 4.4 (2.3-7.9) | 1.4 (0.8-2.1) | 2.8 (1.7-4.2) | -0.58 (-1.69-0.54) |
| Acute lymphoid leukemia | Principality of Monaco | 1.3 (0.8-2) | 8.5 (4.6-13.5) | 1.9 (1.1-2.7) | 8.8 (4.9-13) | 0.43 (-0.8-1.67) |
| Acute lymphoid leukemia | Puerto Rico | 41.9 (38-46.8) | 1.2 (1.1-1.3) | 32.1 (26.4-37.9) | 1 (0.8-1.3) | -0.23 (-1.03-0.58) |
| Acute lymphoid leukemia | Republic of Albania | 41.4 (29.1-56.1) | 1.2 (0.9-1.7) | 24.9 (15.1-40) | 1.2 (0.7-1.8) | 0.18 (-0.57-0.94) |
| Acute lymphoid leukemia | Republic of Angola | 86.7 (44.5-157.7) | 0.8 (0.4-1.2) | 187.7 (101.2-269.2) | 0.6 (0.3-0.8) | -0.04 (-0.59-0.51) |
| Acute lymphoid leukemia | Republic of Armenia | 86.6 (77.1-96.8) | 2.5 (2.3-2.8) | 32.9 (28.2-38.1) | 1.2 (1-1.4) | -0.36 (-1.1-0.38) |
| Acute lymphoid leukemia | Republic of Austria | 110.1 (94.9-128.9) | 1.9 (1.5-2.3) | 96 (82.4-111.8) | 1.6 (1.3-1.9) | -0.08 (-1.07-0.91) |
| Acute lymphoid leukemia | Republic of Azerbaijan | 130.1 (89.1-171.6) | 1.7 (1.1-2.2) | 116.8 (72.9-178.9) | 1.3 (0.8-1.9) | -0.47 (-1.22-0.3) |
| Acute lymphoid leukemia | Republic of Belarus | 195.1 (169.8-222.6) | 2 (1.7-2.2) | 75.2 (59.4-92.5) | 0.9 (0.7-1.2) | -0.11 (-0.97-0.76) |
| Acute lymphoid leukemia | Republic of Benin | 31 (18.1-46.6) | 0.4 (0.3-0.6) | 83 (32.1-126.8) | 0.5 (0.2-0.7) | 0.23 (-0.15-0.62) |
| Acute lymphoid leukemia | Republic of Botswana | 8.8 (5.3-12.7) | 0.8 (0.4-1.2) | 14.3 (8.3-21) | 0.7 (0.4-1) | 0 (-0.74-0.73) |
| Acute lymphoid leukemia | Republic of Bulgaria | 117.2 (99.4-134) | 1.4 (1.2-1.6) | 44.9 (34.5-56) | 0.7 (0.5-0.9) | 0.23 (-0.51-0.98) |
| Acute lymphoid leukemia | Republic of Burundi | 62 (39.8-94.6) | 0.9 (0.6-1.3) | 97.7 (47.2-158.1) | 0.7 (0.3-1.1) | -0.1 (-0.65-0.45) |
| Acute lymphoid leukemia | Republic of Cabo Verde | 3.7 (2.5-5.4) | 0.8 (0.6-1.2) | 4.4 (2.5-6.1) | 0.8 (0.5-1.2) | 0.35 (-0.28-0.99) |
| Acute lymphoid leukemia | Republic of Cameroon | 58.5 (35.1-82) | 0.4 (0.3-0.6) | 177.2 (72.7-268) | 0.5 (0.2-0.7) | 0.27 (-0.14-0.68) |
| Acute lymphoid leukemia | Republic of Chad | 31.9 (17.3-50.6) | 0.4 (0.2-0.6) | 124.7 (52.3-204.5) | 0.5 (0.2-0.8) | 0.4 (0.03-0.78) |
| Acute lymphoid leukemia | Republic of Chile | 183.7 (171.3-197.2) | 1.4 (1.3-1.5) | 256.8 (229.3-290) | 1.5 (1.3-1.7) | 0.06 (-0.67-0.79) |
| Acute lymphoid leukemia | Republic of Colombia | 676.4 (620.2-734) | 2 (1.8-2.1) | 973.1 (814.5-1166.8) | 2.1 (1.8-2.6) | -0.05 (-0.85-0.76) |
| Acute lymphoid leukemia | Republic of Costa Rica | 56.6 (51.1-62.4) | 1.8 (1.7-2) | 114.3 (100.7-129.3) | 2.6 (2.2-3) | 0.26 (-0.56-1.08) |
| Acute lymphoid leukemia | République de Côte d’Ivoire | 45.5 (22.7-65) | 0.3 (0.2-0.4) | 88.4 (40.8-151.6) | 0.3 (0.1-0.4) | 0.02 (-0.28-0.32) |
| Acute lymphoid leukemia | Republic of Croatia | 63.9 (52.8-76.9) | 1.3 (1.1-1.6) | 37.2 (27.7-50.3) | 1.1 (0.8-1.6) | 0.2 (-0.77-1.17) |
| Acute lymphoid leukemia | Republic of Cuba | 137.3 (123.5-152) | 1.3 (1.2-1.5) | 109.1 (93.5-128.3) | 1.1 (0.9-1.3) | -0.16 (-0.92-0.61) |
| Acute lymphoid leukemia | Republic of Cyprus | 12.2 (8.7-18.4) | 1.7 (1.2-2.6) | 22.3 (12.7-31.2) | 2.1 (1.2-3) | 0.65 (-0.53-1.84) |
| Acute lymphoid leukemia | Republic of Djibouti | 3.3 (1.9-5) | 0.7 (0.4-1) | 8.2 (4.2-14) | 0.7 (0.3-1.1) | 0.27 (-0.3-0.83) |
| Acute lymphoid leukemia | Republic of Ecuador | 178.3 (165.2-191.6) | 1.7 (1.6-1.8) | 434.5 (357.1-510.8) | 2.5 (2-2.9) | 0.5 (-0.36-1.37) |
| Acute lymphoid leukemia | Republic of El Salvador | 126.5 (100.6-169.2) | 2.1 (1.7-2.8) | 130.1 (76.8-173) | 2 (1.2-2.7) | 0.24 (-0.59-1.07) |
| Acute lymphoid leukemia | Republic of Equatorial Guinea | 3.3 (1.8-5.5) | 0.7 (0.4-1.1) | 7.1 (3.1-12.1) | 0.5 (0.2-0.8) | -0.06 (-0.6-0.49) |
| Acute lymphoid leukemia | Republic of Estonia | 18.3 (16-20.7) | 1.3 (1.1-1.5) | 7.4 (6.2-8.9) | 0.6 (0.5-0.8) | -0.4 (-1.4-0.61) |
| Acute lymphoid leukemia | Republic of Fiji | 3 (0.6-5.1) | 0.4 (0.1-0.7) | 2.4 (0.7-4.1) | 0.3 (0.1-0.5) | -0.31 (-1.42-0.81) |
| Acute lymphoid leukemia | Republic of Finland | 58 (53.5-63.9) | 1.2 (1.1-1.4) | 79.3 (69.5-90.9) | 1.9 (1.5-2.2) | 0.09 (-0.78-0.98) |
| Acute lymphoid leukemia | Republic of Ghana | 177.1 (68.1-265.5) | 0.9 (0.4-1.3) | 176 (100.2-284.7) | 0.5 (0.3-0.7) | -0.72 (-1.21--0.23) |
| Acute lymphoid leukemia | Republic of Guatemala | 144.7 (128.8-162.8) | 1.4 (1.3-1.6) | 311.9 (265.7-363.8) | 2 (1.7-2.3) | 0.11 (-0.67-0.89) |
| Acute lymphoid leukemia | Republic of Guinea | 19 (9.3-28.7) | 0.2 (0.1-0.3) | 28 (11.3-53.5) | 0.2 (0.1-0.3) | -0.02 (-0.2-0.17) |
| Acute lymphoid leukemia | Republic of Guinea-Bissau | 7.2 (4-12) | 0.6 (0.3-0.9) | 11.8 (5.1-17.3) | 0.5 (0.2-0.7) | 0.26 (-0.18-0.7) |
| Acute lymphoid leukemia | Republic of Guyana | 8.6 (7.1-9.9) | 1 (0.9-1.2) | 9.9 (7.6-12.7) | 1.3 (1-1.7) | 0.19 (-0.46-0.84) |
| Acute lymphoid leukemia | Republic of Haiti | 183.3 (74-364) | 2.4 (1.1-4.6) | 238.4 (122.4-444.8) | 1.8 (1-3.3) | -0.31 (-1.05-0.44) |
| Acute lymphoid leukemia | Republic of Honduras | 133.9 (102-167.8) | 2.5 (1.8-3.1) | 164.1 (90.7-250.9) | 1.8 (1-2.6) | -0.11 (-0.91-0.69) |
| Acute lymphoid leukemia | Republic of Iceland | 5.7 (4.8-6.6) | 2.4 (2-2.8) | 6.5 (5.6-7.6) | 2.3 (1.9-2.7) | -0.09 (-1.04-0.86) |
| Acute lymphoid leukemia | Republic of India | 7785.9 (5312.4-11421.4) | 0.9 (0.6-1.3) | 7751.1 (4937.8-10617.6) | 0.6 (0.4-0.8) | -0.26 (-0.86-0.35) |
| Acute lymphoid leukemia | Republic of Indonesia | 2822.8 (1941.6-4209.4) | 1.5 (1-2.3) | 3382.9 (2017.4-4470.2) | 1.3 (0.8-1.8) | -0.1 (-0.93-0.74) |
| Acute lymphoid leukemia | Republic of Iraq | 285.6 (178.6-425.1) | 1.3 (0.8-1.9) | 462.1 (253.5-672.8) | 1.1 (0.6-1.6) | 0.05 (-0.81-0.92) |
| Acute lymphoid leukemia | Republic of Italy | 1558.4 (1391.8-1778) | 4 (3.4-4.7) | 1160.7 (1058.5-1281.2) | 3.1 (2.7-3.5) | -0.71 (-1.74-0.32) |
| Acute lymphoid leukemia | Republic of Kazakhstan | 202.3 (165.3-235) | 1.2 (1-1.4) | 126.2 (107.3-151.4) | 0.7 (0.6-0.8) | -0.33 (-1-0.35) |
| Acute lymphoid leukemia | Republic of Kenya | 129.5 (86.5-179.8) | 0.5 (0.3-0.7) | 240.1 (139.7-334.5) | 0.5 (0.3-0.7) | 0.32 (-0.15-0.79) |
| Acute lymphoid leukemia | Republic of Kiribati | 0.3 (0.2-0.5) | 0.4 (0.2-0.6) | 0.4 (0.2-0.6) | 0.4 (0.2-0.5) | -0.04 (-0.75-0.68) |
| Acute lymphoid leukemia | Republic of Korea | 652 (425.5-876.9) | 1.5 (1-2.1) | 562.8 (281.2-790.6) | 1.8 (0.8-2.7) | -0.01 (-0.86-0.85) |
| Acute lymphoid leukemia | Republic of Latvia | 35.8 (31.2-41) | 1.4 (1.2-1.7) | 11.8 (10-14) | 0.6 (0.5-0.8) | -0.47 (-1.42-0.49) |
| Acute lymphoid leukemia | Republic of Liberia | 15.5 (8.6-26.2) | 0.5 (0.3-0.7) | 28.5 (11.2-43.7) | 0.5 (0.2-0.7) | 0.28 (-0.09-0.66) |
| Acute lymphoid leukemia | Republic of Lithuania | 48.2 (42.6-54.8) | 1.4 (1.3-1.6) | 20.7 (17.4-24.2) | 0.7 (0.6-0.9) | -0.45 (-1.37-0.48) |
| Acute lymphoid leukemia | Republic of Madagascar | 102.9 (69-152) | 0.7 (0.5-1) | 187.3 (110.4-273.6) | 0.6 (0.4-0.9) | 0.05 (-0.44-0.54) |
| Acute lymphoid leukemia | Republic of Malawi | 48.1 (28.7-66.7) | 0.3 (0.2-0.5) | 50.5 (22.3-92.8) | 0.2 (0.1-0.4) | -0.06 (-0.36-0.23) |
| Acute lymphoid leukemia | Republic of Maldives | 3.6 (1.7-6.7) | 1.5 (0.8-2.6) | 3.8 (2.4-5.7) | 1 (0.6-1.4) | -0.85 (-1.59--0.1) |
| Acute lymphoid leukemia | Republic of Mali | 51 (23.8-75.1) | 0.4 (0.2-0.6) | 89.4 (39.2-164.9) | 0.3 (0.1-0.5) | -0.09 (-0.41-0.23) |
| Acute lymphoid leukemia | Republic of Malta | 5.8 (4.8-6.9) | 1.7 (1.4-2.1) | 7.7 (6.3-9.6) | 2.7 (2.1-3.4) | -0.05 (-0.94-0.85) |
| Acute lymphoid leukemia | Republic of Mauritius | 11.4 (10.6-12.3) | 1.1 (1-1.2) | 7.2 (6.6-7.8) | 0.6 (0.6-0.7) | -0.66 (-1.44-0.13) |
| Acute lymphoid leukemia | Republic of Moldova | 96.2 (83.4-106.9) | 2.2 (1.9-2.4) | 33.8 (29.3-39.3) | 1 (0.8-1.2) | -0.54 (-1.25-0.18) |
| Acute lymphoid leukemia | Republic of Mozambique | 255 (164.3-387.2) | 1.3 (0.9-2) | 345 (183.2-608.4) | 1 (0.6-1.5) | 0.18 (-0.48-0.84) |
| Acute lymphoid leukemia | Republic of Namibia | 7.7 (4.6-10.8) | 0.6 (0.4-0.8) | 13.5 (7.7-20.2) | 0.6 (0.3-0.9) | 0.04 (-0.59-0.67) |
| Acute lymphoid leukemia | Republic of Nauru | 0 (0-0.1) | 0.4 (0.3-0.7) | 0 (0-0.1) | 0.5 (0.3-0.8) | -0.17 (-1.1-0.77) |
| Acute lymphoid leukemia | Republic of Nicaragua | 105.7 (84.4-138.3) | 2.2 (1.8-2.9) | 103.7 (62.7-135) | 1.6 (1-2.1) | -0.02 (-0.79-0.74) |
| Acute lymphoid leukemia | Republic of Niue | 0 (0-0) | 0.3 (0.2-0.5) | 0 (0-0) | 1 (0.6-1.5) | 0.05 (-0.76-0.86) |
| Acute lymphoid leukemia | Republic of Palau | 0.1 (0-0.1) | 0.5 (0.3-0.7) | 0.1 (0-0.1) | 0.5 (0.3-0.7) | -0.06 (-0.58-0.46) |
| Acute lymphoid leukemia | Republic of Panama | 41.8 (37.9-46) | 1.6 (1.5-1.8) | 91.9 (75.4-111.6) | 2.2 (1.8-2.7) | 0.32 (-0.48-1.12) |
| Acute lymphoid leukemia | Republic of Paraguay | 66.3 (47.1-82.5) | 1.4 (1-1.8) | 103.3 (64.9-141.5) | 1.5 (0.9-2.1) | 0.42 (-0.32-1.16) |
| Acute lymphoid leukemia | Republic of Peru | 440.7 (338.6-627.2) | 1.9 (1.4-2.6) | 748.1 (379.1-1038.6) | 2.2 (1.1-3) | 0.24 (-0.58-1.06) |
| Acute lymphoid leukemia | Republic of Poland | 395.7 (339-457.8) | 1.1 (0.9-1.3) | 281.6 (250-315.2) | 0.8 (0.7-1) | -0.2 (-1.1-0.71) |
| Acute lymphoid leukemia | Republic of Rwanda | 89 (58.7-133.7) | 1 (0.7-1.5) | 95 (49.5-152.1) | 0.7 (0.4-1.1) | -0.42 (-1-0.16) |
| Acute lymphoid leukemia | Republic of San Marino | 1.7 (1.2-2.5) | 11.1 (7.3-16.6) | 1.2 (0.8-1.8) | 6 (3.6-9) | -0.71 (-1.9-0.49) |
| Acute lymphoid leukemia | Republic of Senegal | 48.4 (27.2-69.8) | 0.5 (0.3-0.6) | 76.4 (29.7-131.7) | 0.4 (0.2-0.7) | 0.26 (-0.13-0.65) |
| Acute lymphoid leukemia | Republic of Serbia | 104.9 (69.1-138.6) | 1.2 (0.8-1.6) | 66.2 (37.3-93.2) | 0.7 (0.4-1) | 0.05 (-0.8-0.91) |
| Acute lymphoid leukemia | Republic of Seychelles | 0.8 (0.5-1.1) | 1.2 (0.7-1.6) | 0.9 (0.4-1.2) | 0.7 (0.4-1) | -0.24 (-0.96-0.5) |
| Acute lymphoid leukemia | Republic of Sierra Leone | 25.8 (13.4-44.3) | 0.4 (0.2-0.7) | 50.6 (18.2-78.2) | 0.5 (0.2-0.7) | 0.29 (-0.08-0.67) |
| Acute lymphoid leukemia | Republic of Singapore | 40.9 (37.5-45.4) | 1.6 (1.4-1.8) | 78.1 (68.1-89) | 2.1 (1.7-2.4) | -0.07 (-0.99-0.85) |
| Acute lymphoid leukemia | Republic of Slovenia | 27 (24.1-29.6) | 1.5 (1.3-1.6) | 21.8 (18.4-25.9) | 1.2 (1-1.5) | 0.57 (-0.5-1.65) |
| Acute lymphoid leukemia | Republic of South Africa | 278.8 (199-376.4) | 0.8 (0.5-1.1) | 410.7 (242.3-532) | 0.8 (0.4-1) | 0.18 (-0.61-0.97) |
| Acute lymphoid leukemia | Republic of South Sudan | 56.1 (34.3-91.8) | 0.8 (0.5-1.2) | 106.4 (67.4-162.7) | 0.9 (0.6-1.4) | 0.36 (-0.2-0.92) |
| Acute lymphoid leukemia | Republic of Sudan | 483.1 (216.8-936.5) | 2.1 (0.9-4.1) | 722.5 (317.7-1112.7) | 1.6 (0.7-2.6) | -0.17 (-1-0.66) |
| Acute lymphoid leukemia | Republic of Suriname | 3.2 (1.8-4.1) | 0.8 (0.4-1) | 3.7 (2.4-5.1) | 0.7 (0.4-0.9) | -0.07 (-0.69-0.55) |
| Acute lymphoid leukemia | Republic of Tajikistan | 94.4 (58.8-129.2) | 1.5 (0.9-2) | 101.6 (57.7-168.7) | 0.9 (0.5-1.5) | -0.74 (-1.39--0.08) |
| Acute lymphoid leukemia | Republic of the Congo | 16 (10-25.2) | 0.7 (0.4-1) | 28.7 (15.7-41.2) | 0.6 (0.3-0.8) | -0.09 (-0.67-0.5) |
| Acute lymphoid leukemia | Republic of the Gambia | 3 (1.5-4.4) | 0.2 (0.1-0.3) | 5.3 (2.9-8.8) | 0.2 (0.1-0.3) | 0 (-0.22-0.23) |
| Acute lymphoid leukemia | Republic of the Marshall Islands | 0.1 (0.1-0.2) | 0.3 (0.2-0.5) | 0.2 (0.1-0.3) | 0.4 (0.2-0.5) | -0.02 (-0.85-0.81) |
| Acute lymphoid leukemia | Republic of the Niger | 61.9 (32-107.1) | 0.5 (0.3-0.8) | 143.2 (49.9-234.1) | 0.4 (0.2-0.7) | 0.14 (-0.23-0.51) |
| Acute lymphoid leukemia | Republic of the Philippines | 1023.6 (736.9-1426.6) | 1.5 (1.1-2) | 1255 (800.7-1553.6) | 1.1 (0.7-1.4) | -0.29 (-1.12-0.54) |
| Acute lymphoid leukemia | Republic of the Union of Myanmar | 964.4 (434.8-1839.9) | 2.3 (1.1-4.3) | 805 (510.2-1108.6) | 1.5 (0.9-2) | -0.88 (-1.75-0.01) |
| Acute lymphoid leukemia | Republic of Trinidad and Tobago | 13.1 (11.6-14.6) | 1.1 (0.9-1.2) | 11.4 (8.9-14.6) | 0.9 (0.7-1.1) | -0.25 (-0.94-0.46) |
| Acute lymphoid leukemia | Republic of Tunisia | 90.6 (50.9-125.8) | 1 (0.5-1.4) | 86.5 (33.8-136.2) | 0.8 (0.3-1.3) | -0.16 (-0.83-0.51) |
| Acute lymphoid leukemia | Republic of Turkey | 1380.8 (862.2-2073.8) | 2.3 (1.5-3.4) | 1160.7 (659.2-1553.4) | 1.6 (0.9-2.1) | -0.44 (-1.35-0.48) |
| Acute lymphoid leukemia | Republic of Uganda | 117.9 (70.3-173.8) | 0.5 (0.3-0.7) | 253.3 (122.5-418.1) | 0.5 (0.2-0.7) | 0.02 (-0.45-0.5) |
| Acute lymphoid leukemia | Republic of Uzbekistan | 334.9 (272.1-410.2) | 1.5 (1.2-1.8) | 323 (257.2-407.7) | 0.9 (0.7-1.2) | -0.6 (-1.27-0.07) |
| Acute lymphoid leukemia | Republic of Vanuatu | 0.4 (0.2-0.6) | 0.3 (0.2-0.4) | 0.8 (0.4-1.2) | 0.3 (0.2-0.4) | -0.11 (-0.88-0.66) |
| Acute lymphoid leukemia | Republic of Yemen | 254.4 (95.3-516.3) | 1.7 (0.6-3.5) | 428.6 (160.5-699.7) | 1.4 (0.4-2.3) | -0.19 (-0.96-0.59) |
| Acute lymphoid leukemia | Republic of Zambia | 89.9 (63.1-132.8) | 0.9 (0.6-1.3) | 131.7 (70.2-198.3) | 0.6 (0.3-0.9) | 0.02 (-0.56-0.61) |
| Acute lymphoid leukemia | Republic of Zimbabwe | 67.7 (39.3-89.4) | 0.8 (0.4-1) | 171.6 (97.6-240.5) | 1.2 (0.7-1.7) | 0.62 (-0.2-1.46) |
| Acute lymphoid leukemia | Romania | 299.4 (272.9-324.9) | 1.4 (1.2-1.5) | 195.2 (168.6-224.8) | 1 (0.9-1.2) | 0.35 (-0.39-1.1) |
| Acute lymphoid leukemia | Russian Federation | 2281.9 (2146.3-2402.7) | 1.6 (1.5-1.7) | 1484.6 (1387.7-1583) | 1.1 (1-1.2) | -0.07 (-0.79-0.65) |
| Acute lymphoid leukemia | Saint Kitts and Nevis | 0.4 (0.4-0.5) | 1 (0.9-1.1) | 0.3 (0.3-0.4) | 0.8 (0.6-1) | -0.21 (-0.92-0.51) |
| Acute lymphoid leukemia | Saint Lucia | 1.2 (1.1-1.4) | 0.8 (0.7-0.9) | 1 (0.8-1.3) | 0.7 (0.5-0.8) | -0.51 (-1.14-0.13) |
| Acute lymphoid leukemia | Saint Vincent and the Grenadines | 0.9 (0.8-1.1) | 0.7 (0.6-0.8) | 0.7 (0.6-0.8) | 0.7 (0.6-0.8) | -0.2 (-0.88-0.48) |
| Acute lymphoid leukemia | Slovak Republic | 55.6 (34.9-74.2) | 1.1 (0.7-1.4) | 40.5 (21.9-60.6) | 0.9 (0.5-1.3) | 0.05 (-0.91-1.01) |
| Acute lymphoid leukemia | Socialist Republic of Viet Nam | 854.5 (622.6-1111.9) | 1.4 (1-1.8) | 1154.3 (696.1-1558.2) | 1.2 (0.7-1.6) | -0.2 (-0.9-0.51) |
| Acute lymphoid leukemia | Solomon Islands | 1.3 (0.6-2.2) | 0.3 (0.2-0.6) | 2.2 (1.1-3.3) | 0.4 (0.2-0.5) | -0.04 (-0.85-0.77) |
| Acute lymphoid leukemia | State of Eritrea | 31.9 (18.4-51.3) | 0.8 (0.5-1.2) | 54.8 (30.2-86.9) | 0.8 (0.4-1.2) | 0.21 (-0.35-0.78) |
| Acute lymphoid leukemia | State of Israel | 105.1 (87.2-128.1) | 2 (1.7-2.5) | 150.3 (124.9-184.8) | 1.6 (1.3-2) | -0.15 (-1.27-0.99) |
| Acute lymphoid leukemia | State of Kuwait | 26.6 (22.5-31.8) | 1.6 (1.4-1.9) | 42.9 (33.3-55.1) | 1.3 (1-1.7) | -0.59 (-1.35-0.18) |
| Acute lymphoid leukemia | State of Libya | 69.1 (38.3-95.3) | 1.6 (0.8-2.2) | 130.5 (65.9-190.9) | 2.2 (1.1-3.2) | 0.47 (-0.43-1.38) |
| Acute lymphoid leukemia | State of Qatar | 3.2 (1.7-4.5) | 0.9 (0.5-1.2) | 16.2 (8.8-25.2) | 0.8 (0.4-1.2) | -0.67 (-1.49-0.15) |
| Acute lymphoid leukemia | Sultanate of Oman | 5.4 (2.7-8.9) | 0.3 (0.1-0.4) | 7.6 (3.4-12.2) | 0.2 (0.1-0.3) | -0.14 (-0.92-0.65) |
| Acute lymphoid leukemia | Swiss Confederation | 185.4 (160.5-215.2) | 3.8 (3.1-4.5) | 130.4 (113.4-153) | 2.3 (1.9-2.7) | -0.65 (-1.67-0.39) |
| Acute lymphoid leukemia | Syrian Arab Republic | 297.7 (169.5-442) | 2.2 (1.1-3.4) | 216.7 (116.5-305.9) | 1.6 (0.9-2.3) | -0.4 (-1.27-0.48) |
| Acute lymphoid leukemia | Taiwan (Province of China) | 137.3 (101-172.2) | 0.7 (0.5-0.9) | 210.9 (182.3-241.3) | 1.2 (1-1.5) | 0.82 (0-1.65) |
| Acute lymphoid leukemia | Togolese Republic | 19.2 (10.3-27.2) | 0.4 (0.2-0.5) | 39.4 (14.2-63.8) | 0.4 (0.2-0.7) | 0.32 (-0.06-0.7) |
| Acute lymphoid leukemia | Tokelau | 0 (0-0) | 0.4 (0.2-0.6) | 0 (0-0) | 1.1 (0.5-2.3) | -0.05 (-0.85-0.76) |
| Acute lymphoid leukemia | Turkmenistan | 46.3 (37.2-57.4) | 1.1 (0.9-1.3) | 42.1 (32.5-53.7) | 0.8 (0.6-1) | -0.31 (-0.95-0.33) |
| Acute lymphoid leukemia | Tuvalu | 0 (0-0.1) | 0.5 (0.3-0.8) | 0 (0-0.1) | 0.3 (0.2-0.5) | -0.3 (-1.12-0.52) |
| Acute lymphoid leukemia | Ukraine | 1180.8 (1009.7-1386.8) | 2.5 (2.1-2.9) | 455.6 (350.1-573.1) | 1.1 (0.9-1.4) | -0.96 (-1.7--0.22) |
| Acute lymphoid leukemia | Union of the Comoros | 4.4 (2.6-6.3) | 0.8 (0.5-1.1) | 5.6 (3.3-8.4) | 0.8 (0.4-1.1) | 0.07 (-0.48-0.63) |
| Acute lymphoid leukemia | United Arab Emirates | 16.8 (9.8-26.7) | 1.1 (0.6-1.8) | 37 (22.4-55.2) | 0.6 (0.4-0.9) | 0.09 (-0.8-0.98) |
| Acute lymphoid leukemia | United Kingdom of Great Britain and Northern Ireland | 1157.6 (1090.4-1232.9) | 2.6 (2.4-2.8) | 931.8 (882.2-983.2) | 1.9 (1.7-2) | -0.54 (-1.58-0.5) |
| Acute lymphoid leukemia | United Mexican States | 2005.1 (1900.4-2131) | 2.1 (2-2.2) | 3057 (2771.5-3384.5) | 2.4 (2.2-2.7) | -0.05 (-0.9-0.82) |
| Acute lymphoid leukemia | United Republic of Tanzania | 288.7 (204.6-391.2) | 0.9 (0.6-1.2) | 499.2 (274.6-745.5) | 0.7 (0.4-1.1) | 0.06 (-0.5-0.62) |
| Acute lymphoid leukemia | United States of America | 4968.2 (4807.3-5139) | 2.2 (2.1-2.3) | 4368.6 (4115.9-4608.6) | 1.6 (1.5-1.7) | -0.85 (-1.95-0.26) |
| Acute lymphoid leukemia | United States Virgin Islands | 0.8 (0.5-1.1) | 0.7 (0.5-1) | 0.3 (0.2-0.5) | 0.5 (0.3-0.8) | -0.35 (-1.16-0.46) |
| Acute lymphoid leukemia | African Union | 6824.46 (4613.31-10472.57) | 0.95 (0.65-1.41) | 11029.82 (6269.11-13943.2) | 0.77 (0.42-0.96) | -0.57 (-0.61--0.52) |
| Acute lymphoid leukemia | Association of Southeast Asian Nations | 7073.51 (4961.71-10213.1) | 1.6 (1.08-2.24) | 8155.59 (4984.55-10245.56) | 1.28 (0.79-1.59) | -0.71 (-0.76--0.66) |
| Acute lymphoid leukemia | Central Europe, Eastern Europe, and Central Asia | 6482.96 (6018.97-6942.87) | 1.59 (1.47-1.7) | 3907 (3617.39-4203.24) | 0.98 (0.9-1.07) | -1.58 (-1.72--1.43) |
| Acute lymphoid leukemia | Commonwealth | 15415.17 (11130.94-21398.59) | 0.97 (0.72-1.3) | 17705.74 (12194.48-22439.9) | 0.69 (0.47-0.88) | -1.09 (-1.16--1.02) |
| Acute lymphoid leukemia | European Union | 7561.33 (7237.2-7927.5) | 2.16 (2.05-2.29) | 6433.25 (6012.25-6956.42) | 2.01 (1.84-2.21) | -0.26 (-0.52--0.01) |
| Acute lymphoid leukemia | Four World Regions | 98037.45 (77788.71-123383.95) | 1.79 (1.42-2.24) | 103629.86 (71525.85-122401.32) | 1.37 (0.95-1.64) | -0.64 (-0.74--0.54) |
| Acute lymphoid leukemia | G20 | 74261.37 (59292.07-92193.46) | 2.03 (1.62-2.51) | 73461.75 (51163.54-87253.41) | 1.7 (1.18-2.09) | -0.32 (-0.46--0.18) |
| Acute lymphoid leukemia | Gulf Cooperation Council | 193.54 (138.86-280.88) | 0.85 (0.61-1.21) | 391.59 (266.98-590.28) | 0.78 (0.53-1.13) | 0.01 (-0.17-0.2) |
| Acute lymphoid leukemia | Health System Grouping Levels | 98081.54 (77836.05-123427.28) | 1.79 (1.42-2.24) | 103663.45 (71556.94-122442.46) | 1.37 (0.95-1.64) | -0.64 (-0.74--0.54) |
| Acute lymphoid leukemia | High SDI | 15130.96 (14589.34-15929.92) | 1.99 (1.91-2.1) | 14604.37 (13252.81-15745.21) | 1.78 (1.58-1.95) | -0.12 (-0.28-0.05) |
| Acute lymphoid leukemia | High-income | 17206.76 (16711.68-17804.53) | 2.24 (2.16-2.33) | 15359.96 (14479.86-16331.35) | 1.86 (1.73-2) | -0.46 (-0.67--0.25) |
| Acute lymphoid leukemia | High-middle SDI | 25273.07 (20189.2-30015.91) | 2.5 (2-2.99) | 26547.02 (16505.94-33499.48) | 2.63 (1.59-3.52) | 0.58 (0.38-0.79) |
| Acute lymphoid leukemia | Latin America and Caribbean | 6667.45 (6242.44-7294.86) | 1.58 (1.49-1.73) | 9460.56 (8401.41-10682.1) | 1.66 (1.46-1.88) | 0.47 (0.35-0.58) |
| Acute lymphoid leukemia | Low SDI | 6532.11 (3764.47-10835.76) | 1.15 (0.74-1.84) | 9618.5 (6035.57-12254.01) | 0.83 (0.51-1.05) | -1.16 (-1.22--1.1) |
| Acute lymphoid leukemia | Low-middle SDI | 13702.4 (9609.72-19653.45) | 1.1 (0.78-1.54) | 15861.62 (10874.01-19380.5) | 0.85 (0.58-1.06) | -0.75 (-0.8--0.7) |
| Acute lymphoid leukemia | Middle SDI | 37443 (27583.42-47979.67) | 2.15 (1.6-2.72) | 37031.94 (24451.88-44677.03) | 1.65 (1.09-2.02) | -0.64 (-0.8--0.48) |
| Acute lymphoid leukemia | Nordic Region | 393.09 (355.43-435.29) | 2.12 (1.87-2.41) | 394.71 (358.48-437.53) | 1.93 (1.73-2.19) | -0.42 (-0.63--0.2) |
| Acute lymphoid leukemia | North Africa and Middle East | 5930.18 (3789.45-8779.83) | 1.62 (0.99-2.4) | 7429.01 (4026.38-9319.14) | 1.24 (0.67-1.54) | -0.54 (-0.65--0.44) |
| Acute lymphoid leukemia | OECD Countries | 21657.69 (20818.25-22747.87) | 2.14 (2.05-2.25) | 20512.8 (19150.99-22016.16) | 1.85 (1.72-2.02) | -0.27 (-0.45--0.08) |
| Acute lymphoid leukemia | Organization of Islamic Cooperation | 14171.66 (9855.62-20280.42) | 1.25 (0.86-1.75) | 18649.91 (11469.96-22247.26) | 0.96 (0.59-1.15) | -0.68 (-0.74--0.62) |
| Acute lymphoid leukemia | Sahel Region | 1462.89 (812.89-2488.72) | 0.8 (0.45-1.33) | 3007.7 (1484.9-3987.1) | 0.65 (0.32-0.86) | -0.54 (-0.59--0.48) |
| Acute lymphoid leukemia | South Asia | 10712.02 (7071.01-16023.48) | 0.94 (0.65-1.35) | 11450.34 (7596.67-15732.81) | 0.65 (0.43-0.9) | -1.26 (-1.33--1.18) |
| Acute lymphoid leukemia | Southeast Asia, East Asia, and Oceania | 45965.07 (32252.53-61729.25) | 2.78 (1.98-3.71) | 47707.61 (27495.07-60585.21) | 2.59 (1.5-3.43) | 0.14 (-0.12-0.39) |
| Acute lymphoid leukemia | Sub-Saharan Africa | 5184.88 (3367.63-8128.69) | 0.91 (0.63-1.36) | 8412.73 (4971.8-10756.55) | 0.69 (0.41-0.88) | -0.89 (-0.91--0.86) |
| Acute lymphoid leukemia | WHO region | 97899.76 (77648.66-123247.16) | 1.79 (1.43-2.24) | 103418.69 (71321.3-122193.13) | 1.37 (0.95-1.64) | -0.65 (-0.75--0.55) |
| Acute lymphoid leukemia | World Bank Income Levels | 98081.5 (77836.01-123427.24) | 1.79 (1.42-2.24) | 103663.4 (71556.91-122442.39) | 1.37 (0.95-1.64) | -0.64 (-0.74--0.54) |
| Acute lymphoid leukemia | World Bank Regions | 98055.02 (77817.77-123398.25) | 1.79 (1.42-2.24) | 103643.82 (71547.15-122417.61) | 1.37 (0.95-1.64) | -0.64 (-0.74--0.54) |
| Acute myeloid leukemia | American Samoa | 0.8 (0.5-1) | 2.3 (1.2-3) | 0.6 (0.4-1) | 1.2 (0.9-2) | 0.28 (-0.53-1.1) |
| Acute myeloid leukemia | Antigua and Barbuda | 0.8 (0.7-0.9) | 1.4 (1.3-1.6) | 1.9 (1.8-2) | 1.9 (1.8-2) | -0.02 (-0.8-0.76) |
| Acute myeloid leukemia | Arab Republic of Egypt | 730.6 (463.6-1401.7) | 1.9 (1.2-3.7) | 2200.6 (1481.4-3159.5) | 2.9 (1.9-4.5) | 1.14 (0.33-1.95) |
| Acute myeloid leukemia | Argentine Republic | 629.5 (574.8-693.5) | 1.9 (1.8-2.1) | 1027.6 (940.1-1135.9) | 1.9 (1.8-2.1) | -0.36 (-1.12-0.4) |
| Acute myeloid leukemia | Australia | 616.8 (571.1-666.1) | 3.2 (3-3.5) | 2145.2 (1901.3-2393) | 4.9 (4.4-5.5) | -0.18 (-1.19-0.84) |
| Acute myeloid leukemia | Barbados | 4.9 (4.5-5.4) | 1.9 (1.7-2) | 10 (7.7-12.4) | 2.4 (1.9-3) | 0.15 (-0.64-0.95) |
| Acute myeloid leukemia | Belize | 0.5 (0.5-0.6) | 0.3 (0.3-0.4) | 1.9 (1.6-2.1) | 0.5 (0.4-0.6) | 0.09 (-0.54-0.71) |
| Acute myeloid leukemia | Bermuda | 1.7 (1.5-1.9) | 2.8 (2.5-3.2) | 2.3 (1.9-2.7) | 2.2 (1.8-2.6) | -0.15 (-0.92-0.62) |
| Acute myeloid leukemia | Bolivarian Republic of Venezuela | 200.4 (185.9-222.2) | 1.3 (1.2-1.4) | 456.9 (339.9-598.2) | 1.6 (1.2-2.1) | -0.04 (-0.78-0.72) |
| Acute myeloid leukemia | Bosnia and Herzegovina | 62.6 (47.5-89.1) | 1.5 (1.1-2.1) | 91.9 (59.1-127) | 1.6 (1-2.2) | 0.51 (-0.29-1.31) |
| Acute myeloid leukemia | Brunei Darussalam | 5.1 (3.9-7.6) | 2.9 (2.2-4.2) | 9.4 (7.1-12.8) | 2.3 (1.7-3) | -0.24 (-1.06-0.59) |
| Acute myeloid leukemia | Burkina Faso | 22.8 (10.8-45.7) | 0.3 (0.2-0.4) | 61.6 (27-91.6) | 0.4 (0.2-0.5) | 0.29 (-0.1-0.67) |
| Acute myeloid leukemia | Canada | 838.4 (787-891.4) | 2.7 (2.5-2.8) | 2142.3 (1923.8-2325.8) | 3.1 (2.8-3.4) | -0.44 (-1.56-0.68) |
| Acute myeloid leukemia | Central African Republic | 14.3 (6.7-29.6) | 0.8 (0.4-1.3) | 25.1 (13.1-40.6) | 0.7 (0.4-1.1) | -0.05 (-0.64-0.54) |
| Acute myeloid leukemia | Commonwealth of Dominica | 1.1 (0.8-1.4) | 1.7 (1.3-2.3) | 1.3 (1-1.9) | 1.8 (1.3-2.5) | 0.22 (-0.54-0.98) |
| Acute myeloid leukemia | Commonwealth of the Bahamas | 2.6 (2.3-2.9) | 1.2 (1-1.3) | 5.9 (4.7-7.4) | 1.4 (1.1-1.8) | -0.01 (-0.67-0.66) |
| Acute myeloid leukemia | Cook Islands | 0.1 (0.1-0.2) | 0.9 (0.5-1.2) | 0.2 (0.1-0.2) | 0.7 (0.4-1) | -0.33 (-0.79-0.14) |
| Acute myeloid leukemia | Czech Republic | 302 (281.6-327) | 2.3 (2.2-2.5) | 481.8 (410.9-565.2) | 2.4 (2-2.8) | -0.06 (-0.98-0.87) |
| Acute myeloid leukemia | Democratic People's Republic of Korea | 310.4 (194.5-507.8) | 1.6 (1-2.6) | 399.5 (250.4-719.8) | 1.3 (0.9-2.4) | -0.03 (-0.83-0.78) |
| Acute myeloid leukemia | Democratic Republic of Sao Tome and Principe | 0.3 (0.1-0.4) | 0.2 (0.2-0.3) | 0.3 (0.2-0.6) | 0.2 (0.1-0.4) | -0.05 (-0.34-0.24) |
| Acute myeloid leukemia | Democratic Republic of the Congo | 164.3 (80.8-277) | 0.7 (0.3-1) | 334.6 (188.3-472.2) | 0.6 (0.3-0.9) | 0.05 (-0.47-0.57) |
| Acute myeloid leukemia | Democratic Republic of Timor-Leste | 9.1 (4.2-17) | 1.8 (0.9-2.8) | 19.3 (12.7-26.8) | 1.8 (1.2-2.5) | -0.24 (-1.02-0.54) |
| Acute myeloid leukemia | Democratic Socialist Republic of Sri Lanka | 236.4 (151-319.5) | 1.7 (1.1-2.3) | 338.4 (205-479.1) | 1.3 (0.8-1.9) | -0.53 (-1.32-0.27) |
| Acute myeloid leukemia | Dominican Republic | 75.6 (53.1-110) | 1.2 (0.8-1.8) | 130.5 (92.5-197.3) | 1.2 (0.9-1.9) | 0.02 (-0.63-0.67) |
| Acute myeloid leukemia | Eastern Republic of Uruguay | 71.6 (65.3-78.3) | 2 (1.9-2.2) | 109.9 (97.3-122.4) | 2.4 (2.1-2.6) | -0.12 (-0.95-0.72) |
| Acute myeloid leukemia | Federal Democratic Republic of Ethiopia | 458 (185.3-930.1) | 1.2 (0.5-2.2) | 738.7 (396-1164.5) | 1 (0.5-1.6) | -1 (-1.91--0.09) |
| Acute myeloid leukemia | Federal Democratic Republic of Nepal | 134.8 (66.4-265.8) | 0.9 (0.5-1.5) | 241.4 (165.2-370.4) | 0.9 (0.6-1.4) | -0.19 (-0.81-0.43) |
| Acute myeloid leukemia | Federal Republic of Germany | 2412.2 (2180-2634.8) | 2.1 (2-2.3) | 5351.9 (4793.1-5888.1) | 2.9 (2.6-3.1) | -0.13 (-1.11-0.86) |
| Acute myeloid leukemia | Federal Republic of Nigeria | 211.7 (106.2-423.3) | 0.3 (0.2-0.5) | 456.1 (254.7-620.8) | 0.3 (0.2-0.4) | 0.01 (-0.35-0.38) |
| Acute myeloid leukemia | Federal Republic of Somalia | 24.8 (9-55.5) | 0.5 (0.2-0.9) | 57.9 (24.6-103.7) | 0.5 (0.2-0.7) | 0.2 (-0.37-0.78) |
| Acute myeloid leukemia | Federated States of Micronesia | 1.7 (0.9-2.5) | 2.2 (1.1-3.2) | 1.8 (1-2.6) | 2.1 (1.1-3) | -0.23 (-1.09-0.63) |
| Acute myeloid leukemia | Federative Republic of Brazil | 2102.7 (2032.1-2178.5) | 1.8 (1.7-1.8) | 4529.7 (4242.7-4752.5) | 1.9 (1.7-2) | -0.16 (-0.89-0.57) |
| Acute myeloid leukemia | French Republic | 1678.8 (1573.6-1795.4) | 2.2 (2.1-2.3) | 3782.5 (3220-4247.7) | 2.8 (2.5-3.1) | 0.07 (-0.86-1) |
| Acute myeloid leukemia | Gabonese Republic | 5.9 (3.3-8.3) | 0.8 (0.5-1.2) | 10.4 (6.1-15.3) | 0.8 (0.5-1.1) | 0.06 (-0.52-0.65) |
| Acute myeloid leukemia | Georgia | 75 (61.7-94.8) | 1.3 (1.1-1.7) | 95.3 (82.9-109.7) | 2 (1.7-2.3) | -0.11 (-0.84-0.63) |
| Acute myeloid leukemia | Grand Duchy of Luxembourg | 15.9 (14.9-16.9) | 3.2 (3-3.4) | 37.1 (33.3-41.2) | 3.6 (3.3-4) | -0.52 (-1.55-0.53) |
| Acute myeloid leukemia | Greenland | 0.7 (0.5-0.8) | 1.8 (1.2-2.2) | 0.8 (0.5-1.1) | 1.3 (0.7-1.8) | -0.45 (-1.1-0.2) |
| Acute myeloid leukemia | Grenada | 0.7 (0.6-0.9) | 0.9 (0.7-1.1) | 1.3 (1.1-1.4) | 1.2 (1-1.3) | 0.11 (-0.57-0.79) |
| Acute myeloid leukemia | Guam | 1.7 (1.3-2.3) | 1.7 (1.3-2.3) | 3.2 (2.1-4) | 1.8 (1.2-2.2) | -0.18 (-0.97-0.61) |
| Acute myeloid leukemia | Hashemite Kingdom of Jordan | 110.3 (82.6-144.8) | 5 (3.7-6.5) | 338.3 (235.6-447.2) | 3.7 (2.6-4.9) | -0.28 (-1.31-0.75) |
| Acute myeloid leukemia | Hellenic Republic | 332 (313.1-353.4) | 2.3 (2.2-2.5) | 778 (699.6-838.7) | 3.5 (3.2-3.7) | -0.59 (-1.44-0.28) |
| Acute myeloid leukemia | Hungary | 290.6 (271.3-310.9) | 2.1 (2-2.3) | 433.7 (365.7-518.3) | 2.5 (2.1-3) | -0.25 (-1.18-0.69) |
| Acute myeloid leukemia | Independent State of Papua New Guinea | 49.7 (19.3-76.4) | 1.6 (0.6-2.5) | 126.8 (61.3-194.2) | 1.6 (0.7-2.5) | -0.2 (-0.93-0.53) |
| Acute myeloid leukemia | Independent State of Samoa | 3.1 (1.8-4.3) | 2.5 (1.4-3.5) | 4 (2.5-5.6) | 2.4 (1.4-3.3) | -0.18 (-1.09-0.74) |
| Acute myeloid leukemia | Ireland | 81.7 (77-86.8) | 2 (1.9-2.1) | 163.8 (147-181.8) | 2.2 (2-2.4) | -0.25 (-1.25-0.76) |
| Acute myeloid leukemia | Islamic Republic of Afghanistan | 277.6 (114.3-572.1) | 3.6 (1.5-7) | 610.9 (297.5-1121) | 3.9 (1.8-6.9) | -0.13 (-1.06-0.81) |
| Acute myeloid leukemia | Islamic Republic of Iran | 1053.4 (621.5-1368.6) | 2.6 (1.7-3.4) | 1807.1 (1189.4-2304.3) | 2.2 (1.5-2.9) | -0.14 (-0.95-0.68) |
| Acute myeloid leukemia | Islamic Republic of Mauritania | 4.5 (2.7-8.4) | 0.3 (0.2-0.5) | 10.2 (5.7-14.1) | 0.3 (0.2-0.5) | 0.19 (-0.17-0.56) |
| Acute myeloid leukemia | Islamic Republic of Pakistan | 888.9 (551.7-1473.6) | 1 (0.6-1.6) | 2069.4 (1416-3272.1) | 1.1 (0.8-1.8) | -0.1 (-0.81-0.61) |
| Acute myeloid leukemia | Jamaica | 12.9 (11.1-15.3) | 0.6 (0.5-0.7) | 32.2 (24.5-42.2) | 1.1 (0.8-1.4) | 0.23 (-0.5-0.96) |
| Acute myeloid leukemia | Japan | 3503.1 (3347-3618.3) | 2.3 (2.2-2.3) | 6506.4 (5635-7006.6) | 2 (1.8-2.1) | -0.42 (-1.32-0.48) |
| Acute myeloid leukemia | Kingdom of Bahrain | 7 (4.6-9.3) | 2.8 (1.7-3.7) | 20.7 (13.4-32.5) | 2 (1.3-2.9) | -0.93 (-1.92-0.06) |
| Acute myeloid leukemia | Kingdom of Belgium | 342.9 (309.9-374.8) | 2.4 (2.2-2.6) | 699.9 (605.6-787.5) | 3.2 (2.8-3.5) | -0.5 (-1.41-0.41) |
| Acute myeloid leukemia | Kingdom of Bhutan | 4.2 (1.9-7.8) | 0.9 (0.5-1.6) | 6.4 (3.9-10.7) | 1 (0.6-1.6) | -0.28 (-0.94-0.38) |
| Acute myeloid leukemia | Kingdom of Cambodia | 172.4 (86.1-340.8) | 2.5 (1.3-4.2) | 351.3 (210.8-493.3) | 2.5 (1.5-3.4) | -0.4 (-1.26-0.47) |
| Acute myeloid leukemia | Kingdom of Denmark | 240.7 (225.7-256.5) | 3.3 (3.1-3.5) | 303.5 (267.9-340.3) | 2.7 (2.4-3) | -0.1 (-1.08-0.9) |
| Acute myeloid leukemia | Kingdom of Eswatini | 5.6 (3.6-9.7) | 1.4 (0.9-2.2) | 11.9 (6.9-21) | 1.6 (0.9-2.7) | 0.38 (-0.47-1.24) |
| Acute myeloid leukemia | Kingdom of Lesotho | 9.6 (6.1-15.7) | 1 (0.6-1.5) | 20.2 (12.8-37.3) | 1.5 (1-2.7) | 0.96 (0.16-1.75) |
| Acute myeloid leukemia | Kingdom of Morocco | 90 (59.7-145.8) | 0.5 (0.3-0.8) | 180.2 (118.7-288.7) | 0.5 (0.3-0.8) | -0.02 (-0.4-0.35) |
| Acute myeloid leukemia | Kingdom of Norway | 159.1 (150.4-166.9) | 2.6 (2.5-2.7) | 241.2 (216.4-261.6) | 2.5 (2.2-2.7) | 0.14 (-0.8-1.08) |
| Acute myeloid leukemia | Kingdom of Saudi Arabia | 83.2 (52.5-141.7) | 0.8 (0.6-1.5) | 359.2 (240.1-630.3) | 1.2 (0.8-2) | 0.78 (0.15-1.41) |
| Acute myeloid leukemia | Kingdom of Spain | 845.3 (789.7-898.6) | 1.7 (1.6-1.8) | 2052.2 (1771.8-2305.4) | 2.3 (2-2.5) | -0.38 (-1.32-0.58) |
| Acute myeloid leukemia | Kingdom of Sweden | 296.5 (274-318.9) | 2.2 (2-2.4) | 492 (425.4-556) | 2.3 (2-2.6) | -0.5 (-1.52-0.54) |
| Acute myeloid leukemia | Kingdom of Thailand | 1080.7 (748.5-1445.3) | 2.5 (1.7-3.2) | 3025.3 (1413.1-4199.1) | 3.3 (1.6-4.5) | -0.2 (-1.05-0.66) |
| Acute myeloid leukemia | Kingdom of the Netherlands | 468.3 (435.7-500.7) | 2.5 (2.3-2.7) | 937.4 (826.8-1048.5) | 2.8 (2.5-3.1) | -0.46 (-1.44-0.52) |
| Acute myeloid leukemia | Kingdom of Tonga | 1.2 (0.7-1.6) | 1.6 (0.9-2.1) | 1.7 (1-2.3) | 1.8 (1-2.4) | -0.1 (-0.83-0.65) |
| Acute myeloid leukemia | Kyrgyz Republic | 33.9 (28-41.9) | 0.8 (0.7-1) | 65.8 (54-79.1) | 1.1 (0.9-1.3) | -0.61 (-1.2--0.02) |
| Acute myeloid leukemia | Lao People's Democratic Republic | 70.4 (31.1-159.8) | 2.4 (1.1-4.6) | 125.6 (81-180) | 2.2 (1.4-3.1) | -0.71 (-1.56-0.15) |
| Acute myeloid leukemia | Lebanese Republic | 66.4 (44.3-112.3) | 2.8 (1.9-4.6) | 156.8 (114.8-211) | 2.6 (1.9-3.5) | 0.23 (-0.62-1.08) |
| Acute myeloid leukemia | Malaysia | 240.6 (148-312.9) | 1.8 (1.2-2.4) | 555.6 (393.7-759.5) | 1.8 (1.3-2.6) | -0.14 (-0.97-0.7) |
| Acute myeloid leukemia | Mongolia | 22.2 (13.4-36.9) | 1.3 (0.8-2) | 38.5 (26.4-51.3) | 1.3 (0.9-1.7) | -0.33 (-1.01-0.37) |
| Acute myeloid leukemia | Montenegro | 12 (9-16.5) | 1.9 (1.4-2.6) | 18.1 (12.7-25.3) | 2 (1.4-2.8) | 0.28 (-0.62-1.2) |
| Acute myeloid leukemia | New Zealand | 112.8 (102.4-121.4) | 2.9 (2.7-3.2) | 188.3 (165.8-208.4) | 2.4 (2.2-2.7) | -0.17 (-1.15-0.82) |
| Acute myeloid leukemia | North Macedonia | 44.7 (32.4-59) | 2.4 (1.7-3.1) | 68.7 (42-91.6) | 2.2 (1.4-3) | 0.23 (-0.91-1.38) |
| Acute myeloid leukemia | Northern Mariana Islands | 0.6 (0.3-0.9) | 2.1 (1.1-2.9) | 0.4 (0.3-1) | 0.9 (0.6-2) | -0.43 (-1.2-0.34) |
| Acute myeloid leukemia | Palestine | 12.1 (7.2-20) | 0.9 (0.5-1.4) | 26.9 (17.1-40) | 0.8 (0.5-1.1) | -0.45 (-1.39-0.5) |
| Acute myeloid leukemia | People's Democratic Republic of Algeria | 208.3 (129.5-290.7) | 1 (0.6-1.4) | 339.5 (224.1-537.3) | 0.9 (0.6-1.4) | -0.14 (-0.8-0.53) |
| Acute myeloid leukemia | People's Republic of Bangladesh | 868.1 (455.9-1726.2) | 1 (0.6-1.6) | 1308.1 (823-1932) | 0.9 (0.5-1.3) | -0.5 (-1.16-0.15) |
| Acute myeloid leukemia | People's Republic of China | 15309.3 (8205.9-24141.8) | 1.5 (0.8-2.2) | 17835.2 (11876.2-24800.4) | 1 (0.7-1.4) | 0.02 (-0.91-0.96) |
| Acute myeloid leukemia | Plurinational State of Bolivia | 98.5 (58.2-186) | 1.9 (1.2-3.4) | 207.9 (129-299.2) | 2 (1.3-3) | -0.32 (-1.21-0.57) |
| Acute myeloid leukemia | Portuguese Republic | 245 (227.4-262.6) | 2 (1.9-2.1) | 553.7 (483.4-618.5) | 2.5 (2.2-2.7) | 0 (-0.92-0.93) |
| Acute myeloid leukemia | Principality of Andorra | 2 (1.3-3.1) | 3.5 (2.2-5.5) | 3.7 (2.2-5.4) | 2.6 (1.5-3.8) | -0.58 (-1.69-0.54) |
| Acute myeloid leukemia | Principality of Monaco | 2.7 (1.9-3.9) | 4.5 (3.2-6.6) | 5.2 (3.1-6.9) | 6.3 (4-8.3) | 0.43 (-0.8-1.67) |
| Acute myeloid leukemia | Puerto Rico | 86.5 (80.4-93.8) | 2.4 (2.2-2.6) | 146.3 (119.3-173.7) | 2.6 (2.1-3.1) | -0.23 (-1.03-0.58) |
| Acute myeloid leukemia | Republic of Albania | 39 (27.8-59.1) | 1.6 (1.1-2.4) | 55.3 (34.6-81.7) | 1.4 (0.9-2.1) | 0.18 (-0.57-0.94) |
| Acute myeloid leukemia | Republic of Angola | 50.5 (22.6-106.7) | 0.7 (0.4-1.1) | 136.8 (73.7-202) | 0.7 (0.4-1) | -0.04 (-0.59-0.51) |
| Acute myeloid leukemia | Republic of Armenia | 30.3 (24-37.4) | 0.9 (0.8-1.2) | 53.1 (44.2-64.3) | 1.4 (1.1-1.6) | -0.36 (-1.1-0.38) |
| Acute myeloid leukemia | Republic of Austria | 216.1 (199.6-231.3) | 2 (1.9-2.2) | 437.7 (386.5-484.3) | 2.5 (2.3-2.8) | -0.08 (-1.07-0.91) |
| Acute myeloid leukemia | Republic of Azerbaijan | 107.3 (74.3-164.9) | 1.6 (1.1-2.4) | 145.6 (97.5-232.5) | 1.4 (0.9-2.2) | -0.47 (-1.22-0.3) |
| Acute myeloid leukemia | Republic of Belarus | 143.8 (119.1-182.1) | 1.2 (1-1.5) | 278.9 (224.6-339.1) | 2.1 (1.7-2.5) | -0.11 (-0.97-0.76) |
| Acute myeloid leukemia | Republic of Benin | 11.9 (5.9-25.2) | 0.3 (0.2-0.5) | 33.2 (16-45.8) | 0.3 (0.2-0.5) | 0.23 (-0.15-0.62) |
| Acute myeloid leukemia | Republic of Botswana | 8.9 (5.4-14.1) | 1.2 (0.8-1.8) | 20.3 (11.8-30.1) | 1.1 (0.7-1.7) | 0 (-0.74-0.73) |
| Acute myeloid leukemia | Republic of Bulgaria | 162.7 (149.9-177.2) | 1.5 (1.4-1.7) | 312.6 (251-382.6) | 2.6 (2.1-3.2) | 0.23 (-0.51-0.98) |
| Acute myeloid leukemia | Republic of Burundi | 24.9 (10-45.5) | 0.5 (0.2-0.9) | 43.4 (18.1-69.8) | 0.5 (0.2-0.8) | -0.1 (-0.65-0.45) |
| Acute myeloid leukemia | Republic of Cabo Verde | 2.3 (1.6-4.1) | 0.7 (0.5-1.3) | 4.3 (2.8-7.3) | 0.9 (0.6-1.5) | 0.35 (-0.28-0.99) |
| Acute myeloid leukemia | Republic of Cameroon | 26.5 (14.2-50) | 0.3 (0.2-0.5) | 82.6 (43-119.6) | 0.4 (0.2-0.5) | 0.27 (-0.14-0.68) |
| Acute myeloid leukemia | Republic of Chad | 11.7 (5.7-25.3) | 0.2 (0.1-0.4) | 40.6 (17.3-67.8) | 0.3 (0.2-0.5) | 0.4 (0.03-0.78) |
| Acute myeloid leukemia | Republic of Chile | 171.8 (160.8-182.4) | 1.5 (1.4-1.6) | 391.9 (355.9-428.3) | 1.7 (1.5-1.8) | 0.06 (-0.67-0.79) |
| Acute myeloid leukemia | Republic of Colombia | 336.9 (314.7-359.5) | 1.3 (1.2-1.4) | 877.2 (727.5-1036) | 1.7 (1.4-2) | -0.05 (-0.85-0.76) |
| Acute myeloid leukemia | Republic of Costa Rica | 39.3 (36.5-43.9) | 1.7 (1.6-1.9) | 128.6 (113.5-144.3) | 2.4 (2.2-2.7) | 0.26 (-0.56-1.08) |
| Acute myeloid leukemia | République de Côte d’Ivoire | 20.1 (10.3-32.4) | 0.2 (0.1-0.3) | 42.3 (23.9-63.2) | 0.2 (0.1-0.3) | 0.02 (-0.28-0.32) |
| Acute myeloid leukemia | Republic of Croatia | 98 (82.2-115.7) | 1.7 (1.4-2) | 208.1 (166.9-254.2) | 2.6 (2.1-3.1) | 0.2 (-0.77-1.17) |
| Acute myeloid leukemia | Republic of Cuba | 178.4 (166.3-193.9) | 1.7 (1.6-1.8) | 273.5 (234.8-316.6) | 1.7 (1.5-2) | -0.16 (-0.92-0.61) |
| Acute myeloid leukemia | Republic of Cyprus | 24.8 (18.6-37.4) | 3.2 (2.4-5.1) | 57.4 (35.4-74.1) | 2.8 (1.8-3.6) | 0.65 (-0.53-1.84) |
| Acute myeloid leukemia | Republic of Djibouti | 1.5 (0.7-2.3) | 0.5 (0.2-0.8) | 5 (2.5-8.3) | 0.5 (0.3-0.9) | 0.27 (-0.3-0.83) |
| Acute myeloid leukemia | Republic of Ecuador | 97.9 (90.1-108.1) | 1.3 (1.2-1.4) | 310.5 (245.9-384.5) | 1.8 (1.4-2.3) | 0.5 (-0.36-1.37) |
| Acute myeloid leukemia | Republic of El Salvador | 57.8 (38.7-91.1) | 1.3 (0.9-1.9) | 97.3 (59.5-128) | 1.5 (0.9-2) | 0.24 (-0.59-1.07) |
| Acute myeloid leukemia | Republic of Equatorial Guinea | 2.1 (1-3.9) | 0.7 (0.4-1.1) | 6.4 (3.3-10.3) | 0.7 (0.4-1.1) | -0.06 (-0.6-0.49) |
| Acute myeloid leukemia | Republic of Estonia | 27.2 (24-30.8) | 1.6 (1.4-1.8) | 46.7 (39.2-55) | 2.2 (1.9-2.5) | -0.4 (-1.4-0.61) |
| Acute myeloid leukemia | Republic of Fiji | 23.3 (14.2-30.5) | 4.3 (2.6-5.7) | 33.6 (18.6-46) | 4.1 (2.3-5.7) | -0.31 (-1.42-0.81) |
| Acute myeloid leukemia | Republic of Finland | 132.7 (121.9-144.2) | 2 (1.9-2.2) | 247.8 (215.8-280.3) | 2.2 (2-2.5) | 0.09 (-0.78-0.98) |
| Acute myeloid leukemia | Republic of Ghana | 70.5 (33-103) | 0.6 (0.3-0.8) | 96.8 (62.5-168.2) | 0.4 (0.2-0.7) | -0.72 (-1.21--0.23) |
| Acute myeloid leukemia | Republic of Guatemala | 66.1 (53.2-77.3) | 0.9 (0.7-1) | 148.1 (126.6-171.8) | 1.1 (0.9-1.3) | 0.11 (-0.67-0.89) |
| Acute myeloid leukemia | Republic of Guinea | 6.8 (3-14.8) | 0.1 (0.1-0.2) | 10.7 (5.5-17.1) | 0.1 (0.1-0.2) | -0.02 (-0.2-0.17) |
| Acute myeloid leukemia | Republic of Guinea-Bissau | 2.7 (1.3-6.1) | 0.4 (0.2-0.6) | 5.3 (2.9-7.4) | 0.4 (0.2-0.6) | 0.26 (-0.18-0.7) |
| Acute myeloid leukemia | Republic of Guyana | 1.6 (1.3-1.9) | 0.3 (0.2-0.3) | 2.9 (2.1-3.8) | 0.4 (0.3-0.5) | 0.19 (-0.46-0.84) |
| Acute myeloid leukemia | Republic of Haiti | 86.5 (43-185.3) | 1.6 (0.9-3) | 157.1 (87.7-256.4) | 1.5 (0.9-2.3) | -0.31 (-1.05-0.44) |
| Acute myeloid leukemia | Republic of Honduras | 53.2 (29.8-83.7) | 1.4 (0.9-2.1) | 116 (72.2-175.8) | 1.5 (0.9-2.3) | -0.11 (-0.91-0.69) |
| Acute myeloid leukemia | Republic of Iceland | 6.2 (5.5-6.7) | 2.2 (2-2.4) | 14.9 (12.9-17) | 2.7 (2.4-3.1) | -0.09 (-1.04-0.86) |
| Acute myeloid leukemia | Republic of India | 5289.5 (3302.3-8511.9) | 0.8 (0.5-1.2) | 11040.3 (8211.1-15171) | 0.9 (0.6-1.2) | -0.26 (-0.86-0.35) |
| Acute myeloid leukemia | Republic of Indonesia | 2773.2 (1801.3-4215.5) | 2 (1.3-2.9) | 5554.8 (3964.3-7575) | 2.2 (1.6-3) | -0.1 (-0.93-0.74) |
| Acute myeloid leukemia | Republic of Iraq | 224.6 (119-515.8) | 1.8 (0.9-3.8) | 558.3 (353.6-1034.7) | 1.8 (1.1-3.4) | 0.05 (-0.81-0.92) |
| Acute myeloid leukemia | Republic of Italy | 1588.9 (1501.6-1655.7) | 2 (1.9-2.1) | 3947 (3493.9-4267) | 2.9 (2.7-3.1) | -0.71 (-1.74-0.32) |
| Acute myeloid leukemia | Republic of Kazakhstan | 234.2 (194.6-286.6) | 1.5 (1.3-1.9) | 227.7 (186.9-272.7) | 1.2 (1-1.4) | -0.33 (-1-0.35) |
| Acute myeloid leukemia | Republic of Kenya | 57.8 (33.3-86) | 0.4 (0.2-0.6) | 160.1 (106.6-221.8) | 0.5 (0.3-0.7) | 0.32 (-0.15-0.79) |
| Acute myeloid leukemia | Republic of Kiribati | 0.8 (0.3-1.4) | 1.3 (0.5-2.2) | 1.5 (0.6-2.4) | 1.4 (0.6-2.3) | -0.04 (-0.75-0.68) |
| Acute myeloid leukemia | Republic of Korea | 831.6 (578-1151.6) | 2.2 (1.7-3.2) | 1688.6 (819.8-2146.2) | 2.1 (1-2.6) | -0.01 (-0.86-0.85) |
| Acute myeloid leukemia | Republic of Latvia | 70.4 (61.7-80.7) | 2.3 (2.1-2.6) | 57 (47.6-67) | 1.9 (1.6-2.2) | -0.47 (-1.42-0.49) |
| Acute myeloid leukemia | Republic of Liberia | 6.1 (2.7-14.7) | 0.3 (0.2-0.5) | 12.3 (5.7-17.7) | 0.3 (0.2-0.5) | 0.28 (-0.09-0.66) |
| Acute myeloid leukemia | Republic of Lithuania | 55.7 (49.1-63) | 1.4 (1.2-1.6) | 112.5 (94.9-130.1) | 2.4 (2.1-2.8) | -0.45 (-1.37-0.48) |
| Acute myeloid leukemia | Republic of Madagascar | 44.5 (21-66.4) | 0.5 (0.2-0.7) | 86.5 (42.9-123.2) | 0.4 (0.2-0.6) | 0.05 (-0.44-0.54) |
| Acute myeloid leukemia | Republic of Malawi | 17.2 (5.4-31.9) | 0.2 (0.1-0.3) | 22.6 (10.7-39) | 0.2 (0.1-0.2) | -0.06 (-0.36-0.23) |
| Acute myeloid leukemia | Republic of Maldives | 2.6 (1.5-5.3) | 2 (1.2-3.4) | 5.2 (3.3-7.6) | 1.3 (0.9-1.8) | -0.85 (-1.59--0.1) |
| Acute myeloid leukemia | Republic of Mali | 20 (7.9-40.8) | 0.3 (0.1-0.4) | 36.9 (20.2-56.9) | 0.2 (0.1-0.3) | -0.09 (-0.41-0.23) |
| Acute myeloid leukemia | Republic of Malta | 8.8 (7.9-9.7) | 2.1 (1.9-2.3) | 23.9 (20.8-27) | 2.7 (2.4-3.1) | -0.05 (-0.94-0.85) |
| Acute myeloid leukemia | Republic of Mauritius | 6.2 (5.7-6.9) | 0.7 (0.6-0.8) | 15.3 (13.9-16.4) | 0.9 (0.9-1) | -0.66 (-1.44-0.13) |
| Acute myeloid leukemia | Republic of Moldova | 79.2 (72-92.7) | 1.8 (1.6-2.1) | 72.2 (64.5-81.5) | 1.4 (1.2-1.6) | -0.54 (-1.25-0.18) |
| Acute myeloid leukemia | Republic of Mozambique | 99.5 (34.1-190.1) | 0.8 (0.3-1.3) | 171.6 (86.8-295.1) | 0.7 (0.4-1.1) | 0.18 (-0.48-0.84) |
| Acute myeloid leukemia | Republic of Namibia | 7.8 (5-11.6) | 0.9 (0.6-1.3) | 16.2 (10.4-24.9) | 0.9 (0.6-1.5) | 0.04 (-0.59-0.67) |
| Acute myeloid leukemia | Republic of Nauru | 0.2 (0.1-0.3) | 2.6 (1.4-3.9) | 0.2 (0.1-0.3) | 2.3 (1.2-3.6) | -0.17 (-1.1-0.77) |
| Acute myeloid leukemia | Republic of Nicaragua | 30 (18.8-46.4) | 0.9 (0.6-1.3) | 53.4 (34.8-70.8) | 0.9 (0.6-1.2) | -0.02 (-0.79-0.74) |
| Acute myeloid leukemia | Republic of Niue | 0 (0-0.1) | 1.9 (0.9-2.7) | 0.1 (0-0.1) | 3 (1.9-3.9) | 0.05 (-0.76-0.86) |
| Acute myeloid leukemia | Republic of Palau | 0.1 (0-0.1) | 0.5 (0.2-0.6) | 0.1 (0-0.1) | 0.4 (0.2-0.6) | -0.06 (-0.58-0.46) |
| Acute myeloid leukemia | Republic of Panama | 26.6 (24.3-29.3) | 1.3 (1.2-1.4) | 80.7 (65.2-97.4) | 1.9 (1.5-2.2) | 0.32 (-0.48-1.12) |
| Acute myeloid leukemia | Republic of Paraguay | 37.4 (26.6-52) | 1.2 (0.9-1.7) | 100.9 (66.6-136.5) | 1.6 (1.1-2.2) | 0.42 (-0.32-1.16) |
| Acute myeloid leukemia | Republic of Peru | 255.7 (170.1-386.6) | 1.5 (1-2.1) | 549 (309-750.9) | 1.6 (0.9-2.2) | 0.24 (-0.58-1.06) |
| Acute myeloid leukemia | Republic of Poland | 780.8 (707.4-907) | 1.9 (1.7-2.1) | 1361.9 (1230.2-1482.5) | 2.1 (1.9-2.2) | -0.2 (-1.1-0.71) |
| Acute myeloid leukemia | Republic of Rwanda | 37.3 (16.2-70.1) | 0.6 (0.3-1.1) | 52.8 (26.6-80.3) | 0.5 (0.3-0.8) | -0.42 (-1-0.16) |
| Acute myeloid leukemia | Republic of San Marino | 0.7 (0.5-1.1) | 2 (1.5-3.2) | 0.8 (0.4-1.3) | 1.2 (0.7-2) | -0.71 (-1.9-0.49) |
| Acute myeloid leukemia | Republic of Senegal | 18.2 (9.5-34.1) | 0.3 (0.2-0.5) | 40.2 (22-56.2) | 0.4 (0.2-0.5) | 0.26 (-0.13-0.65) |
| Acute myeloid leukemia | Republic of Serbia | 215.3 (155.5-287.7) | 2.1 (1.5-2.8) | 315.8 (194.1-407.3) | 2.1 (1.3-2.7) | 0.05 (-0.8-0.91) |
| Acute myeloid leukemia | Republic of Seychelles | 1.1 (0.7-1.4) | 1.7 (1.1-2.2) | 1.5 (0.9-2) | 1.4 (0.8-1.8) | -0.24 (-0.96-0.5) |
| Acute myeloid leukemia | Republic of Sierra Leone | 10 (4.6-23) | 0.3 (0.1-0.5) | 21.5 (10.1-30.6) | 0.3 (0.2-0.4) | 0.29 (-0.08-0.67) |
| Acute myeloid leukemia | Republic of Singapore | 60.8 (56.6-64.8) | 2.4 (2.2-2.6) | 156.7 (143-170.3) | 2 (1.9-2.2) | -0.07 (-0.99-0.85) |
| Acute myeloid leukemia | Republic of Slovenia | 31.6 (28.1-35.4) | 1.3 (1.2-1.5) | 70.6 (57.4-84) | 1.7 (1.4-2.1) | 0.57 (-0.5-1.65) |
| Acute myeloid leukemia | Republic of South Africa | 342.5 (214-474.1) | 1.3 (0.8-1.8) | 671 (442.8-911.8) | 1.3 (0.9-1.8) | 0.18 (-0.61-0.97) |
| Acute myeloid leukemia | Republic of South Sudan | 24.6 (10.1-46) | 0.5 (0.2-0.9) | 38.1 (16.9-64.1) | 0.6 (0.3-0.9) | 0.36 (-0.2-0.92) |
| Acute myeloid leukemia | Republic of Sudan | 338.2 (164.8-657.3) | 2.4 (1.2-4.2) | 716 (422.4-1106.6) | 2.5 (1.5-3.7) | -0.17 (-1-0.66) |
| Acute myeloid leukemia | Republic of Suriname | 3.8 (2.5-5) | 1.1 (0.8-1.5) | 7.2 (4.9-10) | 1.2 (0.8-1.6) | -0.07 (-0.69-0.55) |
| Acute myeloid leukemia | Republic of Tajikistan | 57.8 (34.7-85.6) | 1.2 (0.8-1.7) | 88.7 (51.3-142.4) | 1 (0.6-1.5) | -0.74 (-1.39--0.08) |
| Acute myeloid leukemia | Republic of the Congo | 13.3 (8-22.3) | 0.8 (0.5-1.3) | 28.3 (16.8-40.1) | 0.7 (0.4-1) | -0.09 (-0.67-0.5) |
| Acute myeloid leukemia | Republic of the Gambia | 1 (0.5-2) | 0.1 (0.1-0.2) | 2.4 (1.5-3.9) | 0.2 (0.1-0.3) | 0 (-0.22-0.23) |
| Acute myeloid leukemia | Republic of the Marshall Islands | 0.6 (0.3-0.8) | 1.8 (0.9-2.6) | 0.9 (0.5-1.3) | 2 (1-2.9) | -0.02 (-0.85-0.81) |
| Acute myeloid leukemia | Republic of the Niger | 19.1 (7.3-49.4) | 0.3 (0.1-0.5) | 48.9 (19.9-75.6) | 0.3 (0.1-0.5) | 0.14 (-0.23-0.51) |
| Acute myeloid leukemia | Republic of the Philippines | 1037.8 (758.8-1450) | 2.3 (1.8-3) | 2060.9 (1643.8-2651.3) | 2.1 (1.7-2.9) | -0.29 (-1.12-0.54) |
| Acute myeloid leukemia | Republic of the Union of Myanmar | 810.7 (414.2-1635.3) | 2.6 (1.4-4.8) | 1122.9 (796.7-1609.5) | 2.2 (1.6-3.1) | -0.88 (-1.75-0.01) |
| Acute myeloid leukemia | Republic of Trinidad and Tobago | 16.6 (15-18.3) | 1.6 (1.5-1.8) | 32.9 (24.9-42.1) | 1.9 (1.5-2.5) | -0.25 (-0.94-0.46) |
| Acute myeloid leukemia | Republic of Tunisia | 88.4 (63.4-142.7) | 1.4 (1-2.3) | 169.5 (111.1-275.3) | 1.3 (0.9-2.1) | -0.16 (-0.83-0.51) |
| Acute myeloid leukemia | Republic of Turkey | 1769.3 (1129.2-2458.9) | 4 (2.6-5.2) | 2605.4 (1826.5-3379.9) | 2.9 (2-3.7) | -0.44 (-1.35-0.48) |
| Acute myeloid leukemia | Republic of Uganda | 48.8 (25-80.6) | 0.4 (0.2-0.6) | 134.2 (72.5-206.8) | 0.4 (0.2-0.7) | 0.02 (-0.45-0.5) |
| Acute myeloid leukemia | Republic of Uzbekistan | 257.9 (201.3-316.7) | 1.4 (1.1-1.7) | 378.5 (310.2-467) | 1.1 (0.9-1.4) | -0.6 (-1.27-0.07) |
| Acute myeloid leukemia | Republic of Vanuatu | 1.7 (0.8-2.5) | 1.7 (0.7-2.4) | 4.2 (2.2-5.8) | 1.7 (0.9-2.4) | -0.11 (-0.88-0.66) |
| Acute myeloid leukemia | Republic of Yemen | 167.7 (78.1-312.5) | 2.2 (1-3.6) | 450.9 (239.1-671.2) | 2.3 (1.2-3.4) | -0.19 (-0.96-0.59) |
| Acute myeloid leukemia | Republic of Zambia | 37.2 (16.8-65.1) | 0.6 (0.3-0.9) | 94.5 (50.4-142.5) | 0.7 (0.4-1) | 0.02 (-0.56-0.61) |
| Acute myeloid leukemia | Republic of Zimbabwe | 65.8 (41.4-100.5) | 1.2 (0.8-1.9) | 149.5 (91.2-234.3) | 1.5 (1-2.5) | 0.62 (-0.2-1.46) |
| Acute myeloid leukemia | Romania | 302.3 (274.1-334.1) | 1.2 (1.1-1.3) | 576.2 (493.3-654.6) | 1.8 (1.6-2.1) | 0.35 (-0.39-1.1) |
| Acute myeloid leukemia | Russian Federation | 2233.1 (2020.8-2440.4) | 1.4 (1.2-1.5) | 2942.9 (2699.6-3189.6) | 1.4 (1.3-1.5) | -0.07 (-0.79-0.65) |
| Acute myeloid leukemia | Saint Kitts and Nevis | 0.1 (0.1-0.1) | 0.2 (0.2-0.3) | 0.2 (0.1-0.2) | 0.3 (0.2-0.3) | -0.21 (-0.92-0.51) |
| Acute myeloid leukemia | Saint Lucia | 1.3 (1.2-1.5) | 1.2 (1.1-1.4) | 2.6 (2.1-3.2) | 1.3 (1-1.5) | -0.51 (-1.14-0.13) |
| Acute myeloid leukemia | Saint Vincent and the Grenadines | 1.1 (1-1.2) | 1 (0.9-1.1) | 1.5 (1.2-1.7) | 1.2 (1-1.4) | -0.2 (-0.88-0.48) |
| Acute myeloid leukemia | Slovak Republic | 133.8 (105.2-196.2) | 2.3 (1.8-3.4) | 178.7 (105.2-238) | 2.1 (1.3-2.8) | 0.05 (-0.91-1.01) |
| Acute myeloid leukemia | Socialist Republic of Viet Nam | 506.1 (293-732.3) | 1.1 (0.6-1.5) | 1038.8 (688-1475.9) | 1 (0.7-1.5) | -0.2 (-0.9-0.51) |
| Acute myeloid leukemia | Solomon Islands | 4.3 (1.8-6.5) | 1.8 (0.7-2.7) | 10.2 (5.3-14.4) | 2 (1-2.8) | -0.04 (-0.85-0.77) |
| Acute myeloid leukemia | State of Eritrea | 11.9 (5.3-21.4) | 0.5 (0.2-0.8) | 26.4 (14.2-37.8) | 0.6 (0.3-0.8) | 0.21 (-0.35-0.78) |
| Acute myeloid leukemia | State of Israel | 136.5 (124.2-149.2) | 2.8 (2.5-3.1) | 358.5 (316.7-396) | 3 (2.6-3.3) | -0.15 (-1.27-0.99) |
| Acute myeloid leukemia | State of Kuwait | 14.5 (13-16.3) | 1.6 (1.4-1.8) | 47 (37.8-56.2) | 1.3 (1-1.6) | -0.59 (-1.35-0.18) |
| Acute myeloid leukemia | State of Libya | 76.9 (52.7-118.5) | 3 (2-4.7) | 172.6 (103.6-293.5) | 2.9 (1.8-4.8) | 0.47 (-0.43-1.38) |
| Acute myeloid leukemia | State of Qatar | 4.6 (2.8-6.3) | 2.3 (1.3-3.2) | 21.2 (13.9-33.3) | 1.4 (0.9-2.1) | -0.67 (-1.49-0.15) |
| Acute myeloid leukemia | Sultanate of Oman | 24.7 (15-34.8) | 1.9 (1-2.8) | 45.6 (30.5-61.3) | 1.5 (1-2) | -0.14 (-0.92-0.65) |
| Acute myeloid leukemia | Swiss Confederation | 308.8 (281.7-339.1) | 3.4 (3.1-3.8) | 546.7 (478.9-606.5) | 3.5 (3.1-3.9) | -0.65 (-1.67-0.39) |
| Acute myeloid leukemia | Syrian Arab Republic | 264.2 (170-422.7) | 3.3 (2-5) | 387.5 (256.3-605.3) | 2.8 (1.9-4.5) | -0.4 (-1.27-0.48) |
| Acute myeloid leukemia | Taiwan (Province of China) | 299.6 (262.4-350.9) | 1.7 (1.5-2) | 921.9 (835.5-1004) | 2.6 (2.3-2.8) | 0.82 (0-1.65) |
| Acute myeloid leukemia | Togolese Republic | 7.1 (3.7-12.6) | 0.3 (0.2-0.4) | 19.8 (10.1-28.1) | 0.3 (0.2-0.5) | 0.32 (-0.06-0.7) |
| Acute myeloid leukemia | Tokelau | 0 (0-0) | 1.9 (0.9-2.7) | 0 (0-0.1) | 3.4 (1.8-5.2) | -0.05 (-0.85-0.76) |
| Acute myeloid leukemia | Turkmenistan | 42.4 (34.9-49.7) | 1.2 (1-1.4) | 62.2 (46.9-81.5) | 1.2 (0.9-1.6) | -0.31 (-0.95-0.33) |
| Acute myeloid leukemia | Tuvalu | 0.2 (0.1-0.2) | 1.9 (0.9-2.8) | 0.2 (0.1-0.3) | 1.8 (1-2.5) | -0.3 (-1.12-0.52) |
| Acute myeloid leukemia | Ukraine | 1215.3 (1039.2-1391.7) | 2.1 (1.8-2.4) | 768.4 (569-1011.2) | 1.2 (0.9-1.6) | -0.96 (-1.7--0.22) |
| Acute myeloid leukemia | Union of the Comoros | 2 (0.8-2.9) | 0.5 (0.3-0.8) | 3.4 (1.9-5.1) | 0.5 (0.3-0.8) | 0.07 (-0.48-0.63) |
| Acute myeloid leukemia | United Arab Emirates | 27.4 (16.8-38.3) | 3.3 (1.8-4.6) | 133.2 (83.2-184.5) | 2.4 (1.5-3.2) | 0.09 (-0.8-0.98) |
| Acute myeloid leukemia | United Kingdom of Great Britain and Northern Ireland | 2235.1 (2158.2-2302.9) | 2.7 (2.6-2.8) | 3580.1 (3282.4-3732.4) | 2.9 (2.7-3) | -0.54 (-1.58-0.5) |
| Acute myeloid leukemia | United Mexican States | 969.1 (939.1-1007.1) | 1.4 (1.3-1.4) | 1988.3 (1753-2204.6) | 1.5 (1.4-1.7) | -0.05 (-0.9-0.82) |
| Acute myeloid leukemia | United Republic of Tanzania | 120.2 (53.8-186.2) | 0.6 (0.3-0.8) | 245.3 (125.8-360.8) | 0.5 (0.3-0.8) | 0.06 (-0.5-0.62) |
| Acute myeloid leukemia | United States of America | 10694 (10075.1-11004.9) | 3.5 (3.3-3.6) | 21533 (19545.6-22516.3) | 3.8 (3.5-4) | -0.85 (-1.95-0.26) |
| Acute myeloid leukemia | United States Virgin Islands | 1.5 (1.1-1.9) | 1.5 (1.1-1.9) | 1.2 (0.8-1.7) | 1.1 (0.7-1.5) | -0.35 (-1.16-0.46) |
| Acute myeloid leukemia | African Union | 3714.26 (2213.06-6152.82) | 0.83 (0.52-1.28) | 8132.6 (5169.84-10891.69) | 0.9 (0.59-1.25) | 0.45 (0.37-0.53) |
| Acute myeloid leukemia | Association of Southeast Asian Nations | 6757.97 (4630.24-10096.11) | 2.02 (1.38-2.85) | 14001.31 (9949.88-17030.6) | 2.16 (1.55-2.62) | 0.14 (0.07-0.2) |
| Acute myeloid leukemia | Central Europe, Eastern Europe, and Central Asia | 7201.37 (6743.28-7739.43) | 1.61 (1.5-1.72) | 9668.79 (9080.88-10302.86) | 1.69 (1.58-1.79) | 0.28 (0.2-0.37) |
| Acute myeloid leukemia | Commonwealth | 12676.53 (9236.87-17924.19) | 1.19 (0.92-1.53) | 26191.62 (21824.22-32500.77) | 1.23 (1.05-1.52) | 0.1 (0.04-0.15) |
| Acute myeloid leukemia | European Union | 11185.83 (10732.68-11609.01) | 2.05 (1.98-2.13) | 23705.43 (21423.87-25203.5) | 2.64 (2.45-2.79) | 1.05 (0.91-1.18) |
| Acute myeloid leukemia | Four World Regions | 79192.67 (62638.58-99236.88) | 1.77 (1.43-2.16) | 144338.35 (125955.3-164529.23) | 1.73 (1.51-1.98) | -0.03 (-0.12-0.06) |
| Acute myeloid leukemia | G20 | 61406.28 (49650.65-75034.33) | 1.89 (1.55-2.27) | 109855.26 (97755.46-121880.96) | 1.8 (1.6-2.01) | -0.12 (-0.23--0.02) |
| Acute myeloid leukemia | Gulf Cooperation Council | 161.42 (112.13-233.89) | 1.18 (0.84-1.7) | 626.84 (466.05-933.34) | 1.39 (1.02-1.93) | 0.85 (0.55-1.14) |
| Acute myeloid leukemia | Health System Grouping Levels | 79285.49 (62730.94-99331.43) | 1.77 (1.43-2.16) | 144492.34 (126102.51-164688.86) | 1.73 (1.51-1.98) | -0.03 (-0.12-0.06) |
| Acute myeloid leukemia | High SDI | 27830.53 (26575.12-28799.24) | 2.65 (2.54-2.75) | 56646.1 (51611.41-59717.06) | 2.88 (2.66-3.02) | 0.41 (0.25-0.56) |
| Acute myeloid leukemia | High-income | 29328.05 (28004.21-30138.76) | 2.61 (2.51-2.68) | 60538.95 (54578.02-63529.6) | 2.94 (2.7-3.06) | 0.52 (0.36-0.67) |
| Acute myeloid leukemia | High-middle SDI | 18663.36 (14113.97-22745.26) | 1.83 (1.39-2.23) | 29327.17 (24601.66-33129.91) | 1.69 (1.4-1.92) | -0.31 (-0.38--0.23) |
| Acute myeloid leukemia | Latin America and Caribbean | 4866.71 (4585.82-5240.7) | 1.55 (1.47-1.65) | 10485.51 (9689.61-11384.81) | 1.71 (1.58-1.86) | 0.38 (0.31-0.45) |
| Acute myeloid leukemia | Low SDI | 2992.44 (1361.17-5453.12) | 0.81 (0.41-1.25) | 6027.45 (3672.92-7907.26) | 0.79 (0.5-1.04) | -0.1 (-0.17--0.04) |
| Acute myeloid leukemia | Low-middle SDI | 9631.36 (6241.71-15986.1) | 1.06 (0.71-1.62) | 19061.92 (14795.45-25945.29) | 1.17 (0.92-1.6) | 0.34 (0.31-0.38) |
| Acute myeloid leukemia | Middle SDI | 20167.8 (13539.84-29687.38) | 1.41 (0.98-2.01) | 33429.7 (27391.59-41622.46) | 1.29 (1.06-1.61) | -0.4 (-0.46--0.34) |
| Acute myeloid leukemia | Nordic Region | 835.93 (794.49-871.9) | 2.47 (2.36-2.57) | 1300.18 (1175.01-1402.64) | 2.4 (2.2-2.56) | -0.04 (-0.13-0.05) |
| Acute myeloid leukemia | North Africa and Middle East | 5642.53 (3743.54-8176.22) | 2.37 (1.62-3.26) | 11358 (8396.46-15191.54) | 2.22 (1.66-3) | -0.07 (-0.15-0.02) |
| Acute myeloid leukemia | OECD Countries | 33318.05 (31902.51-34447.47) | 2.61 (2.5-2.7) | 67463.73 (61302.57-70842.19) | 2.86 (2.65-2.98) | 0.41 (0.28-0.54) |
| Acute myeloid leukemia | Organization of Islamic Cooperation | 11772.8 (7598.72-17345.92) | 1.54 (1.03-2.16) | 23178.56 (17601.75-30575.86) | 1.53 (1.15-2.04) | 0.03 (0-0.07) |
| Acute myeloid leukemia | Sahel Region | 684.15 (322.96-1390.36) | 0.64 (0.33-1.04) | 1543.01 (883.72-2191.55) | 0.63 (0.37-0.88) | 0.02 (-0.01-0.05) |
| Acute myeloid leukemia | South Asia | 7185.58 (4451.75-11929.88) | 0.84 (0.54-1.29) | 14665.52 (11306.31-20276.84) | 0.91 (0.68-1.25) | 0.15 (0.08-0.23) |
| Acute myeloid leukemia | Southeast Asia, East Asia, and Oceania | 22973.06 (12918.98-34333.67) | 1.58 (0.92-2.29) | 33589.83 (24225.71-42667.34) | 1.34 (0.97-1.69) | -0.75 (-0.87--0.62) |
| Acute myeloid leukemia | Sub-Saharan Africa | 2175.22 (1095.23-3558.39) | 0.61 (0.33-0.88) | 4339.01 (2567.48-5759.43) | 0.58 (0.36-0.76) | -0.13 (-0.16--0.11) |
| Acute myeloid leukemia | WHO region | 78892.39 (62353.98-98968.89) | 1.77 (1.43-2.16) | 143415.61 (125022.92-163633.73) | 1.73 (1.51-1.98) | -0.04 (-0.13-0.05) |
| Acute myeloid leukemia | World Bank Income Levels | 79285.28 (62730.82-99331.23) | 1.77 (1.43-2.16) | 144492.09 (126102.31-164688.6) | 1.73 (1.51-1.98) | -0.03 (-0.12-0.06) |
| Acute myeloid leukemia | World Bank Regions | 79240.55 (62701.75-99272.34) | 1.77 (1.43-2.16) | 144423.43 (126054.74-164611.16) | 1.73 (1.51-1.98) | -0.03 (-0.12-0.06) |
| Chronic lymphoid leukemia | American Samoa | 0 (0-0) | 0 (0-0.1) | 0 (0-0) | 0 (0-0) | 0.28 (-0.53-1.1) |
| Chronic lymphoid leukemia | Antigua and Barbuda | 0.4 (0.3-0.4) | 0.6 (0.6-0.7) | 0.7 (0.6-0.7) | 0.7 (0.6-0.7) | -0.02 (-0.8-0.76) |
| Chronic lymphoid leukemia | Arab Republic of Egypt | 153.9 (49.2-219.9) | 0.6 (0.2-0.9) | 970.6 (240.8-1665.9) | 1.7 (0.4-2.7) | 1.14 (0.33-1.95) |
| Chronic lymphoid leukemia | Argentine Republic | 290.1 (245.8-340.1) | 0.9 (0.8-1.1) | 388.2 (325.9-458.3) | 0.7 (0.6-0.8) | -0.36 (-1.12-0.4) |
| Chronic lymphoid leukemia | Australia | 652.4 (596.9-719.9) | 3.3 (3-3.6) | 1517.2 (1272.9-1758.8) | 3.2 (2.7-3.8) | -0.18 (-1.19-0.84) |
| Chronic lymphoid leukemia | Barbados | 1.9 (1.7-2.1) | 0.6 (0.6-0.7) | 4.9 (3.8-6.1) | 0.9 (0.7-1.2) | 0.15 (-0.64-0.95) |
| Chronic lymphoid leukemia | Belize | 0.5 (0.4-0.6) | 0.5 (0.5-0.6) | 2.3 (1.9-2.6) | 0.8 (0.7-0.9) | 0.09 (-0.54-0.71) |
| Chronic lymphoid leukemia | Bermuda | 0.6 (0.5-0.7) | 1 (0.8-1.1) | 1.6 (1.4-2) | 1.2 (1-1.4) | -0.15 (-0.92-0.62) |
| Chronic lymphoid leukemia | Bolivarian Republic of Venezuela | 44.1 (39.9-48.3) | 0.5 (0.4-0.5) | 153.8 (112-205.8) | 0.5 (0.4-0.7) | -0.04 (-0.78-0.72) |
| Chronic lymphoid leukemia | Bosnia and Herzegovina | 41.4 (27.5-65.6) | 1 (0.7-1.6) | 140.1 (82.6-210.2) | 2.2 (1.3-3.4) | 0.51 (-0.29-1.31) |
| Chronic lymphoid leukemia | Brunei Darussalam | 0.5 (0.3-0.7) | 0.5 (0.3-0.7) | 1.7 (0.8-2.6) | 0.5 (0.3-0.8) | -0.24 (-1.06-0.59) |
| Chronic lymphoid leukemia | Burkina Faso | 9.8 (5.2-13.6) | 0.3 (0.1-0.3) | 31.3 (9.9-44.2) | 0.4 (0.1-0.5) | 0.29 (-0.1-0.67) |
| Chronic lymphoid leukemia | Canada | 1675.4 (1549.7-1796.1) | 5.1 (4.7-5.5) | 2709.9 (2395.9-3039.9) | 3.6 (3.2-4) | -0.44 (-1.56-0.68) |
| Chronic lymphoid leukemia | Central African Republic | 6.7 (3.1-9.6) | 0.6 (0.3-0.9) | 13.9 (5-20.2) | 0.7 (0.3-1) | -0.05 (-0.64-0.54) |
| Chronic lymphoid leukemia | Commonwealth of Dominica | 0.5 (0.2-0.6) | 0.8 (0.4-1) | 0.8 (0.5-1.1) | 1 (0.6-1.3) | 0.22 (-0.54-0.98) |
| Chronic lymphoid leukemia | Commonwealth of the Bahamas | 1.6 (1.3-1.8) | 1 (0.9-1.1) | 4.5 (3.6-5.5) | 1.1 (0.9-1.4) | -0.01 (-0.67-0.66) |
| Chronic lymphoid leukemia | Cook Islands | 0 (0-0) | 0 (0-0.1) | 0 (0-0) | 0 (0-0) | -0.33 (-0.79-0.14) |
| Chronic lymphoid leukemia | Czech Republic | 274.4 (229.3-325.4) | 2 (1.6-2.3) | 860.9 (670.9-1039) | 3.8 (3-4.7) | -0.06 (-0.98-0.87) |
| Chronic lymphoid leukemia | Democratic People's Republic of Korea | 75.2 (37-115.9) | 0.4 (0.2-0.6) | 199.7 (107.4-326.2) | 0.6 (0.3-1) | -0.03 (-0.83-0.78) |
| Chronic lymphoid leukemia | Democratic Republic of Sao Tome and Principe | 0.1 (0.1-0.2) | 0.2 (0.1-0.3) | 0.3 (0.2-0.4) | 0.3 (0.1-0.4) | -0.05 (-0.34-0.24) |
| Chronic lymphoid leukemia | Democratic Republic of the Congo | 74.2 (34-107.8) | 0.5 (0.2-0.8) | 227 (85.5-365.1) | 0.7 (0.3-1.1) | 0.05 (-0.47-0.57) |
| Chronic lymphoid leukemia | Democratic Republic of Timor-Leste | 0.5 (0.2-0.9) | 0.2 (0.1-0.3) | 1.6 (0.7-2.9) | 0.2 (0.1-0.4) | -0.24 (-1.02-0.54) |
| Chronic lymphoid leukemia | Democratic Socialist Republic of Sri Lanka | 5.2 (1.6-14.5) | 0.1 (0-0.1) | 36.9 (18.6-66.8) | 0.1 (0.1-0.3) | -0.53 (-1.32-0.27) |
| Chronic lymphoid leukemia | Dominican Republic | 11.8 (6-17.2) | 0.3 (0.2-0.5) | 45.7 (27.8-71.1) | 0.5 (0.3-0.7) | 0.02 (-0.63-0.67) |
| Chronic lymphoid leukemia | Eastern Republic of Uruguay | 81.1 (70.4-91.8) | 2 (1.8-2.3) | 143.8 (124.3-164.3) | 2.5 (2.1-2.8) | -0.12 (-0.95-0.72) |
| Chronic lymphoid leukemia | Federal Democratic Republic of Ethiopia | 537 (318.1-790.8) | 3 (1.8-4.3) | 1004.7 (536.8-1701.8) | 2.5 (1.4-4.1) | -1 (-1.91--0.09) |
| Chronic lymphoid leukemia | Federal Democratic Republic of Nepal | 4.2 (1.6-8.5) | 0.1 (0-0.1) | 14.7 (6.4-27.4) | 0.1 (0-0.1) | -0.19 (-0.81-0.43) |
| Chronic lymphoid leukemia | Federal Republic of Germany | 3787.2 (3186.9-4374.4) | 2.9 (2.5-3.4) | 6497.2 (5426.9-7512.9) | 3.2 (2.7-3.6) | -0.13 (-1.11-0.86) |
| Chronic lymphoid leukemia | Federal Republic of Nigeria | 85.7 (45.6-118) | 0.2 (0.1-0.3) | 221 (101.5-304) | 0.3 (0.1-0.4) | 0.01 (-0.35-0.38) |
| Chronic lymphoid leukemia | Federal Republic of Somalia | 15.4 (8.6-23.6) | 0.7 (0.4-1.1) | 48.3 (23.9-74.6) | 0.9 (0.5-1.3) | 0.2 (-0.37-0.78) |
| Chronic lymphoid leukemia | Federated States of Micronesia | 0 (0-0.1) | 0.1 (0-0.2) | 0 (0-0) | 0 (0-0) | -0.23 (-1.09-0.63) |
| Chronic lymphoid leukemia | Federative Republic of Brazil | 433.9 (401.7-466.8) | 0.5 (0.5-0.6) | 1727.2 (1565.4-1865) | 0.7 (0.6-0.8) | -0.16 (-0.89-0.57) |
| Chronic lymphoid leukemia | French Republic | 2887.7 (2598.9-3151.3) | 3.4 (3.1-3.7) | 5301.3 (4308.4-6438.6) | 3.5 (2.8-4.2) | 0.07 (-0.86-1) |
| Chronic lymphoid leukemia | Gabonese Republic | 3.4 (1.6-4.5) | 0.6 (0.3-0.8) | 8.5 (3.8-13) | 0.9 (0.4-1.3) | 0.06 (-0.52-0.65) |
| Chronic lymphoid leukemia | Georgia | 66.9 (48.2-84.9) | 1.1 (0.8-1.3) | 66.3 (51.8-83.7) | 1.2 (0.9-1.5) | -0.11 (-0.84-0.63) |
| Chronic lymphoid leukemia | Grand Duchy of Luxembourg | 20.9 (19.2-22.7) | 3.8 (3.5-4.1) | 39.2 (34.2-44.5) | 3.6 (3.1-4.1) | -0.52 (-1.55-0.53) |
| Chronic lymphoid leukemia | Greenland | 0.1 (0-0.2) | 0.3 (0.1-0.5) | 0.3 (0.1-0.5) | 0.4 (0.1-0.8) | -0.45 (-1.1-0.2) |
| Chronic lymphoid leukemia | Grenada | 0.8 (0.7-1) | 1.1 (1-1.4) | 1.7 (1.5-2) | 1.6 (1.4-1.8) | 0.11 (-0.57-0.79) |
| Chronic lymphoid leukemia | Guam | 0.1 (0-0.3) | 0.1 (0-0.3) | 0 (0-0) | 0 (0-0) | -0.18 (-0.97-0.61) |
| Chronic lymphoid leukemia | Hashemite Kingdom of Jordan | 5.1 (2.5-10.7) | 0.4 (0.2-0.9) | 74.1 (32.1-114.7) | 1.1 (0.5-1.6) | -0.28 (-1.31-0.75) |
| Chronic lymphoid leukemia | Hellenic Republic | 494.6 (452.6-536.3) | 3.2 (2.9-3.5) | 751.6 (665.2-829.1) | 2.9 (2.6-3.2) | -0.59 (-1.44-0.28) |
| Chronic lymphoid leukemia | Hungary | 422.6 (387.4-455.1) | 2.8 (2.6-3) | 694.4 (592.7-805.3) | 3.6 (3-4.1) | -0.25 (-1.18-0.69) |
| Chronic lymphoid leukemia | Independent State of Papua New Guinea | 0.8 (0-2.4) | 0 (0-0.1) | 0.2 (0.1-0.5) | 0 (0-0) | -0.2 (-0.93-0.53) |
| Chronic lymphoid leukemia | Independent State of Samoa | 0.1 (0-0.3) | 0.1 (0-0.3) | 0 (0-0.1) | 0 (0-0.1) | -0.18 (-1.09-0.74) |
| Chronic lymphoid leukemia | Ireland | 152.6 (141.3-165.8) | 3.6 (3.4-3.9) | 254 (220.6-291) | 3.1 (2.7-3.6) | -0.25 (-1.25-0.76) |
| Chronic lymphoid leukemia | Islamic Republic of Afghanistan | 65.8 (11.3-119) | 1.1 (0.2-1.9) | 106 (21.5-199.1) | 1.2 (0.3-2.2) | -0.13 (-1.06-0.81) |
| Chronic lymphoid leukemia | Islamic Republic of Iran | 297.1 (114.7-415.9) | 1.2 (0.5-1.7) | 1279.4 (461.7-1677.7) | 1.7 (0.6-2.2) | -0.14 (-0.95-0.68) |
| Chronic lymphoid leukemia | Islamic Republic of Mauritania | 2.7 (1.5-3.7) | 0.3 (0.2-0.4) | 9.4 (3.6-14) | 0.5 (0.2-0.7) | 0.19 (-0.17-0.56) |
| Chronic lymphoid leukemia | Islamic Republic of Pakistan | 37.5 (13.4-68.6) | 0.1 (0-0.1) | 78.8 (39.8-144.6) | 0.1 (0-0.1) | -0.1 (-0.81-0.61) |
| Chronic lymphoid leukemia | Jamaica | 4.6 (3.7-5.6) | 0.3 (0.2-0.3) | 13.8 (10.3-18.3) | 0.4 (0.3-0.6) | 0.23 (-0.5-0.96) |
| Chronic lymphoid leukemia | Japan | 450.8 (416.1-480.2) | 0.3 (0.2-0.3) | 1056.5 (917.9-1150.9) | 0.3 (0.3-0.3) | -0.42 (-1.32-0.48) |
| Chronic lymphoid leukemia | Kingdom of Bahrain | 0.1 (0-0.2) | 0.1 (0-0.2) | 0.6 (0.1-1.1) | 0.1 (0-0.2) | -0.93 (-1.92-0.06) |
| Chronic lymphoid leukemia | Kingdom of Belgium | 462.3 (396.9-532.5) | 2.9 (2.5-3.4) | 656.2 (536.6-781.8) | 2.6 (2.2-3.1) | -0.5 (-1.41-0.41) |
| Chronic lymphoid leukemia | Kingdom of Bhutan | 0.1 (0-0.2) | 0 (0-0.1) | 0.4 (0.1-0.8) | 0.1 (0-0.1) | -0.28 (-0.94-0.38) |
| Chronic lymphoid leukemia | Kingdom of Cambodia | 12.1 (5.8-21.4) | 0.3 (0.1-0.5) | 37.1 (17.8-63.4) | 0.3 (0.2-0.5) | -0.4 (-1.26-0.47) |
| Chronic lymphoid leukemia | Kingdom of Denmark | 341.9 (306.9-381.7) | 4.2 (3.7-4.7) | 564.6 (473.6-655) | 4.5 (3.8-5.3) | -0.1 (-1.08-0.9) |
| Chronic lymphoid leukemia | Kingdom of Eswatini | 2.7 (1.7-3.7) | 1.1 (0.7-1.4) | 7.1 (3.7-11) | 1.4 (0.8-2.1) | 0.38 (-0.47-1.24) |
| Chronic lymphoid leukemia | Kingdom of Lesotho | 6.8 (3.7-9.5) | 0.9 (0.5-1.2) | 16.1 (7.7-23.9) | 1.6 (0.8-2.3) | 0.96 (0.16-1.75) |
| Chronic lymphoid leukemia | Kingdom of Morocco | 27.8 (5-46.7) | 0.2 (0-0.4) | 84.1 (19.8-138.9) | 0.3 (0.1-0.4) | -0.02 (-0.4-0.35) |
| Chronic lymphoid leukemia | Kingdom of Norway | 151.5 (139.3-162.6) | 2.2 (2-2.3) | 317.6 (272.3-361.5) | 3 (2.5-3.4) | 0.14 (-0.8-1.08) |
| Chronic lymphoid leukemia | Kingdom of Saudi Arabia | 24.2 (12.3-58.8) | 0.5 (0.2-1.1) | 255.7 (150.3-529.4) | 1.3 (0.8-2.5) | 0.78 (0.15-1.41) |
| Chronic lymphoid leukemia | Kingdom of Spain | 1515.3 (1356.8-1658.8) | 2.7 (2.5-3) | 2880.4 (2382.6-3374.8) | 2.8 (2.3-3.2) | -0.38 (-1.32-0.58) |
| Chronic lymphoid leukemia | Kingdom of Sweden | 564.6 (506-615.9) | 3.6 (3.2-3.9) | 692.2 (573.4-813.2) | 2.9 (2.4-3.4) | -0.5 (-1.52-0.54) |
| Chronic lymphoid leukemia | Kingdom of Thailand | 158.7 (52.1-247.3) | 0.5 (0.2-0.7) | 461.2 (246.2-720.9) | 0.4 (0.2-0.7) | -0.2 (-1.05-0.66) |
| Chronic lymphoid leukemia | Kingdom of the Netherlands | 683 (608.6-747.3) | 3.3 (3-3.6) | 811.9 (667.5-956.2) | 2.2 (1.8-2.6) | -0.46 (-1.44-0.52) |
| Chronic lymphoid leukemia | Kingdom of Tonga | 0.1 (0-0.1) | 0.1 (0-0.3) | 0 (0-0) | 0 (0-0) | -0.1 (-0.83-0.65) |
| Chronic lymphoid leukemia | Kyrgyz Republic | 15.9 (13.3-18.5) | 0.5 (0.4-0.6) | 20.6 (16.1-26) | 0.4 (0.3-0.5) | -0.61 (-1.2--0.02) |
| Chronic lymphoid leukemia | Lao People's Democratic Republic | 5 (2.6-9.1) | 0.2 (0.1-0.4) | 10 (5.2-17.6) | 0.2 (0.1-0.4) | -0.71 (-1.56-0.15) |
| Chronic lymphoid leukemia | Lebanese Republic | 25.1 (13.4-47.1) | 1.3 (0.7-2.3) | 127.8 (67.6-181.4) | 2.1 (1.1-3) | 0.23 (-0.62-1.08) |
| Chronic lymphoid leukemia | Malaysia | 19.1 (11.1-30.2) | 0.2 (0.1-0.3) | 82 (54.7-147.6) | 0.3 (0.2-0.5) | -0.14 (-0.97-0.7) |
| Chronic lymphoid leukemia | Mongolia | 2.2 (0.8-3.7) | 0.2 (0.1-0.3) | 5.2 (2.8-8.7) | 0.2 (0.1-0.3) | -0.33 (-1.01-0.37) |
| Chronic lymphoid leukemia | Montenegro | 12 (7.3-18.1) | 1.9 (1.2-2.9) | 32.4 (19.6-51.9) | 3.3 (2-5.2) | 0.28 (-0.62-1.2) |
| Chronic lymphoid leukemia | New Zealand | 153.9 (136.2-171.6) | 3.8 (3.4-4.3) | 334.4 (283.2-387.1) | 3.8 (3.2-4.4) | -0.17 (-1.15-0.82) |
| Chronic lymphoid leukemia | North Macedonia | 31.4 (20.3-45.7) | 1.8 (1.1-2.5) | 99.3 (61-146.5) | 3 (1.9-4.4) | 0.23 (-0.91-1.38) |
| Chronic lymphoid leukemia | Northern Mariana Islands | 0 (0-0) | 0 (0-0) | 0 (0-0) | 0 (0-0) | -0.43 (-1.2-0.34) |
| Chronic lymphoid leukemia | Palestine | 11.3 (7-19.4) | 1.4 (0.9-2.3) | 39.8 (25.4-61.2) | 1.6 (1-2.5) | -0.45 (-1.39-0.5) |
| Chronic lymphoid leukemia | People's Democratic Republic of Algeria | 14.6 (4.9-21.5) | 0.2 (0.1-0.2) | 68 (22.4-113.6) | 0.2 (0.1-0.4) | -0.14 (-0.8-0.53) |
| Chronic lymphoid leukemia | People's Republic of Bangladesh | 31.7 (11.4-57.3) | 0.1 (0-0.1) | 103.3 (34-196.7) | 0.1 (0-0.1) | -0.5 (-1.16-0.15) |
| Chronic lymphoid leukemia | People's Republic of China | 6780.3 (3988.3-9157.3) | 0.7 (0.4-1) | 28926.7 (17957.7-40579.4) | 1.4 (0.9-2) | 0.02 (-0.91-0.96) |
| Chronic lymphoid leukemia | Plurinational State of Bolivia | 11.6 (6.8-19.6) | 0.4 (0.2-0.6) | 41.2 (24-62.9) | 0.5 (0.3-0.7) | -0.32 (-1.21-0.57) |
| Chronic lymphoid leukemia | Portuguese Republic | 280.1 (251.4-309.8) | 2 (1.8-2.2) | 638.5 (525.6-739.5) | 2.4 (2-2.8) | 0 (-0.92-0.93) |
| Chronic lymphoid leukemia | Principality of Andorra | 2.6 (1.5-4.2) | 4.5 (2.6-7.4) | 6.5 (3.7-11.6) | 4.2 (2.3-7.5) | -0.58 (-1.69-0.54) |
| Chronic lymphoid leukemia | Principality of Monaco | 3.9 (2.4-6.1) | 5.4 (3.4-8.7) | 7 (3.6-10.5) | 6.9 (3.6-10.6) | 0.43 (-0.8-1.67) |
| Chronic lymphoid leukemia | Puerto Rico | 55.2 (49.2-62.2) | 1.5 (1.4-1.7) | 93.9 (73.9-114.2) | 1.3 (1-1.6) | -0.23 (-1.03-0.58) |
| Chronic lymphoid leukemia | Republic of Albania | 15.2 (8.1-27.6) | 0.8 (0.4-1.4) | 52.4 (24.2-121.8) | 1.2 (0.5-2.7) | 0.18 (-0.57-0.94) |
| Chronic lymphoid leukemia | Republic of Angola | 19.5 (8.1-28.3) | 0.6 (0.2-0.8) | 76 (27.7-121.3) | 0.7 (0.3-1.2) | -0.04 (-0.59-0.51) |
| Chronic lymphoid leukemia | Republic of Armenia | 41.9 (37.2-46.4) | 1.4 (1.3-1.6) | 53.3 (46-63.4) | 1.2 (1.1-1.5) | -0.36 (-1.1-0.38) |
| Chronic lymphoid leukemia | Republic of Austria | 424.2 (385.3-461.3) | 3.5 (3.2-3.8) | 715.5 (598.5-822.9) | 3.7 (3.1-4.3) | -0.08 (-1.07-0.91) |
| Chronic lymphoid leukemia | Republic of Azerbaijan | 21.2 (10.8-37.2) | 0.4 (0.2-0.7) | 47.2 (19.7-100.3) | 0.4 (0.2-0.9) | -0.47 (-1.22-0.3) |
| Chronic lymphoid leukemia | Republic of Belarus | 294.1 (237.7-360.3) | 2.2 (1.8-2.7) | 637.8 (492.6-819.8) | 4 (3.1-5.1) | -0.11 (-0.97-0.76) |
| Chronic lymphoid leukemia | Republic of Benin | 4.4 (2.6-5.9) | 0.2 (0.1-0.3) | 17.2 (5.7-24.5) | 0.4 (0.1-0.5) | 0.23 (-0.15-0.62) |
| Chronic lymphoid leukemia | Republic of Botswana | 4.8 (2.5-7) | 1 (0.5-1.4) | 14.5 (6.8-21.9) | 1.1 (0.5-1.6) | 0 (-0.74-0.73) |
| Chronic lymphoid leukemia | Republic of Bulgaria | 93.5 (73.7-118.4) | 0.8 (0.6-1) | 182.9 (126.2-260.9) | 1.3 (0.9-1.8) | 0.23 (-0.51-0.98) |
| Chronic lymphoid leukemia | Republic of Burundi | 15.2 (8.5-22.6) | 0.7 (0.4-1) | 34.3 (15.2-52.7) | 0.8 (0.4-1.1) | -0.1 (-0.65-0.45) |
| Chronic lymphoid leukemia | Republic of Cabo Verde | 1.3 (0.8-2) | 0.5 (0.3-0.8) | 5.2 (2.8-7.7) | 1.2 (0.7-1.8) | 0.35 (-0.28-0.99) |
| Chronic lymphoid leukemia | Republic of Cameroon | 11.6 (6.7-16.2) | 0.3 (0.2-0.4) | 48.9 (16.4-80) | 0.4 (0.2-0.7) | 0.27 (-0.14-0.68) |
| Chronic lymphoid leukemia | Republic of Chad | 5.4 (3-7.5) | 0.2 (0.1-0.3) | 18.1 (5.9-27.5) | 0.4 (0.1-0.5) | 0.4 (0.03-0.78) |
| Chronic lymphoid leukemia | Republic of Chile | 63.5 (55.6-72.5) | 0.7 (0.6-0.8) | 193.7 (162.4-227.1) | 0.7 (0.6-0.9) | 0.06 (-0.67-0.79) |
| Chronic lymphoid leukemia | Republic of Colombia | 98.1 (86.6-109.8) | 0.6 (0.5-0.6) | 306.1 (243.9-377.2) | 0.6 (0.4-0.7) | -0.05 (-0.85-0.76) |
| Chronic lymphoid leukemia | Republic of Costa Rica | 14.9 (12.5-17.2) | 0.9 (0.7-1) | 64.1 (53.7-76.1) | 1.2 (1-1.4) | 0.26 (-0.56-1.08) |
| Chronic lymphoid leukemia | République de Côte d’Ivoire | 6.8 (3.3-9) | 0.2 (0.1-0.3) | 24.3 (10.4-37) | 0.2 (0.1-0.4) | 0.02 (-0.28-0.32) |
| Chronic lymphoid leukemia | Republic of Croatia | 156.9 (134.6-181.2) | 2.6 (2.3-3) | 429.2 (359.8-508.7) | 4.7 (3.9-5.5) | 0.2 (-0.77-1.17) |
| Chronic lymphoid leukemia | Republic of Cuba | 129.1 (115.7-140.1) | 1.3 (1.1-1.4) | 239.7 (203.8-278.4) | 1.2 (1-1.4) | -0.16 (-0.92-0.61) |
| Chronic lymphoid leukemia | Republic of Cyprus | 24.9 (16.6-40.7) | 3.6 (2.4-6) | 98.8 (60.9-133.8) | 4.8 (3-6.4) | 0.65 (-0.53-1.84) |
| Chronic lymphoid leukemia | Republic of Djibouti | 0.8 (0.4-1.2) | 0.7 (0.3-0.9) | 5.9 (2.5-9.5) | 1 (0.5-1.5) | 0.27 (-0.3-0.83) |
| Chronic lymphoid leukemia | Republic of Ecuador | 16.4 (13.8-19.4) | 0.3 (0.3-0.4) | 70.4 (53.4-91.3) | 0.4 (0.3-0.6) | 0.5 (-0.36-1.37) |
| Chronic lymphoid leukemia | Republic of El Salvador | 8.7 (5.4-12.6) | 0.3 (0.2-0.4) | 33.2 (19.6-46.9) | 0.5 (0.3-0.8) | 0.24 (-0.59-1.07) |
| Chronic lymphoid leukemia | Republic of Equatorial Guinea | 1.1 (0.5-1.5) | 0.6 (0.3-0.8) | 4.1 (1.6-6.9) | 0.8 (0.4-1.3) | -0.06 (-0.6-0.49) |
| Chronic lymphoid leukemia | Republic of Estonia | 98.8 (85.8-112.5) | 4.8 (4.2-5.5) | 130.1 (103.2-156.7) | 4.9 (3.8-5.9) | -0.4 (-1.4-0.61) |
| Chronic lymphoid leukemia | Republic of Fiji | 0.1 (0-0.2) | 0 (0-0.1) | 0.1 (0-0.3) | 0 (0-0) | -0.31 (-1.42-0.81) |
| Chronic lymphoid leukemia | Republic of Finland | 179.1 (159.8-198.2) | 2.5 (2.2-2.7) | 326.1 (268.5-384.4) | 2.5 (2.1-2.9) | 0.09 (-0.78-0.98) |
| Chronic lymphoid leukemia | Republic of Ghana | 27.8 (10.9-38.7) | 0.5 (0.2-0.7) | 60.9 (40.2-92.2) | 0.4 (0.3-0.6) | -0.72 (-1.21--0.23) |
| Chronic lymphoid leukemia | Republic of Guatemala | 5.7 (5-6.4) | 0.2 (0.2-0.2) | 24.1 (20.4-28.3) | 0.2 (0.2-0.3) | 0.11 (-0.67-0.89) |
| Chronic lymphoid leukemia | Republic of Guinea | 2.7 (1.4-3.8) | 0.1 (0-0.1) | 5.5 (2.5-8) | 0.1 (0-0.2) | -0.02 (-0.2-0.17) |
| Chronic lymphoid leukemia | Republic of Guinea-Bissau | 1 (0.5-1.5) | 0.3 (0.2-0.4) | 2.9 (1-4.4) | 0.5 (0.2-0.7) | 0.26 (-0.18-0.7) |
| Chronic lymphoid leukemia | Republic of Guyana | 2.2 (1.9-2.6) | 0.6 (0.5-0.7) | 3.8 (2.9-5) | 0.6 (0.5-0.8) | 0.19 (-0.46-0.84) |
| Chronic lymphoid leukemia | Republic of Haiti | 17.3 (9.7-31.1) | 0.6 (0.3-1) | 36.9 (21.1-62.5) | 0.6 (0.3-1) | -0.31 (-1.05-0.44) |
| Chronic lymphoid leukemia | Republic of Honduras | 6.8 (3.9-9.8) | 0.3 (0.2-0.5) | 33.3 (19.4-54.8) | 0.6 (0.3-0.9) | -0.11 (-0.91-0.69) |
| Chronic lymphoid leukemia | Republic of Iceland | 8 (7-9.1) | 2.7 (2.4-3.1) | 16.2 (13.5-19.4) | 2.7 (2.2-3.2) | -0.09 (-1.04-0.86) |
| Chronic lymphoid leukemia | Republic of India | 338.3 (121.3-473.5) | 0.1 (0-0.1) | 1298.4 (600.9-1781.1) | 0.1 (0.1-0.2) | -0.26 (-0.86-0.35) |
| Chronic lymphoid leukemia | Republic of Indonesia | 182 (99.6-263.3) | 0.2 (0.1-0.3) | 624.1 (378.9-1097.1) | 0.3 (0.2-0.5) | -0.1 (-0.93-0.74) |
| Chronic lymphoid leukemia | Republic of Iraq | 55.6 (15.3-95.9) | 0.7 (0.2-1.2) | 242.2 (76.7-394.4) | 1 (0.3-1.6) | 0.05 (-0.81-0.92) |
| Chronic lymphoid leukemia | Republic of Italy | 3437.7 (3180.7-3662.9) | 3.8 (3.5-4) | 5502.9 (4728.4-6153.4) | 3.5 (3.1-3.9) | -0.71 (-1.74-0.32) |
| Chronic lymphoid leukemia | Republic of Kazakhstan | 64 (38.5-99.5) | 0.5 (0.3-0.8) | 103.2 (59.8-160.1) | 0.6 (0.3-0.9) | -0.33 (-1-0.35) |
| Chronic lymphoid leukemia | Republic of Kenya | 29.7 (19.1-47.5) | 0.4 (0.3-0.6) | 122.5 (81.7-175.6) | 0.6 (0.4-0.8) | 0.32 (-0.15-0.79) |
| Chronic lymphoid leukemia | Republic of Kiribati | 0 (0-0) | 0 (0-0.1) | 0 (0-0) | 0 (0-0) | -0.04 (-0.75-0.68) |
| Chronic lymphoid leukemia | Republic of Korea | 58.1 (38-106.4) | 0.2 (0.1-0.4) | 264 (160.2-466.6) | 0.3 (0.2-0.5) | -0.01 (-0.86-0.85) |
| Chronic lymphoid leukemia | Republic of Latvia | 121.4 (96.5-144.8) | 3.4 (2.7-4) | 170.6 (139.3-209) | 4.4 (3.6-5.4) | -0.47 (-1.42-0.49) |
| Chronic lymphoid leukemia | Republic of Liberia | 2.4 (1.3-3.4) | 0.2 (0.1-0.3) | 7.7 (2.6-11.9) | 0.4 (0.1-0.6) | 0.28 (-0.09-0.66) |
| Chronic lymphoid leukemia | Republic of Lithuania | 191.6 (162.3-217) | 4.2 (3.6-4.8) | 195.8 (159.1-234.3) | 3.5 (2.8-4.1) | -0.45 (-1.37-0.48) |
| Chronic lymphoid leukemia | Republic of Madagascar | 26.5 (14-35.1) | 0.6 (0.3-0.7) | 67.4 (31.5-98.6) | 0.7 (0.3-0.9) | 0.05 (-0.44-0.54) |
| Chronic lymphoid leukemia | Republic of Malawi | 7.2 (3.8-9.7) | 0.2 (0.1-0.3) | 18.2 (9.4-25.7) | 0.3 (0.1-0.4) | -0.06 (-0.36-0.23) |
| Chronic lymphoid leukemia | Republic of Maldives | 0.1 (0.1-0.3) | 0.2 (0.1-0.4) | 0.6 (0.2-1.1) | 0.1 (0.1-0.3) | -0.85 (-1.59--0.1) |
| Chronic lymphoid leukemia | Republic of Mali | 7.8 (3.9-10.7) | 0.2 (0.1-0.3) | 19.6 (8.9-27.9) | 0.2 (0.1-0.3) | -0.09 (-0.41-0.23) |
| Chronic lymphoid leukemia | Republic of Malta | 6.9 (5.9-8) | 1.6 (1.4-1.9) | 15.2 (11.9-18.7) | 1.5 (1.2-1.8) | -0.05 (-0.94-0.85) |
| Chronic lymphoid leukemia | Republic of Mauritius | 11.9 (10.6-13.4) | 1.6 (1.4-1.8) | 2.9 (2.5-3.4) | 0.2 (0.1-0.2) | -0.66 (-1.44-0.13) |
| Chronic lymphoid leukemia | Republic of Moldova | 32.1 (26.7-36.6) | 0.7 (0.6-0.8) | 47.9 (41.3-55.3) | 0.8 (0.7-0.9) | -0.54 (-1.25-0.18) |
| Chronic lymphoid leukemia | Republic of Mozambique | 49.9 (28-67.7) | 1 (0.6-1.3) | 119.2 (70.9-168.5) | 1.2 (0.8-1.7) | 0.18 (-0.48-0.84) |
| Chronic lymphoid leukemia | Republic of Namibia | 4 (2.6-5.3) | 0.7 (0.5-0.9) | 10.4 (6.6-15) | 0.8 (0.6-1.2) | 0.04 (-0.59-0.67) |
| Chronic lymphoid leukemia | Republic of Nauru | 0 (0-0) | 0.1 (0-0.4) | 0 (0-0) | 0 (0-0) | -0.17 (-1.1-0.77) |
| Chronic lymphoid leukemia | Republic of Nicaragua | 5.8 (3.9-8.3) | 0.4 (0.3-0.5) | 26.9 (16.8-36.8) | 0.6 (0.4-0.8) | -0.02 (-0.79-0.74) |
| Chronic lymphoid leukemia | Republic of Niue | 0 (0-0) | 0.1 (0-0.2) | 0 (0-0) | 0 (0-0) | 0.05 (-0.76-0.86) |
| Chronic lymphoid leukemia | Republic of Palau | 0 (0-0.1) | 0.3 (0.1-0.5) | 0.1 (0-0.1) | 0.4 (0.1-0.5) | -0.06 (-0.58-0.46) |
| Chronic lymphoid leukemia | Republic of Panama | 5.4 (4.9-6) | 0.4 (0.3-0.4) | 23.8 (18.4-29.1) | 0.5 (0.4-0.7) | 0.32 (-0.48-1.12) |
| Chronic lymphoid leukemia | Republic of Paraguay | 13.7 (9.1-19.2) | 0.7 (0.4-0.9) | 63 (41.1-94.6) | 1.1 (0.7-1.7) | 0.42 (-0.32-1.16) |
| Chronic lymphoid leukemia | Republic of Peru | 28.2 (18.1-43.2) | 0.2 (0.2-0.4) | 150.6 (75.1-217.5) | 0.4 (0.2-0.6) | 0.24 (-0.58-1.06) |
| Chronic lymphoid leukemia | Republic of Poland | 918.6 (794.6-1012.7) | 2.1 (1.8-2.3) | 2682.5 (2389.1-2983.4) | 3.6 (3.3-4) | -0.2 (-1.1-0.71) |
| Chronic lymphoid leukemia | Republic of Rwanda | 21.4 (13.4-29.4) | 0.8 (0.5-1.1) | 55.5 (26.1-82.8) | 1 (0.5-1.4) | -0.42 (-1-0.16) |
| Chronic lymphoid leukemia | Republic of San Marino | 1.7 (1.1-2.5) | 4.6 (3-6.9) | 2.4 (1.2-3.8) | 3.1 (1.6-5.2) | -0.71 (-1.9-0.49) |
| Chronic lymphoid leukemia | Republic of Senegal | 7 (3.9-9.5) | 0.2 (0.1-0.3) | 30 (10.9-43.3) | 0.4 (0.2-0.6) | 0.26 (-0.13-0.65) |
| Chronic lymphoid leukemia | Republic of Serbia | 149.8 (108.1-218.1) | 1.4 (1-2.1) | 477 (328.8-684.1) | 2.8 (2-4.1) | 0.05 (-0.8-0.91) |
| Chronic lymphoid leukemia | Republic of Seychelles | 0.6 (0.4-0.9) | 1.1 (0.7-1.6) | 1.3 (0.8-1.9) | 1.2 (0.7-1.6) | -0.24 (-0.96-0.5) |
| Chronic lymphoid leukemia | Republic of Sierra Leone | 4.1 (2.1-5.5) | 0.2 (0.1-0.3) | 12.6 (4.1-17.9) | 0.4 (0.1-0.5) | 0.29 (-0.08-0.67) |
| Chronic lymphoid leukemia | Republic of Singapore | 7.4 (6.6-8.2) | 0.4 (0.3-0.4) | 35.7 (31.6-39.9) | 0.4 (0.4-0.5) | -0.07 (-0.99-0.85) |
| Chronic lymphoid leukemia | Republic of Slovenia | 81.8 (69.7-94.4) | 3.3 (2.8-3.8) | 296.1 (235.6-371) | 6.5 (5.2-8.2) | 0.57 (-0.5-1.65) |
| Chronic lymphoid leukemia | Republic of South Africa | 212.9 (85.9-302.2) | 1.1 (0.4-1.5) | 648.7 (294.4-862.3) | 1.5 (0.7-2) | 0.18 (-0.61-0.97) |
| Chronic lymphoid leukemia | Republic of South Sudan | 16.1 (9-24.2) | 0.7 (0.4-1) | 33.2 (17.8-49.4) | 0.9 (0.5-1.4) | 0.36 (-0.2-0.92) |
| Chronic lymphoid leukemia | Republic of Sudan | 72.4 (12.2-130.5) | 0.9 (0.1-1.6) | 193 (36.9-343.4) | 1 (0.2-1.9) | -0.17 (-1-0.66) |
| Chronic lymphoid leukemia | Republic of Suriname | 1 (0.6-1.4) | 0.4 (0.2-0.6) | 2.9 (1.6-4.3) | 0.5 (0.3-0.7) | -0.07 (-0.69-0.55) |
| Chronic lymphoid leukemia | Republic of Tajikistan | 9 (3.9-15.1) | 0.3 (0.1-0.5) | 13.3 (6.6-24.7) | 0.2 (0.1-0.3) | -0.74 (-1.39--0.08) |
| Chronic lymphoid leukemia | Republic of the Congo | 6.8 (3.3-9.6) | 0.7 (0.3-0.9) | 20.8 (8.6-29.8) | 0.8 (0.4-1.1) | -0.09 (-0.67-0.5) |
| Chronic lymphoid leukemia | Republic of the Gambia | 0.4 (0.2-0.6) | 0.1 (0.1-0.2) | 1.7 (0.8-2.5) | 0.2 (0.1-0.3) | 0 (-0.22-0.23) |
| Chronic lymphoid leukemia | Republic of the Marshall Islands | 0 (0-0) | 0.1 (0-0.2) | 0 (0-0) | 0 (0-0) | -0.02 (-0.85-0.81) |
| Chronic lymphoid leukemia | Republic of the Niger | 4.8 (2.6-6.8) | 0.2 (0.1-0.3) | 24.2 (7.8-37.7) | 0.3 (0.1-0.5) | 0.14 (-0.23-0.51) |
| Chronic lymphoid leukemia | Republic of the Philippines | 84.5 (31.4-115.4) | 0.3 (0.1-0.4) | 211.3 (114.3-320.2) | 0.3 (0.1-0.4) | -0.29 (-1.12-0.54) |
| Chronic lymphoid leukemia | Republic of the Union of Myanmar | 57.5 (31.2-98.7) | 0.3 (0.1-0.4) | 95.4 (60.6-177) | 0.2 (0.1-0.4) | -0.88 (-1.75-0.01) |
| Chronic lymphoid leukemia | Republic of Trinidad and Tobago | 4.3 (3.9-4.7) | 0.5 (0.5-0.6) | 9.7 (7.4-12.3) | 0.5 (0.4-0.6) | -0.25 (-0.94-0.46) |
| Chronic lymphoid leukemia | Republic of Tunisia | 32.8 (6-56.5) | 0.7 (0.1-1.2) | 119 (22.9-207.2) | 0.9 (0.2-1.6) | -0.16 (-0.83-0.51) |
| Chronic lymphoid leukemia | Republic of Turkey | 471 (253.4-658.9) | 1.5 (0.8-2) | 1709.7 (1026.6-2521.5) | 1.9 (1.1-2.7) | -0.44 (-1.35-0.48) |
| Chronic lymphoid leukemia | Republic of Uganda | 25.8 (14.7-35.3) | 0.4 (0.3-0.6) | 69.4 (40-97.4) | 0.5 (0.3-0.7) | 0.02 (-0.45-0.5) |
| Chronic lymphoid leukemia | Republic of Uzbekistan | 48.3 (34.6-65.6) | 0.4 (0.3-0.5) | 92.7 (62.4-130.7) | 0.3 (0.2-0.4) | -0.6 (-1.27-0.07) |
| Chronic lymphoid leukemia | Republic of Vanuatu | 0 (0-0.1) | 0.1 (0-0.2) | 0 (0-0) | 0 (0-0) | -0.11 (-0.88-0.66) |
| Chronic lymphoid leukemia | Republic of Yemen | 43.3 (5.7-86) | 1 (0.1-1.9) | 143.5 (21.1-267.6) | 1.1 (0.2-2) | -0.19 (-0.96-0.59) |
| Chronic lymphoid leukemia | Republic of Zambia | 18 (11.7-23.6) | 0.7 (0.5-0.9) | 68.3 (25.7-108.4) | 1 (0.4-1.5) | 0.02 (-0.56-0.61) |
| Chronic lymphoid leukemia | Republic of Zimbabwe | 38 (21.1-53.4) | 1.1 (0.6-1.4) | 91.8 (50.1-128.4) | 1.4 (0.9-1.9) | 0.62 (-0.2-1.46) |
| Chronic lymphoid leukemia | Romania | 206.1 (183.6-226.8) | 0.7 (0.7-0.8) | 664.1 (562.5-768.8) | 1.8 (1.5-2.1) | 0.35 (-0.39-1.1) |
| Chronic lymphoid leukemia | Russian Federation | 2548.7 (2366.4-2788.6) | 1.4 (1.3-1.5) | 5503.5 (4995.7-6037.3) | 2.3 (2.1-2.5) | -0.07 (-0.79-0.65) |
| Chronic lymphoid leukemia | Saint Kitts and Nevis | 0.3 (0.2-0.3) | 0.7 (0.6-0.8) | 0.6 (0.4-0.7) | 0.8 (0.6-1) | -0.21 (-0.92-0.51) |
| Chronic lymphoid leukemia | Saint Lucia | 0.8 (0.7-0.9) | 1 (0.9-1) | 2.6 (2.1-3.2) | 1.1 (0.9-1.3) | -0.51 (-1.14-0.13) |
| Chronic lymphoid leukemia | Saint Vincent and the Grenadines | 0.9 (0.8-1) | 1.2 (1.1-1.4) | 1.5 (1.3-1.7) | 1.1 (0.9-1.2) | -0.2 (-0.88-0.48) |
| Chronic lymphoid leukemia | Slovak Republic | 132.3 (73.1-193.6) | 2.2 (1.2-3.2) | 349.9 (210.1-484.5) | 3.6 (2.2-5) | 0.05 (-0.91-1.01) |
| Chronic lymphoid leukemia | Socialist Republic of Viet Nam | 11.5 (4.1-17.4) | 0 (0-0) | 33.8 (17.1-51.3) | 0 (0-0.1) | -0.2 (-0.9-0.51) |
| Chronic lymphoid leukemia | Solomon Islands | 0.1 (0-0.3) | 0.1 (0-0.2) | 0 (0-0.1) | 0 (0-0) | -0.04 (-0.85-0.77) |
| Chronic lymphoid leukemia | State of Eritrea | 6.8 (4.1-10) | 0.7 (0.4-0.9) | 23 (11.6-33.7) | 0.9 (0.5-1.3) | 0.21 (-0.35-0.78) |
| Chronic lymphoid leukemia | State of Israel | 200.6 (176.5-228) | 4.2 (3.7-4.7) | 549.1 (453.7-633) | 4.3 (3.6-4.9) | -0.15 (-1.27-0.99) |
| Chronic lymphoid leukemia | State of Kuwait | 3.4 (2.7-4.1) | 0.6 (0.5-0.7) | 21.6 (16.5-27.7) | 0.8 (0.6-1) | -0.59 (-1.35-0.18) |
| Chronic lymphoid leukemia | State of Libya | 23.2 (4.7-39.5) | 1.3 (0.3-2.2) | 81.3 (21.5-131.3) | 1.7 (0.4-2.7) | 0.47 (-0.43-1.38) |
| Chronic lymphoid leukemia | State of Qatar | 1.6 (0.6-2.5) | 1.8 (0.7-2.7) | 19.2 (6.1-32.8) | 2 (0.9-3.2) | -0.67 (-1.49-0.15) |
| Chronic lymphoid leukemia | Sultanate of Oman | 5.5 (2-9.8) | 0.8 (0.3-1.4) | 23 (10.1-39.1) | 1.2 (0.6-1.9) | -0.14 (-0.92-0.65) |
| Chronic lymphoid leukemia | Swiss Confederation | 396.5 (338.6-452.2) | 3.7 (3.2-4.3) | 610.5 (496-752.8) | 3.2 (2.6-3.9) | -0.65 (-1.67-0.39) |
| Chronic lymphoid leukemia | Syrian Arab Republic | 77.8 (13.7-135) | 1.6 (0.3-2.8) | 255 (62.2-428.3) | 2.1 (0.5-3.4) | -0.4 (-1.27-0.48) |
| Chronic lymphoid leukemia | Taiwan (Province of China) | 38.3 (30-49.8) | 0.2 (0.2-0.3) | 242 (205.3-288.2) | 0.6 (0.5-0.7) | 0.82 (0-1.65) |
| Chronic lymphoid leukemia | Togolese Republic | 2.7 (1.4-3.6) | 0.2 (0.1-0.3) | 15.1 (4.8-22.7) | 0.4 (0.1-0.7) | 0.32 (-0.06-0.7) |
| Chronic lymphoid leukemia | Tokelau | 0 (0-0) | 0.1 (0-0.2) | 0 (0-0) | 0 (0-0) | -0.05 (-0.85-0.76) |
| Chronic lymphoid leukemia | Turkmenistan | 6.1 (4.1-8.5) | 0.3 (0.2-0.4) | 13 (7.5-20.2) | 0.3 (0.2-0.4) | -0.31 (-0.95-0.33) |
| Chronic lymphoid leukemia | Tuvalu | 0 (0-0) | 0.1 (0-0.2) | 0 (0-0) | 0 (0-0) | -0.3 (-1.12-0.52) |
| Chronic lymphoid leukemia | Ukraine | 1185 (901.2-1480.2) | 1.6 (1.2-2) | 1348.9 (979-1815.3) | 1.7 (1.3-2.4) | -0.96 (-1.7--0.22) |
| Chronic lymphoid leukemia | Union of the Comoros | 1.3 (0.7-1.8) | 0.7 (0.4-1) | 4.3 (2.1-6.2) | 0.9 (0.5-1.3) | 0.07 (-0.48-0.63) |
| Chronic lymphoid leukemia | United Arab Emirates | 6.4 (3.7-10.7) | 1.7 (0.9-2.7) | 59.2 (32.7-95.8) | 1.9 (1.2-3) | 0.09 (-0.8-0.98) |
| Chronic lymphoid leukemia | United Kingdom of Great Britain and Northern Ireland | 3474.1 (3250.7-3615.7) | 3.7 (3.5-3.9) | 3945.1 (3579.6-4167.8) | 2.8 (2.6-3) | -0.54 (-1.58-0.5) |
| Chronic lymphoid leukemia | United Mexican States | 158.2 (152-163.7) | 0.4 (0.4-0.4) | 574.6 (506.4-643) | 0.5 (0.4-0.5) | -0.05 (-0.9-0.82) |
| Chronic lymphoid leukemia | United Republic of Tanzania | 72.3 (39.2-101) | 0.7 (0.4-1) | 209.9 (94.3-310.4) | 0.9 (0.4-1.3) | 0.06 (-0.5-0.62) |
| Chronic lymphoid leukemia | United States of America | 15901.4 (14864.6-16481.9) | 4.9 (4.6-5) | 18898 (17021.4-20040.1) | 3 (2.8-3.2) | -0.85 (-1.95-0.26) |
| Chronic lymphoid leukemia | United States Virgin Islands | 1.7 (1.1-2.1) | 2 (1.3-2.6) | 2.8 (1.7-4) | 1.7 (1-2.3) | -0.35 (-1.16-0.46) |
| Chronic lymphoid leukemia | African Union | 1750.1 (930.96-2245.37) | 0.67 (0.36-0.87) | 5099.28 (2398.25-6843.52) | 0.85 (0.41-1.12) | 0.82 (0.79-0.85) |
| Chronic lymphoid leukemia | Association of Southeast Asian Nations | 538.26 (275.95-710.85) | 0.23 (0.11-0.3) | 1592.3 (1008.24-2333.57) | 0.26 (0.16-0.38) | 0.25 (0.14-0.37) |
| Chronic lymphoid leukemia | Central Europe, Eastern Europe, and Central Asia | 7324.29 (6726.14-7918.21) | 1.52 (1.39-1.64) | 15513.03 (14440.1-16596.12) | 2.36 (2.2-2.53) | 1.35 (1.22-1.47) |
| Chronic lymphoid leukemia | Commonwealth | 7044.51 (6565.89-7377.94) | 0.96 (0.89-1.01) | 12012.55 (10184.55-13102.29) | 0.67 (0.57-0.73) | -1.27 (-1.55--0.98) |
| Chronic lymphoid leukemia | European Union | 17960.83 (16876.77-18910.83) | 2.95 (2.77-3.1) | 32401.84 (28838.12-35163.87) | 3.21 (2.88-3.47) | 0.25 (-0.1-0.6) |
| Chronic lymphoid leukemia | Four World Regions | 57835.32 (52980.19-61552.81) | 1.55 (1.41-1.64) | 117732.19 (98101.04-132433.71) | 1.39 (1.16-1.56) | -0.47 (-0.67--0.28) |
| Chronic lymphoid leukemia | G20 | 51612.68 (47787.03-54636.55) | 1.78 (1.64-1.88) | 102449.43 (87791.46-116122.3) | 1.58 (1.35-1.79) | -0.52 (-0.73--0.32) |
| Chronic lymphoid leukemia | Gulf Cooperation Council | 41.22 (25.03-81.48) | 0.57 (0.35-1.11) | 379.3 (246.34-683.47) | 1.25 (0.85-2.04) | 2.99 (2.78-3.21) |
| Chronic lymphoid leukemia | Health System Grouping Levels | 57892.91 (53033.16-61612.03) | 1.55 (1.41-1.64) | 117830.59 (98188.45-132552.14) | 1.39 (1.16-1.56) | -0.47 (-0.67--0.28) |
| Chronic lymphoid leukemia | High SDI | 34575.23 (32535.14-35901.95) | 3.08 (2.9-3.2) | 52424.27 (47118.89-55939.41) | 2.38 (2.16-2.52) | -0.98 (-1.31--0.64) |
| Chronic lymphoid leukemia | High-income | 38852.8 (36598.9-40331.74) | 3.18 (2.99-3.3) | 56771.14 (50500.32-60749.47) | 2.42 (2.18-2.57) | -1.05 (-1.41--0.69) |
| Chronic lymphoid leukemia | High-middle SDI | 15011.65 (13722.87-16362.84) | 1.53 (1.4-1.67) | 37237.28 (30038.23-43674.53) | 1.91 (1.54-2.25) | 0.64 (0.5-0.78) |
| Chronic lymphoid leukemia | Latin America and Caribbean | 1095.16 (1030.86-1157.04) | 0.52 (0.49-0.55) | 3778.96 (3433.72-4112.1) | 0.62 (0.56-0.67) | 0.52 (0.4-0.63) |
| Chronic lymphoid leukemia | Low SDI | 1132.39 (639.24-1499.73) | 0.54 (0.31-0.71) | 2590.8 (1326.01-3643.96) | 0.56 (0.29-0.79) | 0.02 (-0.08-0.11) |
| Chronic lymphoid leukemia | Low-middle SDI | 1127.28 (627.1-1421.36) | 0.2 (0.11-0.25) | 3949.48 (2143.26-4956.8) | 0.28 (0.16-0.35) | 1.2 (1.15-1.25) |
| Chronic lymphoid leukemia | Middle SDI | 6046.38 (3631.2-7497.51) | 0.55 (0.34-0.67) | 21628.75 (13983.63-28514.84) | 0.8 (0.52-1.05) | 1.31 (1.26-1.37) |
| Chronic lymphoid leukemia | Nordic Region | 1245.12 (1158.02-1326.61) | 3.24 (3.02-3.45) | 1917.03 (1674.04-2135.98) | 3.15 (2.78-3.51) | -0.03 (-0.25-0.19) |
| Chronic lymphoid leukemia | North Africa and Middle East | 1418.91 (593.83-1904.27) | 0.92 (0.4-1.24) | 5878.21 (2720.1-7511.21) | 1.37 (0.65-1.73) | 1.5 (1.37-1.64) |
| Chronic lymphoid leukemia | OECD Countries | 41400.92 (39101.17-42931.73) | 3.04 (2.87-3.15) | 64078.39 (57538.18-68224.25) | 2.39 (2.18-2.53) | -0.92 (-1.26--0.59) |
| Chronic lymphoid leukemia | Organization of Islamic Cooperation | 2125.29 (1127.39-2710.28) | 0.44 (0.24-0.56) | 7849.8 (4023.19-9708.66) | 0.66 (0.35-0.82) | 1.53 (1.44-1.62) |
| Chronic lymphoid leukemia | Sahel Region | 226.2 (101.97-317.26) | 0.38 (0.18-0.54) | 644.77 (253.81-881.35) | 0.5 (0.21-0.69) | 0.94 (0.91-0.98) |
| Chronic lymphoid leukemia | South Asia | 411.74 (154.37-575.43) | 0.08 (0.03-0.11) | 1495.66 (731.74-2050.52) | 0.11 (0.05-0.15) | 0.81 (0.7-0.91) |
| Chronic lymphoid leukemia | Southeast Asia, East Asia, and Oceania | 7444.79 (4321.37-10023.1) | 0.59 (0.35-0.78) | 30969.33 (19297.13-43797.36) | 1.08 (0.68-1.54) | 2.29 (2.19-2.39) |
| Chronic lymphoid leukemia | Sub-Saharan Africa | 1413.42 (819.85-1825.4) | 0.71 (0.43-0.92) | 3580.8 (2010.05-4720.36) | 0.83 (0.47-1.09) | 0.45 (0.42-0.47) |
| Chronic lymphoid leukemia | WHO region | 57796.91 (52939.33-61520.73) | 1.55 (1.42-1.65) | 117489.92 (97864.87-132208.74) | 1.39 (1.17-1.56) | -0.47 (-0.67--0.28) |
| Chronic lymphoid leukemia | World Bank Income Levels | 57892.9 (53033.16-61612.02) | 1.55 (1.41-1.64) | 117830.58 (98188.45-132552.14) | 1.39 (1.16-1.56) | -0.47 (-0.67--0.28) |
| Chronic lymphoid leukemia | World Bank Regions | 57861.49 (53013.21-61580.94) | 1.55 (1.41-1.64) | 117731.26 (98131.03-132403.62) | 1.39 (1.16-1.56) | -0.48 (-0.67--0.28) |
| Chronic myeloid leukemia | American Samoa | 0.1 (0-0.3) | 0.2 (0.1-1.1) | 0.5 (0.2-0.8) | 1.1 (0.5-1.6) | 0.28 (-0.53-1.1) |
| Chronic myeloid leukemia | Antigua and Barbuda | 0.9 (0.8-1) | 1.6 (1.4-1.8) | 1.1 (1-1.2) | 1 (0.9-1.1) | -0.02 (-0.8-0.76) |
| Chronic myeloid leukemia | Arab Republic of Egypt | 190.7 (104-409.7) | 0.5 (0.3-1.4) | 427 (196.7-777.2) | 0.6 (0.3-1.3) | 1.14 (0.33-1.95) |
| Chronic myeloid leukemia | Argentine Republic | 394.1 (331.3-494.2) | 1.2 (1-1.5) | 233.9 (197.2-271) | 0.4 (0.4-0.5) | -0.36 (-1.12-0.4) |
| Chronic myeloid leukemia | Australia | 311.4 (285.1-340.4) | 1.6 (1.5-1.8) | 258 (218.4-302) | 0.6 (0.5-0.7) | -0.18 (-1.19-0.84) |
| Chronic myeloid leukemia | Barbados | 1.9 (1.7-2.2) | 0.7 (0.6-0.8) | 2.9 (2.2-3.6) | 0.6 (0.5-0.8) | 0.15 (-0.64-0.95) |
| Chronic myeloid leukemia | Belize | 0.4 (0.3-0.5) | 0.4 (0.3-0.4) | 1.2 (1-1.3) | 0.3 (0.3-0.4) | 0.09 (-0.54-0.71) |
| Chronic myeloid leukemia | Bermuda | 0.6 (0.5-0.7) | 0.9 (0.7-1) | 0.6 (0.5-0.8) | 0.6 (0.4-0.7) | -0.15 (-0.92-0.62) |
| Chronic myeloid leukemia | Bolivarian Republic of Venezuela | 121 (109.3-140.2) | 1 (0.9-1.1) | 159.7 (112.9-218.3) | 0.6 (0.4-0.7) | -0.04 (-0.78-0.72) |
| Chronic myeloid leukemia | Bosnia and Herzegovina | 12 (3.5-30.4) | 0.3 (0.1-0.7) | 18.2 (5.1-39.1) | 0.3 (0.1-0.7) | 0.51 (-0.29-1.31) |
| Chronic myeloid leukemia | Brunei Darussalam | 1.2 (0.5-2.1) | 0.6 (0.2-1.1) | 1.5 (0.5-2.4) | 0.3 (0.1-0.5) | -0.24 (-1.06-0.59) |
| Chronic myeloid leukemia | Burkina Faso | 4 (1.3-11.1) | 0.1 (0-0.2) | 7.2 (3.1-19.4) | 0 (0-0.1) | 0.29 (-0.1-0.67) |
| Chronic myeloid leukemia | Canada | 595.6 (532.9-654.1) | 1.9 (1.7-2.1) | 454.5 (389.1-529.8) | 0.7 (0.6-0.9) | -0.44 (-1.56-0.68) |
| Chronic myeloid leukemia | Central African Republic | 2.2 (0.9-4.5) | 0.1 (0.1-0.3) | 3.2 (1.1-6.3) | 0.1 (0-0.2) | -0.05 (-0.64-0.54) |
| Chronic myeloid leukemia | Commonwealth of Dominica | 0.5 (0.2-0.8) | 0.8 (0.3-1.3) | 0.6 (0.2-1) | 0.8 (0.3-1.2) | 0.22 (-0.54-0.98) |
| Chronic myeloid leukemia | Commonwealth of the Bahamas | 1.6 (1.3-1.9) | 0.8 (0.7-1) | 2.4 (1.9-3) | 0.6 (0.5-0.7) | -0.01 (-0.67-0.66) |
| Chronic myeloid leukemia | Cook Islands | 0.1 (0-0.1) | 0.4 (0.3-0.6) | 0.1 (0-0.1) | 0.3 (0.1-0.5) | -0.33 (-0.79-0.14) |
| Chronic myeloid leukemia | Czech Republic | 89.8 (75.1-107.5) | 0.7 (0.6-0.8) | 87.8 (62.5-121) | 0.4 (0.3-0.6) | -0.06 (-0.98-0.87) |
| Chronic myeloid leukemia | Democratic People's Republic of Korea | 74 (38.7-122.6) | 0.4 (0.2-0.6) | 90.6 (44.4-179.2) | 0.3 (0.1-0.6) | -0.03 (-0.83-0.78) |
| Chronic myeloid leukemia | Democratic Republic of Sao Tome and Principe | 0 (0-0.1) | 0 (0-0.1) | 0 (0-0.1) | 0 (0-0.1) | -0.05 (-0.34-0.24) |
| Chronic myeloid leukemia | Democratic Republic of the Congo | 20.3 (7.8-41.4) | 0.1 (0-0.2) | 31 (10.3-63.4) | 0.1 (0-0.1) | 0.05 (-0.47-0.57) |
| Chronic myeloid leukemia | Democratic Republic of Timor-Leste | 3.4 (1.5-6.8) | 0.7 (0.3-1.4) | 4.7 (2.5-8.4) | 0.5 (0.2-0.9) | -0.24 (-1.02-0.54) |
| Chronic myeloid leukemia | Democratic Socialist Republic of Sri Lanka | 51.6 (28-98.7) | 0.4 (0.2-0.8) | 91.7 (43.3-159.5) | 0.4 (0.2-0.6) | -0.53 (-1.32-0.27) |
| Chronic myeloid leukemia | Dominican Republic | 11.8 (5.7-17.4) | 0.3 (0.1-0.4) | 23.5 (11.3-38) | 0.2 (0.1-0.4) | 0.02 (-0.63-0.67) |
| Chronic myeloid leukemia | Eastern Republic of Uruguay | 50.1 (43.2-58.5) | 1.4 (1.2-1.6) | 34.2 (28.8-40.8) | 0.7 (0.6-0.8) | -0.12 (-0.95-0.72) |
| Chronic myeloid leukemia | Federal Democratic Republic of Ethiopia | 142.2 (57.5-284.9) | 0.5 (0.2-1.1) | 147.7 (55.4-416.1) | 0.2 (0.1-0.7) | -1 (-1.91--0.09) |
| Chronic myeloid leukemia | Federal Democratic Republic of Nepal | 73.2 (36.7-124.3) | 0.6 (0.3-1.1) | 98.6 (49.7-174.8) | 0.4 (0.2-0.7) | -0.19 (-0.81-0.43) |
| Chronic myeloid leukemia | Federal Republic of Germany | 2477.7 (2085.8-3127.6) | 2.1 (1.8-2.6) | 1751.8 (1468.1-2062.6) | 1 (0.8-1.1) | -0.13 (-1.11-0.86) |
| Chronic myeloid leukemia | Federal Republic of Nigeria | 26.5 (9.8-64.8) | 0 (0-0.1) | 36.6 (17.5-68) | 0 (0-0.1) | 0.01 (-0.35-0.38) |
| Chronic myeloid leukemia | Federal Republic of Somalia | 8.2 (3.4-17) | 0.2 (0.1-0.4) | 20.2 (7.8-51.7) | 0.2 (0.1-0.5) | 0.2 (-0.37-0.78) |
| Chronic myeloid leukemia | Federated States of Micronesia | 0.9 (0.6-1.4) | 1.5 (1-2.2) | 1.2 (0.7-1.7) | 1.4 (0.9-2) | -0.23 (-1.09-0.63) |
| Chronic myeloid leukemia | Federative Republic of Brazil | 870.4 (823.3-918) | 0.8 (0.8-0.9) | 891.5 (808.9-988.6) | 0.4 (0.3-0.4) | -0.16 (-0.89-0.57) |
| Chronic myeloid leukemia | French Republic | 1389.5 (1258.4-1515.5) | 1.8 (1.7-2) | 1176.3 (958.8-1445.9) | 0.9 (0.8-1.1) | 0.07 (-0.86-1) |
| Chronic myeloid leukemia | Gabonese Republic | 0.6 (0.3-1.4) | 0.1 (0-0.2) | 0.8 (0.3-1.6) | 0.1 (0-0.1) | 0.06 (-0.52-0.65) |
| Chronic myeloid leukemia | Georgia | 27.8 (17.6-40.4) | 0.5 (0.3-0.7) | 17.2 (11.5-24) | 0.3 (0.2-0.5) | -0.11 (-0.84-0.63) |
| Chronic myeloid leukemia | Grand Duchy of Luxembourg | 9.3 (8.6-10) | 1.8 (1.7-1.9) | 7.1 (6.2-8.2) | 0.7 (0.6-0.8) | -0.52 (-1.55-0.53) |
| Chronic myeloid leukemia | Greenland | 0.2 (0.1-0.2) | 0.4 (0.1-0.6) | 0.1 (0-0.3) | 0.2 (0-0.4) | -0.45 (-1.1-0.2) |
| Chronic myeloid leukemia | Grenada | 0.6 (0.5-0.7) | 0.8 (0.7-1) | 0.7 (0.6-0.8) | 0.6 (0.5-0.7) | 0.11 (-0.57-0.79) |
| Chronic myeloid leukemia | Guam | 1.3 (1-1.7) | 1.4 (1.1-1.9) | 2 (1.5-2.7) | 1 (0.8-1.4) | -0.18 (-0.97-0.61) |
| Chronic myeloid leukemia | Hashemite Kingdom of Jordan | 15.2 (8.5-24.4) | 1 (0.6-1.7) | 27.9 (14.6-49.5) | 0.4 (0.2-0.6) | -0.28 (-1.31-0.75) |
| Chronic myeloid leukemia | Hellenic Republic | 305.7 (279.6-332.5) | 2.2 (2-2.3) | 221.3 (193.6-248.7) | 1 (0.9-1.1) | -0.59 (-1.44-0.28) |
| Chronic myeloid leukemia | Hungary | 178.6 (157.2-204.4) | 1.3 (1.1-1.5) | 113.3 (87.6-148.4) | 0.7 (0.5-0.9) | -0.25 (-1.18-0.69) |
| Chronic myeloid leukemia | Independent State of Papua New Guinea | 28.6 (14.3-46.6) | 1.1 (0.6-1.7) | 67.5 (35.9-105.6) | 0.9 (0.5-1.4) | -0.2 (-0.93-0.53) |
| Chronic myeloid leukemia | Independent State of Samoa | 1.6 (1-2.5) | 1.7 (1.1-2.5) | 2.7 (1.7-3.9) | 1.8 (1.1-2.5) | -0.18 (-1.09-0.74) |
| Chronic myeloid leukemia | Ireland | 60.7 (55.3-66.2) | 1.5 (1.4-1.6) | 41.4 (34.6-49.7) | 0.6 (0.5-0.7) | -0.25 (-1.25-0.76) |
| Chronic myeloid leukemia | Islamic Republic of Afghanistan | 113 (31.6-234.7) | 1.4 (0.4-2.8) | 185.9 (73.7-335.6) | 1.1 (0.5-2) | -0.13 (-1.06-0.81) |
| Chronic myeloid leukemia | Islamic Republic of Iran | 354.8 (70.8-508.5) | 1 (0.2-1.4) | 560.9 (123.7-822.9) | 0.7 (0.2-1) | -0.14 (-0.95-0.68) |
| Chronic myeloid leukemia | Islamic Republic of Mauritania | 0.5 (0.2-1.1) | 0 (0-0.1) | 0.7 (0.2-1.6) | 0 (0-0.1) | 0.19 (-0.17-0.56) |
| Chronic myeloid leukemia | Islamic Republic of Pakistan | 395.1 (229.9-656.2) | 0.6 (0.3-0.9) | 672.4 (414-1019) | 0.4 (0.3-0.7) | -0.1 (-0.81-0.61) |
| Chronic myeloid leukemia | Jamaica | 5.5 (4.2-6.8) | 0.3 (0.2-0.4) | 10.7 (7.8-14.4) | 0.3 (0.3-0.5) | 0.23 (-0.5-0.96) |
| Chronic myeloid leukemia | Japan | 2051.6 (1918.5-2199.1) | 1.3 (1.2-1.4) | 1145.3 (979-1272.8) | 0.4 (0.4-0.5) | -0.42 (-1.32-0.48) |
| Chronic myeloid leukemia | Kingdom of Bahrain | 2.1 (1.1-3.3) | 1 (0.5-1.8) | 5.1 (1.8-8.7) | 0.5 (0.2-1) | -0.93 (-1.92-0.06) |
| Chronic myeloid leukemia | Kingdom of Belgium | 234.1 (197.2-267.7) | 1.6 (1.4-1.9) | 147.7 (119-186.5) | 0.7 (0.6-0.9) | -0.5 (-1.41-0.41) |
| Chronic myeloid leukemia | Kingdom of Bhutan | 2 (0.9-3.7) | 0.5 (0.3-1) | 2.1 (0.9-4.4) | 0.3 (0.2-0.7) | -0.28 (-0.94-0.38) |
| Chronic myeloid leukemia | Kingdom of Cambodia | 73.7 (36.4-127.1) | 1.2 (0.6-2) | 111.2 (55.1-190.7) | 0.8 (0.4-1.4) | -0.4 (-1.26-0.47) |
| Chronic myeloid leukemia | Kingdom of Denmark | 95.6 (84.4-106.6) | 1.3 (1.1-1.4) | 111.1 (91.7-137.8) | 1 (0.9-1.3) | -0.1 (-1.08-0.9) |
| Chronic myeloid leukemia | Kingdom of Eswatini | 0.2 (0-0.4) | 0 (0-0.1) | 0.3 (0.1-0.6) | 0 (0-0.1) | 0.38 (-0.47-1.24) |
| Chronic myeloid leukemia | Kingdom of Lesotho | 0.3 (0.1-0.7) | 0 (0-0.1) | 0.6 (0.2-1) | 0 (0-0.1) | 0.96 (0.16-1.75) |
| Chronic myeloid leukemia | Kingdom of Morocco | 24 (13.4-44.1) | 0.1 (0.1-0.3) | 32 (14.8-66.4) | 0.1 (0-0.2) | -0.02 (-0.4-0.35) |
| Chronic myeloid leukemia | Kingdom of Norway | 51.3 (47-55.5) | 0.8 (0.7-0.9) | 38.5 (33.2-44.5) | 0.4 (0.4-0.5) | 0.14 (-0.8-1.08) |
| Chronic myeloid leukemia | Kingdom of Saudi Arabia | 44.1 (17.8-127.4) | 0.6 (0.2-1.6) | 198.5 (90.2-623) | 0.7 (0.3-2.1) | 0.78 (0.15-1.41) |
| Chronic myeloid leukemia | Kingdom of Spain | 813.9 (730.4-895.4) | 1.7 (1.5-1.8) | 525.7 (422.8-637.1) | 0.6 (0.5-0.7) | -0.38 (-1.32-0.58) |
| Chronic myeloid leukemia | Kingdom of Sweden | 171.7 (152.5-191.7) | 1.3 (1.2-1.5) | 107.4 (89.8-128.5) | 0.6 (0.5-0.7) | -0.5 (-1.52-0.54) |
| Chronic myeloid leukemia | Kingdom of Thailand | 390.3 (182.2-590.2) | 1 (0.4-1.4) | 619.5 (294.7-978.9) | 0.7 (0.3-1) | -0.2 (-1.05-0.66) |
| Chronic myeloid leukemia | Kingdom of the Netherlands | 279.5 (247.5-305.3) | 1.5 (1.3-1.6) | 210.7 (180.4-247.9) | 0.7 (0.6-0.8) | -0.46 (-1.44-0.52) |
| Chronic myeloid leukemia | Kingdom of Tonga | 0.4 (0.2-0.6) | 0.5 (0.3-0.9) | 0.4 (0.3-0.6) | 0.5 (0.3-0.7) | -0.1 (-0.83-0.65) |
| Chronic myeloid leukemia | Kyrgyz Republic | 19.5 (16.1-23) | 0.5 (0.4-0.6) | 15.7 (12.1-20) | 0.3 (0.2-0.3) | -0.61 (-1.2--0.02) |
| Chronic myeloid leukemia | Lao People's Democratic Republic | 36.2 (14.5-67) | 1.3 (0.6-2.2) | 35 (18.6-63.3) | 0.6 (0.3-1.1) | -0.71 (-1.56-0.15) |
| Chronic myeloid leukemia | Lebanese Republic | 18.4 (8.7-30) | 0.9 (0.4-1.4) | 37 (13.6-63) | 0.6 (0.2-1) | 0.23 (-0.62-1.08) |
| Chronic myeloid leukemia | Malaysia | 40 (19.4-65.6) | 0.3 (0.2-0.6) | 78.1 (34.3-134) | 0.3 (0.1-0.4) | -0.14 (-0.97-0.7) |
| Chronic myeloid leukemia | Mongolia | 3.7 (1.8-6.9) | 0.3 (0.1-0.5) | 5.4 (2.9-8.5) | 0.2 (0.1-0.3) | -0.33 (-1.01-0.37) |
| Chronic myeloid leukemia | Montenegro | 2.7 (0.8-5.6) | 0.4 (0.1-0.9) | 3.2 (1.2-5.5) | 0.4 (0.1-0.6) | 0.28 (-0.62-1.2) |
| Chronic myeloid leukemia | New Zealand | 78.8 (69.3-88.3) | 2.1 (1.8-2.3) | 47.2 (40-55.8) | 0.6 (0.5-0.8) | -0.17 (-1.15-0.82) |
| Chronic myeloid leukemia | North Macedonia | 0 (0-0) | 0 (0-0) | 0 (0-0) | 0 (0-0) | 0.23 (-0.91-1.38) |
| Chronic myeloid leukemia | Northern Mariana Islands | 0.2 (0.1-0.4) | 0.9 (0.6-1.5) | 0.4 (0.3-0.6) | 0.7 (0.5-1) | -0.43 (-1.2-0.34) |
| Chronic myeloid leukemia | Palestine | 6.8 (3.9-11.9) | 0.7 (0.4-1.3) | 11.1 (6.6-18.1) | 0.4 (0.3-0.7) | -0.45 (-1.39-0.5) |
| Chronic myeloid leukemia | People's Democratic Republic of Algeria | 48.5 (22.3-73.3) | 0.3 (0.2-0.5) | 80.5 (31.7-132) | 0.2 (0.1-0.4) | -0.14 (-0.8-0.53) |
| Chronic myeloid leukemia | People's Republic of Bangladesh | 463.7 (238.5-726.7) | 0.7 (0.3-1) | 461.4 (194.8-881.8) | 0.3 (0.1-0.6) | -0.5 (-1.16-0.15) |
| Chronic myeloid leukemia | People's Republic of China | 3915.2 (1815.6-5747) | 0.4 (0.2-0.5) | 3849.9 (2086.9-6105.4) | 0.2 (0.1-0.3) | 0.02 (-0.91-0.96) |
| Chronic myeloid leukemia | Plurinational State of Bolivia | 37.6 (19.8-62.8) | 0.9 (0.5-1.5) | 55 (30.2-84.5) | 0.6 (0.3-0.9) | -0.32 (-1.21-0.57) |
| Chronic myeloid leukemia | Portuguese Republic | 169.1 (150.1-184) | 1.3 (1.2-1.5) | 139.6 (115-168.7) | 0.6 (0.5-0.8) | 0 (-0.92-0.93) |
| Chronic myeloid leukemia | Principality of Andorra | 0.9 (0.4-1.5) | 1.6 (0.7-2.6) | 1.2 (0.5-2.5) | 0.9 (0.4-1.8) | -0.58 (-1.69-0.54) |
| Chronic myeloid leukemia | Principality of Monaco | 0.9 (0.4-1.5) | 1.5 (0.7-2.6) | 1.3 (0.6-2.3) | 1.8 (0.9-3.2) | 0.43 (-0.8-1.67) |
| Chronic myeloid leukemia | Puerto Rico | 36.5 (31.4-41.9) | 1 (0.9-1.2) | 35.8 (28.4-44.8) | 0.6 (0.5-0.7) | -0.23 (-1.03-0.58) |
| Chronic myeloid leukemia | Republic of Albania | 5.8 (1.6-14.5) | 0.3 (0.1-0.7) | 9.1 (2.3-22.7) | 0.2 (0.1-0.6) | 0.18 (-0.57-0.94) |
| Chronic myeloid leukemia | Republic of Angola | 7.2 (2.5-17.7) | 0.1 (0-0.2) | 12.8 (4.8-29) | 0.1 (0-0.2) | -0.04 (-0.59-0.51) |
| Chronic myeloid leukemia | Republic of Armenia | 26.6 (21-32.6) | 0.9 (0.7-1.1) | 15.4 (12.4-19.2) | 0.4 (0.3-0.5) | -0.36 (-1.1-0.38) |
| Chronic myeloid leukemia | Republic of Austria | 185.3 (167.7-205.8) | 1.7 (1.5-1.9) | 142.5 (118.6-170.2) | 0.9 (0.7-1) | -0.08 (-1.07-0.91) |
| Chronic myeloid leukemia | Republic of Azerbaijan | 25.2 (12.9-45.3) | 0.4 (0.2-0.8) | 33.2 (12.5-70.2) | 0.3 (0.1-0.6) | -0.47 (-1.22-0.3) |
| Chronic myeloid leukemia | Republic of Belarus | 74.8 (48-108.3) | 0.6 (0.4-0.9) | 91 (67.7-118.9) | 0.6 (0.5-0.8) | -0.11 (-0.97-0.76) |
| Chronic myeloid leukemia | Republic of Benin | 1.6 (0.5-3.8) | 0 (0-0.1) | 2.8 (1-7) | 0 (0-0.1) | 0.23 (-0.15-0.62) |
| Chronic myeloid leukemia | Republic of Botswana | 0.3 (0.1-0.8) | 0 (0-0.1) | 0.4 (0.1-1.3) | 0 (0-0.1) | 0 (-0.74-0.73) |
| Chronic myeloid leukemia | Republic of Bulgaria | 68.8 (48.9-92) | 0.6 (0.5-0.8) | 38 (27-53) | 0.3 (0.2-0.4) | 0.23 (-0.51-0.98) |
| Chronic myeloid leukemia | Republic of Burundi | 7.2 (3-16.8) | 0.2 (0.1-0.5) | 10.5 (3.7-25.9) | 0.1 (0-0.4) | -0.1 (-0.65-0.45) |
| Chronic myeloid leukemia | Republic of Cabo Verde | 0.2 (0.1-0.7) | 0.1 (0-0.3) | 0.3 (0.1-0.9) | 0.1 (0-0.2) | 0.35 (-0.28-0.99) |
| Chronic myeloid leukemia | Republic of Cameroon | 3.1 (1.2-7.2) | 0 (0-0.1) | 7.3 (3.4-14) | 0 (0-0.1) | 0.27 (-0.14-0.68) |
| Chronic myeloid leukemia | Republic of Chad | 1.9 (0.6-4.8) | 0 (0-0.1) | 4.7 (1.8-10.8) | 0 (0-0.1) | 0.4 (0.03-0.78) |
| Chronic myeloid leukemia | Republic of Chile | 106.8 (97.4-117) | 1 (0.9-1.1) | 121.6 (104.7-141.9) | 0.5 (0.4-0.6) | 0.06 (-0.67-0.79) |
| Chronic myeloid leukemia | Republic of Colombia | 185.2 (167-201.7) | 0.8 (0.8-0.9) | 266.4 (214.8-328) | 0.5 (0.4-0.6) | -0.05 (-0.85-0.76) |
| Chronic myeloid leukemia | Republic of Costa Rica | 26.1 (22.6-30.1) | 1.3 (1.1-1.5) | 45.9 (38.7-54.4) | 0.9 (0.7-1) | 0.26 (-0.56-1.08) |
| Chronic myeloid leukemia | République de Côte d’Ivoire | 2.1 (0.8-5) | 0 (0-0.1) | 3.9 (1.5-9.8) | 0 (0-0.1) | 0.02 (-0.28-0.32) |
| Chronic myeloid leukemia | Republic of Croatia | 67.8 (52.4-87.7) | 1.2 (0.9-1.5) | 42.7 (29.4-58.8) | 0.5 (0.4-0.7) | 0.2 (-0.77-1.17) |
| Chronic myeloid leukemia | Republic of Cuba | 142.7 (130.6-159.2) | 1.4 (1.2-1.5) | 194.4 (163.5-227.7) | 1.1 (0.9-1.3) | -0.16 (-0.92-0.61) |
| Chronic myeloid leukemia | Republic of Cyprus | 4.3 (2.5-6.9) | 0.6 (0.4-1) | 9.7 (4.9-15.4) | 0.5 (0.2-0.8) | 0.65 (-0.53-1.84) |
| Chronic myeloid leukemia | Republic of Djibouti | 0.3 (0.1-0.7) | 0.1 (0-0.3) | 1 (0.3-2.7) | 0.1 (0-0.3) | 0.27 (-0.3-0.83) |
| Chronic myeloid leukemia | Republic of Ecuador | 26.7 (22.6-32.8) | 0.4 (0.3-0.5) | 74.8 (57.1-96.2) | 0.4 (0.3-0.6) | 0.5 (-0.36-1.37) |
| Chronic myeloid leukemia | Republic of El Salvador | 11 (5.9-16) | 0.3 (0.2-0.4) | 17.7 (9.6-25.3) | 0.3 (0.2-0.4) | 0.24 (-0.59-1.07) |
| Chronic myeloid leukemia | Republic of Equatorial Guinea | 0.3 (0.1-0.5) | 0.1 (0-0.2) | 0.5 (0.1-1.1) | 0.1 (0-0.1) | -0.06 (-0.6-0.49) |
| Chronic myeloid leukemia | Republic of Estonia | 28.2 (22.6-34.8) | 1.5 (1.2-1.8) | 13.7 (10.7-17.5) | 0.6 (0.5-0.8) | -0.4 (-1.4-0.61) |
| Chronic myeloid leukemia | Republic of Fiji | 3.2 (1.3-5.2) | 0.7 (0.3-1.2) | 3.3 (1.6-5.2) | 0.4 (0.2-0.7) | -0.31 (-1.42-0.81) |
| Chronic myeloid leukemia | Republic of Finland | 53.7 (48.4-60.1) | 0.8 (0.7-0.9) | 51.2 (42.1-61.4) | 0.4 (0.4-0.5) | 0.09 (-0.78-0.98) |
| Chronic myeloid leukemia | Republic of Ghana | 6.7 (3.1-13.6) | 0.1 (0-0.1) | 6.6 (2.4-17.6) | 0 (0-0.1) | -0.72 (-1.21--0.23) |
| Chronic myeloid leukemia | Republic of Guatemala | 18.9 (15.4-22.2) | 0.4 (0.3-0.5) | 26.5 (22.8-31.2) | 0.2 (0.2-0.3) | 0.11 (-0.67-0.89) |
| Chronic myeloid leukemia | Republic of Guinea | 1.1 (0.3-2.9) | 0 (0-0) | 1.1 (0.3-2.9) | 0 (0-0) | -0.02 (-0.2-0.17) |
| Chronic myeloid leukemia | Republic of Guinea-Bissau | 0.5 (0.2-1.1) | 0.1 (0-0.2) | 0.6 (0.3-1.4) | 0.1 (0-0.1) | 0.26 (-0.18-0.7) |
| Chronic myeloid leukemia | Republic of Guyana | 5.9 (5.1-6.7) | 1.2 (1.1-1.4) | 5.2 (3.8-7.2) | 0.8 (0.6-1.1) | 0.19 (-0.46-0.84) |
| Chronic myeloid leukemia | Republic of Haiti | 63.1 (20.5-126.6) | 1.4 (0.6-2.2) | 76.9 (33.3-131.1) | 0.9 (0.4-1.4) | -0.31 (-1.05-0.44) |
| Chronic myeloid leukemia | Republic of Honduras | 19.5 (10.3-26.9) | 0.7 (0.4-1) | 39.9 (23.1-61.9) | 0.6 (0.3-0.9) | -0.11 (-0.91-0.69) |
| Chronic myeloid leukemia | Republic of Iceland | 2.9 (2.6-3.3) | 1 (0.9-1.2) | 3 (2.4-3.6) | 0.6 (0.5-0.7) | -0.09 (-1.04-0.86) |
| Chronic myeloid leukemia | Republic of India | 2933.5 (1836.5-4202.1) | 0.5 (0.3-0.7) | 4473.2 (2669.4-6446.6) | 0.4 (0.2-0.5) | -0.26 (-0.86-0.35) |
| Chronic myeloid leukemia | Republic of Indonesia | 773.3 (394.2-1188) | 0.6 (0.3-0.9) | 962.9 (455.1-1619.4) | 0.4 (0.2-0.6) | -0.1 (-0.93-0.74) |
| Chronic myeloid leukemia | Republic of Iraq | 303.7 (162.5-436.3) | 2.8 (1.5-4) | 788.1 (303.5-1190.3) | 2.9 (1.2-4.1) | 0.05 (-0.81-0.92) |
| Chronic myeloid leukemia | Republic of Italy | 1824.8 (1696.3-1973.1) | 2.3 (2.1-2.5) | 1174.3 (990.1-1372.6) | 0.9 (0.8-1) | -0.71 (-1.74-0.32) |
| Chronic myeloid leukemia | Republic of Kazakhstan | 70.6 (54.4-95) | 0.5 (0.4-0.7) | 51.1 (36.8-71.8) | 0.3 (0.2-0.4) | -0.33 (-1-0.35) |
| Chronic myeloid leukemia | Republic of Kenya | 15.3 (8-32.1) | 0.1 (0.1-0.3) | 33.7 (18-64) | 0.1 (0.1-0.2) | 0.32 (-0.15-0.79) |
| Chronic myeloid leukemia | Republic of Kiribati | 0.5 (0.3-0.8) | 1 (0.6-1.6) | 0.9 (0.6-1.3) | 1 (0.7-1.5) | -0.04 (-0.75-0.68) |
| Chronic myeloid leukemia | Republic of Korea | 334.3 (174.6-456.7) | 0.9 (0.5-1.2) | 320.7 (166.2-568.5) | 0.4 (0.2-0.7) | -0.01 (-0.86-0.85) |
| Chronic myeloid leukemia | Republic of Latvia | 35.2 (26.5-45.8) | 1 (0.8-1.3) | 19.3 (14.6-25.9) | 0.6 (0.4-0.8) | -0.47 (-1.42-0.49) |
| Chronic myeloid leukemia | Republic of Liberia | 1.1 (0.4-2.5) | 0 (0-0.1) | 1.3 (0.5-2.8) | 0 (0-0.1) | 0.28 (-0.09-0.66) |
| Chronic myeloid leukemia | Republic of Lithuania | 63.8 (50.3-80.7) | 1.5 (1.2-1.9) | 32.4 (25.5-41.3) | 0.7 (0.5-0.8) | -0.45 (-1.37-0.48) |
| Chronic myeloid leukemia | Republic of Madagascar | 9.5 (5-17.3) | 0.1 (0.1-0.3) | 16.5 (7.2-31.9) | 0.1 (0-0.2) | 0.05 (-0.44-0.54) |
| Chronic myeloid leukemia | Republic of Malawi | 3.1 (1.5-6.1) | 0.1 (0-0.1) | 4.5 (1.9-8.9) | 0 (0-0.1) | -0.06 (-0.36-0.23) |
| Chronic myeloid leukemia | Republic of Maldives | 0.6 (0.2-1.4) | 0.4 (0.2-0.9) | 0.5 (0.3-0.9) | 0.1 (0.1-0.2) | -0.85 (-1.59--0.1) |
| Chronic myeloid leukemia | Republic of Mali | 4.3 (1.1-11) | 0.1 (0-0.1) | 4.7 (1.3-12.9) | 0 (0-0.1) | -0.09 (-0.41-0.23) |
| Chronic myeloid leukemia | Republic of Malta | 6.1 (5.4-6.9) | 1.5 (1.3-1.7) | 5.5 (4.3-6.8) | 0.7 (0.5-0.8) | -0.05 (-0.94-0.85) |
| Chronic myeloid leukemia | Republic of Mauritius | 12.2 (10.6-13.4) | 1.4 (1.2-1.5) | 7.3 (6.6-8) | 0.4 (0.4-0.5) | -0.66 (-1.44-0.13) |
| Chronic myeloid leukemia | Republic of Moldova | 14.6 (12.5-17.5) | 0.3 (0.3-0.4) | 10.3 (8.9-12.2) | 0.2 (0.2-0.2) | -0.54 (-1.25-0.18) |
| Chronic myeloid leukemia | Republic of Mozambique | 17 (8.2-36) | 0.2 (0.1-0.4) | 28.5 (12.9-59.6) | 0.2 (0.1-0.4) | 0.18 (-0.48-0.84) |
| Chronic myeloid leukemia | Republic of Namibia | 0.2 (0.1-0.5) | 0 (0-0.1) | 0.3 (0.1-0.8) | 0 (0-0) | 0.04 (-0.59-0.67) |
| Chronic myeloid leukemia | Republic of Nauru | 0.1 (0.1-0.1) | 1.6 (1-2.2) | 0.1 (0.1-0.2) | 1.8 (1-2.6) | -0.17 (-1.1-0.77) |
| Chronic myeloid leukemia | Republic of Nicaragua | 12.3 (7.4-16.9) | 0.5 (0.3-0.8) | 22.5 (13.9-34.7) | 0.4 (0.3-0.6) | -0.02 (-0.79-0.74) |
| Chronic myeloid leukemia | Republic of Niue | 0 (0-0) | 1 (0.7-1.5) | 0 (0-0) | 1.2 (0.8-1.7) | 0.05 (-0.76-0.86) |
| Chronic myeloid leukemia | Republic of Palau | 0.1 (0.1-0.2) | 0.8 (0.5-1.3) | 0.1 (0.1-0.2) | 0.7 (0.5-1) | -0.06 (-0.58-0.46) |
| Chronic myeloid leukemia | Republic of Panama | 11 (9.9-12.5) | 0.6 (0.6-0.7) | 20.2 (16.1-25.1) | 0.5 (0.4-0.6) | 0.32 (-0.48-1.12) |
| Chronic myeloid leukemia | Republic of Paraguay | 19.3 (10.7-27.5) | 0.8 (0.4-1.1) | 37.8 (21.3-58.1) | 0.6 (0.4-1) | 0.42 (-0.32-1.16) |
| Chronic myeloid leukemia | Republic of Peru | 64.5 (41.2-107.9) | 0.4 (0.3-0.7) | 147.9 (72.4-229) | 0.4 (0.2-0.7) | 0.24 (-0.58-1.06) |
| Chronic myeloid leukemia | Republic of Poland | 621.3 (469.5-792.5) | 1.4 (1.1-1.8) | 277.7 (245.9-313.3) | 0.4 (0.4-0.5) | -0.2 (-1.1-0.71) |
| Chronic myeloid leukemia | Republic of Rwanda | 10.5 (5.1-20.9) | 0.3 (0.1-0.5) | 11.4 (4-28.2) | 0.1 (0.1-0.3) | -0.42 (-1-0.16) |
| Chronic myeloid leukemia | Republic of San Marino | 0.4 (0.2-0.7) | 1.3 (0.6-2.1) | 0.4 (0.2-0.8) | 0.6 (0.3-1.3) | -0.71 (-1.9-0.49) |
| Chronic myeloid leukemia | Republic of Senegal | 2.2 (0.7-5.3) | 0 (0-0.1) | 3.2 (0.9-8.8) | 0 (0-0.1) | 0.26 (-0.13-0.65) |
| Chronic myeloid leukemia | Republic of Serbia | 42.9 (21.7-61.8) | 0.4 (0.2-0.6) | 46.1 (28.9-70) | 0.3 (0.2-0.5) | 0.05 (-0.8-0.91) |
| Chronic myeloid leukemia | Republic of Seychelles | 0.6 (0.3-0.8) | 0.9 (0.5-1.3) | 0.7 (0.4-0.9) | 0.6 (0.3-0.8) | -0.24 (-0.96-0.5) |
| Chronic myeloid leukemia | Republic of Sierra Leone | 1.6 (0.6-4.2) | 0 (0-0.1) | 2.3 (0.9-5.1) | 0 (0-0.1) | 0.29 (-0.08-0.67) |
| Chronic myeloid leukemia | Republic of Singapore | 29.5 (27.3-32.1) | 1.1 (1-1.2) | 33.9 (30.3-39.1) | 0.4 (0.4-0.5) | -0.07 (-0.99-0.85) |
| Chronic myeloid leukemia | Republic of Slovenia | 36.2 (30.7-42.2) | 1.5 (1.3-1.7) | 45.9 (35.3-57.6) | 1.2 (0.9-1.4) | 0.57 (-0.5-1.65) |
| Chronic myeloid leukemia | Republic of South Africa | 13.2 (5.6-25.8) | 0 (0-0.1) | 15.9 (7.9-29.6) | 0 (0-0.1) | 0.18 (-0.61-0.97) |
| Chronic myeloid leukemia | Republic of South Sudan | 5.1 (2.3-12.6) | 0.1 (0.1-0.3) | 8.9 (3.7-20.5) | 0.2 (0.1-0.3) | 0.36 (-0.2-0.92) |
| Chronic myeloid leukemia | Republic of Sudan | 119.7 (39.6-234.9) | 0.8 (0.3-1.7) | 132.2 (65-216.1) | 0.5 (0.2-0.8) | -0.17 (-1-0.66) |
| Chronic myeloid leukemia | Republic of Suriname | 1.9 (1.1-2.5) | 0.7 (0.4-0.9) | 2.7 (1.6-4.1) | 0.4 (0.3-0.7) | -0.07 (-0.69-0.55) |
| Chronic myeloid leukemia | Republic of Tajikistan | 14.7 (7.8-24) | 0.4 (0.2-0.6) | 13.3 (6.1-24.4) | 0.2 (0.1-0.3) | -0.74 (-1.39--0.08) |
| Chronic myeloid leukemia | Republic of the Congo | 1.4 (0.7-2.9) | 0.1 (0-0.2) | 2.5 (1.2-5.6) | 0.1 (0-0.1) | -0.09 (-0.67-0.5) |
| Chronic myeloid leukemia | Republic of the Gambia | 0.1 (0-0.3) | 0 (0-0) | 0.2 (0.1-0.6) | 0 (0-0) | 0 (-0.22-0.23) |
| Chronic myeloid leukemia | Republic of the Marshall Islands | 0.3 (0.2-0.4) | 1.2 (0.8-1.6) | 0.6 (0.3-0.9) | 1.2 (0.7-1.8) | -0.02 (-0.85-0.81) |
| Chronic myeloid leukemia | Republic of the Niger | 3.6 (0.8-12.3) | 0 (0-0.1) | 5.3 (1.5-14) | 0 (0-0.1) | 0.14 (-0.23-0.51) |
| Chronic myeloid leukemia | Republic of the Philippines | 340.2 (183.1-456) | 0.8 (0.4-1.1) | 564.8 (343.4-840.7) | 0.6 (0.4-0.9) | -0.29 (-1.12-0.54) |
| Chronic myeloid leukemia | Republic of the Union of Myanmar | 341.3 (143.4-618.5) | 1.2 (0.5-1.9) | 253.2 (135.3-423.7) | 0.5 (0.3-0.9) | -0.88 (-1.75-0.01) |
| Chronic myeloid leukemia | Republic of Trinidad and Tobago | 8.4 (7.2-9.3) | 1 (0.8-1.1) | 11.3 (8.5-14.8) | 0.6 (0.5-0.8) | -0.25 (-0.94-0.46) |
| Chronic myeloid leukemia | Republic of Tunisia | 20.4 (11.2-40.3) | 0.4 (0.2-0.8) | 36.3 (18.1-87.4) | 0.3 (0.1-0.7) | -0.16 (-0.83-0.51) |
| Chronic myeloid leukemia | Republic of Turkey | 352 (184.1-520.7) | 1 (0.5-1.4) | 421.9 (233.4-745.6) | 0.5 (0.3-0.8) | -0.44 (-1.35-0.48) |
| Chronic myeloid leukemia | Republic of Uganda | 9.3 (4.7-20.7) | 0.1 (0-0.2) | 23.4 (9.6-47.7) | 0.1 (0-0.2) | 0.02 (-0.45-0.5) |
| Chronic myeloid leukemia | Republic of Uzbekistan | 64.4 (47.5-89.5) | 0.4 (0.3-0.6) | 64.4 (43.3-94.8) | 0.2 (0.1-0.3) | -0.6 (-1.27-0.07) |
| Chronic myeloid leukemia | Republic of Vanuatu | 0.8 (0.5-1.3) | 1 (0.6-1.4) | 2.2 (1.3-3.1) | 1 (0.6-1.4) | -0.11 (-0.88-0.66) |
| Chronic myeloid leukemia | Republic of Yemen | 70.7 (23.8-135.9) | 0.9 (0.4-1.7) | 112.4 (51.7-177.1) | 0.6 (0.3-1) | -0.19 (-0.96-0.59) |
| Chronic myeloid leukemia | Republic of Zambia | 7.2 (4.2-13.7) | 0.2 (0.1-0.3) | 11.8 (4.4-25.1) | 0.1 (0-0.2) | 0.02 (-0.56-0.61) |
| Chronic myeloid leukemia | Republic of Zimbabwe | 1.5 (0.4-3.7) | 0 (0-0.1) | 3.7 (1.3-7.4) | 0 (0-0.1) | 0.62 (-0.2-1.46) |
| Chronic myeloid leukemia | Romania | 122.8 (104.7-147.3) | 0.5 (0.4-0.6) | 82.3 (69.5-96.6) | 0.3 (0.2-0.3) | 0.35 (-0.39-1.1) |
| Chronic myeloid leukemia | Russian Federation | 1023.4 (790.4-1251.8) | 0.6 (0.4-0.7) | 1078.4 (959.9-1230) | 0.5 (0.4-0.6) | -0.07 (-0.79-0.65) |
| Chronic myeloid leukemia | Saint Kitts and Nevis | 0.4 (0.4-0.5) | 1.2 (0.9-1.4) | 0.4 (0.3-0.5) | 0.6 (0.5-0.8) | -0.21 (-0.92-0.51) |
| Chronic myeloid leukemia | Saint Lucia | 1.2 (1-1.3) | 1.3 (1.1-1.5) | 1.2 (1-1.4) | 0.5 (0.4-0.6) | -0.51 (-1.14-0.13) |
| Chronic myeloid leukemia | Saint Vincent and the Grenadines | 0.7 (0.6-0.8) | 0.9 (0.8-1) | 0.8 (0.7-0.9) | 0.6 (0.5-0.7) | -0.2 (-0.88-0.48) |
| Chronic myeloid leukemia | Slovak Republic | 21.5 (11.8-33.7) | 0.4 (0.2-0.6) | 17.2 (8.9-30) | 0.2 (0.1-0.3) | 0.05 (-0.91-1.01) |
| Chronic myeloid leukemia | Socialist Republic of Viet Nam | 57.8 (24.9-94) | 0.1 (0.1-0.2) | 120.9 (58.9-214.9) | 0.1 (0.1-0.2) | -0.2 (-0.9-0.51) |
| Chronic myeloid leukemia | Solomon Islands | 2.2 (0.9-3.9) | 1.2 (0.5-1.9) | 6 (3.4-8.9) | 1.3 (0.7-1.9) | -0.04 (-0.85-0.77) |
| Chronic myeloid leukemia | State of Eritrea | 3.6 (1.6-7.1) | 0.2 (0.1-0.4) | 6.3 (2.6-13.1) | 0.2 (0.1-0.3) | 0.21 (-0.35-0.78) |
| Chronic myeloid leukemia | State of Israel | 64.3 (53.9-77) | 1.3 (1.1-1.6) | 69.7 (58.7-82.7) | 0.6 (0.5-0.7) | -0.15 (-1.27-0.99) |
| Chronic myeloid leukemia | State of Kuwait | 2.2 (1.6-2.9) | 0.3 (0.3-0.5) | 7.2 (5.4-9.1) | 0.2 (0.2-0.3) | -0.59 (-1.35-0.18) |
| Chronic myeloid leukemia | State of Libya | 15.1 (8.3-28.5) | 0.6 (0.3-1.3) | 30.2 (13.6-70.6) | 0.5 (0.2-1.2) | 0.47 (-0.43-1.38) |
| Chronic myeloid leukemia | State of Qatar | 1.4 (0.5-2.3) | 0.8 (0.4-1.4) | 7.2 (2.2-14.7) | 0.4 (0.2-0.9) | -0.67 (-1.49-0.15) |
| Chronic myeloid leukemia | Sultanate of Oman | 4.5 (1.9-11.9) | 0.4 (0.2-1.2) | 5.1 (1.9-17.3) | 0.2 (0.1-0.7) | -0.14 (-0.92-0.65) |
| Chronic myeloid leukemia | Swiss Confederation | 184.9 (156.2-228.2) | 1.9 (1.6-2.4) | 116.2 (95.6-141.3) | 0.7 (0.6-0.9) | -0.65 (-1.67-0.39) |
| Chronic myeloid leukemia | Syrian Arab Republic | 67.8 (39.3-120.7) | 1 (0.5-1.8) | 89.3 (43.1-182.4) | 0.7 (0.3-1.5) | -0.4 (-1.27-0.48) |
| Chronic myeloid leukemia | Taiwan (Province of China) | 80.4 (47.9-126.3) | 0.5 (0.3-0.7) | 158.3 (128.8-194.9) | 0.4 (0.4-0.5) | 0.82 (0-1.65) |
| Chronic myeloid leukemia | Togolese Republic | 0.9 (0.3-2.2) | 0 (0-0.1) | 1.9 (0.7-4.4) | 0 (0-0.1) | 0.32 (-0.06-0.7) |
| Chronic myeloid leukemia | Tokelau | 0 (0-0) | 1.2 (0.8-1.8) | 0 (0-0) | 1.3 (0.8-1.8) | -0.05 (-0.85-0.76) |
| Chronic myeloid leukemia | Turkmenistan | 10.2 (7.8-13.9) | 0.4 (0.3-0.5) | 10.8 (7-16.3) | 0.2 (0.1-0.3) | -0.31 (-0.95-0.33) |
| Chronic myeloid leukemia | Tuvalu | 0.1 (0.1-0.2) | 1.6 (1.1-2.3) | 0.1 (0.1-0.2) | 1.2 (0.7-1.6) | -0.3 (-1.12-0.52) |
| Chronic myeloid leukemia | Ukraine | 627.2 (459.6-861.1) | 1 (0.7-1.3) | 365.9 (253.4-496.6) | 0.5 (0.4-0.7) | -0.96 (-1.7--0.22) |
| Chronic myeloid leukemia | Union of the Comoros | 0.4 (0.2-0.9) | 0.1 (0.1-0.3) | 0.6 (0.2-1.4) | 0.1 (0-0.3) | 0.07 (-0.48-0.63) |
| Chronic myeloid leukemia | United Arab Emirates | 5.1 (2.6-9.1) | 0.9 (0.5-1.7) | 14.1 (7.4-23) | 0.4 (0.2-0.7) | 0.09 (-0.8-0.98) |
| Chronic myeloid leukemia | United Kingdom of Great Britain and Northern Ireland | 1467 (1383.4-1546) | 1.8 (1.7-1.9) | 697.9 (651.4-740.2) | 0.6 (0.6-0.7) | -0.54 (-1.58-0.5) |
| Chronic myeloid leukemia | United Mexican States | 331.9 (321.9-342.6) | 0.6 (0.6-0.6) | 492.7 (434.5-549.9) | 0.4 (0.3-0.4) | -0.05 (-0.9-0.82) |
| Chronic myeloid leukemia | United Republic of Tanzania | 25.1 (12.3-53.8) | 0.2 (0.1-0.4) | 42.5 (16.9-89.8) | 0.1 (0-0.3) | 0.06 (-0.5-0.62) |
| Chronic myeloid leukemia | United States of America | 7935.8 (7614.7-8255.3) | 2.6 (2.5-2.7) | 4610.8 (4277.4-4861.8) | 0.9 (0.8-0.9) | -0.85 (-1.95-0.26) |
| Chronic myeloid leukemia | United States Virgin Islands | 0.4 (0.2-0.6) | 0.5 (0.3-0.6) | 0.3 (0.2-0.5) | 0.2 (0.1-0.3) | -0.35 (-1.16-0.46) |
| Chronic myeloid leukemia | African Union | 804.52 (461.51-1461.48) | 0.2 (0.12-0.38) | 1276.62 (695.31-2187.2) | 0.16 (0.09-0.28) | -0.67 (-0.74--0.59) |
| Chronic myeloid leukemia | Association of Southeast Asian Nations | 2083.73 (1164.65-2969.01) | 0.67 (0.35-0.95) | 2781.07 (1540.88-3837.78) | 0.43 (0.24-0.58) | -1.64 (-1.75--1.53) |
| Chronic myeloid leukemia | Central Europe, Eastern Europe, and Central Asia | 3420.47 (2912.37-4035.3) | 0.74 (0.63-0.87) | 2630.54 (2364.14-3025.94) | 0.44 (0.39-0.5) | -2.27 (-2.6--1.94) |
| Chronic myeloid leukemia | Commonwealth | 6596.57 (4999.05-8496.41) | 0.69 (0.54-0.86) | 7641.27 (5047.67-10447.93) | 0.36 (0.24-0.49) | -2.33 (-2.51--2.15) |
| Chronic myeloid leukemia | European Union | 9414.95 (8694.31-10479.42) | 1.68 (1.56-1.86) | 6593.62 (5839.88-7446.22) | 0.76 (0.68-0.85) | -3.31 (-3.64--2.98) |
| Chronic myeloid leukemia | Four World Regions | 39396.68 (33210.63-45024.5) | 0.94 (0.81-1.07) | 35749.12 (27869.81-42496.2) | 0.43 (0.33-0.51) | -3.03 (-3.23--2.83) |
| Chronic myeloid leukemia | G20 | 32761.76 (28409.83-36625.31) | 1.04 (0.91-1.16) | 26699.66 (21974.07-31395.18) | 0.43 (0.35-0.51) | -3.39 (-3.63--3.16) |
| Chronic myeloid leukemia | Gulf Cooperation Council | 59.31 (29.62-152.29) | 0.55 (0.27-1.44) | 237.24 (120.4-704.44) | 0.54 (0.29-1.49) | 0.18 (-0.39-0.76) |
| Chronic myeloid leukemia | Health System Grouping Levels | 39435.79 (33250.99-45066.57) | 0.94 (0.81-1.07) | 35788.77 (27905.24-42536.08) | 0.43 (0.33-0.51) | -3.03 (-3.23--2.83) |
| Chronic myeloid leukemia | High SDI | 19285.65 (18200.56-20679.06) | 1.82 (1.72-1.95) | 12806.34 (11654.3-13982.83) | 0.69 (0.64-0.75) | -3.77 (-4.06--3.47) |
| Chronic myeloid leukemia | High-income | 21751.55 (20657.28-23143.98) | 1.93 (1.83-2.05) | 14019.23 (12749.37-15249.85) | 0.73 (0.68-0.79) | -3.84 (-4.15--3.53) |
| Chronic myeloid leukemia | High-middle SDI | 8438.63 (6967.65-9966.31) | 0.84 (0.7-0.99) | 7394.59 (5719.44-8846.91) | 0.41 (0.31-0.49) | -2.94 (-3.21--2.66) |
| Chronic myeloid leukemia | Latin America and Caribbean | 2050.19 (1921.48-2214.62) | 0.78 (0.73-0.84) | 2684.09 (2393.6-2969.26) | 0.43 (0.39-0.48) | -2.33 (-2.58--2.09) |
| Chronic myeloid leukemia | Low SDI | 1192.76 (628.25-1841.76) | 0.38 (0.22-0.64) | 1671.82 (953.06-2700.22) | 0.26 (0.15-0.44) | -1.31 (-1.4--1.22) |
| Chronic myeloid leukemia | Low-middle SDI | 4235.69 (2666.05-5884.92) | 0.54 (0.36-0.76) | 5679.43 (3637.88-8178.65) | 0.37 (0.24-0.53) | -1.27 (-1.31--1.22) |
| Chronic myeloid leukemia | Middle SDI | 6283.06 (4115.13-7822.31) | 0.49 (0.33-0.61) | 8236.59 (5394.67-10946.13) | 0.31 (0.21-0.41) | -1.7 (-1.81--1.59) |
| Chronic myeloid leukemia | Nordic Region | 375.4 (345.62-405.88) | 1.11 (1.03-1.2) | 311.31 (272.78-359.12) | 0.63 (0.55-0.71) | -2.32 (-2.65--1.99) |
| Chronic myeloid leukemia | North Africa and Middle East | 1781.06 (1022.09-2547.34) | 0.84 (0.52-1.26) | 3212.95 (1605.98-4552.55) | 0.65 (0.33-0.94) | -0.63 (-0.72--0.54) |
| Chronic myeloid leukemia | OECD Countries | 23225.19 (22027.54-24713.41) | 1.8 (1.71-1.91) | 15525.71 (14082.27-16843.6) | 0.68 (0.63-0.74) | -3.81 (-4.11--3.52) |
| Chronic myeloid leukemia | Organization of Islamic Cooperation | 3761.88 (2283.63-5061.91) | 0.56 (0.35-0.76) | 5749.63 (3127.02-7738.61) | 0.41 (0.23-0.56) | -0.98 (-1.01--0.95) |
| Chronic myeloid leukemia | Sahel Region | 183.93 (68.33-342.03) | 0.17 (0.08-0.35) | 240.87 (116.9-373.63) | 0.11 (0.06-0.19) | -1.58 (-1.68--1.47) |
| Chronic myeloid leukemia | South Asia | 3867.51 (2330.85-5585.72) | 0.54 (0.33-0.77) | 5707.62 (3329.9-8444.59) | 0.37 (0.22-0.56) | -1.21 (-1.3--1.12) |
| Chronic myeloid leukemia | Southeast Asia, East Asia, and Oceania | 6237.48 (3232.97-8460.27) | 0.44 (0.23-0.61) | 7045.68 (4096.88-10036.25) | 0.27 (0.16-0.38) | -1.92 (-2.05--1.79) |
| Chronic myeloid leukemia | Sub-Saharan Africa | 373.48 (184.65-685.3) | 0.12 (0.06-0.23) | 530.75 (244.35-1111.21) | 0.08 (0.04-0.17) | -1.53 (-1.59--1.47) |
| Chronic myeloid leukemia | WHO region | 39316.1 (33126.67-44939.79) | 0.94 (0.81-1.07) | 35590.62 (27722.8-42341.33) | 0.43 (0.33-0.51) | -3.04 (-3.24--2.84) |
| Chronic myeloid leukemia | World Bank Income Levels | 39435.69 (33250.91-45066.46) | 0.94 (0.81-1.07) | 35788.67 (27905.18-42535.96) | 0.43 (0.33-0.51) | -3.03 (-3.23--2.83) |
| Chronic myeloid leukemia | World Bank Regions | 39435.69 (33250.9-45066.46) | 0.94 (0.81-1.07) | 35788.66 (27905.18-42535.95) | 0.43 (0.33-0.51) | -3.03 (-3.23--2.83) |
| Other leukemia | American Samoa | 0.2 (0.2-0.4) | 0.9 (0.6-1.5) | 0.6 (0.3-0.8) | 1.3 (0.7-1.7) | 0.28 (-0.53-1.1) |
| Other leukemia | Antigua and Barbuda | 0.1 (0.1-0.1) | 0.2 (0.2-0.2) | 0.1 (0.1-0.1) | 0.1 (0.1-0.1) | -0.02 (-0.8-0.76) |
| Other leukemia | Arab Republic of Egypt | 183.2 (74.7-626) | 0.5 (0.2-1.9) | 445.1 (146.8-1195.1) | 0.6 (0.2-1.9) | 1.14 (0.33-1.95) |
| Other leukemia | Argentine Republic | 239.8 (202.3-279.6) | 0.8 (0.6-0.9) | 442.9 (361.8-518.6) | 0.8 (0.7-0.9) | -0.36 (-1.12-0.4) |
| Other leukemia | Australia | 147.6 (105.9-169.4) | 0.8 (0.6-0.9) | 193.7 (156.9-228.9) | 0.4 (0.3-0.5) | -0.18 (-1.19-0.84) |
| Other leukemia | Barbados | 5 (4.7-5.4) | 1.8 (1.6-1.9) | 5.5 (4.3-6.8) | 1.2 (0.9-1.5) | 0.15 (-0.64-0.95) |
| Other leukemia | Belize | 1.1 (1-1.4) | 0.9 (0.8-1.1) | 3.1 (2.7-3.6) | 0.9 (0.8-1) | 0.09 (-0.54-0.71) |
| Other leukemia | Bermuda | 0.8 (0.7-1) | 1.3 (1.1-1.5) | 0.9 (0.7-1.1) | 0.7 (0.6-0.9) | -0.15 (-0.92-0.62) |
| Other leukemia | Bolivarian Republic of Venezuela | 40.1 (36.4-44.3) | 0.3 (0.3-0.4) | 96.5 (70.2-127.6) | 0.3 (0.2-0.4) | -0.04 (-0.78-0.72) |
| Other leukemia | Bosnia and Herzegovina | 14.7 (4.8-22.7) | 0.4 (0.1-0.6) | 20.4 (6.5-32.5) | 0.3 (0.1-0.5) | 0.51 (-0.29-1.31) |
| Other leukemia | Brunei Darussalam | 1.6 (1-2.4) | 1.2 (0.8-1.8) | 3.6 (1.9-5) | 1 (0.5-1.3) | -0.24 (-1.06-0.59) |
| Other leukemia | Burkina Faso | 5 (1.9-7.8) | 0.1 (0-0.1) | 19.1 (4.5-34) | 0.1 (0-0.3) | 0.29 (-0.1-0.67) |
| Other leukemia | Canada | 146.4 (132.6-159) | 0.4 (0.4-0.5) | 321.4 (279.4-359.4) | 0.4 (0.4-0.5) | -0.44 (-1.56-0.68) |
| Other leukemia | Central African Republic | 1 (0.4-1.8) | 0.1 (0-0.1) | 2.5 (0.7-4.1) | 0.1 (0-0.2) | -0.05 (-0.64-0.54) |
| Other leukemia | Commonwealth of Dominica | 0.4 (0.2-0.5) | 0.6 (0.3-0.8) | 0.4 (0.3-0.6) | 0.6 (0.3-0.8) | 0.22 (-0.54-0.98) |
| Other leukemia | Commonwealth of the Bahamas | 1 (0.9-1.1) | 0.5 (0.5-0.6) | 1.6 (1.3-2) | 0.4 (0.3-0.5) | -0.01 (-0.67-0.66) |
| Other leukemia | Cook Islands | 0 (0-0.1) | 0.3 (0.1-0.5) | 0 (0-0.1) | 0.2 (0.1-0.3) | -0.33 (-0.79-0.14) |
| Other leukemia | Czech Republic | 200.6 (168.6-227.3) | 1.5 (1.2-1.7) | 251.5 (193.5-313.4) | 1.2 (0.9-1.5) | -0.06 (-0.98-0.87) |
| Other leukemia | Democratic People's Republic of Korea | 119.5 (68.9-190.4) | 0.6 (0.4-1) | 206.4 (118.3-354) | 0.6 (0.4-1.1) | -0.03 (-0.83-0.78) |
| Other leukemia | Democratic Republic of Sao Tome and Principe | 0 (0-0) | 0 (0-0.1) | 0.1 (0-0.1) | 0 (0-0.1) | -0.05 (-0.34-0.24) |
| Other leukemia | Democratic Republic of the Congo | 9.4 (3.5-16.5) | 0.1 (0-0.1) | 30.9 (8-60.6) | 0.1 (0-0.1) | 0.05 (-0.47-0.57) |
| Other leukemia | Democratic Republic of Timor-Leste | 2.5 (1.1-5.8) | 0.6 (0.3-1.5) | 5.5 (3-13.4) | 0.6 (0.3-1.4) | -0.24 (-1.02-0.54) |
| Other leukemia | Democratic Socialist Republic of Sri Lanka | 168.2 (90.4-222.2) | 1.4 (0.8-1.8) | 191.4 (104.3-332.5) | 0.7 (0.4-1.3) | -0.53 (-1.32-0.27) |
| Other leukemia | Dominican Republic | 41.7 (23.9-58.3) | 0.8 (0.5-1.1) | 71 (45.6-106.6) | 0.7 (0.4-1) | 0.02 (-0.63-0.67) |
| Other leukemia | Eastern Republic of Uruguay | 21.7 (18.4-25.3) | 0.6 (0.5-0.7) | 37.4 (31.6-43.1) | 0.7 (0.6-0.8) | -0.12 (-0.95-0.72) |
| Other leukemia | Federal Democratic Republic of Ethiopia | 702.1 (300.3-1183.7) | 2.2 (1.2-3.5) | 919.1 (393-1772) | 1.4 (0.6-2.5) | -1 (-1.91--0.09) |
| Other leukemia | Federal Democratic Republic of Nepal | 130.2 (70.5-213.3) | 1 (0.6-1.5) | 210.3 (127.3-324.6) | 0.9 (0.5-1.3) | -0.19 (-0.81-0.43) |
| Other leukemia | Federal Republic of Germany | 435.4 (364.2-520.2) | 0.4 (0.3-0.4) | 978.6 (774.3-1180) | 0.5 (0.4-0.6) | -0.13 (-1.11-0.86) |
| Other leukemia | Federal Republic of Nigeria | 74.2 (27.4-117.6) | 0.1 (0-0.2) | 142.5 (46.2-222.8) | 0.1 (0-0.2) | 0.01 (-0.35-0.38) |
| Other leukemia | Federal Republic of Somalia | 18 (6.9-44.9) | 0.4 (0.2-1) | 61.3 (31-106.2) | 0.6 (0.3-1) | 0.2 (-0.37-0.78) |
| Other leukemia | Federated States of Micronesia | 0.3 (0.1-0.7) | 0.7 (0.3-1.2) | 0.4 (0.2-0.8) | 0.5 (0.2-1.1) | -0.23 (-1.09-0.63) |
| Other leukemia | Federative Republic of Brazil | 432.4 (396.6-460.2) | 0.5 (0.4-0.5) | 1040.6 (929.9-1114.5) | 0.4 (0.4-0.5) | -0.16 (-0.89-0.57) |
| Other leukemia | French Republic | 741 (633.3-824.3) | 0.9 (0.8-1) | 1218.5 (987.6-1433.8) | 0.9 (0.7-1) | 0.07 (-0.86-1) |
| Other leukemia | Gabonese Republic | 0.3 (0.1-0.5) | 0.1 (0-0.1) | 0.8 (0.2-1.5) | 0.1 (0-0.1) | 0.06 (-0.52-0.65) |
| Other leukemia | Georgia | 89.2 (75.7-103.2) | 1.5 (1.3-1.7) | 73.8 (63.2-85.1) | 1.4 (1.2-1.6) | -0.11 (-0.84-0.63) |
| Other leukemia | Grand Duchy of Luxembourg | 4.9 (4.5-5.4) | 0.9 (0.9-1) | 6.7 (5.7-7.8) | 0.6 (0.5-0.7) | -0.52 (-1.55-0.53) |
| Other leukemia | Greenland | 0.2 (0.1-0.5) | 0.6 (0.4-1.5) | 0.3 (0.2-0.7) | 0.5 (0.3-1.1) | -0.45 (-1.1-0.2) |
| Other leukemia | Grenada | 1.1 (1-1.3) | 1.5 (1.3-1.7) | 1.5 (1.3-1.7) | 1.4 (1.2-1.5) | 0.11 (-0.57-0.79) |
| Other leukemia | Guam | 0.1 (0-0.2) | 0.2 (0-0.3) | 0.1 (0-0.3) | 0 (0-0.1) | -0.18 (-0.97-0.61) |
| Other leukemia | Hashemite Kingdom of Jordan | 12.8 (6.3-22.4) | 0.6 (0.3-1.2) | 46.6 (18.7-77.3) | 0.6 (0.2-0.9) | -0.28 (-1.31-0.75) |
| Other leukemia | Hellenic Republic | 278 (250-302.2) | 1.9 (1.8-2.1) | 378.7 (332.8-420.5) | 1.6 (1.5-1.8) | -0.59 (-1.44-0.28) |
| Other leukemia | Hungary | 85.5 (77-94.9) | 0.6 (0.5-0.7) | 97 (79.3-117.3) | 0.5 (0.4-0.6) | -0.25 (-1.18-0.69) |
| Other leukemia | Independent State of Papua New Guinea | 8.4 (1.9-19.1) | 0.5 (0.1-1) | 16.9 (3.8-38.7) | 0.3 (0.1-0.8) | -0.2 (-0.93-0.53) |
| Other leukemia | Independent State of Samoa | 0.7 (0.3-1.6) | 0.8 (0.4-1.8) | 0.9 (0.4-2.2) | 0.6 (0.3-1.5) | -0.18 (-1.09-0.74) |
| Other leukemia | Ireland | 25 (22.1-28.1) | 0.6 (0.5-0.7) | 32.6 (27.4-38.1) | 0.4 (0.4-0.5) | -0.25 (-1.25-0.76) |
| Other leukemia | Islamic Republic of Afghanistan | 74.6 (17.4-223.2) | 1 (0.3-3.1) | 140.3 (48.9-355.7) | 0.9 (0.3-2.4) | -0.13 (-1.06-0.81) |
| Other leukemia | Islamic Republic of Iran | 305.7 (74.5-464.2) | 0.9 (0.2-1.3) | 508.5 (135.4-717.6) | 0.7 (0.2-0.9) | -0.14 (-0.95-0.68) |
| Other leukemia | Islamic Republic of Mauritania | 1.1 (0.5-1.5) | 0.1 (0-0.1) | 3.3 (0.9-6.5) | 0.1 (0-0.2) | 0.19 (-0.17-0.56) |
| Other leukemia | Islamic Republic of Pakistan | 797 (463.1-1205.1) | 1.1 (0.6-1.5) | 1632.4 (1054.9-2460.6) | 1 (0.7-1.6) | -0.1 (-0.81-0.61) |
| Other leukemia | Jamaica | 30.4 (27.3-33.8) | 1.6 (1.5-1.8) | 47.8 (35.8-63.2) | 1.5 (1.2-2.1) | 0.23 (-0.5-0.96) |
| Other leukemia | Japan | 1021.6 (959-1082.5) | 0.6 (0.6-0.7) | 3244 (2791.3-3531.7) | 1 (0.9-1.1) | -0.42 (-1.32-0.48) |
| Other leukemia | Kingdom of Bahrain | 6.9 (4.2-9.5) | 3.4 (2.1-4.5) | 19.9 (12.4-28.4) | 2.2 (1.5-3.1) | -0.93 (-1.92-0.06) |
| Other leukemia | Kingdom of Belgium | 209.1 (174-241.6) | 1.4 (1.2-1.6) | 198.9 (163.4-236.2) | 0.9 (0.7-1) | -0.5 (-1.41-0.41) |
| Other leukemia | Kingdom of Bhutan | 3.9 (1.7-6.4) | 1 (0.6-1.6) | 5.4 (3.2-8.5) | 0.9 (0.5-1.3) | -0.28 (-0.94-0.38) |
| Other leukemia | Kingdom of Cambodia | 54 (23.9-131.7) | 1 (0.5-2.3) | 102.8 (54.2-252.1) | 0.8 (0.4-1.9) | -0.4 (-1.26-0.47) |
| Other leukemia | Kingdom of Denmark | 78.5 (66.2-88.1) | 1.1 (1-1.3) | 89.4 (73.4-106.7) | 0.8 (0.6-0.9) | -0.1 (-1.08-0.9) |
| Other leukemia | Kingdom of Eswatini | 0.2 (0.1-0.5) | 0.1 (0-0.1) | 0.5 (0.2-0.9) | 0.1 (0-0.1) | 0.38 (-0.47-1.24) |
| Other leukemia | Kingdom of Lesotho | 0.5 (0.2-1.1) | 0.1 (0-0.1) | 1 (0.4-1.7) | 0.1 (0-0.2) | 0.96 (0.16-1.75) |
| Other leukemia | Kingdom of Morocco | 24.4 (10-75.7) | 0.1 (0.1-0.4) | 40.7 (13.7-115.3) | 0.1 (0-0.3) | -0.02 (-0.4-0.35) |
| Other leukemia | Kingdom of Norway | 24.9 (22.9-26.6) | 0.4 (0.4-0.4) | 50.9 (44.7-56.3) | 0.5 (0.5-0.6) | 0.14 (-0.8-1.08) |
| Other leukemia | Kingdom of Saudi Arabia | 47.4 (17-94.4) | 0.6 (0.2-1.2) | 195.5 (74.1-314.8) | 0.7 (0.3-1.2) | 0.78 (0.15-1.41) |
| Other leukemia | Kingdom of Spain | 535.9 (472.5-592.8) | 1 (0.9-1.2) | 739.4 (602.8-872.9) | 0.8 (0.6-0.9) | -0.38 (-1.32-0.58) |
| Other leukemia | Kingdom of Sweden | 26.9 (22.2-30.9) | 0.2 (0.2-0.2) | 87.7 (70.2-105.8) | 0.4 (0.3-0.5) | -0.5 (-1.52-0.54) |
| Other leukemia | Kingdom of Thailand | 270.2 (143.5-416.5) | 0.7 (0.4-1) | 421.4 (252.7-864.9) | 0.4 (0.3-0.9) | -0.2 (-1.05-0.66) |
| Other leukemia | Kingdom of the Netherlands | 100.6 (78.8-114.8) | 0.5 (0.4-0.6) | 219.4 (142.1-277) | 0.6 (0.4-0.8) | -0.46 (-1.44-0.52) |
| Other leukemia | Kingdom of Tonga | 0.3 (0.1-0.7) | 0.6 (0.2-1.4) | 0.4 (0.2-1) | 0.5 (0.2-1.2) | -0.1 (-0.83-0.65) |
| Other leukemia | Kyrgyz Republic | 40.2 (34.3-47.5) | 1.1 (1-1.3) | 29.6 (23.7-37.4) | 0.5 (0.4-0.6) | -0.61 (-1.2--0.02) |
| Other leukemia | Lao People's Democratic Republic | 23.1 (8.9-56.9) | 0.9 (0.4-2.3) | 33.9 (17.3-84.9) | 0.6 (0.3-1.6) | -0.71 (-1.56-0.15) |
| Other leukemia | Lebanese Republic | 13.8 (7.1-33.9) | 0.6 (0.3-1.5) | 29.4 (15.5-60.5) | 0.5 (0.3-1) | 0.23 (-0.62-1.08) |
| Other leukemia | Malaysia | 155.5 (112-281.9) | 1.4 (1-2.6) | 377 (263.5-737.1) | 1.3 (0.9-2.5) | -0.14 (-0.97-0.7) |
| Other leukemia | Mongolia | 4.3 (2.3-6.7) | 0.3 (0.2-0.5) | 6.4 (3.3-10) | 0.2 (0.1-0.4) | -0.33 (-1.01-0.37) |
| Other leukemia | Montenegro | 2.5 (0.8-4) | 0.4 (0.1-0.7) | 3.5 (1.4-5.3) | 0.4 (0.1-0.6) | 0.28 (-0.62-1.2) |
| Other leukemia | New Zealand | 24.3 (19.8-29.1) | 0.6 (0.5-0.7) | 138.2 (113.2-165.5) | 1.6 (1.4-2) | -0.17 (-1.15-0.82) |
| Other leukemia | North Macedonia | 0 (0-0) | 0 (0-0) | 0 (0-0) | 0 (0-0) | 0.23 (-0.91-1.38) |
| Other leukemia | Northern Mariana Islands | 0.2 (0.1-0.4) | 1.1 (0.3-1.8) | 0.8 (0.2-1.1) | 1.6 (0.4-2.2) | -0.43 (-1.2-0.34) |
| Other leukemia | Palestine | 50.8 (34.3-71) | 4.3 (3-5.8) | 97.4 (63.2-135.7) | 3.1 (2.2-4.3) | -0.45 (-1.39-0.5) |
| Other leukemia | People's Democratic Republic of Algeria | 258 (169.4-356.6) | 1.5 (1.1-2) | 446.8 (292.9-632.7) | 1.2 (0.8-1.7) | -0.14 (-0.8-0.53) |
| Other leukemia | People's Republic of Bangladesh | 808.6 (448.2-1325.9) | 1.1 (0.6-1.7) | 1113.7 (611.3-1756) | 0.8 (0.4-1.2) | -0.5 (-1.16-0.15) |
| Other leukemia | People's Republic of China | 12173.7 (6913-16817.6) | 1.2 (0.7-1.7) | 16484.5 (8183.5-23107.5) | 0.9 (0.4-1.3) | 0.02 (-0.91-0.96) |
| Other leukemia | Plurinational State of Bolivia | 58.7 (33.2-89.5) | 1.5 (0.9-2.3) | 114.3 (66.4-169.3) | 1.2 (0.7-1.7) | -0.32 (-1.21-0.57) |
| Other leukemia | Portuguese Republic | 63.8 (55.1-71.7) | 0.5 (0.4-0.6) | 107.9 (83.7-132.5) | 0.4 (0.3-0.5) | 0 (-0.92-0.93) |
| Other leukemia | Principality of Andorra | 0.3 (0.2-0.9) | 0.6 (0.3-1.6) | 0.8 (0.4-1.6) | 0.5 (0.2-1.1) | -0.58 (-1.69-0.54) |
| Other leukemia | Principality of Monaco | 0.5 (0.3-1) | 0.8 (0.4-1.5) | 1.1 (0.5-2.4) | 1.2 (0.6-2.7) | 0.43 (-0.8-1.67) |
| Other leukemia | Puerto Rico | 10.5 (8.6-12.4) | 0.3 (0.2-0.3) | 27.9 (21.4-34.8) | 0.5 (0.4-0.6) | -0.23 (-1.03-0.58) |
| Other leukemia | Republic of Albania | 8.8 (3.1-14.4) | 0.4 (0.2-0.7) | 13.4 (4.7-24) | 0.3 (0.1-0.6) | 0.18 (-0.57-0.94) |
| Other leukemia | Republic of Angola | 2.7 (1.1-4.6) | 0.1 (0-0.1) | 9.6 (2.4-18.2) | 0.1 (0-0.1) | -0.04 (-0.59-0.51) |
| Other leukemia | Republic of Armenia | 20.8 (16.7-25.2) | 0.7 (0.5-0.8) | 31.1 (24.7-38.1) | 0.8 (0.6-0.9) | -0.36 (-1.1-0.38) |
| Other leukemia | Republic of Austria | 67 (60.6-74.7) | 0.6 (0.5-0.7) | 104.2 (83.6-126) | 0.6 (0.5-0.7) | -0.08 (-1.07-0.91) |
| Other leukemia | Republic of Azerbaijan | 57.2 (30.4-84.4) | 1 (0.5-1.4) | 69.4 (35.9-116.4) | 0.7 (0.4-1.1) | -0.47 (-1.22-0.3) |
| Other leukemia | Republic of Belarus | 207 (167.3-258.3) | 1.7 (1.4-2.1) | 78.4 (59.9-98.1) | 0.5 (0.4-0.7) | -0.11 (-0.97-0.76) |
| Other leukemia | Republic of Benin | 2.3 (1-3.4) | 0.1 (0-0.1) | 10.6 (2.7-17.7) | 0.1 (0-0.2) | 0.23 (-0.15-0.62) |
| Other leukemia | Republic of Botswana | 0.4 (0.1-0.7) | 0.1 (0-0.1) | 0.8 (0.2-1.7) | 0 (0-0.1) | 0 (-0.74-0.73) |
| Other leukemia | Republic of Bulgaria | 108.7 (87.9-130.4) | 1 (0.8-1.2) | 117.9 (86.6-152.6) | 0.9 (0.6-1.2) | 0.23 (-0.51-0.98) |
| Other leukemia | Republic of Burundi | 13.7 (6.3-31.9) | 0.4 (0.2-0.8) | 33.2 (13.8-59.1) | 0.4 (0.2-0.7) | -0.1 (-0.65-0.45) |
| Other leukemia | Republic of Cabo Verde | 0.6 (0.3-0.9) | 0.3 (0.1-0.4) | 2.5 (1-3.8) | 0.4 (0.2-0.6) | 0.35 (-0.28-0.99) |
| Other leukemia | Republic of Cameroon | 6 (2.9-8.3) | 0.1 (0-0.1) | 32.8 (8.5-58.3) | 0.2 (0-0.3) | 0.27 (-0.14-0.68) |
| Other leukemia | Republic of Chad | 2.6 (1-4.3) | 0.1 (0-0.1) | 13.2 (3-24.4) | 0.2 (0-0.3) | 0.4 (0.03-0.78) |
| Other leukemia | Republic of Chile | 56.2 (48.7-62.3) | 0.5 (0.5-0.6) | 112.9 (93.5-132.4) | 0.5 (0.4-0.5) | 0.06 (-0.67-0.79) |
| Other leukemia | Republic of Colombia | 165.5 (146.9-181.4) | 0.8 (0.7-0.9) | 277.9 (226.9-338.3) | 0.5 (0.4-0.6) | -0.05 (-0.85-0.76) |
| Other leukemia | Republic of Costa Rica | 8 (6.7-9.4) | 0.4 (0.3-0.5) | 22.5 (17.8-27.2) | 0.4 (0.3-0.5) | 0.26 (-0.56-1.08) |
| Other leukemia | République de Côte d’Ivoire | 6.4 (2.5-9.3) | 0.1 (0-0.2) | 17.8 (6.2-30.4) | 0.1 (0-0.2) | 0.02 (-0.28-0.32) |
| Other leukemia | Republic of Croatia | 46 (34.8-59.4) | 0.8 (0.6-1) | 42.8 (29.3-59.9) | 0.5 (0.3-0.7) | 0.2 (-0.77-1.17) |
| Other leukemia | Republic of Cuba | 30.4 (27.1-33.8) | 0.3 (0.3-0.3) | 39.8 (33.8-46.3) | 0.2 (0.2-0.3) | -0.16 (-0.92-0.61) |
| Other leukemia | Republic of Cyprus | 1.9 (1.1-3.8) | 0.3 (0.2-0.5) | 4.2 (2.1-6.5) | 0.2 (0.1-0.3) | 0.65 (-0.53-1.84) |
| Other leukemia | Republic of Djibouti | 0.7 (0.3-1.8) | 0.3 (0.1-0.7) | 3.5 (1.6-6.2) | 0.4 (0.2-0.7) | 0.27 (-0.3-0.83) |
| Other leukemia | Republic of Ecuador | 65.9 (56.1-75.8) | 1 (0.9-1.2) | 107.8 (82.4-139) | 0.6 (0.5-0.8) | 0.5 (-0.36-1.37) |
| Other leukemia | Republic of El Salvador | 41.7 (27.3-59.4) | 1.2 (0.8-1.7) | 85.4 (55.7-115.5) | 1.4 (0.9-1.8) | 0.24 (-0.59-1.07) |
| Other leukemia | Republic of Equatorial Guinea | 0.1 (0.1-0.3) | 0.1 (0-0.1) | 0.4 (0.1-0.9) | 0.1 (0-0.1) | -0.06 (-0.6-0.49) |
| Other leukemia | Republic of Estonia | 18.5 (15.1-23.5) | 1 (0.8-1.3) | 12.3 (9.5-15.2) | 0.5 (0.4-0.6) | -0.4 (-1.4-0.61) |
| Other leukemia | Republic of Fiji | 0.3 (0.1-0.5) | 0.1 (0-0.2) | 0.3 (0.1-0.5) | 0 (0-0.1) | -0.31 (-1.42-0.81) |
| Other leukemia | Republic of Finland | 61.4 (52.2-69.7) | 0.9 (0.8-1) | 97.4 (78.8-119.5) | 0.8 (0.6-0.9) | 0.09 (-0.78-0.98) |
| Other leukemia | Republic of Ghana | 23.4 (7.6-37.1) | 0.2 (0.1-0.4) | 45.6 (23.3-69.5) | 0.2 (0.1-0.3) | -0.72 (-1.21--0.23) |
| Other leukemia | Republic of Guatemala | 37.2 (33.8-41.1) | 0.9 (0.8-1) | 100.3 (85.7-117.5) | 0.8 (0.7-1) | 0.11 (-0.67-0.89) |
| Other leukemia | Republic of Guinea | 1.2 (0.5-1.8) | 0 (0-0) | 2.9 (1.1-4.3) | 0 (0-0.1) | -0.02 (-0.2-0.17) |
| Other leukemia | Republic of Guinea-Bissau | 0.7 (0.4-1.1) | 0.1 (0.1-0.2) | 2.4 (0.7-4.1) | 0.2 (0.1-0.3) | 0.26 (-0.18-0.7) |
| Other leukemia | Republic of Guyana | 2 (1.7-2.3) | 0.4 (0.4-0.5) | 2.5 (1.9-3.4) | 0.4 (0.3-0.5) | 0.19 (-0.46-0.84) |
| Other leukemia | Republic of Haiti | 56.2 (26.3-98.8) | 1.4 (0.7-2.1) | 101.2 (49.2-162.8) | 1.1 (0.6-1.8) | -0.31 (-1.05-0.44) |
| Other leukemia | Republic of Honduras | 14.4 (8.5-22.1) | 0.6 (0.3-0.9) | 37.5 (23.1-64.7) | 0.6 (0.3-1) | -0.11 (-0.91-0.69) |
| Other leukemia | Republic of Iceland | 2.1 (1.8-2.4) | 0.8 (0.6-0.9) | 3.3 (2.6-3.9) | 0.6 (0.5-0.7) | -0.09 (-1.04-0.86) |
| Other leukemia | Republic of India | 5048.1 (3178.1-7054.6) | 0.9 (0.6-1.2) | 9513.5 (6290.6-13186.6) | 0.8 (0.5-1.1) | -0.26 (-0.86-0.35) |
| Other leukemia | Republic of Indonesia | 750.9 (424.5-1867.1) | 0.6 (0.4-1.5) | 1520.6 (928-3867.1) | 0.6 (0.4-1.6) | -0.1 (-0.93-0.74) |
| Other leukemia | Republic of Iraq | 63.1 (20-225.1) | 0.5 (0.1-1.8) | 136.7 (47.7-434.3) | 0.5 (0.2-1.5) | 0.05 (-0.81-0.92) |
| Other leukemia | Republic of Italy | 974.6 (879.3-1058.8) | 1.2 (1-1.3) | 851.1 (709.8-962.7) | 0.6 (0.5-0.7) | -0.71 (-1.74-0.32) |
| Other leukemia | Republic of Kazakhstan | 140.4 (111.5-167.9) | 1 (0.8-1.2) | 127.6 (99.3-158.6) | 0.7 (0.5-0.9) | -0.33 (-1-0.35) |
| Other leukemia | Republic of Kenya | 46.6 (23.5-75.4) | 0.4 (0.2-0.7) | 127.6 (53.1-200.6) | 0.5 (0.2-0.7) | 0.32 (-0.15-0.79) |
| Other leukemia | Republic of Kiribati | 0.1 (0-0.2) | 0.3 (0.1-0.5) | 0.2 (0.1-0.4) | 0.3 (0.1-0.5) | -0.04 (-0.75-0.68) |
| Other leukemia | Republic of Korea | 111.3 (71.6-229) | 0.3 (0.2-0.7) | 192.1 (100.6-334.4) | 0.2 (0.1-0.4) | -0.01 (-0.86-0.85) |
| Other leukemia | Republic of Latvia | 29.1 (24.1-35) | 0.9 (0.7-1) | 16.7 (12.7-20.8) | 0.5 (0.4-0.6) | -0.47 (-1.42-0.49) |
| Other leukemia | Republic of Liberia | 1.3 (0.6-1.9) | 0.1 (0-0.1) | 5.4 (1.3-9.8) | 0.2 (0-0.3) | 0.28 (-0.09-0.66) |
| Other leukemia | Republic of Lithuania | 36.7 (30.1-46.6) | 0.9 (0.7-1.1) | 31.9 (25.1-38.7) | 0.6 (0.5-0.7) | -0.45 (-1.37-0.48) |
| Other leukemia | Republic of Madagascar | 23.9 (13.7-56.6) | 0.3 (0.2-0.6) | 63.2 (32.4-104.8) | 0.3 (0.2-0.5) | 0.05 (-0.44-0.54) |
| Other leukemia | Republic of Malawi | 9.3 (5.4-17.1) | 0.1 (0.1-0.2) | 16.9 (7.4-29.3) | 0.1 (0.1-0.2) | -0.06 (-0.36-0.23) |
| Other leukemia | Republic of Maldives | 1 (0.5-1.8) | 0.9 (0.5-1.5) | 1.4 (0.8-2.1) | 0.4 (0.2-0.6) | -0.85 (-1.59--0.1) |
| Other leukemia | Republic of Mali | 2.7 (1.1-4) | 0.1 (0-0.1) | 6.4 (2.3-9.7) | 0.1 (0-0.1) | -0.09 (-0.41-0.23) |
| Other leukemia | Republic of Malta | 2 (1.6-2.3) | 0.5 (0.4-0.5) | 3.4 (2.6-4.1) | 0.4 (0.3-0.4) | -0.05 (-0.94-0.85) |
| Other leukemia | Republic of Mauritius | 0 (0-0) | 0 (0-0) | 26.3 (24.2-28.3) | 1.5 (1.4-1.7) | -0.66 (-1.44-0.13) |
| Other leukemia | Republic of Moldova | 30.4 (26.6-33.7) | 0.7 (0.6-0.8) | 20.9 (18.1-24) | 0.4 (0.3-0.4) | -0.54 (-1.25-0.18) |
| Other leukemia | Republic of Mozambique | 61.6 (32.4-117.5) | 0.5 (0.3-1.1) | 150.3 (67.7-282.8) | 0.7 (0.4-1.2) | 0.18 (-0.48-0.84) |
| Other leukemia | Republic of Namibia | 0.3 (0.1-0.7) | 0 (0-0.1) | 0.6 (0.2-1.3) | 0 (0-0.1) | 0.04 (-0.59-0.67) |
| Other leukemia | Republic of Nauru | 0 (0-0.1) | 0.8 (0.4-1.5) | 0 (0-0.1) | 0.6 (0.3-1.2) | -0.17 (-1.1-0.77) |
| Other leukemia | Republic of Nicaragua | 6.2 (3.5-10) | 0.3 (0.2-0.5) | 16.8 (9.6-25.4) | 0.3 (0.2-0.5) | -0.02 (-0.79-0.74) |
| Other leukemia | Republic of Niue | 0 (0-0) | 0.5 (0.2-0.9) | 0 (0-0) | 0.4 (0.2-0.8) | 0.05 (-0.76-0.86) |
| Other leukemia | Republic of Palau | 0.1 (0-0.1) | 0.6 (0.4-1) | 0.1 (0.1-0.2) | 0.6 (0.4-1) | -0.06 (-0.58-0.46) |
| Other leukemia | Republic of Panama | 7.5 (6.6-8.2) | 0.4 (0.4-0.5) | 22 (17.5-27) | 0.5 (0.4-0.6) | 0.32 (-0.48-1.12) |
| Other leukemia | Republic of Paraguay | 7.7 (3.9-12.1) | 0.3 (0.2-0.5) | 24.4 (13.4-36.4) | 0.4 (0.2-0.6) | 0.42 (-0.32-1.16) |
| Other leukemia | Republic of Peru | 166.1 (108.3-228) | 1.1 (0.7-1.5) | 332.9 (201.1-476.7) | 1 (0.6-1.4) | 0.24 (-0.58-1.06) |
| Other leukemia | Republic of Poland | 469.4 (413.9-519.5) | 1.1 (1-1.2) | 494.4 (432.5-548.5) | 0.7 (0.6-0.8) | -0.2 (-1.1-0.71) |
| Other leukemia | Republic of Rwanda | 19.3 (9.1-43.9) | 0.4 (0.2-0.9) | 34.8 (15.8-62.9) | 0.4 (0.2-0.7) | -0.42 (-1-0.16) |
| Other leukemia | Republic of San Marino | 1.2 (0.8-1.8) | 3.5 (2.2-5.6) | 1.5 (0.8-2.3) | 2.1 (1.1-3.2) | -0.71 (-1.9-0.49) |
| Other leukemia | Republic of Senegal | 4.1 (1.6-6) | 0.1 (0-0.1) | 15.9 (4-28.7) | 0.1 (0-0.3) | 0.26 (-0.13-0.65) |
| Other leukemia | Republic of Serbia | 83.5 (44-109.8) | 0.8 (0.4-1.1) | 96.4 (53.3-140) | 0.6 (0.3-0.9) | 0.05 (-0.8-0.91) |
| Other leukemia | Republic of Seychelles | 1.2 (0.9-2) | 2 (1.4-3.4) | 1.8 (1.3-2.9) | 1.6 (1.2-2.6) | -0.24 (-0.96-0.5) |
| Other leukemia | Republic of Sierra Leone | 2.1 (0.8-3.2) | 0.1 (0-0.1) | 7.4 (1.7-13.6) | 0.1 (0-0.2) | 0.29 (-0.08-0.67) |
| Other leukemia | Republic of Singapore | 2.1 (1.9-2.3) | 0.1 (0.1-0.1) | 8.9 (7.7-10.2) | 0.1 (0.1-0.1) | -0.07 (-0.99-0.85) |
| Other leukemia | Republic of Slovenia | 9 (7.3-10.5) | 0.4 (0.3-0.4) | 18.8 (14.6-23.5) | 0.4 (0.3-0.5) | 0.57 (-0.5-1.65) |
| Other leukemia | Republic of South Africa | 22.2 (7.4-38.7) | 0.1 (0-0.2) | 40.6 (15.2-69.1) | 0.1 (0-0.1) | 0.18 (-0.61-0.97) |
| Other leukemia | Republic of South Sudan | 14.9 (6.5-37.1) | 0.3 (0.2-0.9) | 37.5 (19.2-65.6) | 0.6 (0.3-0.9) | 0.36 (-0.2-0.92) |
| Other leukemia | Republic of Sudan | 92 (29.5-258.9) | 0.6 (0.2-1.9) | 168 (73.4-422.6) | 0.6 (0.2-1.5) | -0.17 (-1-0.66) |
| Other leukemia | Republic of Suriname | 2.6 (1.4-3.4) | 0.9 (0.5-1.2) | 5 (3-6.9) | 0.8 (0.5-1.1) | -0.07 (-0.69-0.55) |
| Other leukemia | Republic of Tajikistan | 29.4 (13.7-43.4) | 0.8 (0.4-1.2) | 37.9 (16.1-67.2) | 0.5 (0.2-0.8) | -0.74 (-1.39--0.08) |
| Other leukemia | Republic of the Congo | 0.8 (0.4-1.4) | 0.1 (0-0.1) | 2.2 (0.7-4) | 0.1 (0-0.1) | -0.09 (-0.67-0.5) |
| Other leukemia | Republic of the Gambia | 0.3 (0.1-0.4) | 0.1 (0-0.1) | 0.9 (0.4-1.3) | 0.1 (0-0.1) | 0 (-0.22-0.23) |
| Other leukemia | Republic of the Marshall Islands | 0.1 (0-0.2) | 0.6 (0.2-1.2) | 0.2 (0.1-0.4) | 0.5 (0.2-1.1) | -0.02 (-0.85-0.81) |
| Other leukemia | Republic of the Niger | 3.8 (1.4-6.6) | 0.1 (0-0.2) | 15.4 (2.9-36.6) | 0.1 (0-0.3) | 0.14 (-0.23-0.51) |
| Other leukemia | Republic of the Philippines | 102.4 (35.2-133.1) | 0.3 (0.1-0.4) | 201.8 (84.6-271.3) | 0.2 (0.1-0.3) | -0.29 (-1.12-0.54) |
| Other leukemia | Republic of the Union of Myanmar | 264.9 (110.1-624.8) | 1 (0.4-2.3) | 315.6 (175.8-804.7) | 0.6 (0.3-1.6) | -0.88 (-1.75-0.01) |
| Other leukemia | Republic of Trinidad and Tobago | 7.2 (6.5-7.8) | 0.8 (0.7-0.9) | 8.2 (6.2-10.5) | 0.5 (0.3-0.6) | -0.25 (-0.94-0.46) |
| Other leukemia | Republic of Tunisia | 20.3 (9-60.4) | 0.3 (0.1-1) | 31 (9.5-100.5) | 0.3 (0.1-0.8) | -0.16 (-0.83-0.51) |
| Other leukemia | Republic of Turkey | 266.9 (155.3-423.3) | 0.7 (0.4-1.1) | 464.5 (229.2-693.5) | 0.5 (0.3-0.8) | -0.44 (-1.35-0.48) |
| Other leukemia | Republic of Uganda | 56.5 (32.3-86.3) | 0.4 (0.2-0.6) | 142.5 (50-278.5) | 0.5 (0.2-0.8) | 0.02 (-0.45-0.5) |
| Other leukemia | Republic of Uzbekistan | 165.2 (134.7-204.2) | 1.2 (0.9-1.4) | 220.2 (154.4-297.7) | 0.7 (0.5-0.9) | -0.6 (-1.27-0.07) |
| Other leukemia | Republic of Vanuatu | 0.4 (0.1-0.8) | 0.6 (0.2-1.2) | 0.8 (0.3-1.9) | 0.5 (0.2-1) | -0.11 (-0.88-0.66) |
| Other leukemia | Republic of Yemen | 52.9 (17-148.8) | 0.6 (0.2-1.9) | 111.8 (49.8-277.8) | 0.6 (0.2-1.5) | -0.19 (-0.96-0.59) |
| Other leukemia | Republic of Zambia | 19.8 (9.9-46.2) | 0.3 (0.2-0.8) | 52.5 (23.7-92.3) | 0.4 (0.2-0.8) | 0.02 (-0.56-0.61) |
| Other leukemia | Republic of Zimbabwe | 2.9 (0.9-6) | 0.1 (0-0.1) | 7.9 (2.5-14.8) | 0.1 (0-0.2) | 0.62 (-0.2-1.46) |
| Other leukemia | Romania | 254.7 (230.4-282.6) | 1 (0.9-1.1) | 314.3 (270.2-357.9) | 0.9 (0.8-1) | 0.35 (-0.39-1.1) |
| Other leukemia | Russian Federation | 1355.8 (1134-1627.9) | 0.8 (0.7-0.9) | 1550.5 (1339.6-1714.2) | 0.7 (0.6-0.8) | -0.07 (-0.79-0.65) |
| Other leukemia | Saint Kitts and Nevis | 0.8 (0.7-0.9) | 2.2 (1.9-2.4) | 0.9 (0.8-1.1) | 1.5 (1.2-1.8) | -0.21 (-0.92-0.51) |
| Other leukemia | Saint Lucia | 0.9 (0.8-1) | 0.9 (0.8-1) | 1.3 (1-1.6) | 0.6 (0.5-0.7) | -0.51 (-1.14-0.13) |
| Other leukemia | Saint Vincent and the Grenadines | 1.4 (1.3-1.5) | 1.7 (1.6-1.9) | 1.9 (1.7-2.2) | 1.4 (1.2-1.7) | -0.2 (-0.88-0.48) |
| Other leukemia | Slovak Republic | 64.9 (33.5-93.6) | 1.1 (0.6-1.6) | 59.8 (34-93.4) | 0.7 (0.4-1) | 0.05 (-0.91-1.01) |
| Other leukemia | Socialist Republic of Viet Nam | 298.3 (195.2-514.7) | 0.7 (0.5-1.2) | 515 (315.7-1037.2) | 0.5 (0.3-1) | -0.2 (-0.9-0.51) |
| Other leukemia | Solomon Islands | 1 (0.2-2) | 0.7 (0.2-1.4) | 2.1 (0.7-4.6) | 0.6 (0.2-1.1) | -0.04 (-0.85-0.77) |
| Other leukemia | State of Eritrea | 6.9 (3.3-16.7) | 0.3 (0.2-0.8) | 18.9 (10.5-30.7) | 0.5 (0.3-0.7) | 0.21 (-0.35-0.78) |
| Other leukemia | State of Israel | 15.1 (12.1-17.8) | 0.3 (0.3-0.4) | 49.7 (39-59) | 0.4 (0.3-0.5) | -0.15 (-1.27-0.99) |
| Other leukemia | State of Kuwait | 19.8 (16.2-24.7) | 2.1 (1.7-2.4) | 27.3 (19.6-35.3) | 0.9 (0.6-1.1) | -0.59 (-1.35-0.18) |
| Other leukemia | State of Libya | 14.7 (6.6-44.2) | 0.6 (0.2-1.8) | 30.3 (11.2-92.9) | 0.6 (0.2-1.7) | 0.47 (-0.43-1.38) |
| Other leukemia | State of Qatar | 3.9 (1.8-5.8) | 2.5 (1.2-3.7) | 20.9 (11.3-32.7) | 1.5 (0.9-2.3) | -0.67 (-1.49-0.15) |
| Other leukemia | Sultanate of Oman | 12 (5.8-19.8) | 1.2 (0.6-1.9) | 20.1 (11.2-30.1) | 0.9 (0.5-1.2) | -0.14 (-0.92-0.65) |
| Other leukemia | Swiss Confederation | 54.3 (45.3-64.9) | 0.5 (0.5-0.6) | 116.3 (88-143.4) | 0.6 (0.5-0.8) | -0.65 (-1.67-0.39) |
| Other leukemia | Syrian Arab Republic | 55.5 (22.8-176.5) | 0.7 (0.3-2.2) | 78.7 (20.8-249.5) | 0.6 (0.2-2) | -0.4 (-1.27-0.48) |
| Other leukemia | Taiwan (Province of China) | 39.3 (32.2-47.5) | 0.2 (0.2-0.3) | 122.1 (95-149.1) | 0.3 (0.3-0.4) | 0.82 (0-1.65) |
| Other leukemia | Togolese Republic | 1.5 (0.6-2.3) | 0.1 (0-0.1) | 8.8 (2.1-15.4) | 0.2 (0-0.3) | 0.32 (-0.06-0.7) |
| Other leukemia | Tokelau | 0 (0-0) | 0.5 (0.2-1) | 0 (0-0) | 0.4 (0.1-0.8) | -0.05 (-0.85-0.76) |
| Other leukemia | Turkmenistan | 20.1 (16-24.4) | 0.8 (0.7-1) | 28.7 (19.9-40.2) | 0.6 (0.4-0.8) | -0.31 (-0.95-0.33) |
| Other leukemia | Tuvalu | 0 (0-0.1) | 0.5 (0.2-1) | 0 (0-0.1) | 0.5 (0.2-0.9) | -0.3 (-1.12-0.52) |
| Other leukemia | Ukraine | 642.2 (530.2-777.9) | 1 (0.8-1.2) | 421.5 (302.3-557.7) | 0.6 (0.4-0.8) | -0.96 (-1.7--0.22) |
| Other leukemia | Union of the Comoros | 1 (0.6-2.4) | 0.3 (0.2-0.7) | 2.4 (1.2-4.1) | 0.4 (0.2-0.7) | 0.07 (-0.48-0.63) |
| Other leukemia | United Arab Emirates | 12.3 (6.2-18.5) | 1.8 (0.9-2.6) | 55 (25.7-88.3) | 1.2 (0.7-1.7) | 0.09 (-0.8-0.98) |
| Other leukemia | United Kingdom of Great Britain and Northern Ireland | 237.1 (221.8-249.4) | 0.3 (0.2-0.3) | 732.6 (658.1-786.9) | 0.5 (0.5-0.6) | -0.54 (-1.58-0.5) |
| Other leukemia | United Mexican States | 264.8 (253.3-272.7) | 0.5 (0.5-0.5) | 447.6 (397.7-499) | 0.4 (0.3-0.4) | -0.05 (-0.9-0.82) |
| Other leukemia | United Republic of Tanzania | 66.4 (37.3-158.2) | 0.3 (0.2-0.8) | 172.1 (77.3-314.8) | 0.4 (0.2-0.7) | 0.06 (-0.5-0.62) |
| Other leukemia | United States of America | 1129.7 (1041.3-1186.7) | 0.3 (0.3-0.4) | 2649.9 (2389.2-2809.4) | 0.5 (0.4-0.5) | -0.85 (-1.95-0.26) |
| Other leukemia | United States Virgin Islands | 0.2 (0.1-0.3) | 0.2 (0.1-0.3) | 0.2 (0.1-0.3) | 0.2 (0.1-0.3) | -0.35 (-1.16-0.46) |
| Other leukemia | African Union | 1834.66 (1114.01-3284.7) | 0.42 (0.28-0.76) | 3476.74 (1875.56-5585.14) | 0.39 (0.23-0.65) | -0.16 (-0.2--0.11) |
| Other leukemia | Association of Southeast Asian Nations | 1922.99 (1305.58-3911.66) | 0.67 (0.46-1.36) | 3500.63 (2497.2-7692.97) | 0.54 (0.39-1.19) | -0.8 (-0.97--0.64) |
| Other leukemia | Central Europe, Eastern Europe, and Central Asia | 4256.59 (3772.04-4761.14) | 0.92 (0.82-1.03) | 4309.72 (3780.81-4680.97) | 0.71 (0.62-0.77) | -0.65 (-0.83--0.46) |
| Other leukemia | Commonwealth | 8014.19 (5277.97-11068.04) | 0.76 (0.52-1.02) | 15328.77 (10593.07-20928.9) | 0.72 (0.5-0.97) | -0.23 (-0.29--0.17) |
| Other leukemia | European Union | 4928.91 (4549.46-5201.9) | 0.86 (0.79-0.9) | 6575.51 (5700.05-7201.33) | 0.7 (0.61-0.76) | -0.27 (-0.39--0.15) |
| Other leukemia | Four World Regions | 36633.32 (27247.93-47233.2) | 0.84 (0.64-1.08) | 59151.4 (42428.78-74660.57) | 0.71 (0.51-0.9) | -0.42 (-0.47--0.37) |
| Other leukemia | G20 | 28324.71 (20965.41-35688.26) | 0.88 (0.66-1.1) | 45610 (32914.58-55248.07) | 0.75 (0.54-0.92) | -0.36 (-0.41--0.3) |
| Other leukemia | Gulf Cooperation Council | 102.44 (53.5-157.01) | 0.87 (0.45-1.36) | 338.74 (167.43-481.02) | 0.86 (0.45-1.19) | 0.06 (-0.13-0.24) |
| Other leukemia | Health System Grouping Levels | 36645.41 (27258.72-47245.8) | 0.84 (0.64-1.08) | 59181.83 (42454.37-74687.7) | 0.71 (0.51-0.9) | -0.42 (-0.47--0.37) |
| Other leukemia | High SDI | 5845.47 (5384.45-6109.97) | 0.54 (0.5-0.57) | 12646.54 (11037.73-13694.51) | 0.62 (0.54-0.67) | 0.7 (0.61-0.79) |
| Other leukemia | High-income | 6847.36 (6340.13-7160.05) | 0.59 (0.54-0.61) | 13425.03 (11790.13-14472.32) | 0.62 (0.55-0.66) | 0.56 (0.44-0.68) |
| Other leukemia | High-middle SDI | 10589.44 (8069.25-12931.27) | 1.05 (0.8-1.27) | 14250.82 (9797.22-17294.92) | 0.81 (0.54-0.99) | -0.57 (-0.7--0.45) |
| Other leukemia | Latin America and Caribbean | 1516.66 (1321.29-1655.42) | 0.6 (0.53-0.65) | 3058.41 (2638.53-3436.7) | 0.5 (0.43-0.56) | -0.61 (-0.71--0.52) |
| Other leukemia | Low SDI | 2314.94 (1339.36-3576.2) | 0.72 (0.46-1.07) | 4057.19 (2223.91-6126.18) | 0.6 (0.35-0.89) | -0.68 (-0.74--0.62) |
| Other leukemia | Low-middle SDI | 6028.5 (4196.54-9270.04) | 0.78 (0.57-1.19) | 10635.04 (7692.06-16580.27) | 0.69 (0.5-1.08) | -0.41 (-0.45--0.36) |
| Other leukemia | Middle SDI | 11867.06 (7716.35-16196.4) | 0.92 (0.62-1.26) | 17592.24 (11172.41-23471) | 0.67 (0.42-0.89) | -0.97 (-1--0.94) |
| Other leukemia | Nordic Region | 193.98 (170.81-209.43) | 0.58 (0.52-0.63) | 329.01 (282.51-367.52) | 0.58 (0.5-0.65) | 1.02 (0.74-1.3) |
| Other leukemia | North Africa and Middle East | 1591.6 (1072.23-3043.99) | 0.71 (0.47-1.38) | 3117.49 (1766.39-5368.42) | 0.64 (0.38-1.1) | -0.03 (-0.14-0.07) |
| Other leukemia | OECD Countries | 8191.49 (7566.55-8543.78) | 0.62 (0.58-0.65) | 15110.59 (13308.04-16271.9) | 0.6 (0.54-0.65) | 0.17 (0.09-0.25) |
| Other leukemia | Organization of Islamic Cooperation | 4823.51 (3389.3-8065.97) | 0.7 (0.51-1.18) | 8957.63 (6278.28-14730.92) | 0.62 (0.45-1.03) | -0.31 (-0.36--0.27) |
| Other leukemia | Sahel Region | 228.79 (115.1-474.62) | 0.24 (0.13-0.48) | 547.47 (298.53-832.97) | 0.24 (0.13-0.38) | 0.15 (0.07-0.24) |
| Other leukemia | South Asia | 6787.74 (4185.43-9478.04) | 0.93 (0.59-1.28) | 12475.3 (8386.05-17610.46) | 0.81 (0.55-1.15) | -0.56 (-0.63--0.49) |
| Other leukemia | Southeast Asia, East Asia, and Oceania | 14441.16 (9202.15-20816.56) | 1.04 (0.69-1.52) | 20557.67 (12220.94-29415.5) | 0.78 (0.46-1.13) | -0.86 (-0.92--0.81) |
| Other leukemia | Sub-Saharan Africa | 1241.8 (704.13-2005.54) | 0.37 (0.23-0.57) | 2288.31 (945.51-3686.48) | 0.31 (0.14-0.5) | -0.6 (-0.63--0.58) |
| Other leukemia | WHO region | 36593.74 (27208.4-47196.73) | 0.84 (0.64-1.08) | 59028.98 (42323.25-74567.18) | 0.71 (0.51-0.9) | -0.42 (-0.47--0.37) |
| Other leukemia | World Bank Income Levels | 36645.36 (27258.69-47245.71) | 0.84 (0.64-1.08) | 59181.77 (42454.33-74687.59) | 0.71 (0.51-0.9) | -0.42 (-0.47--0.37) |
| Other leukemia | World Bank Regions | 36645.36 (27258.69-47245.71) | 0.84 (0.64-1.08) | 59181.77 (42454.33-74687.59) | 0.71 (0.51-0.9) | -0.42 (-0.47--0.37) |

Table S2. The number of leukemia deaths and age standardized mortality of leukemia caused by different reasons from 1990 to 2021.

| Cause | Location | 1990 | | 2021 | | 1990-2021 |
| --- | --- | --- | --- | --- | --- | --- |
|  |  | Number of deaths  No. ×10^3^ (95% UI) | ASMR per 100,000  No. (95% UI) | Number of deaths  No. ×10^3^ (95% UI) | ASMR per 100,000  No. (95% UI) | EAPC  No. (95% CI) |
| Leukemia | American Samoa | 1.1 (0.9-1.4) | 3.7 (3-4.5) | 1.6 (1.3-2) | 3.6 (2.7-4.5) | 0.18 (-0.62-1) |
| Leukemia | Antigua and Barbuda | 2.5 (2.3-2.7) | 4.4 (4-4.7) | 3.7 (3.5-3.9) | 3.8 (3.6-4) | -0.2 (-0.97-0.58) |
| Leukemia | Arab Republic of Egypt | 1883.7 (1562.9-2762.3) | 4.8 (3.9-7.7) | 4499.9 (3125.5-5676) | 6.4 (4.7-8.1) | 0.92 (0.11-1.74) |
| Leukemia | Argentine Republic | 1922.8 (1849-1993.3) | 6 (5.7-6.2) | 2382 (2197.5-2567.1) | 4.5 (4.1-4.8) | -0.52 (-1.27-0.24) |
| Leukemia | Australia | 1167.1 (1105.7-1217) | 6.2 (5.8-6.4) | 2261.9 (2015.5-2468.5) | 4.9 (4.4-5.3) | -0.68 (-1.46-0.1) |
| Leukemia | Barbados | 14.8 (14-15.6) | 5.2 (4.9-5.5) | 21.7 (16.9-26.5) | 4.8 (3.8-5.9) | -0.03 (-0.83-0.77) |
| Leukemia | Belize | 4.6 (4.3-5) | 2.9 (2.8-3.1) | 9.9 (8.8-11) | 2.9 (2.5-3.2) | -0.03 (-0.62-0.57) |
| Leukemia | Bermuda | 3.6 (3.3-3.8) | 5.9 (5.5-6.3) | 4 (3.4-4.8) | 3.3 (2.8-4) | -0.87 (-1.63--0.11) |
| Leukemia | Bolivarian Republic of Venezuela | 690 (665.7-714.1) | 4.5 (4.3-4.7) | 1172.5 (874.1-1524.8) | 4.3 (3.2-5.5) | -0.22 (-0.94-0.51) |
| Leukemia | Bosnia and Herzegovina | 148.9 (126.9-180.3) | 3.7 (3.1-4.5) | 204.9 (150.4-259.8) | 3.5 (2.6-4.5) | -0.03 (-0.73-0.67) |
| Leukemia | Brunei Darussalam | 10 (8.2-12.7) | 6 (4.9-7.5) | 16 (12-19.2) | 4.2 (3.1-5.1) | -0.39 (-1.21-0.45) |
| Leukemia | Burkina Faso | 96.5 (71.6-125.6) | 1.1 (0.9-1.4) | 246.4 (119.1-343.8) | 1.4 (0.7-1.9) | 0.26 (-0.12-0.65) |
| Leukemia | Canada | 2011 (1887.8-2093.5) | 6.4 (6-6.6) | 3220 (2856.9-3490.8) | 4.4 (4-4.8) | -0.6 (-1.44-0.24) |
| Leukemia | Central African Republic | 47.2 (32.6-66.2) | 2.5 (1.9-3.2) | 82.5 (51.5-118.3) | 2.4 (1.5-3.2) | -0.06 (-0.65-0.54) |
| Leukemia | Commonwealth of Dominica | 3.5 (3-4.2) | 5.5 (4.6-6.6) | 4.3 (3.2-5.3) | 5.8 (4.4-7.2) | 0.07 (-0.69-0.83) |
| Leukemia | Commonwealth of the Bahamas | 7.4 (6.8-8) | 3.7 (3.5-4) | 13.4 (10.8-16.5) | 3.4 (2.8-4.2) | -0.14 (-0.76-0.5) |
| Leukemia | Cook Islands | 0.2 (0.2-0.3) | 1.8 (1.3-2.2) | 0.3 (0.2-0.3) | 1.2 (0.8-1.5) | -0.42 (-0.88-0.03) |
| Leukemia | Czech Republic | 919.7 (865.8-977.2) | 7 (6.6-7.5) | 1095.2 (939.7-1263.6) | 5.1 (4.4-5.9) | -0.7 (-1.46-0.08) |
| Leukemia | Democratic People's Republic of Korea | 919.1 (639.9-1258.6) | 4.8 (3.4-6.5) | 1201.3 (869.1-1689.8) | 4.1 (2.9-5.6) | -0.24 (-1.03-0.56) |
| Leukemia | Democratic Republic of Sao Tome and Principe | 0.9 (0.7-1.2) | 0.8 (0.6-1) | 1 (0.7-1.6) | 0.7 (0.5-0.9) | -0.1 (-0.38-0.18) |
| Leukemia | Democratic Republic of the Congo | 518.1 (378.6-706.4) | 2 (1.4-2.7) | 1042 (655.3-1515.8) | 2 (1.2-3) | 0.02 (-0.51-0.54) |
| Leukemia | Democratic Republic of Timor-Leste | 30 (17.4-43.4) | 5.3 (3.6-7.1) | 48.4 (36-63.8) | 4.4 (3.3-5.8) | -0.3 (-1.08-0.49) |
| Leukemia | Democratic Socialist Republic of Sri Lanka | 697.7 (571.7-845) | 5.2 (4.3-6.5) | 772.2 (509-1068.1) | 3.1 (2.1-4.3) | -0.77 (-1.55-0.01) |
| Leukemia | Dominican Republic | 221.5 (183.7-270) | 3.7 (3-4.8) | 329.7 (251.4-439) | 3.2 (2.4-4.3) | -0.06 (-0.7-0.57) |
| Leukemia | Eastern Republic of Uruguay | 228.4 (216.3-242) | 6.3 (5.9-6.6) | 291.1 (262.7-315.5) | 5.6 (5.1-6) | -0.36 (-1.13-0.42) |
| Leukemia | Federal Democratic Republic of Ethiopia | 3535.6 (1854.1-5311.8) | 10.6 (7.1-14.5) | 4545 (3238.5-6594.2) | 6.8 (4.8-10.5) | -1.11 (-2.01--0.19) |
| Leukemia | Federal Democratic Republic of Nepal | 529.6 (355.3-712.6) | 3.6 (2.6-4.7) | 730.3 (534.8-998.4) | 2.9 (2.2-4) | -0.23 (-0.85-0.4) |
| Leukemia | Federal Republic of Germany | 7074.4 (6666.3-7400.8) | 6 (5.7-6.2) | 9495 (8381-10378.7) | 4.7 (4.3-5.1) | -0.55 (-1.36-0.27) |
| Leukemia | Federal Republic of Nigeria | 929.9 (650.9-1309) | 1.1 (0.8-1.5) | 1961 (1074.6-2663.2) | 1.1 (0.6-1.4) | -0.02 (-0.38-0.34) |
| Leukemia | Federal Republic of Somalia | 135.7 (86-198.3) | 2.7 (1.9-3.7) | 343 (220.6-492) | 2.9 (2-4.1) | 0.19 (-0.39-0.77) |
| Leukemia | Federated States of Micronesia | 3.5 (2.5-4.7) | 5.1 (3.6-6.9) | 3.5 (2.3-4.9) | 4.4 (3-6) | -0.28 (-1.15-0.59) |
| Leukemia | Federative Republic of Brazil | 5161.4 (4994.5-5339.3) | 4.6 (4.4-4.7) | 9171.1 (8619.6-9560.3) | 3.9 (3.6-4) | -0.3 (-1.02-0.43) |
| Leukemia | French Republic | 5348.3 (4999.1-5701.9) | 6.7 (6.3-7.1) | 7513.8 (6544.9-8362.1) | 4.9 (4.4-5.4) | -0.61 (-1.42-0.2) |
| Leukemia | Gabonese Republic | 15.4 (11.6-19.3) | 2.2 (1.6-2.9) | 26.5 (15.9-39.2) | 2.1 (1.3-3.1) | -0.02 (-0.6-0.57) |
| Leukemia | Georgia | 281.2 (260.6-304) | 4.9 (4.5-5.3) | 234 (210.6-260.2) | 4.6 (4.1-5.1) | -0.01 (-0.71-0.7) |
| Leukemia | Grand Duchy of Luxembourg | 40.5 (38.1-42.7) | 8 (7.6-8.4) | 56.9 (50.5-62.8) | 5.3 (4.7-5.9) | -0.95 (-1.9-0) |
| Leukemia | Greenland | 1.5 (1.1-1.8) | 4 (3.2-4.8) | 1.5 (1.1-2) | 2.5 (1.9-3.3) | -0.64 (-1.29-0.03) |
| Leukemia | Grenada | 3.8 (3.5-4.2) | 4.8 (4.3-5.3) | 4.8 (4.2-5.4) | 4.5 (3.9-5) | -0.03 (-0.68-0.62) |
| Leukemia | Guam | 3.5 (2.9-4.3) | 3.9 (3.2-4.8) | 5 (3.9-6) | 2.7 (2.1-3.2) | -0.36 (-1.11-0.38) |
| Leukemia | Hashemite Kingdom of Jordan | 168.4 (135.3-208.9) | 8 (6.3-10) | 432.3 (312-568.7) | 5.2 (3.8-6.9) | -0.79 (-1.83-0.26) |
| Leukemia | Hellenic Republic | 1026.8 (971.8-1072.7) | 7.2 (6.9-7.5) | 1574.7 (1424.1-1692.8) | 6.2 (5.8-6.7) | -0.69 (-1.52-0.14) |
| Leukemia | Hungary | 931.4 (872.8-993.5) | 6.8 (6.4-7.3) | 890.7 (755.6-1055.9) | 4.9 (4.1-5.8) | -0.82 (-1.6--0.03) |
| Leukemia | Independent State of Papua New Guinea | 103.4 (52.5-145.3) | 3.6 (1.9-5.5) | 243.5 (139.7-355.6) | 3.2 (1.8-5) | -0.21 (-0.95-0.53) |
| Leukemia | Independent State of Samoa | 6 (4.8-8) | 5.7 (4.4-7.4) | 8 (6-10.8) | 5.1 (3.8-6.9) | -0.24 (-1.16-0.68) |
| Leukemia | Ireland | 252.1 (237.7-264.7) | 6.2 (5.9-6.5) | 270.3 (239.3-297.6) | 3.5 (3.1-3.8) | -1.01 (-1.81--0.19) |
| Leukemia | Islamic Republic of Afghanistan | 860.2 (451.7-1325.1) | 10.8 (6-16.1) | 1717.6 (998.2-2482.6) | 10 (6-14.6) | -0.2 (-1.14-0.75) |
| Leukemia | Islamic Republic of Iran | 3041.1 (1894.9-3810.3) | 7.5 (4.7-9.3) | 3884.1 (2533.6-4488.3) | 5.1 (3.3-5.8) | -0.61 (-1.39-0.17) |
| Leukemia | Islamic Republic of Mauritania | 17.6 (13.5-22.4) | 1.1 (0.8-1.3) | 37.3 (19.2-55) | 1.2 (0.6-1.7) | 0.12 (-0.24-0.47) |
| Leukemia | Islamic Republic of Pakistan | 3194.2 (2277.5-4058.9) | 3.8 (2.8-4.7) | 6201.1 (4709.4-8410.2) | 3.6 (2.8-4.8) | -0.15 (-0.86-0.56) |
| Leukemia | Jamaica | 72.1 (67-77.9) | 3.4 (3.2-3.7) | 109.8 (85.4-139.8) | 3.6 (2.8-4.6) | 0.12 (-0.59-0.83) |
| Leukemia | Japan | 6377 (6136.6-6508.9) | 4.2 (4-4.3) | 10592.2 (9126.3-11420.5) | 3.1 (2.8-3.2) | -0.53 (-1.28-0.23) |
| Leukemia | Kingdom of Bahrain | 18.1 (13.5-21.5) | 8.4 (6.1-10.2) | 42.5 (30-57.2) | 5 (3.7-6.5) | -1.23 (-2.22--0.23) |
| Leukemia | Kingdom of Belgium | 989.8 (915.1-1062.9) | 6.8 (6.3-7.3) | 1181.5 (1016.1-1313.1) | 4.9 (4.4-5.4) | -0.78 (-1.6-0.05) |
| Leukemia | Kingdom of Bhutan | 15.1 (9.3-21.3) | 3.5 (2.3-4.8) | 18.4 (12.1-28.1) | 2.9 (1.9-4.3) | -0.34 (-0.99-0.32) |
| Leukemia | Kingdom of Cambodia | 540.4 (342-791.4) | 7.3 (5-9.9) | 816.6 (594.5-1080.1) | 5.9 (4.4-7.6) | -0.48 (-1.35-0.4) |
| Leukemia | Kingdom of Denmark | 582.2 (549-607.4) | 7.5 (7.1-7.8) | 645.9 (576.8-703.7) | 5.3 (4.8-5.8) | -0.74 (-1.6-0.13) |
| Leukemia | Kingdom of Eswatini | 14.3 (10.8-20.4) | 3.5 (2.7-4.7) | 27.9 (18.2-41.8) | 4 (2.7-5.9) | 0.34 (-0.51-1.21) |
| Leukemia | Kingdom of Lesotho | 26.2 (19.2-34.9) | 2.6 (1.9-3.5) | 54.1 (39-73.2) | 4.4 (3.2-5.8) | 0.94 (0.14-1.74) |
| Leukemia | Kingdom of Morocco | 249.4 (185.6-317.3) | 1.3 (1-1.7) | 385.5 (268.5-493.8) | 1.2 (0.8-1.5) | -0.08 (-0.46-0.29) |
| Leukemia | Kingdom of Norway | 313.4 (294.4-325.6) | 4.8 (4.6-5) | 413.2 (369.3-441.5) | 4 (3.7-4.3) | -0.33 (-1.13-0.48) |
| Leukemia | Kingdom of Saudi Arabia | 306.8 (213.4-494.6) | 3.2 (2.2-5.6) | 873 (618.9-1354) | 3.6 (2.7-5.3) | 0.26 (-0.34-0.87) |
| Leukemia | Kingdom of Spain | 2887.7 (2732.7-3015.4) | 5.8 (5.5-6.1) | 3855.6 (3311.6-4273.7) | 3.9 (3.4-4.2) | -0.8 (-1.53--0.07) |
| Leukemia | Kingdom of Sweden | 714 (669.7-752.6) | 5 (4.8-5.3) | 844.6 (731.2-947.2) | 3.7 (3.3-4.1) | -0.55 (-1.35-0.26) |
| Leukemia | Kingdom of Thailand | 2457.6 (1848.5-2907.4) | 5.8 (4.2-6.9) | 4522 (2598.3-6029.7) | 4.9 (2.9-6.4) | -0.59 (-1.45-0.27) |
| Leukemia | Kingdom of the Netherlands | 1072.8 (1003.2-1128.2) | 5.6 (5.2-5.9) | 1389.6 (1242.8-1508.6) | 4 (3.6-4.3) | -0.65 (-1.48-0.19) |
| Leukemia | Kingdom of Tonga | 2.1 (1.5-2.7) | 3.1 (2.2-4.2) | 2.6 (1.8-3.5) | 3 (2.1-3.9) | -0.13 (-0.88-0.62) |
| Leukemia | Kyrgyz Republic | 154 (138.5-168.5) | 3.8 (3.5-4.2) | 143.3 (118.7-170.3) | 2.4 (2-2.8) | -0.67 (-1.25--0.1) |
| Leukemia | Lao People's Democratic Republic | 230.7 (126-352.7) | 7.3 (4.5-10.5) | 292.7 (211.8-394.4) | 5.1 (3.7-6.7) | -0.77 (-1.63-0.1) |
| Leukemia | Lebanese Republic | 147.8 (101.4-219.5) | 6.5 (4.5-9.5) | 309.4 (239.6-388.6) | 5 (3.9-6.3) | -0.36 (-1.17-0.46) |
| Leukemia | Malaysia | 721 (563-838.2) | 5.5 (4.5-6.6) | 1333.8 (1106.7-1749.2) | 4.6 (3.8-6.1) | -0.31 (-1.14-0.53) |
| Leukemia | Mongolia | 59.2 (46.5-75.9) | 3.2 (2.5-4.1) | 71 (52.8-90) | 2.4 (1.8-3.1) | -0.43 (-1.11-0.26) |
| Leukemia | Montenegro | 26.2 (21.1-32) | 4.3 (3.5-5.2) | 36.4 (28.3-46.7) | 4 (3.1-5.2) | -0.12 (-0.87-0.64) |
| Leukemia | New Zealand | 233.9 (220.4-245.5) | 6.1 (5.8-6.4) | 362 (326.6-387.2) | 4.4 (4-4.6) | -0.58 (-1.44-0.28) |
| Leukemia | North Macedonia | 90.9 (75.7-114) | 5 (4.2-6.3) | 127 (92.7-160.2) | 4.3 (3.2-5.4) | -0.23 (-1.25-0.79) |
| Leukemia | Northern Mariana Islands | 1.2 (0.8-1.6) | 4.8 (3.2-6.3) | 1.6 (1.2-1.9) | 3.6 (2.7-4.2) | -0.52 (-1.28-0.24) |
| Leukemia | Palestine | 101.7 (78.1-133.6) | 8.5 (6.5-11.2) | 166.8 (135.5-214) | 5.7 (4.6-7.2) | -0.8 (-1.71-0.11) |
| Leukemia | People's Democratic Republic of Algeria | 578.1 (431.3-697.8) | 3.4 (2.5-4.1) | 873.3 (641.3-1150.9) | 2.6 (1.9-3.4) | -0.28 (-0.92-0.36) |
| Leukemia | People's Republic of Bangladesh | 3501.3 (2292.8-4923) | 4.1 (3-5.2) | 3923.7 (2684.1-5367.7) | 2.7 (1.9-3.7) | -0.59 (-1.24-0.06) |
| Leukemia | People's Republic of China | 67423.1 (52044.6-80042.9) | 6.5 (5-7.7) | 58903.5 (43626-74038.9) | 3.4 (2.5-4.3) | -1.08 (-1.87--0.29) |
| Leukemia | Plurinational State of Bolivia | 415.8 (287.6-572.1) | 8 (5.6-10.6) | 647.3 (445.6-868.7) | 6.5 (4.5-8.6) | -0.42 (-1.3-0.48) |
| Leukemia | Portuguese Republic | 718.2 (680.5-752.8) | 5.8 (5.5-6.1) | 1005 (879.2-1103.1) | 4.1 (3.6-4.4) | -0.79 (-1.59-0.01) |
| Leukemia | Principality of Andorra | 4.2 (2.8-6.2) | 7.6 (5.2-11.3) | 6.6 (4.3-9.1) | 4.5 (2.9-6.2) | -0.9 (-1.78--0.01) |
| Leukemia | Principality of Monaco | 5.6 (4.2-7.6) | 8.9 (6.7-11.9) | 8.9 (6.7-11) | 9.8 (7.3-12.1) | 0.1 (-0.95-1.17) |
| Leukemia | Puerto Rico | 199.1 (188.6-211) | 5.6 (5.3-5.9) | 265.5 (216.4-314) | 4.2 (3.4-4.9) | -0.7 (-1.46-0.06) |
| Leukemia | Republic of Albania | 101.4 (83.3-128.6) | 4.1 (3.4-5.3) | 115.4 (81.5-171.6) | 3 (2.1-4.3) | -0.39 (-1.07-0.3) |
| Leukemia | Republic of Angola | 163.5 (106.8-234.7) | 2.3 (1.6-3) | 396.8 (249.2-576.2) | 2.1 (1.2-3.2) | -0.09 (-0.64-0.47) |
| Leukemia | Republic of Armenia | 170.6 (162.6-178.3) | 5.5 (5.2-5.7) | 139 (121.7-160) | 3.6 (3.2-4.1) | -0.62 (-1.3-0.06) |
| Leukemia | Republic of Austria | 640.3 (602.3-672.6) | 5.7 (5.4-6) | 824.1 (724.1-900.1) | 4.4 (3.9-4.7) | -0.49 (-1.3-0.34) |
| Leukemia | Republic of Azerbaijan | 310.1 (256.8-379.8) | 4.6 (3.8-5.6) | 337.4 (237.5-498.6) | 3.3 (2.4-4.9) | -0.64 (-1.38-0.1) |
| Leukemia | Republic of Belarus | 716.3 (671.5-757.3) | 6 (5.6-6.4) | 643.2 (529.4-780.9) | 4.4 (3.6-5.3) | -0.69 (-1.4-0.03) |
| Leukemia | Republic of Benin | 50.1 (38-66.8) | 1.1 (0.9-1.4) | 136.6 (65.6-195.5) | 1.3 (0.6-1.8) | 0.2 (-0.18-0.58) |
| Leukemia | Republic of Botswana | 22.6 (15.9-31) | 3.1 (2.2-4.3) | 46.4 (31.4-66.6) | 2.9 (2-4.1) | -0.07 (-0.81-0.67) |
| Leukemia | Republic of Bulgaria | 485.7 (441.6-530.6) | 4.7 (4.3-5.1) | 583.5 (480-701.5) | 4.7 (3.8-5.6) | 0.05 (-0.67-0.78) |
| Leukemia | Republic of Burundi | 119.2 (87.9-161.1) | 2.7 (2.1-3.6) | 204.9 (114.5-319.6) | 2.5 (1.5-3.7) | -0.13 (-0.68-0.42) |
| Leukemia | Republic of Cabo Verde | 7.6 (5.9-10.6) | 2.3 (1.8-3.3) | 13.8 (10.4-17.4) | 2.9 (2.2-3.7) | 0.14 (-0.46-0.75) |
| Leukemia | Republic of Cameroon | 102.3 (76.3-135.1) | 1.2 (0.9-1.5) | 321.2 (153.6-464.3) | 1.4 (0.7-2.1) | 0.23 (-0.18-0.64) |
| Leukemia | Republic of Chad | 52.7 (38.8-71.6) | 0.9 (0.7-1.2) | 193.9 (101.4-285.2) | 1.4 (0.7-2) | 0.39 (0.02-0.77) |
| Leukemia | Republic of Chile | 526.8 (505-549.1) | 4.7 (4.5-4.9) | 819.5 (752.6-879.6) | 3.4 (3.2-3.7) | -0.41 (-1.09-0.27) |
| Leukemia | Republic of Colombia | 1361.6 (1289.1-1432.4) | 5.2 (5-5.5) | 2223.3 (1877.2-2614.6) | 4.3 (3.6-5) | -0.44 (-1.19-0.31) |
| Leukemia | Republic of Costa Rica | 123.5 (116.9-129.9) | 5.5 (5.1-5.8) | 290.7 (258.6-321.2) | 5.6 (5-6.2) | -0.11 (-0.88-0.68) |
| Leukemia | République de Côte d’Ivoire | 78 (53.6-100.7) | 0.9 (0.6-1.1) | 161.7 (91.1-243.2) | 0.8 (0.5-1.2) | -0.02 (-0.32-0.28) |
| Leukemia | Republic of Croatia | 324.3 (280.9-373.3) | 5.8 (5-6.7) | 416.1 (349.3-491.8) | 4.7 (4-5.6) | -0.54 (-1.3-0.23) |
| Leukemia | Republic of Cuba | 527.9 (503.7-547.9) | 5.1 (4.8-5.3) | 649.8 (561.5-735.8) | 3.8 (3.3-4.3) | -0.44 (-1.14-0.26) |
| Leukemia | Republic of Cyprus | 53.5 (42.9-74) | 7.9 (6.2-11.3) | 98.4 (69.8-125.1) | 5.1 (3.6-6.3) | -0.67 (-1.65-0.33) |
| Leukemia | Republic of Djibouti | 6.1 (4-9.1) | 2.2 (1.5-3.3) | 20.9 (11.9-33.8) | 2.6 (1.6-4) | 0.21 (-0.35-0.77) |
| Leukemia | Republic of Ecuador | 373.9 (353.6-396) | 4.8 (4.5-5.1) | 895.3 (732.7-1091.3) | 5.3 (4.3-6.4) | 0.33 (-0.52-1.18) |
| Leukemia | Republic of El Salvador | 235.1 (204-294.8) | 5 (4.4-6.4) | 313.7 (224.5-387.9) | 4.9 (3.5-6.1) | -0.02 (-0.83-0.79) |
| Leukemia | Republic of Equatorial Guinea | 6.8 (4.8-9.4) | 2.3 (1.6-2.9) | 16.2 (8.2-27.1) | 2 (1.1-3.2) | -0.19 (-0.72-0.35) |
| Leukemia | Republic of Estonia | 134.8 (125.9-143.3) | 7.2 (6.7-7.6) | 114.9 (97.6-131.8) | 4.5 (3.9-5.1) | -1 (-1.8--0.2) |
| Leukemia | Republic of Fiji | 29.2 (16.4-39) | 5.8 (3.2-7.7) | 38.9 (21.7-53.7) | 5.1 (2.8-7) | -0.32 (-1.45-0.82) |
| Leukemia | Republic of Finland | 326.4 (307-342.2) | 4.8 (4.5-5) | 429.5 (375.3-470.3) | 3.4 (3.1-3.7) | -0.61 (-1.32-0.11) |
| Leukemia | Republic of Ghana | 293.3 (151.6-402.4) | 2.3 (1.3-3.1) | 348.8 (237.5-509.1) | 1.3 (1-1.9) | -0.75 (-1.24--0.27) |
| Leukemia | Republic of Guatemala | 264.9 (248.7-279.9) | 3.8 (3.6-3.9) | 566.9 (490.9-654.2) | 4.1 (3.6-4.7) | -0.01 (-0.78-0.77) |
| Leukemia | Republic of Guinea | 30.3 (20.7-40.2) | 0.5 (0.3-0.6) | 46 (24.4-78) | 0.4 (0.2-0.6) | -0.02 (-0.21-0.16) |
| Leukemia | Republic of Guinea-Bissau | 11.9 (8.1-17.2) | 1.4 (1-1.9) | 22.1 (12.3-29.7) | 1.6 (0.9-2.2) | 0.24 (-0.2-0.68) |
| Leukemia | Republic of Guyana | 19.6 (17.1-21.9) | 3.5 (3.1-3.9) | 22.5 (17.2-28.8) | 3.3 (2.5-4.1) | 0.13 (-0.52-0.78) |
| Leukemia | Republic of Haiti | 399 (208.5-625.8) | 7.5 (4.7-10.6) | 588 (365.5-873.5) | 5.9 (3.9-8.4) | -0.34 (-1.08-0.41) |
| Leukemia | Republic of Honduras | 221 (181.1-270.6) | 5.5 (4.4-6.7) | 372.7 (263.2-511.4) | 4.9 (3.6-6.5) | -0.13 (-0.92-0.67) |
| Leukemia | Republic of Iceland | 13.8 (12.5-14.9) | 4.8 (4.4-5.2) | 22.6 (19.7-25.3) | 3.9 (3.5-4.4) | -0.35 (-1.12-0.43) |
| Leukemia | Republic of India | 20556.1 (16329.4-25021.5) | 3.2 (2.6-3.8) | 32145 (26215-39098.3) | 2.6 (2.1-3.2) | -0.31 (-0.92-0.29) |
| Leukemia | Republic of Indonesia | 7086.4 (5312.3-9197.6) | 5 (3.8-6.4) | 11340 (9099.9-14616.1) | 4.7 (3.8-6.1) | -0.16 (-0.99-0.69) |
| Leukemia | Republic of Iraq | 855.8 (663.5-1196.2) | 6.8 (5.4-9.5) | 1602.7 (1221.8-2073.4) | 5.8 (4.4-7.5) | -0.36 (-1.22-0.5) |
| Leukemia | Republic of Italy | 5401.6 (5098.5-5584.3) | 6.7 (6.4-6.9) | 7140.7 (6248.9-7721) | 4.8 (4.3-5.1) | -0.84 (-1.62--0.04) |
| Leukemia | Republic of Kazakhstan | 639.8 (600.7-677.9) | 4.3 (4-4.6) | 498.3 (442-563.9) | 2.7 (2.4-3.1) | -0.54 (-1.21-0.12) |
| Leukemia | Republic of Kenya | 262 (194.6-345.5) | 1.8 (1.3-2.7) | 629.6 (449-853.2) | 2.1 (1.6-2.8) | 0.29 (-0.18-0.76) |
| Leukemia | Republic of Kiribati | 1.8 (1.2-2.2) | 3.2 (2.1-3.9) | 2.9 (1.7-4.1) | 3.1 (1.9-4.2) | -0.04 (-0.76-0.68) |
| Leukemia | Republic of Korea | 1753.7 (1442.7-2161.9) | 4.7 (4-6) | 1949.5 (1211-2389.6) | 2.4 (1.5-2.9) | -1 (-1.72--0.27) |
| Leukemia | Republic of Latvia | 224.1 (209.4-238.1) | 6.9 (6.4-7.3) | 166.1 (141.4-190.9) | 4.6 (3.9-5.2) | -0.81 (-1.58--0.03) |
| Leukemia | Republic of Liberia | 26 (17.7-37.9) | 1.1 (0.9-1.4) | 50.7 (23.4-75) | 1.3 (0.6-1.9) | 0.23 (-0.14-0.6) |
| Leukemia | Republic of Lithuania | 279.8 (264.5-294.9) | 6.6 (6.3-7) | 270.2 (233.9-303.1) | 5.1 (4.4-5.7) | -0.54 (-1.31-0.25) |
| Leukemia | Republic of Madagascar | 197.4 (153.7-257.1) | 2.1 (1.6-2.7) | 382.7 (252-525.5) | 2 (1.3-2.8) | 0.01 (-0.48-0.5) |
| Leukemia | Republic of Malawi | 80.1 (53-103.9) | 0.9 (0.6-1.1) | 101.6 (54.8-168.3) | 0.8 (0.5-1.1) | -0.09 (-0.38-0.2) |
| Leukemia | Republic of Maldives | 7.6 (4.3-11.7) | 5.1 (3.2-7.3) | 9.2 (6.6-12.4) | 2.4 (1.8-3.2) | -1.16 (-1.89--0.42) |
| Leukemia | Republic of Mali | 83.3 (57-109.8) | 1 (0.7-1.2) | 147.1 (88.2-233.8) | 0.8 (0.5-1.1) | -0.11 (-0.42-0.2) |
| Leukemia | Republic of Malta | 21.3 (19.6-23) | 5.2 (4.8-5.6) | 35 (30.3-39.6) | 3.9 (3.4-4.3) | -0.66 (-1.46-0.15) |
| Leukemia | Republic of Mauritius | 35.7 (34.2-37.1) | 4.2 (4-4.4) | 52.6 (48.7-55.4) | 3.2 (3-3.4) | -0.51 (-1.24-0.22) |
| Leukemia | Republic of Moldova | 222.1 (212.9-232.6) | 5.1 (4.8-5.3) | 146 (132.6-163.5) | 2.8 (2.5-3.1) | -0.74 (-1.42--0.06) |
| Leukemia | Republic of Mozambique | 456.3 (340.8-601) | 3.9 (3.1-4.8) | 731.6 (456.9-1137.3) | 3.7 (2.7-5.1) | 0.14 (-0.52-0.8) |
| Leukemia | Republic of Namibia | 19.5 (14.7-24.5) | 2.3 (1.8-2.9) | 37.3 (26.2-52.3) | 2.3 (1.6-3.1) | -0.03 (-0.65-0.6) |
| Leukemia | Republic of Nauru | 0.4 (0.3-0.5) | 5.6 (4.1-7.5) | 0.4 (0.3-0.6) | 5.2 (3.4-7.4) | -0.22 (-1.15-0.73) |
| Leukemia | Republic of Nicaragua | 146.5 (123.3-184) | 4.1 (3.6-5.2) | 187.8 (134.3-230.6) | 3.3 (2.4-4) | -0.21 (-0.94-0.53) |
| Leukemia | Republic of Niue | 0.1 (0.1-0.1) | 3.9 (2.7-5) | 0.1 (0.1-0.1) | 5.2 (3.8-6.3) | -0.06 (-0.86-0.74) |
| Leukemia | Republic of Palau | 0.3 (0.2-0.4) | 2.7 (1.9-3.6) | 0.4 (0.3-0.5) | 2.3 (1.8-2.9) | -0.16 (-0.66-0.36) |
| Leukemia | Republic of Panama | 83.7 (78.4-89.5) | 4.1 (3.8-4.4) | 191.8 (156.6-227.9) | 4.4 (3.6-5.2) | 0.06 (-0.7-0.83) |
| Leukemia | Republic of Paraguay | 134.5 (106.9-161.2) | 4.3 (3.4-5.2) | 284.4 (208.8-361.2) | 4.6 (3.4-5.9) | 0.24 (-0.47-0.96) |
| Leukemia | Republic of Peru | 912 (751.5-1178.8) | 5.1 (4.2-6.5) | 1552.6 (989.9-2056.7) | 4.5 (2.9-5.9) | -0.18 (-0.97-0.62) |
| Leukemia | Republic of Poland | 2807.8 (2615.5-3011.7) | 6.7 (6.2-7.2) | 3310.1 (2997.6-3603.2) | 4.7 (4.3-5.1) | -0.79 (-1.55--0.02) |
| Leukemia | Republic of Rwanda | 171.8 (126.2-229.9) | 3.2 (2.5-4.2) | 225.7 (139-345) | 2.6 (1.7-3.8) | -0.5 (-1.08-0.08) |
| Leukemia | Republic of San Marino | 3 (2.4-4) | 8.9 (7.2-11.9) | 3.3 (2.2-4.6) | 4.5 (2.9-6.3) | -0.78 (-1.6-0.04) |
| Leukemia | Republic of Senegal | 77.4 (59.3-98.5) | 1.1 (0.8-1.4) | 154.1 (76.6-235.8) | 1.3 (0.7-1.9) | 0.23 (-0.16-0.61) |
| Leukemia | Republic of Serbia | 521.8 (423.6-619.6) | 5.3 (4.4-6.4) | 666.5 (505-821.4) | 4.2 (3.2-5.1) | -0.51 (-1.26-0.25) |
| Leukemia | Republic of Seychelles | 4.1 (3.4-5) | 6.5 (5.6-8.2) | 5.2 (4.2-6.3) | 4.7 (3.8-5.7) | -0.43 (-1.15-0.29) |
| Leukemia | Republic of Sierra Leone | 42.6 (30.8-60.3) | 1 (0.8-1.3) | 88.4 (42.5-127.2) | 1.3 (0.6-1.8) | 0.26 (-0.11-0.64) |
| Leukemia | Republic of Singapore | 113.3 (106.8-119.1) | 4.6 (4.3-4.8) | 187.5 (171.9-201.7) | 2.5 (2.3-2.7) | -0.85 (-1.66--0.04) |
| Leukemia | Republic of Slovenia | 126.4 (117.3-135.9) | 5.4 (5-5.7) | 192.8 (163.2-222.7) | 4.3 (3.6-4.9) | -0.5 (-1.23-0.23) |
| Leukemia | Republic of South Africa | 818.4 (611-976.3) | 3.2 (2.2-4) | 1576 (1150.8-1819.1) | 3.4 (2.4-3.9) | 0.09 (-0.68-0.86) |
| Leukemia | Republic of South Sudan | 111 (76.7-163.5) | 2.5 (1.8-3.5) | 201.4 (132.9-280.1) | 3 (2-4.2) | 0.3 (-0.25-0.86) |
| Leukemia | Republic of Sudan | 1057.9 (628.4-1577.9) | 6.9 (4.5-9.5) | 1679.1 (1020.2-2396.2) | 5.8 (3.6-8.2) | -0.33 (-1.16-0.51) |
| Leukemia | Republic of Suriname | 12 (9-14.1) | 3.8 (2.9-4.5) | 19.8 (14.8-25.8) | 3.3 (2.5-4.3) | -0.16 (-0.77-0.45) |
| Leukemia | Republic of Tajikistan | 187.8 (141.4-230.1) | 3.9 (2.9-4.7) | 223.7 (145.3-339) | 2.5 (1.7-3.6) | -0.75 (-1.39--0.1) |
| Leukemia | Republic of the Congo | 37.5 (28-50.3) | 2.4 (1.9-3.1) | 76.1 (48.7-107.5) | 2.2 (1.4-3.1) | -0.14 (-0.73-0.45) |
| Leukemia | Republic of the Gambia | 4.6 (3.1-6.2) | 0.6 (0.4-0.7) | 9.7 (6.5-13.4) | 0.6 (0.4-0.8) | -0.02 (-0.24-0.2) |
| Leukemia | Republic of the Marshall Islands | 1.1 (0.8-1.4) | 4.2 (3-5.4) | 1.7 (1.1-2.5) | 4.2 (2.8-5.8) | -0.05 (-0.88-0.79) |
| Leukemia | Republic of the Niger | 91.2 (59.6-128.3) | 1.1 (0.9-1.5) | 227.2 (102.4-359.2) | 1.2 (0.6-1.9) | 0.12 (-0.25-0.5) |
| Leukemia | Republic of the Philippines | 2451 (2015.8-2967.6) | 5.2 (4.1-6.1) | 3991.1 (3364.2-4758.4) | 4.2 (3.5-5) | -0.35 (-1.19-0.49) |
| Leukemia | Republic of the Union of Myanmar | 2401.8 (1405.6-3556.1) | 7.4 (4.8-10.4) | 2473.3 (1854.2-3257.9) | 4.9 (3.7-6.4) | -0.95 (-1.84--0.07) |
| Leukemia | Republic of Trinidad and Tobago | 47.3 (45-49.5) | 4.8 (4.6-5.1) | 66.6 (51.4-84.1) | 3.9 (3-4.9) | -0.43 (-1.12-0.27) |
| Leukemia | Republic of Tunisia | 219.7 (168.9-272.5) | 3.6 (2.7-4.5) | 328 (229.9-452.7) | 2.6 (1.9-3.6) | -0.48 (-1.11-0.16) |
| Leukemia | Republic of Turkey | 3997.5 (2996.3-4900.6) | 9.1 (6.9-11) | 4803.5 (3623.6-5951.1) | 5.5 (4.1-6.7) | -1.01 (-1.89--0.12) |
| Leukemia | Republic of Uganda | 233.9 (165.2-303.9) | 1.8 (1.3-2.3) | 534.3 (310.1-797.6) | 1.9 (1.2-2.6) | -0.04 (-0.5-0.43) |
| Leukemia | Republic of Uzbekistan | 786.5 (713.2-866.1) | 4.4 (4-4.9) | 898.4 (741.2-1092.8) | 2.8 (2.3-3.4) | -0.68 (-1.33--0.03) |
| Leukemia | Republic of Vanuatu | 3.3 (2-4.6) | 3.7 (2.2-5.2) | 7.8 (5.1-10.2) | 3.6 (2.4-4.7) | -0.13 (-0.91-0.65) |
| Leukemia | Republic of Yemen | 557.4 (334.4-887.5) | 6.5 (4-9.6) | 1130.5 (624.4-1649.8) | 5.8 (3.2-8.4) | -0.28 (-1.07-0.51) |
| Leukemia | Republic of Zambia | 163.4 (123.7-219.9) | 2.7 (2.2-3.5) | 318.9 (197.9-459.5) | 2.6 (1.7-3.6) | -0.05 (-0.63-0.53) |
| Leukemia | Republic of Zimbabwe | 169.4 (123.5-209.7) | 3.1 (2.4-4) | 402.9 (286.3-532.5) | 4.2 (3.1-5.5) | 0.62 (-0.21-1.45) |
| Leukemia | Romania | 1054.2 (1006.5-1103.2) | 4.2 (4-4.4) | 1371.3 (1194.3-1541.3) | 4 (3.5-4.6) | -0.11 (-0.79-0.57) |
| Leukemia | Russian Federation | 7568.5 (7418.3-7726.9) | 4.5 (4.5-4.6) | 8069.5 (7475.8-8676.4) | 3.7 (3.4-4) | -0.44 (-1.09-0.21) |
| Leukemia | Saint Kitts and Nevis | 2 (1.9-2.2) | 5.2 (4.9-5.5) | 2.1 (1.7-2.4) | 3.5 (3-4) | -0.42 (-1.11-0.28) |
| Leukemia | Saint Lucia | 5.2 (4.9-5.5) | 5.1 (4.8-5.4) | 7.4 (6.1-8.9) | 3.5 (2.9-4.2) | -0.73 (-1.34--0.11) |
| Leukemia | Saint Vincent and the Grenadines | 4.6 (4.3-5) | 5.3 (4.9-5.7) | 5.6 (4.9-6.3) | 4.4 (3.8-5) | -0.29 (-0.95-0.37) |
| Leukemia | Slovak Republic | 341.3 (289.3-420.7) | 5.9 (5-7.2) | 390.8 (277.3-501.1) | 4.4 (3.2-5.6) | -0.52 (-1.32-0.29) |
| Leukemia | Socialist Republic of Viet Nam | 1680 (1317.2-2165.3) | 3.3 (2.6-4.3) | 2493.4 (1878-3421.4) | 2.6 (1.9-3.5) | -0.39 (-1.08-0.3) |
| Leukemia | Solomon Islands | 8.7 (4.4-13) | 4.3 (2.2-6.3) | 19.8 (12.1-27) | 4.2 (2.6-5.7) | -0.08 (-0.9-0.74) |
| Leukemia | State of Eritrea | 58.4 (41.4-84.4) | 2.5 (1.9-3.4) | 119.7 (73.8-170.9) | 2.8 (1.8-3.8) | 0.18 (-0.38-0.75) |
| Leukemia | State of Israel | 352.3 (329.2-372.9) | 7.3 (6.8-7.8) | 666.2 (587.7-728.8) | 5.2 (4.7-5.7) | -0.73 (-1.7-0.25) |
| Leukemia | State of Kuwait | 43.9 (40.6-47.8) | 5 (4.6-5.5) | 82.5 (68.8-98.3) | 2.6 (2.2-3.1) | -1.06 (-1.75--0.36) |
| Leukemia | State of Libya | 178.5 (137.1-224.4) | 6.7 (4.8-8.7) | 360.4 (264-484.3) | 6.6 (4.8-8.6) | 0.11 (-0.77-0.99) |
| Leukemia | State of Qatar | 12.3 (8.2-15.7) | 7.8 (5.5-9.9) | 45.2 (30-62.9) | 4.1 (2.8-5.5) | -1.42 (-2.21--0.63) |
| Leukemia | Sultanate of Oman | 45.5 (31.9-61.9) | 4.4 (3.1-6) | 69.7 (52.3-90.6) | 3 (2.3-3.9) | -0.44 (-1.2-0.33) |
| Leukemia | Swiss Confederation | 579.9 (545-611) | 5.9 (5.6-6.2) | 716.1 (620.1-807.6) | 3.8 (3.4-4.3) | -0.68 (-1.44-0.08) |
| Leukemia | Syrian Arab Republic | 700.5 (528.8-874.7) | 8.6 (6.3-10.7) | 787.3 (572.2-1086.9) | 6.3 (4.6-8.6) | -0.8 (-1.65-0.06) |
| Leukemia | Taiwan (Province of China) | 490.5 (468.3-511) | 2.8 (2.7-2.9) | 1197.2 (1090.9-1290.6) | 3.3 (3-3.5) | 0.19 (-0.59-0.99) |
| Leukemia | Togolese Republic | 30.2 (22.5-38.6) | 1 (0.8-1.3) | 78 (36.7-113.8) | 1.3 (0.7-1.9) | 0.28 (-0.09-0.66) |
| Leukemia | Tokelau | 0.1 (0-0.1) | 4.2 (2.8-5.6) | 0.1 (0.1-0.1) | 5.7 (3.6-8.1) | -0.17 (-0.97-0.64) |
| Leukemia | Turkmenistan | 115.1 (106.2-124.1) | 3.6 (3.3-3.8) | 134.3 (106.5-168.9) | 2.8 (2.2-3.5) | -0.41 (-1.04-0.21) |
| Leukemia | Tuvalu | 0.4 (0.3-0.5) | 4.7 (3.4-6.3) | 0.4 (0.3-0.5) | 3.8 (2.6-4.8) | -0.34 (-1.16-0.48) |
| Leukemia | Ukraine | 3794.9 (3610.6-3975.8) | 6.2 (5.9-6.5) | 2343.9 (1752.9-3021.5) | 3.5 (2.7-4.5) | -1.13 (-1.8--0.46) |
| Leukemia | Union of the Comoros | 8.7 (5.9-11.9) | 2.5 (1.8-3.4) | 15.1 (10.1-21.3) | 2.6 (1.8-3.7) | 0.02 (-0.53-0.57) |
| Leukemia | United Arab Emirates | 61.3 (43.5-81.5) | 8.6 (5.8-11.9) | 224.9 (155.6-303.4) | 5.7 (4.3-7.4) | -0.09 (-0.97-0.79) |
| Leukemia | United Kingdom of Great Britain and Northern Ireland | 4340.1 (4142.2-4437.4) | 5.1 (4.9-5.2) | 5731.6 (5177.2-6014.6) | 4.3 (4-4.5) | -0.46 (-1.32-0.41) |
| Leukemia | United Mexican States | 3488.3 (3374.6-3615.6) | 4.9 (4.8-5) | 5764.7 (5196.3-6333.2) | 4.5 (4.1-5) | -0.21 (-1.03-0.62) |
| Leukemia | United Republic of Tanzania | 536.6 (425.1-669.8) | 2.6 (2.1-3.3) | 1033.6 (650.1-1513.9) | 2.5 (1.6-3.6) | -0.01 (-0.56-0.55) |
| Leukemia | United States of America | 21859.1 (20543-22559.2) | 7 (6.6-7.2) | 29786 (26704-31337.3) | 5.2 (4.7-5.4) | -0.7 (-1.61-0.21) |
| Leukemia | United States Virgin Islands | 3.8 (3-4.7) | 4.2 (3.3-5.2) | 3.6 (2.6-4.8) | 2.6 (1.9-3.4) | -0.54 (-1.26-0.18) |
| Leukemia | African Union | 14168.71 (11060.17-17791.95) | 3.06 (2.52-3.77) | 25621.52 (17592.9-31224.74) | 2.86 (2.02-3.44) | -0.09 (-0.15--0.03) |
| Leukemia | Association of Southeast Asian Nations | 17692.14 (13984.15-22119.79) | 5.19 (4.22-6.34) | 27466.41 (22423-32766.89) | 4.36 (3.6-5.21) | -0.65 (-0.73--0.56) |
| Leukemia | Central Europe, Eastern Europe, and Central Asia | 23652.88 (23085.02-24228.76) | 5.27 (5.15-5.41) | 23972.62 (22427.78-25365.78) | 4.04 (3.77-4.28) | -0.92 (-1--0.83) |
| Leukemia | Commonwealth | 41183.84 (34144.53-48123.15) | 3.81 (3.26-4.33) | 64987.46 (53697.99-75391.03) | 3.07 (2.57-3.56) | -0.74 (-0.79--0.69) |
| Leukemia | European Union | 34779.57 (33321.28-35922.42) | 6.23 (5.99-6.42) | 45162.18 (40467.17-48242.57) | 4.62 (4.23-4.92) | -0.91 (-0.94--0.88) |
| Leukemia | Four World Regions | 247632.19 (217333.77-276863.23) | 5.56 (4.97-6.13) | 319663.13 (274415.63-348409.55) | 3.89 (3.34-4.25) | -1.17 (-1.21--1.14) |
| Leukemia | G20 | 190616.9 (169611.59-209140.77) | 5.93 (5.33-6.43) | 233731.7 (207140.39-256644.65) | 3.91 (3.44-4.29) | -1.39 (-1.43--1.35) |
| Leukemia | Gulf Cooperation Council | 487.84 (374.89-705.89) | 3.87 (2.94-5.87) | 1337.79 (1020.72-1896) | 3.62 (2.85-4.87) | 0.01 (-0.3-0.32) |
| Leukemia | Health System Grouping Levels | 247844.5 (217541.55-277074.27) | 5.56 (4.97-6.13) | 319944.5 (274670.02-348692.18) | 3.89 (3.34-4.25) | -1.17 (-1.21--1.14) |
| Leukemia | High SDI | 63305.81 (60254.11-65087.74) | 6.01 (5.73-6.18) | 88885.31 (80251.52-94208.1) | 4.28 (3.94-4.52) | -1.08 (-1.11--1.04) |
| Leukemia | High-income | 68994.59 (65710.92-70944.55) | 6.09 (5.81-6.26) | 95837.7 (85703.38-101665.3) | 4.36 (3.99-4.57) | -1.07 (-1.1--1.03) |
| Leukemia | High-middle SDI | 62571.14 (54728.7-67978.19) | 6.28 (5.51-6.83) | 68974.73 (58980.33-76585.23) | 3.98 (3.36-4.43) | -1.55 (-1.62--1.48) |
| Leukemia | Latin America and Caribbean | 15220.77 (14606.72-16042.09) | 4.88 (4.67-5.09) | 25841.62 (23753.23-28078.61) | 4.3 (3.95-4.68) | -0.36 (-0.42--0.3) |
| Leukemia | Low SDI | 13650.27 (9554.94-18484.26) | 3.64 (2.78-4.56) | 22229.48 (15775.48-27620.98) | 3 (2.16-3.76) | -0.71 (-0.77--0.65) |
| Leukemia | Low-middle SDI | 33315.58 (26492.53-41633.62) | 3.69 (3.07-4.48) | 50958.87 (42774.29-60746.74) | 3.22 (2.7-3.87) | -0.43 (-0.47--0.39) |
| Leukemia | Middle SDI | 75001.71 (62080.32-87496.67) | 5.25 (4.39-6.04) | 88896.12 (72770.98-101476.56) | 3.52 (2.87-4) | -1.34 (-1.37--1.3) |
| Leukemia | Nordic Region | 1951.34 (1849.94-2023.09) | 5.5 (5.26-5.68) | 2357.33 (2112.88-2532.85) | 4.02 (3.67-4.28) | -1.02 (-1.12--0.92) |
| Leukemia | North Africa and Middle East | 15093.81 (11718.34-18439.57) | 6.31 (4.95-7.59) | 24320.95 (18080.41-28697.14) | 5.03 (3.76-5.9) | -0.54 (-0.63--0.46) |
| Leukemia | OECD Countries | 81339.56 (77514.03-83626.07) | 6.37 (6.08-6.56) | 112280.77 (101502.54-118585.45) | 4.61 (4.25-4.83) | -1.04 (-1.08--1.01) |
| Leukemia | Organization of Islamic Cooperation | 34486.96 (27717.61-41989.57) | 4.44 (3.6-5.31) | 55017.28 (43359.91-63879.55) | 3.75 (2.99-4.42) | -0.46 (-0.49--0.42) |
| Leukemia | Sahel Region | 2696.25 (1866.51-3734.53) | 2.25 (1.65-2.89) | 5486.11 (3350.6-6873.76) | 2.08 (1.3-2.59) | -0.19 (-0.23--0.14) |
| Leukemia | South Asia | 27796.3 (21712.15-34052.31) | 3.37 (2.7-4.03) | 43018.53 (35242.9-52979.32) | 2.76 (2.26-3.42) | -0.73 (-0.8--0.67) |
| Leukemia | Southeast Asia, East Asia, and Oceania | 87380.9 (68932.11-104040.57) | 5.97 (4.79-7.08) | 89846.43 (70014.88-107788.38) | 3.63 (2.8-4.32) | -1.71 (-1.77--1.65) |
| Leukemia | Sub-Saharan Africa | 9965.99 (7539.04-12976.63) | 2.73 (2.14-3.3) | 17445.78 (11732.03-22308.35) | 2.4 (1.65-3.07) | -0.43 (-0.46--0.41) |
| Leukemia | WHO region | 247140.14 (216846.39-276355.64) | 5.57 (4.98-6.14) | 318464.29 (273206.18-347227.73) | 3.9 (3.34-4.25) | -1.18 (-1.21--1.14) |
| Leukemia | World Bank Income Levels | 247844.11 (217541.23-277073.86) | 5.56 (4.97-6.13) | 319944.08 (274669.73-348691.74) | 3.89 (3.34-4.25) | -1.17 (-1.21--1.14) |
| Leukemia | World Bank Regions | 247753.21 (217455.59-276967.61) | 5.56 (4.97-6.13) | 319817.13 (274575.04-348545.84) | 3.89 (3.34-4.25) | -1.17 (-1.21--1.14) |
| Acute lymphoid leukemia | American Samoa | 0.1 (0-0.1) | 0.2 (0.1-0.4) | 0.1 (0-0.1) | 0.2 (0.1-0.3) | 0.18 (-0.62-1) |
| Acute lymphoid leukemia | Antigua and Barbuda | 0.4 (0.4-0.5) | 0.7 (0.6-0.7) | 0.4 (0.4-0.5) | 0.5 (0.5-0.6) | -0.2 (-0.97-0.58) |
| Acute lymphoid leukemia | Arab Republic of Egypt | 711.5 (458.7-994.6) | 1.3 (0.8-1.7) | 1161.6 (469.6-1640.5) | 1.3 (0.5-1.8) | 0.92 (0.11-1.74) |
| Acute lymphoid leukemia | Argentine Republic | 446.4 (405.6-487.3) | 1.3 (1.2-1.5) | 477.4 (437.2-520.3) | 1 (0.9-1.1) | -0.52 (-1.27-0.24) |
| Acute lymphoid leukemia | Australia | 149 (140.6-158.3) | 0.9 (0.8-0.9) | 126.1 (112.7-139.5) | 0.4 (0.3-0.4) | -0.68 (-1.46-0.1) |
| Acute lymphoid leukemia | Barbados | 1.3 (1.2-1.5) | 0.5 (0.5-0.6) | 1 (0.8-1.2) | 0.3 (0.2-0.4) | -0.03 (-0.83-0.77) |
| Acute lymphoid leukemia | Belize | 2.3 (2.1-2.6) | 1 (0.9-1.1) | 2.8 (2.5-3.2) | 0.7 (0.6-0.8) | -0.03 (-0.62-0.57) |
| Acute lymphoid leukemia | Bermuda | 0.2 (0.1-0.3) | 0.4 (0.3-0.5) | 0.1 (0.1-0.2) | 0.2 (0.1-0.2) | -0.87 (-1.63--0.11) |
| Acute lymphoid leukemia | Bolivarian Republic of Venezuela | 307.1 (289.6-323.3) | 1.5 (1.4-1.6) | 399.1 (294.5-525.3) | 1.5 (1.1-2) | -0.22 (-0.94-0.51) |
| Acute lymphoid leukemia | Bosnia and Herzegovina | 35.3 (23.4-48) | 0.8 (0.6-1.1) | 22.3 (13.1-36.2) | 0.5 (0.3-0.8) | -0.03 (-0.73-0.67) |
| Acute lymphoid leukemia | Brunei Darussalam | 2.2 (1.5-3.3) | 1 (0.6-1.4) | 2.3 (1.3-3) | 0.5 (0.3-0.7) | -0.39 (-1.21-0.45) |
| Acute lymphoid leukemia | Burkina Faso | 55.5 (30-87.3) | 0.4 (0.3-0.6) | 132.7 (49.6-204.4) | 0.5 (0.2-0.7) | 0.26 (-0.12-0.65) |
| Acute lymphoid leukemia | Canada | 229.7 (218.2-242.8) | 0.8 (0.8-0.9) | 207.3 (187.1-229) | 0.4 (0.4-0.4) | -0.6 (-1.44-0.24) |
| Acute lymphoid leukemia | Central African Republic | 23.4 (12.6-40.6) | 0.8 (0.5-1.3) | 39.4 (20.2-64.3) | 0.7 (0.4-1.1) | -0.06 (-0.65-0.54) |
| Acute lymphoid leukemia | Commonwealth of Dominica | 1.2 (0.9-1.5) | 1.6 (1.2-2.2) | 1.3 (0.9-1.8) | 1.9 (1.3-2.6) | 0.07 (-0.69-0.83) |
| Acute lymphoid leukemia | Commonwealth of the Bahamas | 1.5 (1.3-1.7) | 0.6 (0.5-0.6) | 1.8 (1.4-2.2) | 0.5 (0.4-0.6) | -0.14 (-0.76-0.5) |
| Acute lymphoid leukemia | Cook Islands | 0 (0-0) | 0.1 (0.1-0.2) | 0 (0-0) | 0.1 (0-0.1) | -0.42 (-0.88-0.03) |
| Acute lymphoid leukemia | Czech Republic | 194.8 (172.1-219.1) | 1.7 (1.5-1.8) | 82.6 (67-101.9) | 0.5 (0.4-0.6) | -0.7 (-1.46-0.08) |
| Acute lymphoid leukemia | Democratic People's Republic of Korea | 394 (213.6-558.2) | 2 (1.1-2.8) | 465.4 (238-688.4) | 1.7 (0.9-2.4) | -0.24 (-1.03-0.56) |
| Acute lymphoid leukemia | Democratic Republic of Sao Tome and Principe | 0.5 (0.3-0.7) | 0.3 (0.2-0.4) | 0.4 (0.2-0.8) | 0.2 (0.1-0.3) | -0.1 (-0.38-0.18) |
| Acute lymphoid leukemia | Democratic Republic of the Congo | 255.7 (151.2-424.1) | 0.6 (0.4-0.9) | 457.8 (245.9-678.2) | 0.6 (0.3-0.9) | 0.02 (-0.51-0.54) |
| Acute lymphoid leukemia | Democratic Republic of Timor-Leste | 14.9 (6.8-26.5) | 1.8 (1-2.9) | 18 (10.7-25.2) | 1.3 (0.8-1.8) | -0.3 (-1.08-0.49) |
| Acute lymphoid leukemia | Democratic Socialist Republic of Sri Lanka | 255.6 (170.4-365.3) | 1.6 (1.1-2.4) | 195 (110.5-304.4) | 0.8 (0.5-1.3) | -0.77 (-1.55-0.01) |
| Acute lymphoid leukemia | Dominican Republic | 90.8 (63.7-124.8) | 1.1 (0.8-1.5) | 80.1 (49.9-113.7) | 0.8 (0.5-1.1) | -0.06 (-0.7-0.57) |
| Acute lymphoid leukemia | Eastern Republic of Uruguay | 29.1 (26.6-31.5) | 0.9 (0.8-1) | 30.5 (27.3-33.8) | 0.8 (0.7-0.9) | -0.36 (-1.13-0.42) |
| Acute lymphoid leukemia | Federal Democratic Republic of Ethiopia | 1825.2 (717.2-3388.4) | 3.5 (1.8-6.2) | 2109.3 (1378.4-2994) | 2 (1.3-2.8) | -1.11 (-2.01--0.19) |
| Acute lymphoid leukemia | Federal Democratic Republic of Nepal | 207.8 (111.8-360.3) | 1 (0.6-1.6) | 190.5 (126-276.7) | 0.7 (0.4-0.9) | -0.23 (-0.85-0.4) |
| Acute lymphoid leukemia | Federal Republic of Germany | 671.8 (606.2-727.9) | 0.8 (0.7-0.8) | 505.2 (453.8-556) | 0.4 (0.4-0.4) | -0.55 (-1.36-0.27) |
| Acute lymphoid leukemia | Federal Republic of Nigeria | 533.6 (271.1-886.5) | 0.4 (0.2-0.7) | 1151.9 (469.3-1638.4) | 0.4 (0.2-0.5) | -0.02 (-0.38-0.34) |
| Acute lymphoid leukemia | Federal Republic of Somalia | 74.1 (41.1-128.6) | 0.8 (0.5-1.3) | 172.5 (98.6-272.9) | 0.8 (0.5-1.3) | 0.19 (-0.39-0.77) |
| Acute lymphoid leukemia | Federated States of Micronesia | 0.4 (0.3-0.7) | 0.5 (0.3-0.8) | 0.3 (0.2-0.5) | 0.4 (0.2-0.6) | -0.28 (-1.15-0.59) |
| Acute lymphoid leukemia | Federative Republic of Brazil | 1481.1 (1372.2-1584.1) | 1 (0.9-1.1) | 1683 (1587.1-1775.5) | 0.8 (0.7-0.8) | -0.3 (-1.02-0.43) |
| Acute lymphoid leukemia | French Republic | 535.4 (503.7-564.1) | 0.9 (0.8-0.9) | 421.5 (371.2-473.8) | 0.4 (0.4-0.5) | -0.61 (-1.42-0.2) |
| Acute lymphoid leukemia | Gabonese Republic | 5.3 (3.2-7.5) | 0.6 (0.3-0.7) | 8.4 (4.4-13.1) | 0.5 (0.3-0.8) | -0.02 (-0.6-0.57) |
| Acute lymphoid leukemia | Georgia | 70 (60.7-79.4) | 1.3 (1.1-1.5) | 24 (21.1-27.3) | 0.6 (0.5-0.7) | -0.01 (-0.71-0.7) |
| Acute lymphoid leukemia | Grand Duchy of Luxembourg | 3.6 (3.4-3.8) | 1 (0.9-1) | 2.3 (2.1-2.6) | 0.3 (0.3-0.3) | -0.95 (-1.9-0) |
| Acute lymphoid leukemia | Greenland | 0.4 (0.2-0.5) | 0.8 (0.4-1.1) | 0.2 (0.1-0.3) | 0.3 (0.2-0.5) | -0.64 (-1.29-0.03) |
| Acute lymphoid leukemia | Grenada | 0.7 (0.6-0.8) | 0.7 (0.6-0.8) | 0.5 (0.4-0.5) | 0.5 (0.4-0.5) | -0.03 (-0.68-0.62) |
| Acute lymphoid leukemia | Guam | 0.6 (0.3-0.8) | 0.5 (0.3-0.7) | 0.4 (0.2-0.6) | 0.2 (0.1-0.3) | -0.36 (-1.11-0.38) |
| Acute lymphoid leukemia | Hashemite Kingdom of Jordan | 34.7 (21.1-46.8) | 0.9 (0.5-1.3) | 43.2 (23.8-63.6) | 0.4 (0.2-0.6) | -0.79 (-1.83-0.26) |
| Acute lymphoid leukemia | Hellenic Republic | 99.2 (94.8-103.5) | 0.9 (0.8-0.9) | 80.8 (73.9-86.8) | 0.5 (0.5-0.6) | -0.69 (-1.52-0.14) |
| Acute lymphoid leukemia | Hungary | 134.2 (120.1-150.3) | 1.2 (1.1-1.4) | 70.9 (55.6-90.9) | 0.5 (0.4-0.7) | -0.82 (-1.6--0.03) |
| Acute lymphoid leukemia | Independent State of Papua New Guinea | 18.2 (7.8-35.3) | 0.4 (0.2-0.7) | 40.1 (19.1-72.5) | 0.4 (0.2-0.6) | -0.21 (-0.95-0.53) |
| Acute lymphoid leukemia | Independent State of Samoa | 0.6 (0.4-1) | 0.4 (0.3-0.7) | 0.7 (0.4-1) | 0.4 (0.2-0.5) | -0.24 (-1.16-0.68) |
| Acute lymphoid leukemia | Ireland | 33.3 (31-35.5) | 0.9 (0.8-0.9) | 19.2 (16.6-22.3) | 0.3 (0.3-0.4) | -1.01 (-1.81--0.19) |
| Acute lymphoid leukemia | Islamic Republic of Afghanistan | 329.6 (133-724.5) | 3.4 (1.3-7.6) | 752.5 (348.7-1422.2) | 2.9 (1.2-6.1) | -0.2 (-1.14-0.75) |
| Acute lymphoid leukemia | Islamic Republic of Iran | 1238.5 (762.2-1694.1) | 2 (1.2-2.7) | 802.3 (425.2-1002) | 1 (0.5-1.3) | -0.61 (-1.39-0.17) |
| Acute lymphoid leukemia | Islamic Republic of Mauritania | 8.9 (5.7-12.5) | 0.4 (0.2-0.5) | 16.5 (6.6-27.6) | 0.3 (0.1-0.5) | 0.12 (-0.24-0.47) |
| Acute lymphoid leukemia | Islamic Republic of Pakistan | 1186.3 (727.9-1775.8) | 1 (0.6-1.4) | 2133.2 (1460-3170.3) | 0.9 (0.6-1.4) | -0.15 (-0.86-0.56) |
| Acute lymphoid leukemia | Jamaica | 20.6 (18-23.3) | 0.8 (0.7-0.9) | 15.4 (12-19.6) | 0.6 (0.5-0.7) | 0.12 (-0.59-0.83) |
| Acute lymphoid leukemia | Japan | 1091.4 (1063-1110.5) | 0.8 (0.8-0.9) | 890.6 (802.7-946.6) | 0.4 (0.4-0.4) | -0.53 (-1.28-0.23) |
| Acute lymphoid leukemia | Kingdom of Bahrain | 3.4 (2-4.4) | 0.9 (0.5-1.2) | 6.1 (3.1-9.5) | 0.5 (0.3-0.7) | -1.23 (-2.22--0.23) |
| Acute lymphoid leukemia | Kingdom of Belgium | 94.8 (87.1-103.8) | 0.9 (0.8-1) | 64.9 (57.4-72.7) | 0.4 (0.4-0.5) | -0.78 (-1.6-0.05) |
| Acute lymphoid leukemia | Kingdom of Bhutan | 5.8 (2.8-9.3) | 0.9 (0.5-1.4) | 4.8 (2.9-8.1) | 0.7 (0.4-1.2) | -0.34 (-0.99-0.32) |
| Acute lymphoid leukemia | Kingdom of Cambodia | 233.3 (114.6-424.1) | 2.2 (1.2-3.8) | 242.7 (137.3-353.5) | 1.6 (0.9-2.3) | -0.48 (-1.35-0.4) |
| Acute lymphoid leukemia | Kingdom of Denmark | 40.7 (37.6-43.6) | 0.7 (0.7-0.8) | 30.8 (27.4-34.7) | 0.4 (0.3-0.4) | -0.74 (-1.6-0.13) |
| Acute lymphoid leukemia | Kingdom of Eswatini | 5.8 (4-9.1) | 0.9 (0.6-1.3) | 9.6 (5.6-14.7) | 1 (0.5-1.5) | 0.34 (-0.51-1.21) |
| Acute lymphoid leukemia | Kingdom of Lesotho | 9.1 (5.4-13.5) | 0.7 (0.4-1) | 17.9 (11.7-26) | 1.1 (0.7-1.6) | 0.94 (0.14-1.74) |
| Acute lymphoid leukemia | Kingdom of Morocco | 89.1 (44-141.9) | 0.3 (0.2-0.5) | 80.5 (32.8-119.2) | 0.2 (0.1-0.3) | -0.08 (-0.46-0.29) |
| Acute lymphoid leukemia | Kingdom of Norway | 27.8 (26.7-28.8) | 0.6 (0.6-0.6) | 24.2 (22.5-25.9) | 0.4 (0.3-0.4) | -0.33 (-1.13-0.48) |
| Acute lymphoid leukemia | Kingdom of Saudi Arabia | 124.7 (83.8-190.4) | 0.8 (0.5-1.3) | 204.8 (136-335.5) | 0.6 (0.4-1) | 0.26 (-0.34-0.87) |
| Acute lymphoid leukemia | Kingdom of Spain | 444 (423.4-465.9) | 1.1 (1.1-1.2) | 274.6 (243.6-306.9) | 0.5 (0.4-0.5) | -0.8 (-1.53--0.07) |
| Acute lymphoid leukemia | Kingdom of Sweden | 63.7 (60.1-67.4) | 0.7 (0.7-0.7) | 45.6 (40.6-51.6) | 0.3 (0.3-0.4) | -0.55 (-1.35-0.26) |
| Acute lymphoid leukemia | Kingdom of Thailand | 666.2 (383.1-949.6) | 1.3 (0.7-1.9) | 555.8 (278.1-791.4) | 0.8 (0.4-1.1) | -0.59 (-1.45-0.27) |
| Acute lymphoid leukemia | Kingdom of the Netherlands | 107.4 (100.8-114) | 0.7 (0.6-0.7) | 82.9 (73.4-92.9) | 0.3 (0.3-0.4) | -0.65 (-1.48-0.19) |
| Acute lymphoid leukemia | Kingdom of Tonga | 0.2 (0.1-0.2) | 0.2 (0.1-0.3) | 0.2 (0.1-0.3) | 0.2 (0.1-0.2) | -0.13 (-0.88-0.62) |
| Acute lymphoid leukemia | Kyrgyz Republic | 57.5 (50.5-64.7) | 1.2 (1-1.3) | 36.8 (30.3-43.8) | 0.5 (0.4-0.7) | -0.67 (-1.25--0.1) |
| Acute lymphoid leukemia | Lao People's Democratic Republic | 97.5 (42.1-183.9) | 2.2 (1.1-4.1) | 96 (56.2-138.3) | 1.4 (0.8-2) | -0.77 (-1.63-0.1) |
| Acute lymphoid leukemia | Lebanese Republic | 32.3 (20.9-50.9) | 1.2 (0.7-1.8) | 39.8 (23-56.4) | 0.7 (0.4-1) | -0.36 (-1.17-0.46) |
| Acute lymphoid leukemia | Malaysia | 290.7 (168.8-388.6) | 1.7 (1-2.2) | 367.5 (213.4-474.2) | 1.2 (0.7-1.6) | -0.31 (-1.14-0.53) |
| Acute lymphoid leukemia | Mongolia | 28 (19.5-40.8) | 1.2 (0.8-1.7) | 21.5 (13.5-29.1) | 0.7 (0.4-0.9) | -0.43 (-1.11-0.26) |
| Acute lymphoid leukemia | Montenegro | 5.5 (3.8-7.5) | 0.9 (0.6-1.2) | 3.7 (2.3-6.6) | 0.5 (0.3-0.9) | -0.12 (-0.87-0.64) |
| Acute lymphoid leukemia | New Zealand | 29.4 (27.5-31.3) | 0.9 (0.8-0.9) | 22.2 (20.3-24.1) | 0.4 (0.3-0.4) | -0.58 (-1.44-0.28) |
| Acute lymphoid leukemia | North Macedonia | 24 (16.3-31.6) | 1.3 (0.9-1.7) | 17.3 (10.3-28) | 0.7 (0.4-1.1) | -0.23 (-1.25-0.79) |
| Acute lymphoid leukemia | Northern Mariana Islands | 0.2 (0.1-0.3) | 0.8 (0.4-1.1) | 0.2 (0.1-0.3) | 0.5 (0.3-0.7) | -0.52 (-1.28-0.24) |
| Acute lymphoid leukemia | Palestine | 33 (22.8-46.7) | 1.4 (1-2.1) | 41.2 (27.8-59.4) | 0.8 (0.6-1.2) | -0.8 (-1.71-0.11) |
| Acute lymphoid leukemia | People's Democratic Republic of Algeria | 130.6 (79.2-185.6) | 0.4 (0.3-0.6) | 100.6 (52.1-145) | 0.2 (0.1-0.4) | -0.28 (-0.92-0.36) |
| Acute lymphoid leukemia | People's Republic of Bangladesh | 1475.3 (751.2-2586.3) | 1.2 (0.7-2) | 1140.4 (743.9-1641.7) | 0.7 (0.5-1.1) | -0.59 (-1.24-0.06) |
| Acute lymphoid leukemia | People's Republic of China | 33919.3 (23963.1-44727.7) | 3.1 (2.2-4) | 20612.9 (11781.4-27301.8) | 1.4 (0.8-1.7) | -1.08 (-1.87--0.29) |
| Acute lymphoid leukemia | Plurinational State of Bolivia | 212.8 (138.5-328.5) | 3.1 (2.1-4.7) | 247.5 (143.1-341.6) | 2.2 (1.3-3.1) | -0.42 (-1.3-0.48) |
| Acute lymphoid leukemia | Portuguese Republic | 114.8 (108.6-120.5) | 1.1 (1.1-1.2) | 66.2 (58.8-73.9) | 0.4 (0.4-0.5) | -0.79 (-1.59-0.01) |
| Acute lymphoid leukemia | Principality of Andorra | 0.5 (0.3-0.9) | 1 (0.6-1.7) | 0.5 (0.3-0.9) | 0.5 (0.3-0.8) | -0.9 (-1.78--0.01) |
| Acute lymphoid leukemia | Principality of Monaco | 0.5 (0.3-0.7) | 1.4 (0.9-1.9) | 0.5 (0.3-0.8) | 1.1 (0.7-1.6) | 0.1 (-0.95-1.17) |
| Acute lymphoid leukemia | Puerto Rico | 34.4 (31.5-37.3) | 1 (0.9-1) | 28.7 (23.5-34) | 0.6 (0.5-0.7) | -0.7 (-1.46-0.06) |
| Acute lymphoid leukemia | Republic of Albania | 36.9 (26.6-49) | 1.1 (0.8-1.6) | 17.1 (9.6-30.3) | 0.6 (0.3-1) | -0.39 (-1.07-0.3) |
| Acute lymphoid leukemia | Republic of Angola | 85.4 (43.8-154.8) | 0.8 (0.4-1.2) | 178.2 (94.3-257.4) | 0.6 (0.3-0.9) | -0.09 (-0.64-0.47) |
| Acute lymphoid leukemia | Republic of Armenia | 75 (67.1-83.3) | 2.2 (2-2.5) | 26.4 (22.8-30.4) | 0.8 (0.7-0.9) | -0.62 (-1.3-0.06) |
| Acute lymphoid leukemia | Republic of Austria | 51 (47.7-54.5) | 0.6 (0.6-0.7) | 35.9 (32.1-40.1) | 0.3 (0.3-0.3) | -0.49 (-1.3-0.34) |
| Acute lymphoid leukemia | Republic of Azerbaijan | 119.1 (80.8-155) | 1.6 (1.1-2) | 93.3 (59.1-144.6) | 1 (0.6-1.5) | -0.64 (-1.38-0.1) |
| Acute lymphoid leukemia | Republic of Belarus | 163.4 (143.4-188.2) | 1.6 (1.4-1.8) | 54.5 (44.1-65.7) | 0.5 (0.4-0.6) | -0.69 (-1.4-0.03) |
| Acute lymphoid leukemia | Republic of Benin | 30 (17.5-45.6) | 0.4 (0.3-0.6) | 76.5 (29.6-117.1) | 0.4 (0.2-0.7) | 0.2 (-0.18-0.58) |
| Acute lymphoid leukemia | Republic of Botswana | 8.7 (5.1-12.6) | 0.8 (0.5-1.2) | 13.5 (7.7-20.1) | 0.6 (0.4-0.9) | -0.07 (-0.81-0.67) |
| Acute lymphoid leukemia | Republic of Bulgaria | 105.5 (89.9-120.9) | 1.2 (1.1-1.4) | 40.3 (30.2-51.6) | 0.5 (0.4-0.6) | 0.05 (-0.67-0.78) |
| Acute lymphoid leukemia | Republic of Burundi | 61 (38.7-94.1) | 0.9 (0.6-1.3) | 94.8 (46.3-154.3) | 0.7 (0.3-1.1) | -0.13 (-0.68-0.42) |
| Acute lymphoid leukemia | Republic of Cabo Verde | 3.4 (2.4-4.8) | 0.8 (0.6-1.1) | 3.7 (2.2-5.1) | 0.7 (0.4-0.9) | 0.14 (-0.46-0.75) |
| Acute lymphoid leukemia | Republic of Cameroon | 56.2 (34.3-80.2) | 0.4 (0.3-0.6) | 163.7 (65.3-246.4) | 0.4 (0.2-0.7) | 0.23 (-0.18-0.64) |
| Acute lymphoid leukemia | Republic of Chad | 31.2 (17.4-49.7) | 0.4 (0.2-0.6) | 120.2 (50.4-197.5) | 0.5 (0.2-0.8) | 0.39 (0.02-0.77) |
| Acute lymphoid leukemia | Republic of Chile | 159.1 (150.6-167.1) | 1.2 (1.2-1.3) | 175.5 (160.3-190.9) | 0.8 (0.8-0.9) | -0.41 (-1.09-0.27) |
| Acute lymphoid leukemia | Republic of Colombia | 619.6 (572.5-663.2) | 1.8 (1.7-2) | 744 (629.5-874.9) | 1.5 (1.3-1.8) | -0.44 (-1.19-0.31) |
| Acute lymphoid leukemia | Republic of Costa Rica | 44 (40.8-47.2) | 1.5 (1.4-1.6) | 84.9 (75.7-94.5) | 1.7 (1.5-1.9) | -0.11 (-0.88-0.68) |
| Acute lymphoid leukemia | République de Côte d’Ivoire | 43.8 (22.4-62) | 0.3 (0.2-0.4) | 80.8 (37.3-135.4) | 0.3 (0.1-0.4) | -0.02 (-0.32-0.28) |
| Acute lymphoid leukemia | Republic of Croatia | 56.9 (47.3-68.1) | 1.1 (0.9-1.3) | 24.8 (18.8-32.2) | 0.4 (0.3-0.5) | -0.54 (-1.3-0.23) |
| Acute lymphoid leukemia | Republic of Cuba | 111.8 (103.2-119.7) | 1.1 (1-1.1) | 87.7 (76.2-100) | 0.7 (0.6-0.8) | -0.44 (-1.14-0.26) |
| Acute lymphoid leukemia | Republic of Cyprus | 8.6 (5.9-13.4) | 1.2 (0.8-1.9) | 9.2 (5.2-12.7) | 0.6 (0.3-0.8) | -0.67 (-1.65-0.33) |
| Acute lymphoid leukemia | Republic of Djibouti | 3.1 (1.8-4.7) | 0.7 (0.4-1) | 7.6 (4-12.9) | 0.6 (0.3-1.1) | 0.21 (-0.35-0.77) |
| Acute lymphoid leukemia | Republic of Ecuador | 171.3 (159.3-183.6) | 1.7 (1.6-1.8) | 379.1 (312.2-449.4) | 2.2 (1.8-2.6) | 0.33 (-0.52-1.18) |
| Acute lymphoid leukemia | Republic of El Salvador | 119.2 (96.3-159.2) | 2 (1.6-2.7) | 108 (63-140.3) | 1.7 (1-2.2) | -0.02 (-0.83-0.79) |
| Acute lymphoid leukemia | Republic of Equatorial Guinea | 3.2 (1.8-5.5) | 0.7 (0.4-1.1) | 6.4 (2.8-11.4) | 0.5 (0.2-0.8) | -0.19 (-0.72-0.35) |
| Acute lymphoid leukemia | Republic of Estonia | 14.3 (12.7-16.2) | 0.9 (0.8-1.1) | 5 (4.2-6) | 0.3 (0.2-0.3) | -1 (-1.8--0.2) |
| Acute lymphoid leukemia | Republic of Fiji | 2.9 (0.6-4.8) | 0.4 (0.1-0.7) | 2.1 (0.6-3.6) | 0.4 (0.1-0.5) | -0.32 (-1.45-0.82) |
| Acute lymphoid leukemia | Republic of Finland | 41 (38.3-43.9) | 0.7 (0.7-0.8) | 38.1 (34.1-42.3) | 0.4 (0.4-0.5) | -0.61 (-1.32-0.11) |
| Acute lymphoid leukemia | Republic of Ghana | 169.9 (64.9-252.4) | 0.9 (0.4-1.3) | 160 (92.1-251.1) | 0.4 (0.3-0.6) | -0.75 (-1.24--0.27) |
| Acute lymphoid leukemia | Republic of Guatemala | 140 (125.2-157) | 1.4 (1.3-1.5) | 287.4 (247.1-335.1) | 1.8 (1.6-2.2) | -0.01 (-0.78-0.77) |
| Acute lymphoid leukemia | Republic of Guinea | 18.5 (9.2-27.9) | 0.2 (0.1-0.3) | 26.5 (10.8-50.4) | 0.2 (0.1-0.3) | -0.02 (-0.21-0.16) |
| Acute lymphoid leukemia | Republic of Guinea-Bissau | 7.1 (3.9-11.8) | 0.5 (0.3-0.9) | 11.5 (5-17) | 0.5 (0.2-0.7) | 0.24 (-0.2-0.68) |
| Acute lymphoid leukemia | Republic of Guyana | 8.3 (6.9-9.5) | 1 (0.9-1.2) | 9.5 (7.2-12.1) | 1.3 (1-1.6) | 0.13 (-0.52-0.78) |
| Acute lymphoid leukemia | Republic of Haiti | 181.7 (72.7-361.5) | 2.4 (1.1-4.5) | 232.8 (120.8-432.6) | 1.8 (1-3.3) | -0.34 (-1.08-0.41) |
| Acute lymphoid leukemia | Republic of Honduras | 129.5 (98.4-162.8) | 2.5 (1.8-3.1) | 157.2 (88.7-237.4) | 1.8 (1-2.5) | -0.13 (-0.92-0.67) |
| Acute lymphoid leukemia | Republic of Iceland | 1.8 (1.7-2) | 0.7 (0.6-0.7) | 1.9 (1.7-2.2) | 0.4 (0.4-0.5) | -0.35 (-1.12-0.43) |
| Acute lymphoid leukemia | Republic of India | 7630.3 (5228.1-11254) | 0.9 (0.6-1.3) | 7323.4 (4661.3-10014.8) | 0.6 (0.3-0.8) | -0.31 (-0.92-0.29) |
| Acute lymphoid leukemia | Republic of Indonesia | 2737.8 (1889.9-4092.5) | 1.5 (1-2.2) | 3163.3 (1858.8-4206.7) | 1.3 (0.8-1.7) | -0.16 (-0.99-0.69) |
| Acute lymphoid leukemia | Republic of Iraq | 257.3 (160.1-386) | 1.2 (0.8-1.8) | 293.6 (164.5-411.7) | 0.8 (0.4-1.1) | -0.36 (-1.22-0.5) |
| Acute lymphoid leukemia | Republic of Italy | 618.8 (597.6-636.6) | 1.1 (1-1.1) | 471.8 (428.6-502.7) | 0.5 (0.5-0.6) | -0.84 (-1.62--0.04) |
| Acute lymphoid leukemia | Republic of Kazakhstan | 182.2 (151.6-209.1) | 1.1 (0.9-1.3) | 95.1 (81-114.1) | 0.5 (0.4-0.6) | -0.54 (-1.21-0.12) |
| Acute lymphoid leukemia | Republic of Kenya | 123 (82.4-169.8) | 0.5 (0.3-0.7) | 228.2 (135.7-315.8) | 0.5 (0.3-0.7) | 0.29 (-0.18-0.76) |
| Acute lymphoid leukemia | Republic of Kiribati | 0.3 (0.2-0.5) | 0.4 (0.3-0.6) | 0.4 (0.2-0.6) | 0.4 (0.2-0.5) | -0.04 (-0.76-0.68) |
| Acute lymphoid leukemia | Republic of Korea | 559 (369.7-766.3) | 1.3 (0.9-1.8) | 240.5 (123.7-364.2) | 0.4 (0.2-0.6) | -1 (-1.72--0.27) |
| Acute lymphoid leukemia | Republic of Latvia | 30 (26.4-33.5) | 1.1 (1-1.3) | 10 (8.5-12) | 0.4 (0.3-0.4) | -0.81 (-1.58--0.03) |
| Acute lymphoid leukemia | Republic of Liberia | 15.2 (8.5-25.4) | 0.4 (0.3-0.7) | 26.5 (10.3-40.9) | 0.4 (0.2-0.7) | 0.23 (-0.14-0.6) |
| Acute lymphoid leukemia | Republic of Lithuania | 36.6 (32.8-40.8) | 1 (0.9-1.1) | 17.8 (14.9-21.1) | 0.5 (0.4-0.5) | -0.54 (-1.31-0.25) |
| Acute lymphoid leukemia | Republic of Madagascar | 99.9 (66.7-147.3) | 0.7 (0.5-1) | 178.6 (104.9-260.9) | 0.6 (0.3-0.8) | 0.01 (-0.48-0.5) |
| Acute lymphoid leukemia | Republic of Malawi | 46.6 (28-65.3) | 0.3 (0.2-0.5) | 47.6 (21.2-87.2) | 0.2 (0.1-0.4) | -0.09 (-0.38-0.2) |
| Acute lymphoid leukemia | Republic of Maldives | 3.4 (1.6-6.3) | 1.5 (0.8-2.7) | 2.5 (1.6-3.9) | 0.6 (0.4-0.9) | -1.16 (-1.89--0.42) |
| Acute lymphoid leukemia | Republic of Mali | 49.2 (23.2-73.1) | 0.4 (0.2-0.6) | 83.2 (37.3-151) | 0.3 (0.1-0.5) | -0.11 (-0.42-0.2) |
| Acute lymphoid leukemia | Republic of Malta | 3 (2.7-3.3) | 0.8 (0.7-0.9) | 2.7 (2.3-3.1) | 0.5 (0.4-0.6) | -0.66 (-1.46-0.15) |
| Acute lymphoid leukemia | Republic of Mauritius | 10.2 (9.5-10.9) | 1 (0.9-1.1) | 6.2 (5.7-6.6) | 0.5 (0.4-0.5) | -0.51 (-1.24-0.22) |
| Acute lymphoid leukemia | Republic of Moldova | 83.1 (73.8-90.7) | 1.9 (1.7-2.1) | 29.3 (26-33.1) | 0.7 (0.6-0.8) | -0.74 (-1.42--0.06) |
| Acute lymphoid leukemia | Republic of Mozambique | 247.5 (158.1-383.3) | 1.3 (0.9-2) | 327.6 (178.8-573.4) | 0.9 (0.6-1.5) | 0.14 (-0.52-0.8) |
| Acute lymphoid leukemia | Republic of Namibia | 7.5 (4.5-10.6) | 0.6 (0.4-0.8) | 12.4 (7.1-19) | 0.6 (0.3-0.9) | -0.03 (-0.65-0.6) |
| Acute lymphoid leukemia | Republic of Nauru | 0 (0-0.1) | 0.5 (0.3-0.7) | 0 (0-0.1) | 0.5 (0.3-0.8) | -0.22 (-1.15-0.73) |
| Acute lymphoid leukemia | Republic of Nicaragua | 94.9 (76.4-123.4) | 2 (1.6-2.7) | 86.8 (52.1-111.4) | 1.4 (0.8-1.8) | -0.21 (-0.94-0.53) |
| Acute lymphoid leukemia | Republic of Niue | 0 (0-0) | 0.4 (0.2-0.5) | 0 (0-0) | 0.8 (0.5-1.1) | -0.06 (-0.86-0.74) |
| Acute lymphoid leukemia | Republic of Palau | 0.1 (0-0.1) | 0.6 (0.3-0.8) | 0.1 (0-0.1) | 0.5 (0.3-0.6) | -0.16 (-0.66-0.36) |
| Acute lymphoid leukemia | Republic of Panama | 36 (33.1-38.9) | 1.4 (1.3-1.5) | 65.8 (55-78) | 1.5 (1.3-1.8) | 0.06 (-0.7-0.83) |
| Acute lymphoid leukemia | Republic of Paraguay | 58.7 (41.9-72.9) | 1.3 (0.9-1.7) | 84.3 (53-114.1) | 1.2 (0.8-1.7) | 0.24 (-0.47-0.96) |
| Acute lymphoid leukemia | Republic of Peru | 415.1 (320.2-586.8) | 1.8 (1.4-2.6) | 541.4 (276.3-725.1) | 1.5 (0.8-2.1) | -0.18 (-0.97-0.62) |
| Acute lymphoid leukemia | Republic of Poland | 357.2 (311.2-403.1) | 0.9 (0.8-1.1) | 213.7 (195.3-233.9) | 0.4 (0.4-0.5) | -0.79 (-1.55--0.02) |
| Acute lymphoid leukemia | Republic of Rwanda | 87.7 (57.9-132.1) | 1 (0.7-1.5) | 89 (45.8-145) | 0.7 (0.4-1) | -0.5 (-1.08-0.08) |
| Acute lymphoid leukemia | Republic of San Marino | 0.5 (0.4-0.8) | 2 (1.5-2.8) | 0.5 (0.3-0.8) | 1.1 (0.6-1.6) | -0.78 (-1.6-0.04) |
| Acute lymphoid leukemia | Republic of Senegal | 46.5 (26-66.9) | 0.4 (0.3-0.6) | 71.3 (28-122.3) | 0.4 (0.2-0.7) | 0.23 (-0.16-0.61) |
| Acute lymphoid leukemia | Republic of Serbia | 94.1 (62-123.5) | 1 (0.7-1.4) | 58.2 (32.5-82.4) | 0.5 (0.3-0.6) | -0.51 (-1.26-0.25) |
| Acute lymphoid leukemia | Republic of Seychelles | 0.8 (0.5-1.1) | 1.2 (0.7-1.6) | 0.8 (0.3-1.1) | 0.7 (0.3-0.9) | -0.43 (-1.15-0.29) |
| Acute lymphoid leukemia | Republic of Sierra Leone | 24.9 (12.9-41.8) | 0.4 (0.2-0.7) | 47.3 (16.8-72.9) | 0.5 (0.2-0.7) | 0.26 (-0.11-0.64) |
| Acute lymphoid leukemia | Republic of Singapore | 28.9 (27.3-30.7) | 1.1 (1-1.1) | 30.7 (28.4-33) | 0.5 (0.5-0.6) | -0.85 (-1.66--0.04) |
| Acute lymphoid leukemia | Republic of Slovenia | 21.8 (19.7-23.9) | 1.1 (1-1.2) | 15.1 (12.2-18.1) | 0.5 (0.4-0.5) | -0.5 (-1.23-0.23) |
| Acute lymphoid leukemia | Republic of South Africa | 264 (186.4-349.4) | 0.8 (0.5-1) | 386.6 (223.7-509.8) | 0.7 (0.4-1) | 0.09 (-0.68-0.86) |
| Acute lymphoid leukemia | Republic of South Sudan | 54.3 (33.3-89.5) | 0.8 (0.5-1.2) | 100.6 (62.6-156.9) | 0.9 (0.6-1.4) | 0.3 (-0.25-0.86) |
| Acute lymphoid leukemia | Republic of Sudan | 466.8 (206.5-910.9) | 2.1 (0.9-4.1) | 621.2 (265.5-968.2) | 1.5 (0.6-2.4) | -0.33 (-1.16-0.51) |
| Acute lymphoid leukemia | Republic of Suriname | 3.1 (1.7-3.9) | 0.7 (0.4-0.9) | 3.4 (2.2-4.7) | 0.6 (0.4-0.8) | -0.16 (-0.77-0.45) |
| Acute lymphoid leukemia | Republic of Tajikistan | 86.7 (53.6-118.4) | 1.4 (0.9-1.9) | 89 (51.4-148) | 0.8 (0.5-1.3) | -0.75 (-1.39--0.1) |
| Acute lymphoid leukemia | Republic of the Congo | 15.7 (9.7-24.7) | 0.7 (0.4-1) | 27.5 (14.8-39.7) | 0.6 (0.3-0.8) | -0.14 (-0.73-0.45) |
| Acute lymphoid leukemia | Republic of the Gambia | 2.8 (1.4-4.1) | 0.2 (0.1-0.3) | 4.9 (2.7-7.8) | 0.2 (0.1-0.3) | -0.02 (-0.24-0.2) |
| Acute lymphoid leukemia | Republic of the Marshall Islands | 0.1 (0.1-0.2) | 0.4 (0.2-0.5) | 0.2 (0.1-0.3) | 0.4 (0.2-0.6) | -0.05 (-0.88-0.79) |
| Acute lymphoid leukemia | Republic of the Niger | 60.4 (31.3-104.7) | 0.5 (0.3-0.8) | 137.1 (46.3-225.2) | 0.4 (0.2-0.7) | 0.12 (-0.25-0.5) |
| Acute lymphoid leukemia | Republic of the Philippines | 948.5 (681.5-1325.5) | 1.4 (1-1.9) | 1131 (720.4-1403.1) | 1.1 (0.7-1.4) | -0.35 (-1.19-0.49) |
| Acute lymphoid leukemia | Republic of the Union of Myanmar | 953.4 (433-1800.2) | 2.4 (1.2-4.3) | 749.1 (463.3-1017.1) | 1.4 (0.9-1.9) | -0.95 (-1.84--0.07) |
| Acute lymphoid leukemia | Republic of Trinidad and Tobago | 12.3 (10.9-13.6) | 1 (0.9-1.1) | 10.4 (8.2-13.1) | 0.7 (0.6-0.9) | -0.43 (-1.12-0.27) |
| Acute lymphoid leukemia | Republic of Tunisia | 74.2 (39-99.8) | 0.9 (0.4-1.2) | 58 (21.1-88.5) | 0.5 (0.2-0.8) | -0.48 (-1.11-0.16) |
| Acute lymphoid leukemia | Republic of Turkey | 1272.2 (803.3-1913.9) | 2.2 (1.4-3.2) | 769 (428-1030.4) | 0.9 (0.5-1.2) | -1.01 (-1.89--0.12) |
| Acute lymphoid leukemia | Republic of Uganda | 112.9 (68.7-163.4) | 0.5 (0.3-0.7) | 233.1 (112.9-376.9) | 0.5 (0.2-0.7) | -0.04 (-0.5-0.43) |
| Acute lymphoid leukemia | Republic of Uzbekistan | 303.7 (250.2-368.4) | 1.4 (1.1-1.7) | 263.8 (214.4-328.4) | 0.8 (0.6-1) | -0.68 (-1.33--0.03) |
| Acute lymphoid leukemia | Republic of Vanuatu | 0.4 (0.2-0.6) | 0.3 (0.2-0.5) | 0.8 (0.4-1.2) | 0.3 (0.2-0.5) | -0.13 (-0.91-0.65) |
| Acute lymphoid leukemia | Republic of Yemen | 244.5 (92.6-496.6) | 1.7 (0.6-3.5) | 392.4 (137.9-645.5) | 1.3 (0.4-2.2) | -0.28 (-1.07-0.51) |
| Acute lymphoid leukemia | Republic of Zambia | 87.4 (61-129.4) | 0.9 (0.6-1.2) | 122.2 (65.7-182.6) | 0.6 (0.3-0.8) | -0.05 (-0.63-0.53) |
| Acute lymphoid leukemia | Republic of Zimbabwe | 65.3 (37.2-86.2) | 0.8 (0.4-1.1) | 166.5 (95.3-231.8) | 1.2 (0.7-1.7) | 0.62 (-0.21-1.45) |
| Acute lymphoid leukemia | Romania | 270.3 (249.5-290.4) | 1.2 (1.1-1.3) | 169.3 (146.8-192.6) | 0.7 (0.6-0.8) | -0.11 (-0.79-0.57) |
| Acute lymphoid leukemia | Russian Federation | 1935 (1804.6-2051.9) | 1.3 (1.2-1.4) | 1138.4 (1056.9-1222.7) | 0.6 (0.6-0.7) | -0.44 (-1.09-0.21) |
| Acute lymphoid leukemia | Saint Kitts and Nevis | 0.4 (0.4-0.5) | 0.9 (0.8-1.1) | 0.3 (0.2-0.3) | 0.6 (0.5-0.7) | -0.42 (-1.11-0.28) |
| Acute lymphoid leukemia | Saint Lucia | 1.1 (1-1.3) | 0.8 (0.7-0.9) | 0.9 (0.8-1.1) | 0.6 (0.5-0.7) | -0.73 (-1.34--0.11) |
| Acute lymphoid leukemia | Saint Vincent and the Grenadines | 0.9 (0.7-1) | 0.7 (0.6-0.7) | 0.6 (0.5-0.7) | 0.6 (0.5-0.7) | -0.29 (-0.95-0.37) |
| Acute lymphoid leukemia | Slovak Republic | 49.5 (29.8-66.4) | 0.9 (0.6-1.2) | 29 (15.8-43) | 0.5 (0.3-0.7) | -0.52 (-1.32-0.29) |
| Acute lymphoid leukemia | Socialist Republic of Viet Nam | 814.3 (581.1-1057.3) | 1.4 (0.9-1.8) | 932.6 (572.3-1289.2) | 1 (0.6-1.3) | -0.39 (-1.08-0.3) |
| Acute lymphoid leukemia | Solomon Islands | 1.2 (0.6-2.1) | 0.4 (0.2-0.6) | 2.1 (1.1-3.2) | 0.4 (0.2-0.6) | -0.08 (-0.9-0.74) |
| Acute lymphoid leukemia | State of Eritrea | 31.3 (18-50.2) | 0.8 (0.5-1.2) | 52.7 (28.9-82.6) | 0.8 (0.4-1.2) | 0.18 (-0.38-0.75) |
| Acute lymphoid leukemia | State of Israel | 46.6 (42.6-50.2) | 0.9 (0.8-1) | 42.7 (38.9-47.1) | 0.4 (0.4-0.4) | -0.73 (-1.7-0.25) |
| Acute lymphoid leukemia | State of Kuwait | 15.6 (14.2-17.1) | 1.1 (1-1.2) | 16.7 (13.5-20) | 0.4 (0.4-0.5) | -1.06 (-1.75--0.36) |
| Acute lymphoid leukemia | State of Libya | 59.3 (31.9-80.3) | 1.5 (0.7-2) | 104.4 (47.8-152.5) | 1.7 (0.8-2.5) | 0.11 (-0.77-0.99) |
| Acute lymphoid leukemia | State of Qatar | 2.7 (1.4-3.8) | 0.8 (0.4-1.2) | 7.9 (4-11.7) | 0.4 (0.2-0.5) | -1.42 (-2.21--0.63) |
| Acute lymphoid leukemia | Sultanate of Oman | 4.4 (2.2-7) | 0.2 (0.1-0.4) | 3.5 (1.6-5.3) | 0.1 (0-0.2) | -0.44 (-1.2-0.33) |
| Acute lymphoid leukemia | Swiss Confederation | 54.8 (49.7-59.8) | 0.8 (0.7-0.8) | 35.6 (31.6-39.8) | 0.3 (0.3-0.3) | -0.68 (-1.44-0.08) |
| Acute lymphoid leukemia | Syrian Arab Republic | 272.4 (148.2-404.1) | 2.1 (1-3.2) | 165.4 (77.4-231) | 1.2 (0.6-1.7) | -0.8 (-1.65-0.06) |
| Acute lymphoid leukemia | Taiwan (Province of China) | 101.6 (75.8-125.1) | 0.5 (0.4-0.6) | 129.5 (116.4-142.5) | 0.4 (0.4-0.5) | 0.19 (-0.59-0.99) |
| Acute lymphoid leukemia | Togolese Republic | 18.3 (10-25.8) | 0.4 (0.2-0.5) | 36.6 (12.9-58.6) | 0.4 (0.1-0.6) | 0.28 (-0.09-0.66) |
| Acute lymphoid leukemia | Tokelau | 0 (0-0) | 0.4 (0.2-0.6) | 0 (0-0) | 0.9 (0.4-1.5) | -0.17 (-0.97-0.64) |
| Acute lymphoid leukemia | Turkmenistan | 43 (34.8-52.7) | 1.1 (0.9-1.3) | 36.5 (27.8-46.4) | 0.7 (0.5-0.9) | -0.41 (-1.04-0.21) |
| Acute lymphoid leukemia | Tuvalu | 0 (0-0.1) | 0.5 (0.3-0.9) | 0 (0-0.1) | 0.3 (0.2-0.5) | -0.34 (-1.16-0.48) |
| Acute lymphoid leukemia | Ukraine | 949.9 (830.8-1088.7) | 1.8 (1.6-2.1) | 393.6 (300.8-503.3) | 0.8 (0.6-0.9) | -1.13 (-1.8--0.46) |
| Acute lymphoid leukemia | Union of the Comoros | 4.3 (2.5-6.1) | 0.8 (0.5-1.1) | 5.4 (3.1-7.9) | 0.7 (0.4-1.1) | 0.02 (-0.53-0.57) |
| Acute lymphoid leukemia | United Arab Emirates | 15.3 (8.7-24.3) | 1.1 (0.6-1.8) | 30.1 (18.6-45.4) | 0.5 (0.3-0.7) | -0.09 (-0.97-0.79) |
| Acute lymphoid leukemia | United Kingdom of Great Britain and Northern Ireland | 421.3 (408.3-435.9) | 0.7 (0.7-0.7) | 308.5 (294.8-318.8) | 0.4 (0.4-0.4) | -0.46 (-1.32-0.41) |
| Acute lymphoid leukemia | United Mexican States | 1839.2 (1749.8-1947.6) | 2 (1.9-2.1) | 2641.2 (2408.5-2884.1) | 2.1 (1.9-2.3) | -0.21 (-1.03-0.62) |
| Acute lymphoid leukemia | United Republic of Tanzania | 277 (194-381) | 0.8 (0.6-1.2) | 459.2 (256.4-688.2) | 0.7 (0.4-1) | -0.01 (-0.56-0.55) |
| Acute lymphoid leukemia | United States of America | 2349.5 (2273.5-2399.7) | 0.9 (0.9-0.9) | 2205.2 (2071.1-2295.6) | 0.5 (0.5-0.5) | -0.7 (-1.61-0.21) |
| Acute lymphoid leukemia | United States Virgin Islands | 0.7 (0.5-0.9) | 0.7 (0.4-0.8) | 0.3 (0.2-0.5) | 0.4 (0.2-0.6) | -0.54 (-1.26-0.18) |
| Acute lymphoid leukemia | African Union | 6603 (4439.09-10153.29) | 0.94 (0.64-1.39) | 10037.09 (5749.5-12697.11) | 0.73 (0.4-0.91) | -0.69 (-0.74--0.64) |
| Acute lymphoid leukemia | Association of Southeast Asian Nations | 6772.94 (4735.55-9791.28) | 1.58 (1.05-2.21) | 7270.98 (4437.44-9232.6) | 1.14 (0.71-1.44) | -1.05 (-1.13--0.97) |
| Acute lymphoid leukemia | Central Europe, Eastern Europe, and Central Asia | 5586.05 (5222.6-5970.19) | 1.34 (1.25-1.43) | 3110.63 (2899.21-3323.14) | 0.66 (0.61-0.71) | -2.42 (-2.58--2.27) |
| Acute lymphoid leukemia | Commonwealth | 13861.68 (9699.83-19682.85) | 0.9 (0.66-1.23) | 15438.95 (10427.06-19817.76) | 0.62 (0.42-0.79) | -1.28 (-1.35--1.21) |
| Acute lymphoid leukemia | European Union | 4202.04 (4047.03-4336.4) | 0.96 (0.93-0.99) | 2830.2 (2632.71-3018.13) | 0.45 (0.43-0.48) | -2.38 (-2.43--2.33) |
| Acute lymphoid leukemia | Four World Regions | 82673.59 (63926.61-105445.55) | 1.55 (1.22-1.96) | 71138.83 (49101.01-83081.23) | 0.9 (0.62-1.05) | -1.73 (-1.76--1.7) |
| Acute lymphoid leukemia | G20 | 60651.95 (47000.67-76187.73) | 1.68 (1.31-2.09) | 45208.35 (32172.28-54616.88) | 0.87 (0.61-1.04) | -2.16 (-2.2--2.12) |
| Acute lymphoid leukemia | Gulf Cooperation Council | 166.09 (118.54-243.21) | 0.78 (0.56-1.14) | 269.03 (182.52-417.11) | 0.54 (0.36-0.79) | -1.03 (-1.32--0.75) |
| Acute lymphoid leukemia | Health System Grouping Levels | 82709.79 (63962.66-105482.43) | 1.55 (1.22-1.96) | 71168.61 (49129.93-83111.97) | 0.9 (0.62-1.05) | -1.73 (-1.76--1.7) |
| Acute lymphoid leukemia | High SDI | 8034.29 (7668.96-8659.37) | 0.89 (0.85-0.96) | 6763.21 (6187.12-7331.69) | 0.47 (0.43-0.51) | -1.97 (-2--1.93) |
| Acute lymphoid leukemia | High-income | 8561.98 (8297.46-8862.86) | 0.92 (0.89-0.95) | 6977.06 (6499.55-7315.66) | 0.48 (0.46-0.5) | -1.99 (-2.03--1.95) |
| Acute lymphoid leukemia | High-middle SDI | 20530.78 (16099.77-24578.65) | 2.01 (1.58-2.42) | 14078.4 (9270.71-17115.88) | 0.97 (0.64-1.16) | -2.42 (-2.49--2.35) |
| Acute lymphoid leukemia | Latin America and Caribbean | 6158.75 (5766.07-6761.85) | 1.5 (1.41-1.64) | 8004.56 (7179.17-8908.58) | 1.37 (1.22-1.53) | -0.06 (-0.16-0.03) |
| Acute lymphoid leukemia | Low SDI | 6419.06 (3681.45-10643.99) | 1.16 (0.74-1.84) | 9130.83 (5730.46-11582.31) | 0.82 (0.5-1.03) | -1.22 (-1.27--1.16) |
| Acute lymphoid leukemia | Low-middle SDI | 13287.67 (9223.43-19205.65) | 1.09 (0.78-1.52) | 14555.93 (9979.1-17945.91) | 0.8 (0.55-0.99) | -0.94 (-1.01--0.88) |
| Acute lymphoid leukemia | Middle SDI | 34437.99 (25547.71-43965.85) | 2.03 (1.5-2.56) | 26640.24 (18085.29-31949.52) | 1.09 (0.75-1.3) | -1.94 (-1.99--1.89) |
| Acute lymphoid leukemia | Nordic Region | 175.43 (168.47-182.03) | 0.68 (0.66-0.71) | 140.8 (130.61-152.32) | 0.37 (0.35-0.4) | -2.08 (-2.19--1.97) |
| Acute lymphoid leukemia | North Africa and Middle East | 5414.92 (3426.02-7984.85) | 1.54 (0.94-2.27) | 5700.02 (2938.65-7331.4) | 0.99 (0.51-1.27) | -1.21 (-1.29--1.13) |
| Acute lymphoid leukemia | OECD Countries | 12652.23 (11985.53-13494.49) | 1.14 (1.08-1.22) | 11103.48 (10392.57-11794.01) | 0.7 (0.65-0.75) | -1.45 (-1.5--1.41) |
| Acute lymphoid leukemia | Organization of Islamic Cooperation | 13382.5 (9256.05-19123.11) | 1.22 (0.83-1.7) | 16063.79 (9841.87-19227.68) | 0.87 (0.52-1.04) | -0.97 (-1.02--0.91) |
| Acute lymphoid leukemia | Sahel Region | 1427.26 (809.62-2434.59) | 0.8 (0.46-1.31) | 2790.01 (1364.5-3701.37) | 0.63 (0.31-0.83) | -0.63 (-0.69--0.57) |
| Acute lymphoid leukemia | South Asia | 10505.56 (6929.68-15737.08) | 0.94 (0.65-1.36) | 10792.28 (7303.36-14709.08) | 0.62 (0.42-0.86) | -1.41 (-1.5--1.33) |
| Acute lymphoid leukemia | Southeast Asia, East Asia, and Oceania | 41478.91 (29148.67-55366.23) | 2.56 (1.83-3.38) | 28728.73 (16931.95-36573.43) | 1.27 (0.76-1.59) | -2.27 (-2.33--2.21) |
| Acute lymphoid leukemia | Sub-Saharan Africa | 5062.86 (3269.9-7932.84) | 0.9 (0.63-1.34) | 7907.4 (4745.19-10147.88) | 0.67 (0.4-0.86) | -0.96 (-0.99--0.93) |
| Acute lymphoid leukemia | WHO region | 82571.58 (63809.17-105342.08) | 1.56 (1.22-1.96) | 71009.17 (48977.84-82956.16) | 0.9 (0.62-1.05) | -1.73 (-1.76--1.71) |
| Acute lymphoid leukemia | World Bank Income Levels | 82709.75 (63962.63-105482.39) | 1.55 (1.22-1.96) | 71168.57 (49129.91-83111.94) | 0.9 (0.62-1.05) | -1.73 (-1.76--1.7) |
| Acute lymphoid leukemia | World Bank Regions | 82685.77 (63945.18-105456.53) | 1.55 (1.22-1.96) | 71151.29 (49120.05-83087.66) | 0.9 (0.62-1.05) | -1.73 (-1.76--1.7) |
| Acute myeloid leukemia | American Samoa | 0.8 (0.5-1) | 2.3 (1.3-3.1) | 0.6 (0.4-0.9) | 1.2 (0.9-2) | 0.18 (-0.62-1) |
| Acute myeloid leukemia | Antigua and Barbuda | 0.8 (0.7-0.9) | 1.4 (1.3-1.6) | 1.8 (1.7-2) | 1.9 (1.7-2) | -0.2 (-0.97-0.58) |
| Acute myeloid leukemia | Arab Republic of Egypt | 715.3 (448.8-1390.5) | 1.9 (1.2-3.8) | 2089.4 (1434.3-3066.2) | 2.9 (1.9-4.6) | 0.92 (0.11-1.74) |
| Acute myeloid leukemia | Argentine Republic | 624.6 (569.5-689.8) | 1.9 (1.7-2.1) | 1012.3 (923.6-1122.3) | 1.9 (1.7-2.1) | -0.52 (-1.27-0.24) |
| Acute myeloid leukemia | Australia | 422.2 (392.2-454.2) | 2.2 (2.1-2.4) | 1351.5 (1198.8-1494.6) | 3 (2.7-3.3) | -0.68 (-1.46-0.1) |
| Acute myeloid leukemia | Barbados | 4.9 (4.5-5.3) | 1.8 (1.7-2) | 9.8 (7.6-12.1) | 2.3 (1.8-2.9) | -0.03 (-0.83-0.77) |
| Acute myeloid leukemia | Belize | 0.5 (0.5-0.6) | 0.3 (0.3-0.4) | 1.8 (1.6-2) | 0.5 (0.4-0.5) | -0.03 (-0.62-0.57) |
| Acute myeloid leukemia | Bermuda | 1.6 (1.5-1.9) | 2.7 (2.4-3.1) | 2 (1.7-2.5) | 1.9 (1.5-2.2) | -0.87 (-1.63--0.11) |
| Acute myeloid leukemia | Bolivarian Republic of Venezuela | 193.9 (180.2-213.9) | 1.3 (1.2-1.4) | 443.5 (331.6-580.4) | 1.6 (1.2-2.1) | -0.22 (-0.94-0.51) |
| Acute myeloid leukemia | Bosnia and Herzegovina | 60.8 (46-86.5) | 1.5 (1.1-2.1) | 87.5 (56.8-121.7) | 1.5 (1-2.1) | -0.03 (-0.73-0.67) |
| Acute myeloid leukemia | Brunei Darussalam | 4.9 (3.7-7.3) | 2.9 (2.2-4.1) | 8.7 (6.5-11.8) | 2.2 (1.6-2.9) | -0.39 (-1.21-0.45) |
| Acute myeloid leukemia | Burkina Faso | 22.9 (10.9-45.1) | 0.3 (0.2-0.5) | 61.4 (27.6-92.2) | 0.4 (0.2-0.5) | 0.26 (-0.12-0.65) |
| Acute myeloid leukemia | Canada | 722.3 (675.4-764.8) | 2.3 (2.1-2.4) | 1721.5 (1546.8-1884.1) | 2.4 (2.2-2.6) | -0.6 (-1.44-0.24) |
| Acute myeloid leukemia | Central African Republic | 14.2 (6.7-29.4) | 0.8 (0.4-1.3) | 24.8 (13-40.4) | 0.7 (0.4-1.1) | -0.06 (-0.65-0.54) |
| Acute myeloid leukemia | Commonwealth of Dominica | 1.1 (0.9-1.5) | 1.8 (1.4-2.4) | 1.4 (1-1.9) | 1.8 (1.3-2.5) | 0.07 (-0.69-0.83) |
| Acute myeloid leukemia | Commonwealth of the Bahamas | 2.4 (2.2-2.7) | 1.1 (1-1.3) | 5.6 (4.4-7) | 1.4 (1.1-1.7) | -0.14 (-0.76-0.5) |
| Acute myeloid leukemia | Cook Islands | 0.1 (0.1-0.2) | 0.9 (0.5-1.2) | 0.2 (0.1-0.2) | 0.7 (0.4-0.9) | -0.42 (-0.88-0.03) |
| Acute myeloid leukemia | Czech Republic | 292.9 (273.1-317.5) | 2.2 (2.1-2.4) | 430.8 (365.9-505.9) | 2.1 (1.7-2.4) | -0.7 (-1.46-0.08) |
| Acute myeloid leukemia | Democratic People's Republic of Korea | 301.2 (188.5-495.7) | 1.6 (1-2.6) | 382.2 (241.2-682.8) | 1.3 (0.8-2.3) | -0.24 (-1.03-0.56) |
| Acute myeloid leukemia | Democratic Republic of Sao Tome and Principe | 0.3 (0.1-0.4) | 0.2 (0.2-0.3) | 0.3 (0.2-0.5) | 0.2 (0.1-0.4) | -0.1 (-0.38-0.18) |
| Acute myeloid leukemia | Democratic Republic of the Congo | 163.5 (78.8-282.3) | 0.7 (0.3-1) | 330.1 (186.4-469.5) | 0.6 (0.4-0.9) | 0.02 (-0.51-0.54) |
| Acute myeloid leukemia | Democratic Republic of Timor-Leste | 9 (4.1-17.1) | 1.9 (0.9-3) | 19.4 (12.6-27) | 1.9 (1.2-2.6) | -0.3 (-1.08-0.49) |
| Acute myeloid leukemia | Democratic Socialist Republic of Sri Lanka | 230.2 (147.2-312.6) | 1.7 (1.2-2.4) | 322.2 (195.5-453.7) | 1.3 (0.8-1.8) | -0.77 (-1.55-0.01) |
| Acute myeloid leukemia | Dominican Republic | 73.8 (51.8-106.9) | 1.2 (0.8-1.8) | 127.7 (90.7-194.7) | 1.2 (0.9-1.9) | -0.06 (-0.7-0.57) |
| Acute myeloid leukemia | Eastern Republic of Uruguay | 70.6 (64.5-77.1) | 2 (1.8-2.2) | 106.7 (94.6-118.9) | 2.2 (2-2.5) | -0.36 (-1.13-0.42) |
| Acute myeloid leukemia | Federal Democratic Republic of Ethiopia | 455.8 (185.1-931.9) | 1.3 (0.5-2.2) | 731.2 (391.9-1161.5) | 1 (0.6-1.7) | -1.11 (-2.01--0.19) |
| Acute myeloid leukemia | Federal Democratic Republic of Nepal | 134.6 (65.9-262.6) | 0.9 (0.5-1.6) | 241.3 (163.5-371.2) | 0.9 (0.6-1.4) | -0.23 (-0.85-0.4) |
| Acute myeloid leukemia | Federal Republic of Germany | 2352.1 (2127.4-2576.7) | 2 (1.9-2.2) | 4944 (4380.3-5434.7) | 2.6 (2.3-2.8) | -0.55 (-1.36-0.27) |
| Acute myeloid leukemia | Federal Republic of Nigeria | 213.9 (108.9-420.1) | 0.3 (0.2-0.5) | 456.5 (255.4-617.8) | 0.3 (0.2-0.4) | -0.02 (-0.38-0.34) |
| Acute myeloid leukemia | Federal Republic of Somalia | 24.5 (9-53.7) | 0.5 (0.2-0.9) | 57.3 (24.4-101.8) | 0.5 (0.2-0.8) | 0.19 (-0.39-0.77) |
| Acute myeloid leukemia | Federated States of Micronesia | 1.7 (0.9-2.6) | 2.3 (1.1-3.4) | 1.8 (1-2.6) | 2.1 (1.1-3.1) | -0.28 (-1.15-0.59) |
| Acute myeloid leukemia | Federative Republic of Brazil | 2061.9 (1990.6-2135.3) | 1.8 (1.7-1.9) | 4490.7 (4175.1-4722) | 1.9 (1.7-2) | -0.3 (-1.02-0.43) |
| Acute myeloid leukemia | French Republic | 1647.6 (1539.2-1767.7) | 2.1 (2-2.3) | 3487.8 (2964.5-3905.6) | 2.5 (2.2-2.7) | -0.61 (-1.42-0.2) |
| Acute myeloid leukemia | Gabonese Republic | 6 (3.3-8.4) | 0.9 (0.5-1.2) | 10.1 (5.9-14.9) | 0.8 (0.5-1.1) | -0.02 (-0.6-0.57) |
| Acute myeloid leukemia | Georgia | 70.4 (57.5-89.9) | 1.2 (1-1.6) | 91.7 (80.1-105.7) | 1.9 (1.6-2.1) | -0.01 (-0.71-0.7) |
| Acute myeloid leukemia | Grand Duchy of Luxembourg | 15.5 (14.6-16.5) | 3.1 (2.9-3.2) | 33.5 (30-37) | 3.2 (2.9-3.5) | -0.95 (-1.9-0) |
| Acute myeloid leukemia | Greenland | 0.7 (0.4-0.8) | 1.9 (1.3-2.3) | 0.8 (0.5-1.1) | 1.3 (0.7-1.8) | -0.64 (-1.29-0.03) |
| Acute myeloid leukemia | Grenada | 0.7 (0.6-0.9) | 0.9 (0.7-1.1) | 1.2 (1-1.4) | 1.1 (1-1.3) | -0.03 (-0.68-0.62) |
| Acute myeloid leukemia | Guam | 1.6 (1.2-2.2) | 1.7 (1.3-2.3) | 3.1 (2-3.9) | 1.7 (1.1-2.1) | -0.36 (-1.11-0.38) |
| Acute myeloid leukemia | Hashemite Kingdom of Jordan | 106.4 (79.9-141.7) | 5 (3.8-6.7) | 305.6 (216.2-406) | 3.5 (2.5-4.7) | -0.79 (-1.83-0.26) |
| Acute myeloid leukemia | Hellenic Republic | 315.2 (297-335.4) | 2.2 (2.1-2.3) | 740.8 (670.3-795.9) | 3.2 (2.9-3.4) | -0.69 (-1.52-0.14) |
| Acute myeloid leukemia | Hungary | 284.6 (265.7-304.2) | 2.1 (1.9-2.2) | 397.8 (335.4-475) | 2.2 (1.9-2.7) | -0.82 (-1.6--0.03) |
| Acute myeloid leukemia | Independent State of Papua New Guinea | 49 (18.3-75.4) | 1.6 (0.6-2.6) | 125 (60-192.9) | 1.6 (0.7-2.6) | -0.21 (-0.95-0.53) |
| Acute myeloid leukemia | Independent State of Samoa | 3 (1.8-4.3) | 2.6 (1.4-3.6) | 4 (2.4-5.6) | 2.4 (1.4-3.4) | -0.24 (-1.16-0.68) |
| Acute myeloid leukemia | Ireland | 80 (75.2-85.1) | 2 (1.8-2.1) | 142.1 (127.4-157.8) | 1.8 (1.7-2) | -1.01 (-1.81--0.19) |
| Acute myeloid leukemia | Islamic Republic of Afghanistan | 281.1 (120.2-573.9) | 3.7 (1.6-7.1) | 597.1 (300.5-1092.2) | 3.9 (1.9-6.9) | -0.2 (-1.14-0.75) |
| Acute myeloid leukemia | Islamic Republic of Iran | 1021.2 (608-1330.1) | 2.7 (1.7-3.4) | 1660.2 (1114.6-2159.4) | 2.1 (1.4-2.7) | -0.61 (-1.39-0.17) |
| Acute myeloid leukemia | Islamic Republic of Mauritania | 4.6 (2.7-8.3) | 0.3 (0.2-0.5) | 10.1 (5.7-14.2) | 0.3 (0.2-0.5) | 0.12 (-0.24-0.47) |
| Acute myeloid leukemia | Islamic Republic of Pakistan | 892 (554.6-1476.8) | 1 (0.6-1.6) | 2039.4 (1399.6-3208.8) | 1.2 (0.8-1.8) | -0.15 (-0.86-0.56) |
| Acute myeloid leukemia | Jamaica | 12.6 (10.9-15.1) | 0.6 (0.5-0.7) | 31.1 (23.7-40.3) | 1 (0.8-1.3) | 0.12 (-0.59-0.83) |
| Acute myeloid leukemia | Japan | 3019.9 (2906.3-3088.7) | 1.9 (1.9-2) | 5578.7 (4835.2-6001) | 1.6 (1.5-1.7) | -0.53 (-1.28-0.23) |
| Acute myeloid leukemia | Kingdom of Bahrain | 6.7 (4.4-9) | 2.9 (1.7-3.8) | 18.3 (11.8-28.2) | 2 (1.2-2.8) | -1.23 (-2.22--0.23) |
| Acute myeloid leukemia | Kingdom of Belgium | 333.9 (300.4-367.7) | 2.3 (2.1-2.5) | 646.2 (557.8-729.6) | 2.8 (2.5-3.1) | -0.78 (-1.6-0.05) |
| Acute myeloid leukemia | Kingdom of Bhutan | 4.2 (1.9-7.7) | 1 (0.5-1.6) | 6.4 (3.9-10.8) | 1 (0.6-1.7) | -0.34 (-0.99-0.32) |
| Acute myeloid leukemia | Kingdom of Cambodia | 171.7 (86.7-332.8) | 2.6 (1.3-4.3) | 347.7 (207.8-490.1) | 2.5 (1.6-3.5) | -0.48 (-1.35-0.4) |
| Acute myeloid leukemia | Kingdom of Denmark | 246.3 (230.3-263.5) | 3.3 (3.1-3.5) | 304.5 (269.8-343.2) | 2.6 (2.3-2.9) | -0.74 (-1.6-0.13) |
| Acute myeloid leukemia | Kingdom of Eswatini | 5.6 (3.6-9.6) | 1.4 (0.9-2.3) | 11.7 (6.7-20.4) | 1.6 (1-2.7) | 0.34 (-0.51-1.21) |
| Acute myeloid leukemia | Kingdom of Lesotho | 9.8 (6.3-16) | 1 (0.6-1.6) | 20.2 (12.8-36.5) | 1.6 (1-2.7) | 0.94 (0.14-1.74) |
| Acute myeloid leukemia | Kingdom of Morocco | 90.5 (60-145) | 0.5 (0.3-0.8) | 178.5 (118.5-288.1) | 0.5 (0.3-0.8) | -0.08 (-0.46-0.29) |
| Acute myeloid leukemia | Kingdom of Norway | 155.9 (147.2-163.3) | 2.5 (2.3-2.6) | 230.5 (207.9-249.2) | 2.3 (2.1-2.5) | -0.33 (-1.13-0.48) |
| Acute myeloid leukemia | Kingdom of Saudi Arabia | 81.1 (52.1-137.1) | 0.8 (0.6-1.5) | 310.8 (207.3-545.5) | 1.1 (0.8-1.8) | 0.26 (-0.34-0.87) |
| Acute myeloid leukemia | Kingdom of Spain | 799.7 (748.2-848.1) | 1.6 (1.5-1.7) | 1822.8 (1581.7-2045.8) | 1.9 (1.7-2.1) | -0.8 (-1.53--0.07) |
| Acute myeloid leukemia | Kingdom of Sweden | 284.1 (261.8-307.3) | 2 (1.9-2.2) | 444.4 (380.4-500.8) | 2 (1.7-2.3) | -0.55 (-1.35-0.26) |
| Acute myeloid leukemia | Kingdom of Thailand | 1056.3 (738.5-1411.6) | 2.5 (1.7-3.3) | 2912.1 (1319.4-4091.3) | 3.1 (1.5-4.3) | -0.59 (-1.45-0.27) |
| Acute myeloid leukemia | Kingdom of the Netherlands | 437.7 (404.4-467.6) | 2.3 (2.1-2.4) | 824 (728.3-922.7) | 2.4 (2.1-2.6) | -0.65 (-1.48-0.19) |
| Acute myeloid leukemia | Kingdom of Tonga | 1.2 (0.7-1.6) | 1.7 (0.9-2.2) | 1.7 (1-2.3) | 1.8 (1-2.5) | -0.13 (-0.88-0.62) |
| Acute myeloid leukemia | Kyrgyz Republic | 32.4 (26.8-40.1) | 0.8 (0.6-0.9) | 60.6 (50-73.1) | 1 (0.8-1.2) | -0.67 (-1.25--0.1) |
| Acute myeloid leukemia | Lao People's Democratic Republic | 70.3 (30.9-160.4) | 2.5 (1.2-4.7) | 124.2 (79.8-177.7) | 2.2 (1.4-3.2) | -0.77 (-1.63-0.1) |
| Acute myeloid leukemia | Lebanese Republic | 66 (44-111.5) | 2.9 (2-4.7) | 148.6 (107.7-200.6) | 2.4 (1.8-3.3) | -0.36 (-1.17-0.46) |
| Acute myeloid leukemia | Malaysia | 235.5 (145.1-308.4) | 1.9 (1.2-2.5) | 528.9 (370.3-739.3) | 1.8 (1.3-2.6) | -0.31 (-1.14-0.53) |
| Acute myeloid leukemia | Mongolia | 21.8 (13.2-36.2) | 1.3 (0.8-2) | 36.3 (25-48.5) | 1.2 (0.9-1.6) | -0.43 (-1.11-0.26) |
| Acute myeloid leukemia | Montenegro | 10.9 (8.2-15.1) | 1.8 (1.3-2.4) | 16.2 (11.4-23.1) | 1.8 (1.3-2.5) | -0.12 (-0.87-0.64) |
| Acute myeloid leukemia | New Zealand | 103.7 (93.7-112.1) | 2.7 (2.4-2.9) | 167.6 (147.9-185.7) | 2.1 (1.9-2.3) | -0.58 (-1.44-0.28) |
| Acute myeloid leukemia | North Macedonia | 44.2 (31.9-58.3) | 2.4 (1.7-3.2) | 65.3 (40.1-87.5) | 2.1 (1.3-2.8) | -0.23 (-1.25-0.79) |
| Acute myeloid leukemia | Northern Mariana Islands | 0.6 (0.3-0.8) | 2.1 (1.1-2.9) | 0.4 (0.2-0.9) | 0.9 (0.6-2) | -0.52 (-1.28-0.24) |
| Acute myeloid leukemia | Palestine | 11.9 (7.1-20) | 0.9 (0.5-1.4) | 24.9 (15.9-37.1) | 0.7 (0.4-1.1) | -0.8 (-1.71-0.11) |
| Acute myeloid leukemia | People's Democratic Republic of Algeria | 204.1 (126.6-281.2) | 1.1 (0.6-1.5) | 321.1 (205.3-502.5) | 0.9 (0.6-1.3) | -0.28 (-0.92-0.36) |
| Acute myeloid leukemia | People's Republic of Bangladesh | 865.5 (460.1-1711.4) | 1 (0.6-1.7) | 1297.7 (815-1920.1) | 0.9 (0.6-1.3) | -0.59 (-1.24-0.06) |
| Acute myeloid leukemia | People's Republic of China | 14853.1 (8014.1-23401.9) | 1.4 (0.8-2.2) | 15311.1 (10365.3-21401.3) | 0.9 (0.6-1.2) | -1.08 (-1.87--0.29) |
| Acute myeloid leukemia | Plurinational State of Bolivia | 98.7 (58.3-185.7) | 2 (1.2-3.5) | 207.8 (129.2-298.2) | 2.1 (1.3-3) | -0.42 (-1.3-0.48) |
| Acute myeloid leukemia | Portuguese Republic | 241.9 (224.4-259.2) | 1.9 (1.8-2.1) | 521.6 (453.2-579.3) | 2.2 (2-2.4) | -0.79 (-1.59-0.01) |
| Acute myeloid leukemia | Principality of Andorra | 1.9 (1.2-2.9) | 3.3 (2.1-5.2) | 3.3 (1.9-4.8) | 2.3 (1.3-3.3) | -0.9 (-1.78--0.01) |
| Acute myeloid leukemia | Principality of Monaco | 2.5 (1.8-3.7) | 4.1 (2.9-5.9) | 4.7 (2.8-6.3) | 5.3 (3.4-7) | 0.1 (-0.95-1.17) |
| Acute myeloid leukemia | Puerto Rico | 83.5 (77.4-90.5) | 2.3 (2.1-2.5) | 138.8 (113-165.3) | 2.3 (1.9-2.7) | -0.7 (-1.46-0.06) |
| Acute myeloid leukemia | Republic of Albania | 38.6 (27.1-58.7) | 1.6 (1.1-2.5) | 52.9 (33.5-79) | 1.3 (0.9-2) | -0.39 (-1.07-0.3) |
| Acute myeloid leukemia | Republic of Angola | 50 (22.4-104.2) | 0.8 (0.4-1.2) | 134.2 (72.2-201.7) | 0.7 (0.4-1.1) | -0.09 (-0.64-0.47) |
| Acute myeloid leukemia | Republic of Armenia | 28.6 (22.6-35.2) | 0.9 (0.7-1.1) | 49.8 (41.2-60) | 1.3 (1.1-1.5) | -0.62 (-1.3-0.06) |
| Acute myeloid leukemia | Republic of Austria | 209 (193-224.6) | 1.9 (1.8-2) | 399 (353.7-442.3) | 2.2 (2-2.4) | -0.49 (-1.3-0.34) |
| Acute myeloid leukemia | Republic of Azerbaijan | 102.9 (71-158.1) | 1.5 (1.1-2.3) | 135.8 (91.6-216.2) | 1.3 (0.9-2) | -0.64 (-1.38-0.1) |
| Acute myeloid leukemia | Republic of Belarus | 137 (112.9-174) | 1.2 (1-1.5) | 248.6 (199.9-301.6) | 1.8 (1.5-2.2) | -0.69 (-1.4-0.03) |
| Acute myeloid leukemia | Republic of Benin | 12 (5.9-24.6) | 0.3 (0.2-0.5) | 33 (15.8-46.4) | 0.3 (0.2-0.5) | 0.2 (-0.18-0.58) |
| Acute myeloid leukemia | Republic of Botswana | 9 (5.4-14.3) | 1.3 (0.8-1.9) | 20.1 (11.6-29.9) | 1.2 (0.7-1.7) | -0.07 (-0.81-0.67) |
| Acute myeloid leukemia | Republic of Bulgaria | 157.3 (144.9-171.5) | 1.5 (1.3-1.6) | 301.6 (242.9-368.7) | 2.5 (2-3) | 0.05 (-0.67-0.78) |
| Acute myeloid leukemia | Republic of Burundi | 24.9 (9.9-44.5) | 0.6 (0.2-0.9) | 43.1 (17.9-69.6) | 0.5 (0.2-0.8) | -0.13 (-0.68-0.42) |
| Acute myeloid leukemia | Republic of Cabo Verde | 2.3 (1.7-4.2) | 0.7 (0.5-1.3) | 4.2 (2.8-7.1) | 0.9 (0.6-1.5) | 0.14 (-0.46-0.75) |
| Acute myeloid leukemia | Republic of Cameroon | 26.5 (14.3-50.7) | 0.3 (0.2-0.6) | 81.4 (41.7-118.5) | 0.4 (0.2-0.6) | 0.23 (-0.18-0.64) |
| Acute myeloid leukemia | Republic of Chad | 11.8 (5.8-25.4) | 0.2 (0.1-0.4) | 40.5 (17.2-67.7) | 0.4 (0.2-0.5) | 0.39 (0.02-0.77) |
| Acute myeloid leukemia | Republic of Chile | 166.2 (155.6-176.9) | 1.4 (1.3-1.6) | 361.9 (328.2-394.8) | 1.5 (1.4-1.6) | -0.41 (-1.09-0.27) |
| Acute myeloid leukemia | Republic of Colombia | 327.1 (305.9-349.3) | 1.3 (1.2-1.4) | 837.8 (692.6-985) | 1.6 (1.3-1.9) | -0.44 (-1.19-0.31) |
| Acute myeloid leukemia | Republic of Costa Rica | 37.7 (34.9-41.9) | 1.7 (1.5-1.9) | 119.8 (105.6-134.3) | 2.3 (2-2.5) | -0.11 (-0.88-0.68) |
| Acute myeloid leukemia | République de Côte d’Ivoire | 20 (10.1-32.5) | 0.3 (0.1-0.4) | 41.7 (23.2-62.1) | 0.2 (0.1-0.3) | -0.02 (-0.32-0.28) |
| Acute myeloid leukemia | Republic of Croatia | 92.6 (77.9-109.2) | 1.6 (1.3-1.9) | 183 (147.4-223) | 2.2 (1.8-2.6) | -0.54 (-1.3-0.23) |
| Acute myeloid leukemia | Republic of Cuba | 170.8 (159.9-185.7) | 1.6 (1.5-1.7) | 257.9 (219.8-296.8) | 1.6 (1.3-1.8) | -0.44 (-1.14-0.26) |
| Acute myeloid leukemia | Republic of Cyprus | 25 (18.7-38.4) | 3.3 (2.5-5.3) | 51.7 (31.8-67.7) | 2.6 (1.6-3.3) | -0.67 (-1.65-0.33) |
| Acute myeloid leukemia | Republic of Djibouti | 1.5 (0.7-2.4) | 0.5 (0.2-0.8) | 4.9 (2.4-8.1) | 0.5 (0.3-0.9) | 0.21 (-0.35-0.77) |
| Acute myeloid leukemia | Republic of Ecuador | 98.2 (90.6-108.1) | 1.3 (1.2-1.5) | 305.5 (243.4-377) | 1.8 (1.4-2.2) | 0.33 (-0.52-1.18) |
| Acute myeloid leukemia | Republic of El Salvador | 56.8 (37.7-88.9) | 1.2 (0.9-1.9) | 92.6 (56.8-121.4) | 1.5 (0.9-1.9) | -0.02 (-0.83-0.79) |
| Acute myeloid leukemia | Republic of Equatorial Guinea | 2.1 (1-3.9) | 0.8 (0.4-1.2) | 6.2 (3.1-9.9) | 0.7 (0.4-1.1) | -0.19 (-0.72-0.35) |
| Acute myeloid leukemia | Republic of Estonia | 25.6 (22.4-29) | 1.5 (1.3-1.7) | 41.9 (34.6-49.7) | 1.9 (1.6-2.2) | -1 (-1.8--0.2) |
| Acute myeloid leukemia | Republic of Fiji | 22.8 (13.9-29.9) | 4.5 (2.7-5.9) | 33.3 (18.1-45.8) | 4.3 (2.3-5.8) | -0.32 (-1.45-0.82) |
| Acute myeloid leukemia | Republic of Finland | 116.2 (106.5-125.8) | 1.7 (1.6-1.9) | 189.4 (165-214) | 1.6 (1.4-1.8) | -0.61 (-1.32-0.11) |
| Acute myeloid leukemia | Republic of Ghana | 70.1 (32.6-100.6) | 0.6 (0.3-0.9) | 95.1 (60.7-167.8) | 0.4 (0.2-0.7) | -0.75 (-1.24--0.27) |
| Acute myeloid leukemia | Republic of Guatemala | 65.2 (52.5-76.2) | 0.9 (0.7-1) | 143.4 (121.8-165.6) | 1.1 (0.9-1.2) | -0.01 (-0.78-0.77) |
| Acute myeloid leukemia | Republic of Guinea | 6.9 (3-14.7) | 0.1 (0.1-0.2) | 10.7 (5.6-17.5) | 0.1 (0.1-0.2) | -0.02 (-0.21-0.16) |
| Acute myeloid leukemia | Republic of Guinea-Bissau | 2.7 (1.4-6) | 0.4 (0.2-0.7) | 5.3 (2.8-7.3) | 0.4 (0.2-0.6) | 0.24 (-0.2-0.68) |
| Acute myeloid leukemia | Republic of Guyana | 1.6 (1.3-1.9) | 0.3 (0.2-0.3) | 2.8 (2.1-3.6) | 0.4 (0.3-0.5) | 0.13 (-0.52-0.78) |
| Acute myeloid leukemia | Republic of Haiti | 85.8 (42.5-182.5) | 1.6 (0.9-3) | 155 (85.6-251.3) | 1.5 (0.9-2.3) | -0.34 (-1.08-0.41) |
| Acute myeloid leukemia | Republic of Honduras | 52.3 (29.2-82.5) | 1.4 (0.9-2.1) | 114.3 (70.4-173.9) | 1.5 (1-2.3) | -0.13 (-0.92-0.67) |
| Acute myeloid leukemia | Republic of Iceland | 5.7 (5.1-6.2) | 2 (1.8-2.2) | 12.7 (11-14.5) | 2.3 (2-2.5) | -0.35 (-1.12-0.43) |
| Acute myeloid leukemia | Republic of India | 5249.8 (3288.7-8407.7) | 0.8 (0.5-1.2) | 10981.1 (8158.9-15119.9) | 0.9 (0.7-1.2) | -0.31 (-0.92-0.29) |
| Acute myeloid leukemia | Republic of Indonesia | 2737.5 (1784.1-4170.4) | 2.1 (1.4-3) | 5453.2 (3927.9-7455.9) | 2.2 (1.6-3) | -0.16 (-0.99-0.69) |
| Acute myeloid leukemia | Republic of Iraq | 219.4 (115.9-499.4) | 1.8 (0.9-3.7) | 514.4 (325-939.4) | 1.7 (1.1-3.2) | -0.36 (-1.22-0.5) |
| Acute myeloid leukemia | Republic of Italy | 1496.3 (1410.7-1560.5) | 1.9 (1.8-2) | 3522.5 (3097.1-3803.9) | 2.5 (2.2-2.6) | -0.84 (-1.62--0.04) |
| Acute myeloid leukemia | Republic of Kazakhstan | 223.2 (185.6-274.5) | 1.5 (1.2-1.8) | 209.9 (171.5-250.9) | 1.1 (0.9-1.3) | -0.54 (-1.21-0.12) |
| Acute myeloid leukemia | Republic of Kenya | 57.6 (33.5-85.3) | 0.4 (0.2-0.6) | 157.8 (105.5-218.9) | 0.5 (0.3-0.7) | 0.29 (-0.18-0.76) |
| Acute myeloid leukemia | Republic of Kiribati | 0.8 (0.3-1.4) | 1.4 (0.5-2.2) | 1.4 (0.6-2.3) | 1.4 (0.6-2.3) | -0.04 (-0.76-0.68) |
| Acute myeloid leukemia | Republic of Korea | 780.2 (548.8-1097.8) | 2.1 (1.6-3.2) | 1357.3 (656.2-1722) | 1.6 (0.8-2) | -1 (-1.72--0.27) |
| Acute myeloid leukemia | Republic of Latvia | 66.8 (58.6-76.9) | 2.2 (1.9-2.5) | 53 (44.3-62.5) | 1.7 (1.4-1.9) | -0.81 (-1.58--0.03) |
| Acute myeloid leukemia | Republic of Liberia | 6.2 (2.8-14.6) | 0.3 (0.2-0.5) | 12.1 (5.7-17.6) | 0.4 (0.2-0.5) | 0.23 (-0.14-0.6) |
| Acute myeloid leukemia | Republic of Lithuania | 52.2 (46.1-59.1) | 1.3 (1.1-1.5) | 107.3 (90.2-123.9) | 2.2 (1.9-2.6) | -0.54 (-1.31-0.25) |
| Acute myeloid leukemia | Republic of Madagascar | 44.5 (20.8-66) | 0.5 (0.2-0.7) | 85 (41.8-120.9) | 0.4 (0.2-0.6) | 0.01 (-0.48-0.5) |
| Acute myeloid leukemia | Republic of Malawi | 17.1 (5.4-31.5) | 0.2 (0.1-0.3) | 22.3 (10.4-38.7) | 0.2 (0.1-0.2) | -0.09 (-0.38-0.2) |
| Acute myeloid leukemia | Republic of Maldives | 2.6 (1.5-5.2) | 2.1 (1.3-3.6) | 4.9 (3.1-7.1) | 1.3 (0.9-1.8) | -1.16 (-1.89--0.42) |
| Acute myeloid leukemia | Republic of Mali | 20 (8-40.2) | 0.3 (0.1-0.4) | 36.7 (20-57.4) | 0.2 (0.1-0.3) | -0.11 (-0.42-0.2) |
| Acute myeloid leukemia | Republic of Malta | 8.6 (7.8-9.5) | 2.1 (1.9-2.3) | 22.1 (19.4-24.9) | 2.4 (2.1-2.7) | -0.66 (-1.46-0.15) |
| Acute myeloid leukemia | Republic of Mauritius | 6 (5.5-6.6) | 0.7 (0.6-0.7) | 14.9 (13.6-16.1) | 0.9 (0.8-1) | -0.51 (-1.24-0.22) |
| Acute myeloid leukemia | Republic of Moldova | 76.3 (69.3-89.1) | 1.7 (1.6-2) | 68.2 (61-77.1) | 1.3 (1.1-1.5) | -0.74 (-1.42--0.06) |
| Acute myeloid leukemia | Republic of Mozambique | 99.8 (34-187.6) | 0.8 (0.3-1.3) | 170.6 (84.9-293.2) | 0.8 (0.4-1.2) | 0.14 (-0.52-0.8) |
| Acute myeloid leukemia | Republic of Namibia | 7.8 (5.1-11.6) | 0.9 (0.6-1.4) | 15.9 (10.3-24.4) | 0.9 (0.6-1.5) | -0.03 (-0.65-0.6) |
| Acute myeloid leukemia | Republic of Nauru | 0.2 (0.1-0.3) | 2.7 (1.4-4) | 0.2 (0.1-0.3) | 2.4 (1.3-3.7) | -0.22 (-1.15-0.73) |
| Acute myeloid leukemia | Republic of Nicaragua | 29.2 (18.2-45.3) | 0.9 (0.7-1.3) | 50.6 (33-67) | 0.9 (0.6-1.2) | -0.21 (-0.94-0.53) |
| Acute myeloid leukemia | Republic of Niue | 0 (0-0.1) | 1.9 (0.9-2.7) | 0 (0-0.1) | 2.9 (1.8-3.8) | -0.06 (-0.86-0.74) |
| Acute myeloid leukemia | Republic of Palau | 0.1 (0-0.1) | 0.5 (0.2-0.7) | 0.1 (0-0.1) | 0.4 (0.2-0.6) | -0.16 (-0.66-0.36) |
| Acute myeloid leukemia | Republic of Panama | 25.8 (23.6-28.5) | 1.3 (1.2-1.4) | 76.2 (60.9-91.9) | 1.8 (1.4-2.1) | 0.06 (-0.7-0.83) |
| Acute myeloid leukemia | Republic of Paraguay | 37.1 (26.3-52.5) | 1.2 (0.9-1.7) | 99.3 (66-134.6) | 1.6 (1.1-2.2) | 0.24 (-0.47-0.96) |
| Acute myeloid leukemia | Republic of Peru | 253.9 (169.7-379.5) | 1.5 (1-2.2) | 523.9 (298.4-718.4) | 1.5 (0.9-2.1) | -0.18 (-0.97-0.62) |
| Acute myeloid leukemia | Republic of Poland | 766.3 (695.1-890.2) | 1.8 (1.7-2.1) | 1287.7 (1171.9-1399.6) | 1.9 (1.7-2.1) | -0.79 (-1.55--0.02) |
| Acute myeloid leukemia | Republic of Rwanda | 37.2 (16.1-69.8) | 0.7 (0.3-1.1) | 52 (26.6-79.9) | 0.5 (0.3-0.8) | -0.5 (-1.08-0.08) |
| Acute myeloid leukemia | Republic of San Marino | 0.6 (0.5-1) | 1.9 (1.3-2.9) | 0.7 (0.4-1.1) | 1.1 (0.6-1.7) | -0.78 (-1.6-0.04) |
| Acute myeloid leukemia | Republic of Senegal | 18.3 (9.5-33.9) | 0.3 (0.2-0.5) | 40.1 (22.2-56.5) | 0.4 (0.2-0.5) | 0.23 (-0.16-0.61) |
| Acute myeloid leukemia | Republic of Serbia | 209.8 (151.9-282.5) | 2 (1.5-2.8) | 294 (184.7-377.8) | 1.9 (1.2-2.4) | -0.51 (-1.26-0.25) |
| Acute myeloid leukemia | Republic of Seychelles | 1.1 (0.7-1.4) | 1.7 (1.1-2.2) | 1.5 (0.9-2) | 1.3 (0.8-1.8) | -0.43 (-1.15-0.29) |
| Acute myeloid leukemia | Republic of Sierra Leone | 10.1 (4.8-23.3) | 0.3 (0.1-0.5) | 21.4 (10.1-30.6) | 0.3 (0.2-0.5) | 0.26 (-0.11-0.64) |
| Acute myeloid leukemia | Republic of Singapore | 56.4 (52.8-59.9) | 2.3 (2.1-2.5) | 127.4 (116.2-138.1) | 1.6 (1.5-1.8) | -0.85 (-1.66--0.04) |
| Acute myeloid leukemia | Republic of Slovenia | 29.4 (26.2-33) | 1.2 (1.1-1.4) | 58.6 (48-69.6) | 1.4 (1.1-1.6) | -0.5 (-1.23-0.23) |
| Acute myeloid leukemia | Republic of South Africa | 339.1 (211.1-469.9) | 1.3 (0.8-1.8) | 665.1 (433.6-898) | 1.4 (0.9-1.8) | 0.09 (-0.68-0.86) |
| Acute myeloid leukemia | Republic of South Sudan | 24.7 (10.1-46.1) | 0.6 (0.2-1) | 37.7 (16.6-62.9) | 0.6 (0.3-0.9) | 0.3 (-0.25-0.86) |
| Acute myeloid leukemia | Republic of Sudan | 337.3 (165.3-653.8) | 2.5 (1.3-4.3) | 690.4 (408-1076.8) | 2.5 (1.5-3.8) | -0.33 (-1.16-0.51) |
| Acute myeloid leukemia | Republic of Suriname | 3.7 (2.5-5) | 1.1 (0.8-1.6) | 7.1 (4.8-9.9) | 1.2 (0.8-1.6) | -0.16 (-0.77-0.45) |
| Acute myeloid leukemia | Republic of Tajikistan | 55.9 (33.8-82.6) | 1.2 (0.7-1.6) | 84.2 (48.5-136.6) | 0.9 (0.6-1.5) | -0.75 (-1.39--0.1) |
| Acute myeloid leukemia | Republic of the Congo | 13.2 (8-22.3) | 0.9 (0.5-1.3) | 27.5 (16.3-38.9) | 0.8 (0.4-1.1) | -0.14 (-0.73-0.45) |
| Acute myeloid leukemia | Republic of the Gambia | 1 (0.5-2) | 0.2 (0.1-0.2) | 2.4 (1.5-3.9) | 0.2 (0.1-0.3) | -0.02 (-0.24-0.2) |
| Acute myeloid leukemia | Republic of the Marshall Islands | 0.6 (0.3-0.8) | 1.9 (0.9-2.7) | 0.9 (0.5-1.3) | 2.1 (1.1-3) | -0.05 (-0.88-0.79) |
| Acute myeloid leukemia | Republic of the Niger | 19 (7.3-47.7) | 0.3 (0.1-0.5) | 49 (19.9-75.3) | 0.3 (0.1-0.5) | 0.12 (-0.25-0.5) |
| Acute myeloid leukemia | Republic of the Philippines | 1017.5 (747.6-1413.1) | 2.4 (1.8-3) | 2020.1 (1621.9-2624.5) | 2.2 (1.8-2.9) | -0.35 (-1.19-0.49) |
| Acute myeloid leukemia | Republic of the Union of Myanmar | 807.1 (415.5-1615.1) | 2.6 (1.4-4.9) | 1119.3 (795.2-1605.3) | 2.2 (1.6-3.2) | -0.95 (-1.84--0.07) |
| Acute myeloid leukemia | Republic of Trinidad and Tobago | 16.3 (14.7-17.9) | 1.6 (1.5-1.8) | 32.1 (24.4-40.8) | 1.9 (1.4-2.4) | -0.43 (-1.12-0.27) |
| Acute myeloid leukemia | Republic of Tunisia | 86.5 (62-140.9) | 1.4 (1-2.4) | 157.5 (101.7-259.8) | 1.2 (0.8-2) | -0.48 (-1.11-0.16) |
| Acute myeloid leukemia | Republic of Turkey | 1743.6 (1119.6-2396.8) | 4 (2.7-5.2) | 2446.3 (1718.9-3192.7) | 2.7 (1.9-3.5) | -1.01 (-1.89--0.12) |
| Acute myeloid leukemia | Republic of Uganda | 48.9 (25-80.4) | 0.4 (0.2-0.6) | 131.9 (70.7-205.2) | 0.4 (0.2-0.7) | -0.04 (-0.5-0.43) |
| Acute myeloid leukemia | Republic of Uzbekistan | 246.2 (191.4-302.9) | 1.3 (1.1-1.6) | 354.4 (290.2-439.6) | 1.1 (0.9-1.4) | -0.68 (-1.33--0.03) |
| Acute myeloid leukemia | Republic of Vanuatu | 1.7 (0.8-2.5) | 1.7 (0.7-2.5) | 4.1 (2.2-5.7) | 1.8 (0.9-2.5) | -0.13 (-0.91-0.65) |
| Acute myeloid leukemia | Republic of Yemen | 166.3 (78.3-309.2) | 2.3 (1.1-3.7) | 442.1 (233.5-656.8) | 2.3 (1.2-3.5) | -0.28 (-1.07-0.51) |
| Acute myeloid leukemia | Republic of Zambia | 37 (16.7-65) | 0.6 (0.3-0.9) | 92.1 (49-139.8) | 0.7 (0.4-1) | -0.05 (-0.63-0.53) |
| Acute myeloid leukemia | Republic of Zimbabwe | 66.1 (41.6-102.1) | 1.2 (0.8-2) | 147.2 (90.8-234.7) | 1.6 (1-2.6) | 0.62 (-0.21-1.45) |
| Acute myeloid leukemia | Romania | 290.5 (263.1-321.4) | 1.2 (1-1.3) | 543.5 (469.2-619.7) | 1.7 (1.4-1.9) | -0.11 (-0.79-0.57) |
| Acute myeloid leukemia | Russian Federation | 2097.5 (1897.9-2294.8) | 1.3 (1.2-1.4) | 2681.2 (2460.1-2906.2) | 1.3 (1.2-1.4) | -0.44 (-1.09-0.21) |
| Acute myeloid leukemia | Saint Kitts and Nevis | 0.1 (0.1-0.1) | 0.2 (0.2-0.3) | 0.2 (0.1-0.2) | 0.3 (0.2-0.3) | -0.42 (-1.11-0.28) |
| Acute myeloid leukemia | Saint Lucia | 1.3 (1.2-1.5) | 1.2 (1.1-1.4) | 2.5 (2-3) | 1.2 (1-1.4) | -0.73 (-1.34--0.11) |
| Acute myeloid leukemia | Saint Vincent and the Grenadines | 1.1 (1-1.2) | 1 (0.9-1.1) | 1.4 (1.2-1.6) | 1.1 (1-1.3) | -0.29 (-0.95-0.37) |
| Acute myeloid leukemia | Slovak Republic | 129.3 (101.8-190.3) | 2.2 (1.7-3.3) | 163.3 (93.8-220.1) | 1.9 (1.1-2.5) | -0.52 (-1.32-0.29) |
| Acute myeloid leukemia | Socialist Republic of Viet Nam | 515.4 (298.2-750) | 1.1 (0.6-1.6) | 995.9 (657-1426.1) | 1 (0.7-1.5) | -0.39 (-1.08-0.3) |
| Acute myeloid leukemia | Solomon Islands | 4.3 (1.8-6.5) | 1.9 (0.7-2.8) | 10.1 (5.2-14.3) | 2 (1-2.9) | -0.08 (-0.9-0.74) |
| Acute myeloid leukemia | State of Eritrea | 11.8 (5.1-20.8) | 0.5 (0.2-0.8) | 25.9 (13.7-37) | 0.6 (0.3-0.8) | 0.18 (-0.38-0.75) |
| Acute myeloid leukemia | State of Israel | 134.4 (121.4-147.2) | 2.8 (2.5-3) | 335.6 (294.6-370.8) | 2.7 (2.4-3) | -0.73 (-1.7-0.25) |
| Acute myeloid leukemia | State of Kuwait | 13.1 (11.7-14.6) | 1.5 (1.3-1.7) | 38.6 (30.7-47) | 1.1 (0.9-1.4) | -1.06 (-1.75--0.36) |
| Acute myeloid leukemia | State of Libya | 75.2 (51.3-117.3) | 3 (2.1-4.9) | 161.8 (98.2-274.2) | 2.9 (1.8-4.7) | 0.11 (-0.77-0.99) |
| Acute myeloid leukemia | State of Qatar | 4.3 (2.6-5.9) | 2.3 (1.3-3.3) | 16.9 (11.1-25.9) | 1.3 (0.8-1.9) | -1.42 (-2.21--0.63) |
| Acute myeloid leukemia | Sultanate of Oman | 23.8 (14-33.4) | 2 (1-2.8) | 39.7 (26.3-53.6) | 1.4 (0.9-1.9) | -0.44 (-1.2-0.33) |
| Acute myeloid leukemia | Swiss Confederation | 217.5 (196.6-237.2) | 2.3 (2.1-2.4) | 350.9 (303.9-392.6) | 2 (1.8-2.2) | -0.68 (-1.44-0.08) |
| Acute myeloid leukemia | Syrian Arab Republic | 258.1 (166.5-416.3) | 3.4 (2.1-5.1) | 362.4 (239.9-569.5) | 2.7 (1.8-4.4) | -0.8 (-1.65-0.06) |
| Acute myeloid leukemia | Taiwan (Province of China) | 275.7 (242.5-322.3) | 1.6 (1.4-1.9) | 812.7 (732.7-879.7) | 2.2 (2-2.4) | 0.19 (-0.59-0.99) |
| Acute myeloid leukemia | Togolese Republic | 7 (3.7-12.3) | 0.3 (0.2-0.4) | 19.5 (10-27.5) | 0.4 (0.2-0.5) | 0.28 (-0.09-0.66) |
| Acute myeloid leukemia | Tokelau | 0 (0-0) | 2 (0.9-2.8) | 0 (0-0.1) | 3.4 (1.7-5.1) | -0.17 (-0.97-0.64) |
| Acute myeloid leukemia | Turkmenistan | 40.7 (33.5-48.1) | 1.2 (1-1.4) | 58.3 (44.2-77) | 1.2 (0.9-1.5) | -0.41 (-1.04-0.21) |
| Acute myeloid leukemia | Tuvalu | 0.2 (0.1-0.2) | 2 (0.9-2.9) | 0.2 (0.1-0.3) | 1.9 (1-2.6) | -0.34 (-1.16-0.48) |
| Acute myeloid leukemia | Ukraine | 1130.9 (965.8-1293.3) | 1.9 (1.6-2.2) | 713.6 (527.7-930.4) | 1.1 (0.9-1.5) | -1.13 (-1.8--0.46) |
| Acute myeloid leukemia | Union of the Comoros | 2 (0.8-2.9) | 0.6 (0.3-0.9) | 3.4 (1.9-5.1) | 0.6 (0.3-0.8) | 0.02 (-0.53-0.57) |
| Acute myeloid leukemia | United Arab Emirates | 26 (15.8-36.1) | 3.3 (1.8-4.8) | 118.9 (73.6-165.7) | 2.4 (1.5-3.2) | -0.09 (-0.97-0.79) |
| Acute myeloid leukemia | United Kingdom of Great Britain and Northern Ireland | 1985.5 (1914.7-2049.1) | 2.3 (2.2-2.4) | 3430.7 (3130.3-3583.4) | 2.6 (2.4-2.7) | -0.46 (-1.32-0.41) |
| Acute myeloid leukemia | United Mexican States | 944.3 (915.9-980.1) | 1.4 (1.3-1.4) | 1914.3 (1690.2-2120.3) | 1.5 (1.3-1.7) | -0.21 (-1.03-0.62) |
| Acute myeloid leukemia | United Republic of Tanzania | 119.8 (53.7-184.5) | 0.6 (0.3-0.8) | 241.5 (125.4-359.1) | 0.5 (0.3-0.8) | -0.01 (-0.56-0.55) |
| Acute myeloid leukemia | United States of America | 8954.1 (8426.5-9232.6) | 2.9 (2.7-2.9) | 16648.5 (15097-17423.6) | 2.9 (2.7-3) | -0.7 (-1.61-0.21) |
| Acute myeloid leukemia | United States Virgin Islands | 1.4 (1-1.8) | 1.4 (1-1.8) | 1.2 (0.8-1.6) | 1 (0.7-1.4) | -0.54 (-1.26-0.18) |
| Acute myeloid leukemia | African Union | 3685.37 (2205.06-6101.55) | 0.85 (0.54-1.32) | 7906.38 (4992.15-10630.25) | 0.91 (0.6-1.26) | 0.4 (0.32-0.48) |
| Acute myeloid leukemia | Association of Southeast Asian Nations | 6672.61 (4576.21-9907.99) | 2.07 (1.41-2.92) | 13637.42 (9709.83-16556.05) | 2.16 (1.55-2.62) | 0.05 (-0.01-0.12) |
| Acute myeloid leukemia | Central Europe, Eastern Europe, and Central Asia | 6854.6 (6414.02-7369.05) | 1.53 (1.43-1.64) | 8934.49 (8388.88-9532.11) | 1.54 (1.44-1.64) | 0.16 (0.08-0.24) |
| Acute myeloid leukemia | Commonwealth | 12047.52 (8600.95-17219.85) | 1.15 (0.88-1.5) | 24599.2 (20384.12-30868.69) | 1.18 (0.99-1.46) | 0.06 (0.01-0.11) |
| Acute myeloid leukemia | European Union | 10796.92 (10348.59-11230.5) | 1.95 (1.88-2.02) | 21665.12 (19632.03-23069.54) | 2.32 (2.14-2.45) | 0.81 (0.69-0.92) |
| Acute myeloid leukemia | Four World Regions | 74743.52 (58568.95-94533.76) | 1.69 (1.36-2.08) | 129898.32 (113358.3-149070.21) | 1.57 (1.37-1.8) | -0.21 (-0.29--0.14) |
| Acute myeloid leukemia | G20 | 57413.53 (46137.2-70454.66) | 1.78 (1.45-2.15) | 97019.37 (86333.15-108432.45) | 1.59 (1.41-1.78) | -0.35 (-0.45--0.26) |
| Acute myeloid leukemia | Gulf Cooperation Council | 154.95 (107.21-221.6) | 1.19 (0.84-1.69) | 543.32 (401.03-810.68) | 1.31 (0.94-1.79) | 0.62 (0.31-0.93) |
| Acute myeloid leukemia | Health System Grouping Levels | 74832.99 (58657.96-94620.05) | 1.69 (1.36-2.08) | 130044.46 (113498.83-149228.74) | 1.57 (1.37-1.8) | -0.22 (-0.29--0.14) |
| Acute myeloid leukemia | High SDI | 24617.15 (23487.31-25533.84) | 2.33 (2.22-2.41) | 47188.95 (42904.21-49837.71) | 2.31 (2.13-2.43) | 0.13 (0-0.26) |
| Acute myeloid leukemia | High-income | 26049.02 (24847.32-26801.95) | 2.29 (2.2-2.36) | 50928.55 (45840.64-53584.49) | 2.37 (2.18-2.47) | 0.26 (0.13-0.38) |
| Acute myeloid leukemia | High-middle SDI | 17930.28 (13566.19-21775.59) | 1.77 (1.35-2.15) | 26275.25 (22369.85-29543.72) | 1.49 (1.26-1.69) | -0.61 (-0.69--0.52) |
| Acute myeloid leukemia | Latin America and Caribbean | 4762.21 (4488-5129.78) | 1.55 (1.47-1.65) | 10228.44 (9437.63-11103) | 1.68 (1.54-1.82) | 0.33 (0.26-0.4) |
| Acute myeloid leukemia | Low SDI | 2987.66 (1371.96-5392.04) | 0.83 (0.43-1.29) | 5971.68 (3645.62-7889.13) | 0.81 (0.51-1.08) | -0.11 (-0.17--0.05) |
| Acute myeloid leukemia | Low-middle SDI | 9555.88 (6251.34-15762.24) | 1.09 (0.74-1.65) | 18801.4 (14556.23-25892.55) | 1.18 (0.93-1.62) | 0.31 (0.27-0.35) |
| Acute myeloid leukemia | Middle SDI | 19742.02 (13362.42-28731.4) | 1.42 (1.01-2) | 31807.18 (26106.94-39669.48) | 1.24 (1.02-1.55) | -0.55 (-0.62--0.48) |
| Acute myeloid leukemia | Nordic Region | 809 (766.82-844.68) | 2.32 (2.21-2.41) | 1182.23 (1069.69-1275.08) | 2.09 (1.92-2.24) | -0.3 (-0.4--0.19) |
| Acute myeloid leukemia | North Africa and Middle East | 5540.85 (3722.6-7974.47) | 2.4 (1.68-3.3) | 10653.46 (7890.91-14414.9) | 2.15 (1.6-2.94) | -0.21 (-0.29--0.13) |
| Acute myeloid leukemia | OECD Countries | 29943.38 (28583.15-30999.61) | 2.33 (2.22-2.42) | 57428.72 (52199.1-60425.34) | 2.36 (2.18-2.47) | 0.16 (0.06-0.26) |
| Acute myeloid leukemia | Organization of Islamic Cooperation | 11600.25 (7548.26-17151.27) | 1.57 (1.06-2.18) | 22230.04 (16909.57-29412.63) | 1.52 (1.15-2.04) | -0.04 (-0.08-0) |
| Acute myeloid leukemia | Sahel Region | 684.03 (328.14-1364.24) | 0.66 (0.35-1.07) | 1513.07 (869.54-2146.8) | 0.64 (0.38-0.9) | 0 (-0.04-0.03) |
| Acute myeloid leukemia | South Asia | 7145.95 (4461.07-11772.2) | 0.87 (0.55-1.32) | 14565.83 (11115.8-20178.91) | 0.92 (0.69-1.28) | 0.11 (0.04-0.19) |
| Acute myeloid leukemia | Southeast Asia, East Asia, and Oceania | 22394.92 (12655.56-33443.9) | 1.58 (0.92-2.27) | 30585.42 (22167.33-38900.96) | 1.22 (0.89-1.55) | -1.07 (-1.2--0.93) |
| Acute myeloid leukemia | Sub-Saharan Africa | 2169.98 (1092.8-3513.24) | 0.63 (0.34-0.9) | 4292.87 (2535.14-5699.94) | 0.6 (0.37-0.79) | -0.14 (-0.18--0.11) |
| Acute myeloid leukemia | WHO region | 74467.14 (58307.6-94270.93) | 1.69 (1.35-2.08) | 129084.82 (112531.95-148282.74) | 1.56 (1.36-1.79) | -0.22 (-0.3--0.14) |
| Acute myeloid leukemia | World Bank Income Levels | 74832.78 (58657.84-94619.85) | 1.69 (1.36-2.08) | 130044.21 (113498.65-149228.5) | 1.57 (1.37-1.8) | -0.22 (-0.29--0.14) |
| Acute myeloid leukemia | World Bank Regions | 74788.53 (58629.07-94569.93) | 1.69 (1.36-2.08) | 129978.94 (113452.92-149154.21) | 1.57 (1.37-1.8) | -0.21 (-0.29--0.14) |
| Chronic lymphoid leukemia | American Samoa | 0 (0-0) | 0 (0-0) | 0 (0-0) | 0 (0-0) | 0.18 (-0.62-1) |
| Chronic lymphoid leukemia | Antigua and Barbuda | 0.3 (0.3-0.3) | 0.5 (0.4-0.6) | 0.4 (0.4-0.4) | 0.4 (0.4-0.5) | -0.2 (-0.97-0.58) |
| Chronic lymphoid leukemia | Arab Republic of Egypt | 132.4 (47.1-192) | 0.6 (0.2-1) | 574.6 (142.1-954.9) | 1.2 (0.3-1.8) | 0.92 (0.11-1.74) |
| Chronic lymphoid leukemia | Argentine Republic | 234.8 (200.1-273.2) | 0.8 (0.6-0.9) | 262 (220.9-306.3) | 0.4 (0.4-0.5) | -0.52 (-1.27-0.24) |
| Chronic lymphoid leukemia | Australia | 225.4 (203.5-245.8) | 1.2 (1.1-1.3) | 419.3 (345.8-488) | 0.8 (0.7-0.9) | -0.68 (-1.46-0.1) |
| Chronic lymphoid leukemia | Barbados | 1.5 (1.4-1.7) | 0.5 (0.4-0.5) | 3.1 (2.5-3.8) | 0.6 (0.5-0.7) | -0.03 (-0.83-0.77) |
| Chronic lymphoid leukemia | Belize | 0.4 (0.4-0.5) | 0.5 (0.4-0.5) | 1.6 (1.3-1.8) | 0.6 (0.5-0.6) | -0.03 (-0.62-0.57) |
| Chronic lymphoid leukemia | Bermuda | 0.4 (0.3-0.5) | 0.7 (0.6-0.8) | 0.6 (0.5-0.8) | 0.4 (0.4-0.5) | -0.87 (-1.63--0.11) |
| Chronic lymphoid leukemia | Bolivarian Republic of Venezuela | 36.4 (33.1-39.6) | 0.4 (0.4-0.4) | 103.3 (76.2-137) | 0.4 (0.3-0.5) | -0.22 (-0.94-0.51) |
| Chronic lymphoid leukemia | Bosnia and Herzegovina | 27.4 (18.5-43.7) | 0.7 (0.5-1.1) | 61.8 (38.4-91.7) | 1 (0.6-1.4) | -0.03 (-0.73-0.67) |
| Chronic lymphoid leukemia | Brunei Darussalam | 0.3 (0.2-0.5) | 0.4 (0.2-0.6) | 0.9 (0.4-1.4) | 0.3 (0.2-0.5) | -0.39 (-1.21-0.45) |
| Chronic lymphoid leukemia | Burkina Faso | 9.5 (5-13.1) | 0.3 (0.1-0.4) | 28.6 (9.5-40.9) | 0.4 (0.1-0.5) | 0.26 (-0.12-0.65) |
| Chronic lymphoid leukemia | Canada | 577.3 (529.2-613.2) | 1.8 (1.6-1.9) | 795.6 (683.8-894.4) | 1 (0.8-1.1) | -0.6 (-1.44-0.24) |
| Chronic lymphoid leukemia | Central African Republic | 6.4 (3.1-8.9) | 0.7 (0.3-1) | 13 (4.8-18.6) | 0.7 (0.3-1.1) | -0.06 (-0.65-0.54) |
| Chronic lymphoid leukemia | Commonwealth of Dominica | 0.4 (0.2-0.6) | 0.7 (0.4-1) | 0.6 (0.4-0.9) | 0.8 (0.5-1.1) | 0.07 (-0.69-0.83) |
| Chronic lymphoid leukemia | Commonwealth of the Bahamas | 1.1 (1-1.3) | 0.8 (0.7-0.9) | 2.7 (2.2-3.4) | 0.7 (0.6-0.9) | -0.14 (-0.76-0.5) |
| Chronic lymphoid leukemia | Cook Islands | 0 (0-0) | 0 (0-0.1) | 0 (0-0) | 0 (0-0) | -0.42 (-0.88-0.03) |
| Chronic lymphoid leukemia | Czech Republic | 153.3 (127.6-180.6) | 1.1 (0.9-1.3) | 286.4 (228.7-341.5) | 1.2 (1-1.5) | -0.7 (-1.46-0.08) |
| Chronic lymphoid leukemia | Democratic People's Republic of Korea | 53.7 (26-80.3) | 0.3 (0.2-0.5) | 108.8 (58.3-170.8) | 0.3 (0.2-0.5) | -0.24 (-1.03-0.56) |
| Chronic lymphoid leukemia | Democratic Republic of Sao Tome and Principe | 0.1 (0.1-0.2) | 0.2 (0.1-0.3) | 0.2 (0.1-0.3) | 0.2 (0.1-0.3) | -0.1 (-0.38-0.18) |
| Chronic lymphoid leukemia | Democratic Republic of the Congo | 69.9 (32.1-103.5) | 0.5 (0.3-0.9) | 195.9 (76.7-319.6) | 0.6 (0.3-1.1) | 0.02 (-0.51-0.54) |
| Chronic lymphoid leukemia | Democratic Republic of Timor-Leste | 0.5 (0.2-0.8) | 0.2 (0.1-0.3) | 1.4 (0.6-2.5) | 0.2 (0.1-0.3) | -0.3 (-1.08-0.49) |
| Chronic lymphoid leukemia | Democratic Socialist Republic of Sri Lanka | 4.1 (1.2-11.2) | 0 (0-0.1) | 18.8 (9.8-33.3) | 0.1 (0-0.1) | -0.77 (-1.55-0.01) |
| Chronic lymphoid leukemia | Dominican Republic | 10.7 (5.6-15.9) | 0.3 (0.2-0.5) | 35.8 (21.3-55.9) | 0.4 (0.2-0.6) | -0.06 (-0.7-0.57) |
| Chronic lymphoid leukemia | Eastern Republic of Uruguay | 60.3 (52.6-67.8) | 1.5 (1.3-1.7) | 90.1 (78.2-102.1) | 1.4 (1.2-1.6) | -0.36 (-1.13-0.42) |
| Chronic lymphoid leukemia | Federal Democratic Republic of Ethiopia | 524.9 (319-766.4) | 3.1 (2-4.5) | 882.4 (473-1475.2) | 2.3 (1.3-3.8) | -1.11 (-2.01--0.19) |
| Chronic lymphoid leukemia | Federal Democratic Republic of Nepal | 4.1 (1.6-8.2) | 0.1 (0-0.1) | 12.4 (5.5-23.5) | 0.1 (0-0.1) | -0.23 (-0.85-0.4) |
| Chronic lymphoid leukemia | Federal Republic of Germany | 1896.4 (1618.5-2157.4) | 1.4 (1.2-1.6) | 2447.5 (2036.1-2874.1) | 1 (0.9-1.2) | -0.55 (-1.36-0.27) |
| Chronic lymphoid leukemia | Federal Republic of Nigeria | 85.9 (47-117.9) | 0.2 (0.1-0.3) | 195.1 (94.1-264) | 0.3 (0.1-0.4) | -0.02 (-0.38-0.34) |
| Chronic lymphoid leukemia | Federal Republic of Somalia | 14.7 (8.3-22.3) | 0.8 (0.5-1.1) | 44.9 (22.6-69.9) | 0.9 (0.5-1.3) | 0.19 (-0.39-0.77) |
| Chronic lymphoid leukemia | Federated States of Micronesia | 0 (0-0.1) | 0.1 (0-0.2) | 0 (0-0) | 0 (0-0) | -0.28 (-1.15-0.59) |
| Chronic lymphoid leukemia | Federative Republic of Brazil | 369.7 (341.8-397.9) | 0.5 (0.4-0.5) | 1196 (1067-1295.9) | 0.5 (0.4-0.5) | -0.3 (-1.02-0.43) |
| Chronic lymphoid leukemia | French Republic | 1531.4 (1375.4-1671.8) | 1.7 (1.6-1.9) | 2032.5 (1632.7-2404.4) | 1.1 (0.9-1.3) | -0.61 (-1.42-0.2) |
| Chronic lymphoid leukemia | Gabonese Republic | 3.2 (1.6-4.3) | 0.6 (0.3-0.9) | 6.5 (3.1-9.9) | 0.7 (0.4-1.1) | -0.02 (-0.6-0.57) |
| Chronic lymphoid leukemia | Georgia | 37.2 (27.2-47.4) | 0.6 (0.4-0.8) | 35.7 (27.9-44.6) | 0.6 (0.5-0.8) | -0.01 (-0.71-0.7) |
| Chronic lymphoid leukemia | Grand Duchy of Luxembourg | 10.7 (9.8-11.5) | 2 (1.8-2.1) | 12.9 (11-14.5) | 1.1 (0.9-1.2) | -0.95 (-1.9-0) |
| Chronic lymphoid leukemia | Greenland | 0.1 (0-0.1) | 0.3 (0.1-0.4) | 0.2 (0-0.3) | 0.3 (0.1-0.4) | -0.64 (-1.29-0.03) |
| Chronic lymphoid leukemia | Grenada | 0.7 (0.6-0.9) | 1 (0.8-1.1) | 1.1 (1-1.3) | 1.1 (1-1.3) | -0.03 (-0.68-0.62) |
| Chronic lymphoid leukemia | Guam | 0.1 (0-0.2) | 0.1 (0-0.2) | 0 (0-0) | 0 (0-0) | -0.36 (-1.11-0.38) |
| Chronic lymphoid leukemia | Hashemite Kingdom of Jordan | 3.8 (1.9-7.7) | 0.4 (0.2-0.7) | 32.2 (14.2-47.4) | 0.6 (0.3-0.8) | -0.79 (-1.83-0.26) |
| Chronic lymphoid leukemia | Hellenic Republic | 206.9 (188.9-222.2) | 1.4 (1.3-1.5) | 320.3 (278-355.1) | 1 (0.9-1.1) | -0.69 (-1.52-0.14) |
| Chronic lymphoid leukemia | Hungary | 269.5 (248.1-290.2) | 1.8 (1.7-1.9) | 260.8 (222.9-299.1) | 1.3 (1.1-1.4) | -0.82 (-1.6--0.03) |
| Chronic lymphoid leukemia | Independent State of Papua New Guinea | 0.7 (0-2) | 0 (0-0.1) | 0.2 (0.1-0.4) | 0 (0-0) | -0.21 (-0.95-0.53) |
| Chronic lymphoid leukemia | Independent State of Samoa | 0.1 (0-0.2) | 0.1 (0-0.3) | 0 (0-0.1) | 0 (0-0) | -0.24 (-1.16-0.68) |
| Chronic lymphoid leukemia | Ireland | 75 (68.9-80.5) | 1.8 (1.7-2) | 71.5 (61.6-81.5) | 0.8 (0.7-1) | -1.01 (-1.81--0.19) |
| Chronic lymphoid leukemia | Islamic Republic of Afghanistan | 65.9 (11.4-115.2) | 1.2 (0.2-2) | 93.7 (19.5-173.9) | 1.2 (0.3-2.1) | -0.2 (-1.14-0.75) |
| Chronic lymphoid leukemia | Islamic Republic of Iran | 221 (86.4-308.6) | 1 (0.4-1.4) | 607.2 (226-785.1) | 0.9 (0.3-1.1) | -0.61 (-1.39-0.17) |
| Chronic lymphoid leukemia | Islamic Republic of Mauritania | 2.6 (1.5-3.6) | 0.3 (0.2-0.4) | 7.3 (2.9-11.1) | 0.4 (0.2-0.6) | 0.12 (-0.24-0.47) |
| Chronic lymphoid leukemia | Islamic Republic of Pakistan | 36.9 (13.5-66.4) | 0.1 (0-0.1) | 68.9 (35-125.6) | 0.1 (0-0.1) | -0.15 (-0.86-0.56) |
| Chronic lymphoid leukemia | Jamaica | 3.7 (3-4.5) | 0.2 (0.2-0.2) | 9.1 (6.8-11.9) | 0.3 (0.2-0.4) | 0.12 (-0.59-0.83) |
| Chronic lymphoid leukemia | Japan | 169.9 (157.1-178.3) | 0.1 (0.1-0.1) | 368.7 (304.4-406.7) | 0.1 (0.1-0.1) | -0.53 (-1.28-0.23) |
| Chronic lymphoid leukemia | Kingdom of Bahrain | 0.1 (0-0.1) | 0.1 (0-0.1) | 0.2 (0.1-0.4) | 0.1 (0-0.1) | -1.23 (-2.22--0.23) |
| Chronic lymphoid leukemia | Kingdom of Belgium | 223.3 (193.9-254.4) | 1.4 (1.2-1.6) | 245.1 (196.5-291.7) | 0.8 (0.7-1) | -0.78 (-1.6-0.05) |
| Chronic lymphoid leukemia | Kingdom of Bhutan | 0.1 (0-0.2) | 0 (0-0.1) | 0.3 (0.1-0.7) | 0.1 (0-0.1) | -0.34 (-0.99-0.32) |
| Chronic lymphoid leukemia | Kingdom of Cambodia | 11.5 (5.6-19.7) | 0.3 (0.1-0.5) | 29.1 (14.5-49.9) | 0.3 (0.1-0.5) | -0.48 (-1.35-0.4) |
| Chronic lymphoid leukemia | Kingdom of Denmark | 162.7 (145.7-181.2) | 1.9 (1.7-2.1) | 185 (152-213) | 1.4 (1.1-1.6) | -0.74 (-1.6-0.13) |
| Chronic lymphoid leukemia | Kingdom of Eswatini | 2.5 (1.6-3.3) | 1.1 (0.7-1.4) | 5.9 (3.2-9) | 1.3 (0.8-1.9) | 0.34 (-0.51-1.21) |
| Chronic lymphoid leukemia | Kingdom of Lesotho | 6.5 (3.6-9.2) | 0.9 (0.5-1.2) | 14.5 (7.1-21.2) | 1.6 (0.8-2.2) | 0.94 (0.14-1.74) |
| Chronic lymphoid leukemia | Kingdom of Morocco | 26.5 (4.6-45.1) | 0.2 (0-0.4) | 61.5 (14.1-99.2) | 0.2 (0-0.3) | -0.08 (-0.46-0.29) |
| Chronic lymphoid leukemia | Kingdom of Norway | 73 (66.4-77.9) | 1 (0.9-1) | 102.5 (87.2-115.7) | 0.9 (0.7-1) | -0.33 (-1.13-0.48) |
| Chronic lymphoid leukemia | Kingdom of Saudi Arabia | 20.8 (10.7-50.9) | 0.4 (0.2-1) | 99.7 (58.7-204.7) | 0.7 (0.4-1.3) | 0.26 (-0.34-0.87) |
| Chronic lymphoid leukemia | Kingdom of Spain | 686 (611.6-745.4) | 1.2 (1.1-1.3) | 961.4 (780.2-1137.3) | 0.8 (0.6-0.9) | -0.8 (-1.53--0.07) |
| Chronic lymphoid leukemia | Kingdom of Sweden | 244.5 (222.3-265.7) | 1.5 (1.3-1.6) | 240.4 (198.3-278.9) | 0.9 (0.7-1) | -0.55 (-1.35-0.26) |
| Chronic lymphoid leukemia | Kingdom of Thailand | 119.4 (38.2-183.1) | 0.4 (0.1-0.6) | 231 (112.2-356.2) | 0.2 (0.1-0.3) | -0.59 (-1.45-0.27) |
| Chronic lymphoid leukemia | Kingdom of the Netherlands | 285 (255.9-309) | 1.4 (1.2-1.5) | 226.1 (183.8-265.7) | 0.6 (0.5-0.7) | -0.65 (-1.48-0.19) |
| Chronic lymphoid leukemia | Kingdom of Tonga | 0 (0-0.1) | 0.1 (0-0.2) | 0 (0-0) | 0 (0-0) | -0.13 (-0.88-0.62) |
| Chronic lymphoid leukemia | Kyrgyz Republic | 10.8 (9.3-12.4) | 0.3 (0.3-0.4) | 10.2 (8-12.9) | 0.2 (0.2-0.3) | -0.67 (-1.25--0.1) |
| Chronic lymphoid leukemia | Lao People's Democratic Republic | 4.8 (2.5-9.1) | 0.3 (0.1-0.5) | 8.4 (4.4-15) | 0.2 (0.1-0.4) | -0.77 (-1.63-0.1) |
| Chronic lymphoid leukemia | Lebanese Republic | 19.3 (10.4-35.2) | 1 (0.6-1.8) | 66 (36.1-93.5) | 1 (0.6-1.4) | -0.36 (-1.17-0.46) |
| Chronic lymphoid leukemia | Malaysia | 16.2 (9.5-25.3) | 0.2 (0.1-0.3) | 49.6 (32.8-91) | 0.2 (0.1-0.4) | -0.31 (-1.14-0.53) |
| Chronic lymphoid leukemia | Mongolia | 1.9 (0.7-3.2) | 0.2 (0.1-0.3) | 3.2 (1.7-5.3) | 0.1 (0.1-0.2) | -0.43 (-1.11-0.26) |
| Chronic lymphoid leukemia | Montenegro | 5.4 (3.3-7.9) | 0.9 (0.6-1.3) | 11.3 (6.8-18.4) | 1.2 (0.7-1.9) | -0.12 (-0.87-0.64) |
| Chronic lymphoid leukemia | New Zealand | 51.9 (46.2-57.6) | 1.3 (1.2-1.5) | 91.6 (77.7-104.8) | 1 (0.8-1.1) | -0.58 (-1.44-0.28) |
| Chronic lymphoid leukemia | North Macedonia | 22.7 (15.1-32.8) | 1.3 (0.9-1.9) | 44.4 (27.3-63.7) | 1.5 (1-2.1) | -0.23 (-1.25-0.79) |
| Chronic lymphoid leukemia | Northern Mariana Islands | 0 (0-0) | 0 (0-0) | 0 (0-0) | 0 (0-0) | -0.52 (-1.28-0.24) |
| Chronic lymphoid leukemia | Palestine | 9 (5.6-15.3) | 1.2 (0.7-2) | 20.3 (13-32.1) | 1 (0.6-1.6) | -0.8 (-1.71-0.11) |
| Chronic lymphoid leukemia | People's Democratic Republic of Algeria | 12.5 (4.4-18.3) | 0.2 (0.1-0.2) | 41.1 (14.6-66.7) | 0.2 (0.1-0.2) | -0.28 (-0.92-0.36) |
| Chronic lymphoid leukemia | People's Republic of Bangladesh | 31.1 (11.6-56.1) | 0.1 (0-0.1) | 82.1 (28.5-155.1) | 0.1 (0-0.1) | -0.59 (-1.24-0.06) |
| Chronic lymphoid leukemia | People's Republic of China | 4854.1 (2824.2-6572.6) | 0.5 (0.3-0.7) | 8636.1 (5526.7-12383.9) | 0.4 (0.3-0.6) | -1.08 (-1.87--0.29) |
| Chronic lymphoid leukemia | Plurinational State of Bolivia | 11.2 (6.7-18.9) | 0.4 (0.2-0.6) | 32.9 (19.2-50.3) | 0.4 (0.2-0.6) | -0.42 (-1.3-0.48) |
| Chronic lymphoid leukemia | Portuguese Republic | 168.4 (152.9-184.2) | 1.3 (1.1-1.4) | 257.9 (210-298.3) | 0.8 (0.7-1) | -0.79 (-1.59-0.01) |
| Chronic lymphoid leukemia | Principality of Andorra | 1 (0.6-1.6) | 1.9 (1.1-3.1) | 1.9 (1.1-3.2) | 1.1 (0.7-2) | -0.9 (-1.78--0.01) |
| Chronic lymphoid leukemia | Principality of Monaco | 1.7 (1-2.6) | 2.1 (1.3-3.2) | 2.3 (1.2-3.4) | 2 (1.1-2.9) | 0.1 (-0.95-1.17) |
| Chronic lymphoid leukemia | Puerto Rico | 38.3 (34.1-42.8) | 1.1 (1-1.2) | 45.7 (35.9-55.2) | 0.5 (0.4-0.7) | -0.7 (-1.46-0.06) |
| Chronic lymphoid leukemia | Republic of Albania | 11.9 (6.3-20.9) | 0.6 (0.3-1.1) | 25.1 (12.4-54.4) | 0.6 (0.3-1.2) | -0.39 (-1.07-0.3) |
| Chronic lymphoid leukemia | Republic of Angola | 18.5 (7.9-27) | 0.6 (0.3-0.9) | 63.8 (24.1-103.1) | 0.7 (0.3-1.1) | -0.09 (-0.64-0.47) |
| Chronic lymphoid leukemia | Republic of Armenia | 25.4 (22.7-27.6) | 0.9 (0.8-1) | 24.2 (20.9-28.7) | 0.6 (0.5-0.7) | -0.62 (-1.3-0.06) |
| Chronic lymphoid leukemia | Republic of Austria | 201.4 (183.1-218) | 1.6 (1.5-1.7) | 245.8 (201.9-282.9) | 1.1 (0.9-1.3) | -0.49 (-1.3-0.34) |
| Chronic lymphoid leukemia | Republic of Azerbaijan | 15.3 (8-26.6) | 0.3 (0.2-0.5) | 24.9 (10.5-52.9) | 0.2 (0.1-0.5) | -0.64 (-1.38-0.1) |
| Chronic lymphoid leukemia | Republic of Belarus | 167.2 (136.2-202.7) | 1.3 (1.1-1.6) | 218.6 (170.7-276.5) | 1.3 (1-1.7) | -0.69 (-1.4-0.03) |
| Chronic lymphoid leukemia | Republic of Benin | 4.4 (2.6-5.8) | 0.2 (0.1-0.3) | 15.1 (5.3-21.4) | 0.3 (0.1-0.5) | 0.2 (-0.18-0.58) |
| Chronic lymphoid leukemia | Republic of Botswana | 4.4 (2.4-6.2) | 1 (0.5-1.4) | 11.7 (5.7-17.4) | 1 (0.5-1.4) | -0.07 (-0.81-0.67) |
| Chronic lymphoid leukemia | Republic of Bulgaria | 57.1 (45-71.9) | 0.5 (0.4-0.6) | 95.2 (66.8-131.7) | 0.6 (0.4-0.9) | 0.05 (-0.67-0.78) |
| Chronic lymphoid leukemia | Republic of Burundi | 15.1 (8.5-22) | 0.7 (0.4-1) | 31.4 (14.1-48) | 0.8 (0.4-1.1) | -0.13 (-0.68-0.42) |
| Chronic lymphoid leukemia | Republic of Cabo Verde | 1.2 (0.8-1.9) | 0.5 (0.3-0.8) | 3.6 (2.1-5.3) | 0.9 (0.5-1.3) | 0.14 (-0.46-0.75) |
| Chronic lymphoid leukemia | Republic of Cameroon | 10.9 (6.3-15.3) | 0.3 (0.2-0.4) | 41.3 (14.6-67.1) | 0.4 (0.2-0.6) | 0.23 (-0.18-0.64) |
| Chronic lymphoid leukemia | Republic of Chad | 5.4 (3-7.7) | 0.2 (0.1-0.3) | 16.9 (5.7-25.7) | 0.4 (0.1-0.5) | 0.39 (0.02-0.77) |
| Chronic lymphoid leukemia | Republic of Chile | 48.4 (42.4-55.3) | 0.5 (0.5-0.6) | 94.2 (79-110.5) | 0.4 (0.3-0.4) | -0.41 (-1.09-0.27) |
| Chronic lymphoid leukemia | Republic of Colombia | 80.7 (71.2-90.5) | 0.5 (0.4-0.6) | 178.4 (143-217.8) | 0.3 (0.3-0.4) | -0.44 (-1.19-0.31) |
| Chronic lymphoid leukemia | Republic of Costa Rica | 10.5 (8.8-12.1) | 0.6 (0.5-0.7) | 33.4 (28-39.2) | 0.6 (0.5-0.7) | -0.11 (-0.88-0.68) |
| Chronic lymphoid leukemia | République de Côte d’Ivoire | 6.3 (3.2-8.4) | 0.2 (0.1-0.3) | 20.3 (9-30.5) | 0.2 (0.1-0.3) | -0.02 (-0.32-0.28) |
| Chronic lymphoid leukemia | Republic of Croatia | 75.4 (65.8-87) | 1.3 (1.2-1.5) | 144 (120.3-171.3) | 1.4 (1.2-1.7) | -0.54 (-1.3-0.23) |
| Chronic lymphoid leukemia | Republic of Cuba | 87.7 (78.3-95.7) | 0.9 (0.8-1) | 121.5 (102.9-139.9) | 0.6 (0.5-0.7) | -0.44 (-1.14-0.26) |
| Chronic lymphoid leukemia | Republic of Cyprus | 14.7 (9.4-25.6) | 2.5 (1.6-4.3) | 30.6 (19.1-41.1) | 1.6 (1-2.1) | -0.67 (-1.65-0.33) |
| Chronic lymphoid leukemia | Republic of Djibouti | 0.7 (0.3-1.1) | 0.7 (0.4-1) | 4.8 (2-7.7) | 0.9 (0.4-1.4) | 0.21 (-0.35-0.77) |
| Chronic lymphoid leukemia | Republic of Ecuador | 14.7 (12.3-17.3) | 0.3 (0.2-0.3) | 46.7 (35.8-60) | 0.3 (0.2-0.4) | 0.33 (-0.52-1.18) |
| Chronic lymphoid leukemia | Republic of El Salvador | 7.9 (4.9-11) | 0.3 (0.2-0.4) | 21.2 (12.6-29.9) | 0.3 (0.2-0.5) | -0.02 (-0.83-0.79) |
| Chronic lymphoid leukemia | Republic of Equatorial Guinea | 1 (0.5-1.4) | 0.6 (0.3-0.9) | 2.9 (1.2-4.9) | 0.7 (0.3-1.1) | -0.19 (-0.72-0.35) |
| Chronic lymphoid leukemia | Republic of Estonia | 54.6 (48.2-61.9) | 2.7 (2.4-3) | 48.5 (38.7-58.3) | 1.6 (1.3-2) | -1 (-1.8--0.2) |
| Chronic lymphoid leukemia | Republic of Fiji | 0.1 (0-0.2) | 0 (0-0) | 0.1 (0-0.2) | 0 (0-0) | -0.32 (-1.45-0.82) |
| Chronic lymphoid leukemia | Republic of Finland | 94.6 (84.3-105) | 1.3 (1.2-1.4) | 118.5 (95.9-137.8) | 0.8 (0.6-0.9) | -0.61 (-1.32-0.11) |
| Chronic lymphoid leukemia | Republic of Ghana | 25.5 (10.6-35.4) | 0.5 (0.2-0.7) | 49.4 (32.5-75.6) | 0.3 (0.2-0.5) | -0.75 (-1.24--0.27) |
| Chronic lymphoid leukemia | Republic of Guatemala | 5.4 (4.8-6.1) | 0.2 (0.2-0.2) | 18.8 (16.1-22) | 0.2 (0.2-0.2) | -0.01 (-0.78-0.77) |
| Chronic lymphoid leukemia | Republic of Guinea | 2.6 (1.5-3.7) | 0.1 (0-0.1) | 5.1 (2.4-7.4) | 0.1 (0-0.1) | -0.02 (-0.21-0.16) |
| Chronic lymphoid leukemia | Republic of Guinea-Bissau | 1 (0.5-1.5) | 0.3 (0.2-0.4) | 2.6 (1-3.9) | 0.5 (0.2-0.7) | 0.24 (-0.2-0.68) |
| Chronic lymphoid leukemia | Republic of Guyana | 2 (1.7-2.3) | 0.6 (0.5-0.7) | 3 (2.3-3.9) | 0.5 (0.4-0.6) | 0.13 (-0.52-0.78) |
| Chronic lymphoid leukemia | Republic of Haiti | 17.3 (9.7-30.9) | 0.6 (0.4-1.1) | 34.8 (20.2-58) | 0.6 (0.3-1) | -0.34 (-1.08-0.41) |
| Chronic lymphoid leukemia | Republic of Honduras | 6.4 (3.6-9.1) | 0.3 (0.2-0.5) | 27.4 (16.2-44) | 0.5 (0.3-0.8) | -0.13 (-0.92-0.67) |
| Chronic lymphoid leukemia | Republic of Iceland | 3 (2.7-3.5) | 1 (0.9-1.1) | 4.5 (3.7-5.3) | 0.7 (0.6-0.8) | -0.35 (-1.12-0.43) |
| Chronic lymphoid leukemia | Republic of India | 311.5 (112.6-433.7) | 0.1 (0-0.1) | 977 (471.8-1352.4) | 0.1 (0-0.1) | -0.31 (-0.92-0.29) |
| Chronic lymphoid leukemia | Republic of Indonesia | 161.4 (87.9-229.4) | 0.2 (0.1-0.3) | 458.1 (282-787.6) | 0.2 (0.1-0.4) | -0.16 (-0.99-0.69) |
| Chronic lymphoid leukemia | Republic of Iraq | 46.7 (12.4-81.1) | 0.6 (0.2-1.1) | 124.1 (40-199.9) | 0.6 (0.2-0.9) | -0.36 (-1.22-0.5) |
| Chronic lymphoid leukemia | Republic of Italy | 1406.9 (1292.4-1491.3) | 1.5 (1.4-1.6) | 1903.3 (1602.9-2130.5) | 1 (0.9-1.1) | -0.84 (-1.62--0.04) |
| Chronic lymphoid leukemia | Republic of Kazakhstan | 42.8 (25.9-66.8) | 0.3 (0.2-0.5) | 47.9 (28.3-75) | 0.3 (0.2-0.4) | -0.54 (-1.21-0.12) |
| Chronic lymphoid leukemia | Republic of Kenya | 27.4 (17.7-44.2) | 0.4 (0.3-0.6) | 100.9 (68.5-143.1) | 0.5 (0.4-0.8) | 0.29 (-0.18-0.76) |
| Chronic lymphoid leukemia | Republic of Kiribati | 0 (0-0) | 0 (0-0.1) | 0 (0-0) | 0 (0-0) | -0.04 (-0.76-0.68) |
| Chronic lymphoid leukemia | Republic of Korea | 36.9 (24.7-68.4) | 0.1 (0.1-0.3) | 72.1 (43.9-119.9) | 0.1 (0-0.1) | -1 (-1.72--0.27) |
| Chronic lymphoid leukemia | Republic of Latvia | 69.2 (56.2-81.8) | 1.9 (1.6-2.3) | 74.1 (61.3-91.1) | 1.8 (1.4-2.2) | -0.81 (-1.58--0.03) |
| Chronic lymphoid leukemia | Republic of Liberia | 2.4 (1.3-3.2) | 0.2 (0.1-0.3) | 6.4 (2.2-9.7) | 0.4 (0.1-0.6) | 0.23 (-0.14-0.6) |
| Chronic lymphoid leukemia | Republic of Lithuania | 104.3 (88.8-118.2) | 2.3 (2-2.6) | 91.1 (74.4-108.6) | 1.5 (1.2-1.7) | -0.54 (-1.31-0.25) |
| Chronic lymphoid leukemia | Republic of Madagascar | 25.4 (13.5-33.9) | 0.6 (0.3-0.8) | 57.4 (27.1-82) | 0.6 (0.3-0.9) | 0.01 (-0.48-0.5) |
| Chronic lymphoid leukemia | Republic of Malawi | 6.9 (3.6-9.2) | 0.2 (0.1-0.3) | 15.7 (8.3-21.9) | 0.2 (0.1-0.3) | -0.09 (-0.38-0.2) |
| Chronic lymphoid leukemia | Republic of Maldives | 0.1 (0.1-0.3) | 0.2 (0.1-0.3) | 0.3 (0.1-0.6) | 0.1 (0-0.2) | -1.16 (-1.89--0.42) |
| Chronic lymphoid leukemia | Republic of Mali | 7.4 (3.8-10.2) | 0.2 (0.1-0.3) | 17.1 (8-23.9) | 0.2 (0.1-0.3) | -0.11 (-0.42-0.2) |
| Chronic lymphoid leukemia | Republic of Malta | 3.5 (3-4) | 0.9 (0.7-1) | 5.1 (4.1-6.3) | 0.5 (0.4-0.6) | -0.66 (-1.46-0.15) |
| Chronic lymphoid leukemia | Republic of Mauritius | 8.4 (7.6-9.4) | 1.2 (1.1-1.4) | 1.7 (1.5-2) | 0.1 (0.1-0.1) | -0.51 (-1.24-0.22) |
| Chronic lymphoid leukemia | Republic of Moldova | 21.1 (17.6-24) | 0.5 (0.4-0.6) | 21.9 (18.9-25.1) | 0.4 (0.3-0.4) | -0.74 (-1.42--0.06) |
| Chronic lymphoid leukemia | Republic of Mozambique | 49.4 (28.4-66.7) | 1 (0.6-1.4) | 109.1 (66.9-155.1) | 1.2 (0.8-1.7) | 0.14 (-0.52-0.8) |
| Chronic lymphoid leukemia | Republic of Namibia | 3.6 (2.4-4.8) | 0.7 (0.5-0.9) | 8 (5.3-11.4) | 0.7 (0.5-1) | -0.03 (-0.65-0.6) |
| Chronic lymphoid leukemia | Republic of Nauru | 0 (0-0) | 0.1 (0-0.3) | 0 (0-0) | 0 (0-0) | -0.22 (-1.15-0.73) |
| Chronic lymphoid leukemia | Republic of Nicaragua | 5 (3.3-7.1) | 0.4 (0.2-0.5) | 17.1 (10.8-23.2) | 0.4 (0.2-0.5) | -0.21 (-0.94-0.53) |
| Chronic lymphoid leukemia | Republic of Niue | 0 (0-0) | 0.1 (0-0.1) | 0 (0-0) | 0 (0-0) | -0.06 (-0.86-0.74) |
| Chronic lymphoid leukemia | Republic of Palau | 0 (0-0) | 0.3 (0.1-0.4) | 0 (0-0.1) | 0.2 (0.1-0.4) | -0.16 (-0.66-0.36) |
| Chronic lymphoid leukemia | Republic of Panama | 4.3 (3.9-4.7) | 0.3 (0.3-0.3) | 14 (10.9-17.1) | 0.3 (0.2-0.4) | 0.06 (-0.7-0.83) |
| Chronic lymphoid leukemia | Republic of Paraguay | 12.1 (8.1-16.8) | 0.6 (0.4-0.8) | 44.5 (29-64.9) | 0.8 (0.5-1.2) | 0.24 (-0.47-0.96) |
| Chronic lymphoid leukemia | Republic of Peru | 24.9 (16.1-38) | 0.2 (0.1-0.3) | 81.6 (42.6-117) | 0.2 (0.1-0.4) | -0.18 (-0.97-0.62) |
| Chronic lymphoid leukemia | Republic of Poland | 621.2 (542-684.6) | 1.4 (1.3-1.6) | 1119.3 (1003.7-1224.4) | 1.5 (1.3-1.6) | -0.79 (-1.55--0.02) |
| Chronic lymphoid leukemia | Republic of Rwanda | 20.9 (13.1-28.2) | 0.9 (0.6-1.1) | 47.3 (23-70.5) | 0.9 (0.5-1.3) | -0.5 (-1.08-0.08) |
| Chronic lymphoid leukemia | Republic of San Marino | 0.6 (0.4-0.9) | 1.7 (1.1-2.5) | 0.8 (0.4-1.2) | 0.8 (0.4-1.3) | -0.78 (-1.6-0.04) |
| Chronic lymphoid leukemia | Republic of Senegal | 6.8 (3.9-9.1) | 0.2 (0.1-0.3) | 25.9 (9.7-37.6) | 0.4 (0.1-0.6) | 0.23 (-0.16-0.61) |
| Chronic lymphoid leukemia | Republic of Serbia | 96.9 (72-142.7) | 1 (0.8-1.5) | 190.3 (133.3-276.7) | 1.1 (0.8-1.6) | -0.51 (-1.26-0.25) |
| Chronic lymphoid leukemia | Republic of Seychelles | 0.5 (0.3-0.7) | 0.9 (0.6-1.3) | 0.8 (0.5-1.1) | 0.8 (0.5-1.1) | -0.43 (-1.15-0.29) |
| Chronic lymphoid leukemia | Republic of Sierra Leone | 4 (2.2-5.4) | 0.2 (0.1-0.3) | 11.1 (3.9-16) | 0.3 (0.1-0.5) | 0.26 (-0.11-0.64) |
| Chronic lymphoid leukemia | Republic of Singapore | 3.9 (3.6-4.3) | 0.2 (0.2-0.2) | 9.6 (8.3-10.7) | 0.1 (0.1-0.1) | -0.85 (-1.66--0.04) |
| Chronic lymphoid leukemia | Republic of Slovenia | 39.4 (34-45.4) | 1.6 (1.4-1.8) | 80 (64.2-97.1) | 1.6 (1.3-1.9) | -0.5 (-1.23-0.23) |
| Chronic lymphoid leukemia | Republic of South Africa | 183 (77.8-264.2) | 1 (0.4-1.4) | 473.7 (230.2-627.1) | 1.2 (0.6-1.5) | 0.09 (-0.68-0.86) |
| Chronic lymphoid leukemia | Republic of South Sudan | 15.6 (8.8-23.5) | 0.7 (0.4-1) | 29.1 (15.7-42.9) | 0.9 (0.5-1.3) | 0.3 (-0.25-0.86) |
| Chronic lymphoid leukemia | Republic of Sudan | 69.2 (11.6-127.8) | 0.9 (0.1-1.6) | 144 (27.6-259.6) | 0.9 (0.2-1.5) | -0.33 (-1.16-0.51) |
| Chronic lymphoid leukemia | Republic of Suriname | 0.9 (0.5-1.2) | 0.4 (0.2-0.5) | 2.3 (1.3-3.4) | 0.4 (0.2-0.6) | -0.16 (-0.77-0.45) |
| Chronic lymphoid leukemia | Republic of Tajikistan | 6.9 (3.1-11.7) | 0.2 (0.1-0.4) | 8.7 (4.3-16.1) | 0.1 (0.1-0.2) | -0.75 (-1.39--0.1) |
| Chronic lymphoid leukemia | Republic of the Congo | 6.4 (3.1-8.8) | 0.7 (0.4-0.9) | 16.8 (7.2-23.6) | 0.7 (0.3-1) | -0.14 (-0.73-0.45) |
| Chronic lymphoid leukemia | Republic of the Gambia | 0.4 (0.2-0.6) | 0.1 (0.1-0.2) | 1.5 (0.8-2.1) | 0.2 (0.1-0.2) | -0.02 (-0.24-0.2) |
| Chronic lymphoid leukemia | Republic of the Marshall Islands | 0 (0-0) | 0.1 (0-0.2) | 0 (0-0) | 0 (0-0) | -0.05 (-0.88-0.79) |
| Chronic lymphoid leukemia | Republic of the Niger | 4.7 (2.6-6.6) | 0.2 (0.1-0.3) | 22.2 (7.4-35.2) | 0.3 (0.1-0.5) | 0.12 (-0.25-0.5) |
| Chronic lymphoid leukemia | Republic of the Philippines | 69.7 (27.1-94.9) | 0.3 (0.1-0.3) | 153.8 (81.8-235.5) | 0.2 (0.1-0.3) | -0.35 (-1.19-0.49) |
| Chronic lymphoid leukemia | Republic of the Union of Myanmar | 54.6 (29.3-90.7) | 0.3 (0.1-0.4) | 76.6 (48.9-145.9) | 0.2 (0.1-0.3) | -0.95 (-1.84--0.07) |
| Chronic lymphoid leukemia | Republic of Trinidad and Tobago | 3.7 (3.3-4) | 0.5 (0.4-0.5) | 6.5 (5-8.3) | 0.3 (0.3-0.4) | -0.43 (-1.12-0.27) |
| Chronic lymphoid leukemia | Republic of Tunisia | 25.6 (5-43.7) | 0.6 (0.1-1.1) | 60 (12.6-102.7) | 0.5 (0.1-0.8) | -0.48 (-1.11-0.16) |
| Chronic lymphoid leukemia | Republic of Turkey | 402.7 (225.7-556.3) | 1.3 (0.8-1.8) | 850.2 (518.6-1230.2) | 1 (0.6-1.4) | -1.01 (-1.89--0.12) |
| Chronic lymphoid leukemia | Republic of Uganda | 24.8 (14.3-33.8) | 0.4 (0.3-0.6) | 59.4 (35-83.5) | 0.5 (0.3-0.7) | -0.04 (-0.5-0.43) |
| Chronic lymphoid leukemia | Republic of Uzbekistan | 33.4 (24.3-44.3) | 0.3 (0.2-0.4) | 51.9 (35-72.7) | 0.2 (0.1-0.3) | -0.68 (-1.33--0.03) |
| Chronic lymphoid leukemia | Republic of Vanuatu | 0 (0-0.1) | 0.1 (0-0.2) | 0 (0-0) | 0 (0-0) | -0.13 (-0.91-0.65) |
| Chronic lymphoid leukemia | Republic of Yemen | 40 (5.3-77.7) | 1 (0.1-1.9) | 116.1 (17.9-213.7) | 1 (0.2-1.8) | -0.28 (-1.07-0.51) |
| Chronic lymphoid leukemia | Republic of Zambia | 17.4 (11.2-22.5) | 0.7 (0.5-0.9) | 56.8 (22.3-90.6) | 0.9 (0.4-1.4) | -0.05 (-0.63-0.53) |
| Chronic lymphoid leukemia | Republic of Zimbabwe | 33.9 (19.6-47.6) | 1 (0.6-1.4) | 78.8 (44.8-108.4) | 1.3 (0.9-1.8) | 0.62 (-0.21-1.45) |
| Chronic lymphoid leukemia | Romania | 140.2 (125.2-153.3) | 0.5 (0.5-0.6) | 296.6 (255.4-338.7) | 0.7 (0.6-0.9) | -0.11 (-0.79-0.57) |
| Chronic lymphoid leukemia | Russian Federation | 1438 (1337.4-1571.7) | 0.8 (0.7-0.9) | 2155.4 (1979.1-2343.4) | 0.9 (0.8-1) | -0.44 (-1.09-0.21) |
| Chronic lymphoid leukemia | Saint Kitts and Nevis | 0.3 (0.2-0.3) | 0.7 (0.6-0.8) | 0.4 (0.3-0.5) | 0.6 (0.5-0.7) | -0.42 (-1.11-0.28) |
| Chronic lymphoid leukemia | Saint Lucia | 0.7 (0.6-0.8) | 0.9 (0.8-0.9) | 1.8 (1.5-2.2) | 0.8 (0.6-0.9) | -0.73 (-1.34--0.11) |
| Chronic lymphoid leukemia | Saint Vincent and the Grenadines | 0.8 (0.7-0.8) | 1.1 (1-1.2) | 1.1 (1-1.3) | 0.8 (0.7-0.9) | -0.29 (-0.95-0.37) |
| Chronic lymphoid leukemia | Slovak Republic | 79.5 (43.4-113.7) | 1.3 (0.7-1.9) | 132.1 (80.6-182) | 1.4 (0.8-1.9) | -0.52 (-1.32-0.29) |
| Chronic lymphoid leukemia | Socialist Republic of Viet Nam | 10.5 (3.6-15.7) | 0 (0-0) | 19.4 (10.2-29) | 0 (0-0) | -0.39 (-1.08-0.3) |
| Chronic lymphoid leukemia | Solomon Islands | 0.1 (0-0.2) | 0.1 (0-0.2) | 0 (0-0) | 0 (0-0) | -0.08 (-0.9-0.74) |
| Chronic lymphoid leukemia | State of Eritrea | 6.3 (3.8-9.1) | 0.7 (0.4-1) | 20.3 (10.7-29.6) | 0.9 (0.5-1.3) | 0.18 (-0.38-0.75) |
| Chronic lymphoid leukemia | State of Israel | 110.2 (97.1-125.2) | 2.4 (2.1-2.7) | 215.3 (177-247) | 1.6 (1.3-1.8) | -0.73 (-1.7-0.25) |
| Chronic lymphoid leukemia | State of Kuwait | 1.7 (1.4-2.1) | 0.4 (0.3-0.4) | 7.2 (5.6-9) | 0.3 (0.2-0.4) | -1.06 (-1.75--0.36) |
| Chronic lymphoid leukemia | State of Libya | 18.8 (3.8-31.7) | 1.1 (0.2-1.9) | 47.6 (11.9-76) | 1.1 (0.3-1.7) | 0.11 (-0.77-0.99) |
| Chronic lymphoid leukemia | State of Qatar | 1 (0.4-1.6) | 1.4 (0.6-2.2) | 5 (1.8-8.3) | 0.8 (0.4-1.3) | -1.42 (-2.21--0.63) |
| Chronic lymphoid leukemia | Sultanate of Oman | 4.1 (1.5-7.2) | 0.7 (0.2-1.1) | 9.3 (4.4-15.4) | 0.6 (0.3-0.9) | -0.44 (-1.2-0.33) |
| Chronic lymphoid leukemia | Swiss Confederation | 160.8 (136.1-182.9) | 1.4 (1.2-1.6) | 193.6 (153.3-234.5) | 0.9 (0.7-1) | -0.68 (-1.44-0.08) |
| Chronic lymphoid leukemia | Syrian Arab Republic | 65.6 (12-113) | 1.5 (0.3-2.5) | 130.6 (32.4-215.9) | 1.3 (0.3-2) | -0.8 (-1.65-0.06) |
| Chronic lymphoid leukemia | Taiwan (Province of China) | 18.1 (14.2-23.1) | 0.1 (0.1-0.2) | 70.2 (58.4-84) | 0.2 (0.1-0.2) | 0.19 (-0.59-0.99) |
| Chronic lymphoid leukemia | Togolese Republic | 2.5 (1.4-3.3) | 0.2 (0.1-0.3) | 12.6 (4.1-18.8) | 0.4 (0.1-0.6) | 0.28 (-0.09-0.66) |
| Chronic lymphoid leukemia | Tokelau | 0 (0-0) | 0.1 (0-0.2) | 0 (0-0) | 0 (0-0) | -0.17 (-0.97-0.64) |
| Chronic lymphoid leukemia | Turkmenistan | 4.5 (3-6.3) | 0.2 (0.1-0.3) | 7.2 (4.3-11.2) | 0.2 (0.1-0.3) | -0.41 (-1.04-0.21) |
| Chronic lymphoid leukemia | Tuvalu | 0 (0-0) | 0.1 (0-0.2) | 0 (0-0) | 0 (0-0) | -0.34 (-1.16-0.48) |
| Chronic lymphoid leukemia | Ukraine | 628.5 (480.8-774.9) | 0.9 (0.7-1.1) | 605.8 (442.9-805.1) | 0.8 (0.6-1) | -1.13 (-1.8--0.46) |
| Chronic lymphoid leukemia | Union of the Comoros | 1.2 (0.7-1.7) | 0.7 (0.4-1) | 3.8 (1.9-5.4) | 0.9 (0.5-1.2) | 0.02 (-0.53-0.57) |
| Chronic lymphoid leukemia | United Arab Emirates | 4.7 (2.7-7.8) | 1.4 (0.8-2.2) | 25.9 (14.7-41.8) | 1.2 (0.8-1.9) | -0.09 (-0.97-0.79) |
| Chronic lymphoid leukemia | United Kingdom of Great Britain and Northern Ireland | 1052.7 (974.9-1097.2) | 1.1 (1-1.1) | 1233.2 (1091-1314.5) | 0.8 (0.7-0.9) | -0.46 (-1.32-0.41) |
| Chronic lymphoid leukemia | United Mexican States | 134.9 (129.5-139.9) | 0.4 (0.3-0.4) | 384.8 (341.3-429.1) | 0.3 (0.3-0.4) | -0.21 (-1.03-0.62) |
| Chronic lymphoid leukemia | United Republic of Tanzania | 67.9 (37.2-94.4) | 0.7 (0.4-1) | 179.2 (84.4-266.8) | 0.8 (0.4-1.2) | -0.01 (-0.56-0.55) |
| Chronic lymphoid leukemia | United States of America | 5750.1 (5280.9-6004.1) | 1.7 (1.6-1.8) | 6456.4 (5588.5-6935.4) | 1 (0.9-1.1) | -0.7 (-1.61-0.21) |
| Chronic lymphoid leukemia | United States Virgin Islands | 1.2 (0.8-1.5) | 1.5 (1-1.9) | 1.7 (1.1-2.4) | 1 (0.6-1.3) | -0.54 (-1.26-0.18) |
| Chronic lymphoid leukemia | African Union | 1635.55 (895.39-2103.71) | 0.67 (0.37-0.87) | 3947.56 (1973.85-5235.48) | 0.72 (0.37-0.96) | 0.35 (0.3-0.4) |
| Chronic lymphoid leukemia | Association of Southeast Asian Nations | 452.28 (231.95-594.22) | 0.2 (0.1-0.27) | 1036.54 (650.25-1539.56) | 0.18 (0.11-0.26) | -0.58 (-0.7--0.46) |
| Chronic lymphoid leukemia | Central Europe, Eastern Europe, and Central Asia | 4287.01 (3941.57-4625.63) | 0.91 (0.84-0.98) | 6217.46 (5732.97-6664.04) | 0.94 (0.86-1) | -0.12 (-0.3-0.07) |
| Chronic lymphoid leukemia | Commonwealth | 2896.44 (2461.79-3174.45) | 0.44 (0.38-0.48) | 5196.08 (3943.73-5859.71) | 0.31 (0.25-0.35) | -1.19 (-1.32--1.06) |
| Chronic lymphoid leukemia | European Union | 8875.01 (8279.74-9364.43) | 1.45 (1.35-1.53) | 11932.12 (10279.38-13039.95) | 1.03 (0.9-1.12) | -1.11 (-1.24--0.99) |
| Chronic lymphoid leukemia | Four World Regions | 29196.73 (25648.22-31752.1) | 0.83 (0.74-0.9) | 45459.27 (37590.2-50956.01) | 0.55 (0.46-0.62) | -1.45 (-1.56--1.34) |
| Chronic lymphoid leukemia | G20 | 24798.22 (22181.4-26870.1) | 0.91 (0.82-0.99) | 36770.35 (31375.52-41268.62) | 0.58 (0.49-0.65) | -1.64 (-1.75--1.52) |
| Chronic lymphoid leukemia | Gulf Cooperation Council | 32.48 (19.41-66.09) | 0.5 (0.3-1) | 147.24 (97.06-265.69) | 0.64 (0.44-1.06) | 1.12 (0.83-1.41) |
| Chronic lymphoid leukemia | Health System Grouping Levels | 29236.71 (25690.55-31792.58) | 0.83 (0.74-0.9) | 45507.34 (37634.38-51014.16) | 0.55 (0.46-0.62) | -1.45 (-1.56--1.34) |
| Chronic lymphoid leukemia | High SDI | 14137.39 (13128.32-14772.26) | 1.26 (1.17-1.32) | 17960.85 (15598.07-19401.48) | 0.73 (0.65-0.79) | -1.88 (-2.05--1.71) |
| Chronic lymphoid leukemia | High-income | 15781 (14694.89-16513.27) | 1.29 (1.2-1.35) | 19728.48 (16907.99-21369.79) | 0.74 (0.64-0.8) | -1.94 (-2.11--1.77) |
| Chronic lymphoid leukemia | High-middle SDI | 8300.35 (7452.14-9157.51) | 0.89 (0.8-0.98) | 12978.17 (10852.31-14996.71) | 0.67 (0.56-0.78) | -1.1 (-1.25--0.94) |
| Chronic lymphoid leukemia | Latin America and Caribbean | 902.42 (843.33-958.73) | 0.45 (0.42-0.48) | 2483.65 (2249.52-2695.39) | 0.42 (0.38-0.45) | -0.27 (-0.37--0.16) |
| Chronic lymphoid leukemia | Low SDI | 1099.63 (633.25-1452.51) | 0.55 (0.32-0.73) | 2267.83 (1173.46-3204.76) | 0.53 (0.29-0.75) | -0.2 (-0.28--0.13) |
| Chronic lymphoid leukemia | Low-middle SDI | 1018.83 (563.84-1287.88) | 0.19 (0.11-0.24) | 2863.68 (1607.22-3550.83) | 0.22 (0.13-0.27) | 0.53 (0.43-0.62) |
| Chronic lymphoid leukemia | Middle SDI | 4680.5 (2864.78-5779.68) | 0.46 (0.3-0.56) | 9436.81 (6371.93-12161.44) | 0.37 (0.26-0.48) | -0.73 (-0.8--0.67) |
| Chronic lymphoid leukemia | Nordic Region | 577.88 (533.25-615.53) | 1.43 (1.32-1.52) | 651.05 (555.31-725.54) | 0.95 (0.82-1.06) | -1.41 (-1.52--1.29) |
| Chronic lymphoid leukemia | North Africa and Middle East | 1192.08 (509-1608.71) | 0.84 (0.38-1.13) | 3119.32 (1408.48-4024.66) | 0.81 (0.37-1.03) | 0.07 (-0.03-0.18) |
| Chronic lymphoid leukemia | OECD Countries | 17472.58 (16302.27-18251.46) | 1.29 (1.2-1.35) | 22854.34 (19928.22-24610.8) | 0.77 (0.68-0.83) | -1.79 (-1.94--1.64) |
| Chronic lymphoid leukemia | Organization of Islamic Cooperation | 1814.42 (959.12-2326.17) | 0.4 (0.22-0.52) | 4608.89 (2413.1-5715.34) | 0.43 (0.23-0.54) | 0.44 (0.38-0.51) |
| Chronic lymphoid leukemia | Sahel Region | 219.21 (100.7-310.03) | 0.4 (0.19-0.56) | 550.04 (225.64-748.68) | 0.47 (0.2-0.65) | 0.68 (0.63-0.73) |
| Chronic lymphoid leukemia | South Asia | 383.74 (146.2-536.17) | 0.08 (0.03-0.11) | 1140.62 (575.92-1604.85) | 0.09 (0.04-0.12) | 0.18 (0.08-0.28) |
| Chronic lymphoid leukemia | Southeast Asia, East Asia, and Oceania | 5389.45 (3085.87-7221.9) | 0.45 (0.27-0.6) | 9866 (6355.88-14248.37) | 0.36 (0.24-0.53) | -0.79 (-0.89--0.7) |
| Chronic lymphoid leukemia | Sub-Saharan Africa | 1342.28 (794.69-1743.53) | 0.72 (0.44-0.93) | 3017.7 (1728.52-4001.97) | 0.76 (0.45-1.01) | 0.18 (0.15-0.21) |
| Chronic lymphoid leukemia | WHO region | 29178.52 (25629.77-31733.85) | 0.83 (0.74-0.9) | 45388.9 (37518.34-50885.39) | 0.55 (0.46-0.62) | -1.45 (-1.56--1.34) |
| Chronic lymphoid leukemia | World Bank Income Levels | 29236.7 (25690.55-31792.57) | 0.83 (0.74-0.9) | 45507.34 (37634.37-51014.16) | 0.55 (0.46-0.62) | -1.45 (-1.56--1.34) |
| Chronic lymphoid leukemia | World Bank Regions | 29214.03 (25676.23-31766.92) | 0.83 (0.74-0.9) | 45462.93 (37593.61-50946.62) | 0.55 (0.46-0.62) | -1.45 (-1.56--1.34) |
| Chronic myeloid leukemia | American Samoa | 0.1 (0-0.3) | 0.2 (0.1-1.1) | 0.5 (0.2-0.7) | 1 (0.5-1.4) | 0.18 (-0.62-1) |
| Chronic myeloid leukemia | Antigua and Barbuda | 0.9 (0.8-1) | 1.6 (1.4-1.7) | 0.9 (0.8-1) | 0.9 (0.8-1) | -0.2 (-0.97-0.58) |
| Chronic myeloid leukemia | Arab Republic of Egypt | 182.6 (97.7-397.8) | 0.5 (0.3-1.4) | 344.9 (165.3-668.4) | 0.5 (0.3-1.2) | 0.92 (0.11-1.74) |
| Chronic myeloid leukemia | Argentine Republic | 379.8 (320-475.5) | 1.2 (1-1.5) | 201.1 (168.6-232.4) | 0.4 (0.3-0.4) | -0.52 (-1.27-0.24) |
| Chronic myeloid leukemia | Australia | 217.8 (199.9-239) | 1.1 (1-1.2) | 146.4 (120.3-174.3) | 0.3 (0.3-0.4) | -0.68 (-1.46-0.1) |
| Chronic myeloid leukemia | Barbados | 1.8 (1.6-2.1) | 0.6 (0.6-0.7) | 2.5 (1.9-3.1) | 0.5 (0.4-0.6) | -0.03 (-0.83-0.77) |
| Chronic myeloid leukemia | Belize | 0.4 (0.3-0.5) | 0.4 (0.3-0.4) | 1 (0.9-1.1) | 0.3 (0.3-0.4) | -0.03 (-0.62-0.57) |
| Chronic myeloid leukemia | Bermuda | 0.5 (0.4-0.6) | 0.8 (0.6-0.9) | 0.4 (0.3-0.4) | 0.3 (0.2-0.4) | -0.87 (-1.63--0.11) |
| Chronic myeloid leukemia | Bolivarian Republic of Venezuela | 114.3 (103.7-132) | 1 (0.9-1.1) | 135.7 (97.1-183.4) | 0.5 (0.3-0.6) | -0.22 (-0.94-0.51) |
| Chronic myeloid leukemia | Bosnia and Herzegovina | 10.9 (3.2-27.5) | 0.3 (0.1-0.7) | 13.3 (3.8-27.5) | 0.2 (0.1-0.5) | -0.03 (-0.73-0.67) |
| Chronic myeloid leukemia | Brunei Darussalam | 1.1 (0.4-1.9) | 0.6 (0.2-1) | 1.1 (0.3-1.7) | 0.2 (0.1-0.4) | -0.39 (-1.21-0.45) |
| Chronic myeloid leukemia | Burkina Faso | 4 (1.3-11) | 0.1 (0-0.2) | 6.9 (2.9-19) | 0 (0-0.1) | 0.26 (-0.12-0.65) |
| Chronic myeloid leukemia | Canada | 334.5 (302.5-364.1) | 1 (0.9-1.1) | 183.6 (155.2-213.5) | 0.3 (0.2-0.3) | -0.6 (-1.44-0.24) |
| Chronic myeloid leukemia | Central African Republic | 2.2 (0.9-4.3) | 0.1 (0.1-0.3) | 3.1 (1.1-6) | 0.1 (0-0.2) | -0.06 (-0.65-0.54) |
| Chronic myeloid leukemia | Commonwealth of Dominica | 0.5 (0.2-0.8) | 0.8 (0.3-1.3) | 0.6 (0.2-0.9) | 0.7 (0.2-1.1) | 0.07 (-0.69-0.83) |
| Chronic myeloid leukemia | Commonwealth of the Bahamas | 1.4 (1.2-1.8) | 0.8 (0.7-1) | 2 (1.6-2.5) | 0.5 (0.4-0.6) | -0.14 (-0.76-0.5) |
| Chronic myeloid leukemia | Cook Islands | 0.1 (0-0.1) | 0.4 (0.3-0.6) | 0 (0-0.1) | 0.2 (0.1-0.3) | -0.42 (-0.88-0.03) |
| Chronic myeloid leukemia | Czech Republic | 75.3 (63.1-90.5) | 0.6 (0.5-0.7) | 48.5 (35-67.7) | 0.2 (0.2-0.3) | -0.7 (-1.46-0.08) |
| Chronic myeloid leukemia | Democratic People's Republic of Korea | 68.3 (35.7-113.7) | 0.4 (0.2-0.6) | 72.1 (36.2-141.5) | 0.2 (0.1-0.4) | -0.24 (-1.03-0.56) |
| Chronic myeloid leukemia | Democratic Republic of Sao Tome and Principe | 0 (0-0.1) | 0 (0-0.1) | 0 (0-0.1) | 0 (0-0.1) | -0.1 (-0.38-0.18) |
| Chronic myeloid leukemia | Democratic Republic of the Congo | 19.9 (7.6-40.6) | 0.1 (0-0.2) | 29.6 (9.8-60.5) | 0.1 (0-0.1) | 0.02 (-0.51-0.54) |
| Chronic myeloid leukemia | Democratic Republic of Timor-Leste | 3.3 (1.4-6.7) | 0.8 (0.3-1.4) | 4.5 (2.3-8.3) | 0.5 (0.2-0.9) | -0.3 (-1.08-0.49) |
| Chronic myeloid leukemia | Democratic Socialist Republic of Sri Lanka | 49.1 (27.1-92.1) | 0.4 (0.2-0.8) | 68 (33.5-116) | 0.3 (0.1-0.5) | -0.77 (-1.55-0.01) |
| Chronic myeloid leukemia | Dominican Republic | 11.6 (5.6-17.1) | 0.3 (0.1-0.4) | 21.9 (10.4-34.3) | 0.2 (0.1-0.3) | -0.06 (-0.7-0.57) |
| Chronic myeloid leukemia | Eastern Republic of Uruguay | 46.9 (40.1-54.6) | 1.3 (1.1-1.5) | 27.4 (22.9-32.5) | 0.5 (0.4-0.6) | -0.36 (-1.13-0.42) |
| Chronic myeloid leukemia | Federal Democratic Republic of Ethiopia | 139.2 (56.7-286.4) | 0.5 (0.2-1.1) | 139.1 (51.7-385.3) | 0.2 (0.1-0.6) | -1.11 (-2.01--0.19) |
| Chronic myeloid leukemia | Federal Democratic Republic of Nepal | 72.9 (37.1-124.4) | 0.6 (0.3-1.2) | 95.5 (48.2-169.3) | 0.4 (0.2-0.8) | -0.23 (-0.85-0.4) |
| Chronic myeloid leukemia | Federal Republic of Germany | 1786.3 (1497.2-2274) | 1.4 (1.2-1.8) | 820 (665.1-965.5) | 0.4 (0.3-0.4) | -0.55 (-1.36-0.27) |
| Chronic myeloid leukemia | Federal Republic of Nigeria | 26.9 (10-65.6) | 0 (0-0.1) | 35.4 (16.4-65.1) | 0 (0-0.1) | -0.02 (-0.38-0.34) |
| Chronic myeloid leukemia | Federal Republic of Somalia | 8 (3.3-16.5) | 0.2 (0.1-0.5) | 19.4 (7.5-48.3) | 0.2 (0.1-0.5) | 0.19 (-0.39-0.77) |
| Chronic myeloid leukemia | Federated States of Micronesia | 0.9 (0.6-1.3) | 1.6 (1-2.2) | 1.1 (0.7-1.6) | 1.3 (0.8-1.9) | -0.28 (-1.15-0.59) |
| Chronic myeloid leukemia | Federative Republic of Brazil | 829.7 (783.3-875.3) | 0.8 (0.8-0.9) | 797.2 (723.9-884.5) | 0.3 (0.3-0.4) | -0.3 (-1.02-0.43) |
| Chronic myeloid leukemia | French Republic | 982.1 (888.6-1072) | 1.2 (1.1-1.3) | 559.3 (448.6-679) | 0.3 (0.3-0.4) | -0.61 (-1.42-0.2) |
| Chronic myeloid leukemia | Gabonese Republic | 0.6 (0.3-1.4) | 0.1 (0-0.2) | 0.7 (0.3-1.4) | 0.1 (0-0.1) | -0.02 (-0.6-0.57) |
| Chronic myeloid leukemia | Georgia | 23.8 (14.9-34.7) | 0.4 (0.2-0.6) | 14.1 (9.4-19.6) | 0.3 (0.2-0.4) | -0.01 (-0.71-0.7) |
| Chronic myeloid leukemia | Grand Duchy of Luxembourg | 6.6 (6.1-7.1) | 1.3 (1.2-1.3) | 2.9 (2.5-3.3) | 0.3 (0.2-0.3) | -0.95 (-1.9-0) |
| Chronic myeloid leukemia | Greenland | 0.1 (0-0.2) | 0.4 (0.1-0.6) | 0.1 (0-0.2) | 0.2 (0-0.3) | -0.64 (-1.29-0.03) |
| Chronic myeloid leukemia | Grenada | 0.6 (0.5-0.7) | 0.8 (0.6-1) | 0.6 (0.5-0.7) | 0.5 (0.5-0.6) | -0.03 (-0.68-0.62) |
| Chronic myeloid leukemia | Guam | 1.1 (0.8-1.5) | 1.3 (1-1.8) | 1.5 (1.1-1.9) | 0.7 (0.6-1) | -0.36 (-1.11-0.38) |
| Chronic myeloid leukemia | Hashemite Kingdom of Jordan | 14.3 (8.1-22.9) | 1 (0.6-1.7) | 19.4 (10.9-33.9) | 0.3 (0.2-0.5) | -0.79 (-1.83-0.26) |
| Chronic myeloid leukemia | Hellenic Republic | 185.2 (170.3-201.3) | 1.3 (1.2-1.4) | 116.2 (100.3-130.3) | 0.4 (0.4-0.5) | -0.69 (-1.52-0.14) |
| Chronic myeloid leukemia | Hungary | 156.7 (138.5-178.6) | 1.1 (1-1.3) | 69.4 (53.7-89.6) | 0.4 (0.3-0.5) | -0.82 (-1.6--0.03) |
| Chronic myeloid leukemia | Independent State of Papua New Guinea | 27.4 (13.9-45.2) | 1.1 (0.6-1.7) | 62.4 (33.3-97.5) | 0.9 (0.5-1.4) | -0.21 (-0.95-0.53) |
| Chronic myeloid leukemia | Independent State of Samoa | 1.6 (1-2.4) | 1.7 (1-2.5) | 2.5 (1.5-3.5) | 1.6 (1-2.3) | -0.24 (-1.16-0.68) |
| Chronic myeloid leukemia | Ireland | 42.5 (38.8-46.2) | 1.1 (1-1.1) | 13.8 (11.6-16.5) | 0.2 (0.1-0.2) | -1.01 (-1.81--0.19) |
| Chronic myeloid leukemia | Islamic Republic of Afghanistan | 113.6 (31.9-236.3) | 1.5 (0.4-2.9) | 175.1 (69.1-311.3) | 1.1 (0.5-2) | -0.2 (-1.14-0.75) |
| Chronic myeloid leukemia | Islamic Republic of Iran | 333.6 (67.3-476.9) | 1 (0.3-1.4) | 399.7 (94.9-576.8) | 0.5 (0.1-0.8) | -0.61 (-1.39-0.17) |
| Chronic myeloid leukemia | Islamic Republic of Mauritania | 0.5 (0.2-1.1) | 0 (0-0.1) | 0.6 (0.2-1.4) | 0 (0-0.1) | 0.12 (-0.24-0.47) |
| Chronic myeloid leukemia | Islamic Republic of Pakistan | 398.5 (230.5-650.3) | 0.6 (0.3-1) | 641.8 (395.7-981.2) | 0.5 (0.3-0.7) | -0.15 (-0.86-0.56) |
| Chronic myeloid leukemia | Jamaica | 5.4 (4.1-6.6) | 0.3 (0.2-0.4) | 9.4 (7-12.6) | 0.3 (0.2-0.4) | 0.12 (-0.59-0.83) |
| Chronic myeloid leukemia | Japan | 1196.1 (1141.2-1243.6) | 0.8 (0.7-0.8) | 656.3 (536-737.5) | 0.2 (0.1-0.2) | -0.53 (-1.28-0.23) |
| Chronic myeloid leukemia | Kingdom of Bahrain | 1.9 (1-3) | 1.1 (0.6-1.9) | 3 (1.1-5.3) | 0.4 (0.2-0.8) | -1.23 (-2.22--0.23) |
| Chronic myeloid leukemia | Kingdom of Belgium | 159.3 (135.7-180.9) | 1.1 (0.9-1.2) | 65.9 (53.5-80.4) | 0.3 (0.2-0.3) | -0.78 (-1.6-0.05) |
| Chronic myeloid leukemia | Kingdom of Bhutan | 1.9 (0.9-3.6) | 0.6 (0.3-1) | 2 (0.9-4.3) | 0.3 (0.2-0.7) | -0.34 (-0.99-0.32) |
| Chronic myeloid leukemia | Kingdom of Cambodia | 73 (35.6-126) | 1.2 (0.6-2.1) | 103.5 (52.2-174.9) | 0.8 (0.4-1.3) | -0.48 (-1.35-0.4) |
| Chronic myeloid leukemia | Kingdom of Denmark | 65.5 (57.8-72.6) | 0.8 (0.7-0.9) | 46.9 (38.8-57.4) | 0.4 (0.3-0.5) | -0.74 (-1.6-0.13) |
| Chronic myeloid leukemia | Kingdom of Eswatini | 0.2 (0-0.4) | 0 (0-0.1) | 0.3 (0.1-0.5) | 0 (0-0.1) | 0.34 (-0.51-1.21) |
| Chronic myeloid leukemia | Kingdom of Lesotho | 0.3 (0.1-0.7) | 0 (0-0.1) | 0.6 (0.2-1) | 0 (0-0.1) | 0.94 (0.14-1.74) |
| Chronic myeloid leukemia | Kingdom of Morocco | 23.8 (13.4-43) | 0.1 (0.1-0.3) | 29.2 (13.9-61.3) | 0.1 (0-0.2) | -0.08 (-0.46-0.29) |
| Chronic myeloid leukemia | Kingdom of Norway | 36.3 (33.4-39.1) | 0.5 (0.5-0.6) | 14.7 (12.7-17) | 0.1 (0.1-0.2) | -0.33 (-1.13-0.48) |
| Chronic myeloid leukemia | Kingdom of Saudi Arabia | 42 (17-118.9) | 0.6 (0.2-1.7) | 116.2 (54.8-351.1) | 0.5 (0.2-1.5) | 0.26 (-0.34-0.87) |
| Chronic myeloid leukemia | Kingdom of Spain | 524.7 (474.2-569.6) | 1 (0.9-1.1) | 214.6 (171.3-259.8) | 0.2 (0.2-0.2) | -0.8 (-1.53--0.07) |
| Chronic myeloid leukemia | Kingdom of Sweden | 98.6 (87.8-109.9) | 0.7 (0.6-0.8) | 40.9 (33.3-48.7) | 0.2 (0.2-0.2) | -0.55 (-1.35-0.26) |
| Chronic myeloid leukemia | Kingdom of Thailand | 364.5 (169.6-542.9) | 0.9 (0.4-1.4) | 449.7 (202-695.9) | 0.5 (0.2-0.7) | -0.59 (-1.45-0.27) |
| Chronic myeloid leukemia | Kingdom of the Netherlands | 165.2 (147.4-180.1) | 0.8 (0.7-0.9) | 79.5 (67.1-93.3) | 0.2 (0.2-0.3) | -0.65 (-1.48-0.19) |
| Chronic myeloid leukemia | Kingdom of Tonga | 0.3 (0.2-0.6) | 0.5 (0.3-0.9) | 0.4 (0.2-0.5) | 0.4 (0.3-0.6) | -0.13 (-0.88-0.62) |
| Chronic myeloid leukemia | Kyrgyz Republic | 17.8 (14.6-20.9) | 0.5 (0.4-0.6) | 11.8 (9.1-14.9) | 0.2 (0.2-0.3) | -0.67 (-1.25--0.1) |
| Chronic myeloid leukemia | Lao People's Democratic Republic | 36.1 (14.3-65.8) | 1.4 (0.6-2.3) | 33.2 (17.6-61.1) | 0.6 (0.3-1.1) | -0.77 (-1.63-0.1) |
| Chronic myeloid leukemia | Lebanese Republic | 17.6 (8.4-28.7) | 0.9 (0.4-1.4) | 28.1 (10.2-48.6) | 0.4 (0.2-0.7) | -0.36 (-1.17-0.46) |
| Chronic myeloid leukemia | Malaysia | 37.9 (18.5-61.4) | 0.3 (0.2-0.5) | 61 (27.6-101) | 0.2 (0.1-0.4) | -0.31 (-1.14-0.53) |
| Chronic myeloid leukemia | Mongolia | 3.6 (1.7-6.7) | 0.2 (0.1-0.5) | 4.5 (2.4-7.2) | 0.2 (0.1-0.3) | -0.43 (-1.11-0.26) |
| Chronic myeloid leukemia | Montenegro | 2 (0.6-4.2) | 0.3 (0.1-0.7) | 1.9 (0.7-3.1) | 0.2 (0.1-0.3) | -0.12 (-0.87-0.64) |
| Chronic myeloid leukemia | New Zealand | 38 (34.5-41.7) | 1 (0.9-1.1) | 16.4 (14-19) | 0.2 (0.2-0.2) | -0.58 (-1.44-0.28) |
| Chronic myeloid leukemia | North Macedonia | 0 (0-0) | 0 (0-0) | 0 (0-0) | 0 (0-0) | -0.23 (-1.25-0.79) |
| Chronic myeloid leukemia | Northern Mariana Islands | 0.2 (0.1-0.4) | 0.8 (0.6-1.3) | 0.3 (0.2-0.4) | 0.6 (0.5-0.9) | -0.52 (-1.28-0.24) |
| Chronic myeloid leukemia | Palestine | 6.7 (3.9-11.8) | 0.7 (0.4-1.3) | 8.7 (5.3-13.7) | 0.4 (0.2-0.6) | -0.8 (-1.71-0.11) |
| Chronic myeloid leukemia | People's Democratic Republic of Algeria | 46.3 (21.1-69.8) | 0.4 (0.2-0.5) | 63.6 (26.2-105.4) | 0.2 (0.1-0.4) | -0.28 (-0.92-0.36) |
| Chronic myeloid leukemia | People's Republic of Bangladesh | 463.4 (241.2-723.9) | 0.7 (0.4-1.1) | 435.5 (184.4-826.5) | 0.3 (0.1-0.6) | -0.59 (-1.24-0.06) |
| Chronic myeloid leukemia | People's Republic of China | 3591.5 (1688.5-5239.8) | 0.4 (0.2-0.5) | 1840.7 (1080.2-3094.1) | 0.1 (0.1-0.2) | -1.08 (-1.87--0.29) |
| Chronic myeloid leukemia | Plurinational State of Bolivia | 37.6 (20-62.3) | 1 (0.5-1.5) | 52.6 (29.8-80.1) | 0.6 (0.3-0.9) | -0.42 (-1.3-0.48) |
| Chronic myeloid leukemia | Portuguese Republic | 137.3 (123.7-150.7) | 1.1 (1-1.2) | 68.3 (55-82.9) | 0.3 (0.2-0.3) | -0.79 (-1.59-0.01) |
| Chronic myeloid leukemia | Principality of Andorra | 0.5 (0.2-0.8) | 0.9 (0.4-1.5) | 0.4 (0.2-0.9) | 0.3 (0.1-0.6) | -0.9 (-1.78--0.01) |
| Chronic myeloid leukemia | Principality of Monaco | 0.5 (0.2-0.9) | 0.8 (0.4-1.3) | 0.5 (0.2-0.9) | 0.5 (0.3-1) | 0.1 (-0.95-1.17) |
| Chronic myeloid leukemia | Puerto Rico | 33.4 (28.6-38.7) | 0.9 (0.8-1.1) | 25.9 (20.7-32.2) | 0.4 (0.3-0.4) | -0.7 (-1.46-0.06) |
| Chronic myeloid leukemia | Republic of Albania | 5.6 (1.5-13.9) | 0.3 (0.1-0.7) | 6.9 (1.9-16.6) | 0.2 (0-0.4) | -0.39 (-1.07-0.3) |
| Chronic myeloid leukemia | Republic of Angola | 7 (2.4-17.5) | 0.1 (0-0.2) | 12 (4.4-27.4) | 0.1 (0-0.2) | -0.09 (-0.64-0.47) |
| Chronic myeloid leukemia | Republic of Armenia | 23.1 (18-28.6) | 0.8 (0.6-1) | 11.1 (8.9-13.7) | 0.3 (0.2-0.3) | -0.62 (-1.3-0.06) |
| Chronic myeloid leukemia | Republic of Austria | 124.7 (114.1-137.6) | 1.1 (1-1.2) | 60 (48.3-71.5) | 0.3 (0.3-0.4) | -0.49 (-1.3-0.34) |
| Chronic myeloid leukemia | Republic of Azerbaijan | 23.3 (11.8-41.8) | 0.4 (0.2-0.7) | 26 (10.2-54.4) | 0.2 (0.1-0.5) | -0.64 (-1.38-0.1) |
| Chronic myeloid leukemia | Republic of Belarus | 63.7 (40.9-92.5) | 0.5 (0.3-0.8) | 53.5 (39.7-69.4) | 0.4 (0.3-0.5) | -0.69 (-1.4-0.03) |
| Chronic myeloid leukemia | Republic of Benin | 1.5 (0.5-3.7) | 0 (0-0.1) | 2.7 (1-6.7) | 0 (0-0.1) | 0.2 (-0.18-0.58) |
| Chronic myeloid leukemia | Republic of Botswana | 0.3 (0.1-0.8) | 0 (0-0.1) | 0.4 (0.1-1.2) | 0 (0-0.1) | -0.07 (-0.81-0.67) |
| Chronic myeloid leukemia | Republic of Bulgaria | 60.1 (42.8-81.1) | 0.6 (0.4-0.7) | 29.3 (20.4-41.2) | 0.2 (0.2-0.3) | 0.05 (-0.67-0.78) |
| Chronic myeloid leukemia | Republic of Burundi | 7.2 (3-16.9) | 0.2 (0.1-0.5) | 10.1 (3.6-25.1) | 0.1 (0.1-0.4) | -0.13 (-0.68-0.42) |
| Chronic myeloid leukemia | Republic of Cabo Verde | 0.2 (0.1-0.7) | 0.1 (0-0.3) | 0.3 (0.1-0.9) | 0.1 (0-0.2) | 0.14 (-0.46-0.75) |
| Chronic myeloid leukemia | Republic of Cameroon | 3.1 (1.2-6.8) | 0 (0-0.1) | 6.8 (3.2-12.9) | 0 (0-0.1) | 0.23 (-0.18-0.64) |
| Chronic myeloid leukemia | Republic of Chad | 1.9 (0.6-4.7) | 0 (0-0.1) | 4.6 (1.8-10.3) | 0 (0-0.1) | 0.39 (0.02-0.77) |
| Chronic myeloid leukemia | Republic of Chile | 98.8 (89.9-108.3) | 1 (0.9-1.1) | 83.4 (71.5-97.6) | 0.3 (0.3-0.4) | -0.41 (-1.09-0.27) |
| Chronic myeloid leukemia | Republic of Colombia | 175.1 (158.4-190.5) | 0.8 (0.7-0.9) | 204.7 (166.3-251.4) | 0.4 (0.3-0.5) | -0.44 (-1.19-0.31) |
| Chronic myeloid leukemia | Republic of Costa Rica | 23.5 (20.3-27.2) | 1.2 (1.1-1.4) | 32.3 (27.4-38.5) | 0.6 (0.5-0.7) | -0.11 (-0.88-0.68) |
| Chronic myeloid leukemia | République de Côte d'Ivoire | 2 (0.8-4.9) | 0 (0-0.1) | 3.7 (1.3-9.1) | 0 (0-0.1) | -0.02 (-0.32-0.28) |
| Chronic myeloid leukemia | Republic of Croatia | 53.9 (41.5-69.3) | 0.9 (0.7-1.2) | 23.1 (15.6-31.8) | 0.3 (0.2-0.4) | -0.54 (-1.3-0.23) |
| Chronic myeloid leukemia | Republic of Cuba | 129.4 (118.4-144.7) | 1.3 (1.1-1.4) | 146 (122.5-169.2) | 0.8 (0.6-0.9) | -0.44 (-1.14-0.26) |
| Chronic myeloid leukemia | Republic of Cyprus | 3.4 (2-5.6) | 0.5 (0.3-0.9) | 3.8 (2-6.1) | 0.2 (0.1-0.3) | -0.67 (-1.65-0.33) |
| Chronic myeloid leukemia | Republic of Djibouti | 0.3 (0.1-0.7) | 0.1 (0.1-0.4) | 0.9 (0.3-2.5) | 0.1 (0-0.3) | 0.21 (-0.35-0.77) |
| Chronic myeloid leukemia | Republic of Ecuador | 26.5 (22.3-32.6) | 0.4 (0.4-0.5) | 65.3 (50.2-84.8) | 0.4 (0.3-0.5) | 0.33 (-0.52-1.18) |
| Chronic myeloid leukemia | Republic of El Salvador | 10.7 (5.7-15.3) | 0.3 (0.2-0.4) | 14.2 (7.6-20.6) | 0.2 (0.1-0.3) | -0.02 (-0.83-0.79) |
| Chronic myeloid leukemia | Republic of Equatorial Guinea | 0.3 (0.1-0.5) | 0.1 (0-0.2) | 0.4 (0.1-1) | 0.1 (0-0.1) | -0.19 (-0.72-0.35) |
| Chronic myeloid leukemia | Republic of Estonia | 23.6 (19-29.1) | 1.2 (1-1.5) | 8.1 (6.4-10.3) | 0.3 (0.2-0.4) | -1 (-1.8--0.2) |
| Chronic myeloid leukemia | Republic of Fiji | 3.1 (1.2-5) | 0.7 (0.3-1.1) | 3.1 (1.5-4.8) | 0.4 (0.2-0.6) | -0.32 (-1.45-0.82) |
| Chronic myeloid leukemia | Republic of Finland | 40.5 (36.6-45) | 0.6 (0.5-0.6) | 27.2 (22.2-32.8) | 0.2 (0.2-0.2) | -0.61 (-1.32-0.11) |
| Chronic myeloid leukemia | Republic of Ghana | 6.5 (3.1-13.2) | 0.1 (0-0.1) | 6.2 (2.2-16.6) | 0 (0-0.1) | -0.75 (-1.24--0.27) |
| Chronic myeloid leukemia | Republic of Guatemala | 18.5 (15.1-22) | 0.4 (0.3-0.5) | 24 (20.6-28.2) | 0.2 (0.2-0.2) | -0.01 (-0.78-0.77) |
| Chronic myeloid leukemia | Republic of Guinea | 1.1 (0.3-2.9) | 0 (0-0) | 1.1 (0.3-2.7) | 0 (0-0) | -0.02 (-0.21-0.16) |
| Chronic myeloid leukemia | Republic of Guinea-Bissau | 0.4 (0.2-1.1) | 0.1 (0-0.2) | 0.6 (0.3-1.4) | 0.1 (0-0.1) | 0.24 (-0.2-0.68) |
| Chronic myeloid leukemia | Republic of Guyana | 5.8 (5-6.6) | 1.2 (1.1-1.4) | 4.9 (3.6-6.8) | 0.7 (0.5-1) | 0.13 (-0.52-0.78) |
| Chronic myeloid leukemia | Republic of Haiti | 62.9 (20.5-124.7) | 1.4 (0.6-2.3) | 75.4 (33.1-126) | 0.9 (0.4-1.4) | -0.34 (-1.08-0.41) |
| Chronic myeloid leukemia | Republic of Honduras | 18.8 (10.1-26.1) | 0.7 (0.4-1) | 37.6 (21.7-58.8) | 0.6 (0.3-0.9) | -0.13 (-0.92-0.67) |
| Chronic myeloid leukemia | Republic of Iceland | 1.6 (1.5-1.8) | 0.6 (0.5-0.6) | 1 (0.8-1.2) | 0.2 (0.1-0.2) | -0.35 (-1.12-0.43) |
| Chronic myeloid leukemia | Republic of India | 2889.7 (1809.7-4116.4) | 0.5 (0.3-0.8) | 4184.9 (2493.7-6004.5) | 0.4 (0.2-0.5) | -0.31 (-0.92-0.29) |
| Chronic myeloid leukemia | Republic of Indonesia | 750.5 (380.2-1150.2) | 0.6 (0.3-0.9) | 872.5 (418-1450.6) | 0.4 (0.2-0.6) | -0.16 (-0.99-0.69) |
| Chronic myeloid leukemia | Republic of Iraq | 289.5 (153-413.4) | 2.8 (1.5-4) | 584.5 (227.4-851.7) | 2.4 (1-3.3) | -0.36 (-1.22-0.5) |
| Chronic myeloid leukemia | Republic of Italy | 1099.9 (1029.8-1165.8) | 1.3 (1.2-1.4) | 529.7 (435.9-616.6) | 0.3 (0.3-0.4) | -0.84 (-1.62--0.04) |
| Chronic myeloid leukemia | Republic of Kazakhstan | 63.8 (49-86.4) | 0.5 (0.3-0.6) | 37.1 (27.1-53) | 0.2 (0.2-0.3) | -0.54 (-1.21-0.12) |
| Chronic myeloid leukemia | Republic of Kenya | 15.1 (7.8-31.9) | 0.1 (0.1-0.3) | 31.7 (16.8-60.9) | 0.1 (0.1-0.2) | 0.29 (-0.18-0.76) |
| Chronic myeloid leukemia | Republic of Kiribati | 0.5 (0.3-0.8) | 1 (0.7-1.6) | 0.8 (0.6-1.2) | 1 (0.7-1.5) | -0.04 (-0.76-0.68) |
| Chronic myeloid leukemia | Republic of Korea | 275.3 (145.9-375) | 0.8 (0.5-1) | 124.6 (66.6-218.9) | 0.2 (0.1-0.3) | -1 (-1.72--0.27) |
| Chronic myeloid leukemia | Republic of Latvia | 30.6 (22.9-39.9) | 0.9 (0.7-1.1) | 13.2 (9.9-17.8) | 0.4 (0.3-0.5) | -0.81 (-1.58--0.03) |
| Chronic myeloid leukemia | Republic of Liberia | 1 (0.4-2.5) | 0 (0-0.1) | 1.2 (0.5-2.5) | 0 (0-0.1) | 0.23 (-0.14-0.6) |
| Chronic myeloid leukemia | Republic of Lithuania | 52.8 (41.6-66.2) | 1.2 (1-1.5) | 23.1 (18.1-29.4) | 0.4 (0.3-0.5) | -0.54 (-1.31-0.25) |
| Chronic myeloid leukemia | Republic of Madagascar | 9.3 (4.9-16.9) | 0.1 (0.1-0.3) | 15.5 (6.8-29.9) | 0.1 (0-0.2) | 0.01 (-0.48-0.5) |
| Chronic myeloid leukemia | Republic of Malawi | 3.1 (1.5-6) | 0.1 (0-0.1) | 4.2 (1.8-8.5) | 0 (0-0.1) | -0.09 (-0.38-0.2) |
| Chronic myeloid leukemia | Republic of Maldives | 0.6 (0.2-1.3) | 0.4 (0.2-0.8) | 0.4 (0.2-0.7) | 0.1 (0.1-0.2) | -1.16 (-1.89--0.42) |
| Chronic myeloid leukemia | Republic of Mali | 4.2 (1.1-10.6) | 0.1 (0-0.1) | 4.5 (1.2-12.1) | 0 (0-0.1) | -0.11 (-0.42-0.2) |
| Chronic myeloid leukemia | Republic of Malta | 4.4 (3.9-5) | 1.1 (0.9-1.2) | 2.3 (1.9-2.9) | 0.2 (0.2-0.3) | -0.66 (-1.46-0.15) |
| Chronic myeloid leukemia | Republic of Mauritius | 11.1 (9.6-12.1) | 1.3 (1.2-1.5) | 5.9 (5.3-6.5) | 0.4 (0.3-0.4) | -0.51 (-1.24-0.22) |
| Chronic myeloid leukemia | Republic of Moldova | 13.3 (11.4-16) | 0.3 (0.3-0.4) | 7.5 (6.6-8.8) | 0.1 (0.1-0.2) | -0.74 (-1.42--0.06) |
| Chronic myeloid leukemia | Republic of Mozambique | 17 (8.2-34.7) | 0.2 (0.1-0.4) | 27.6 (12.3-59.1) | 0.2 (0.1-0.4) | 0.14 (-0.52-0.8) |
| Chronic myeloid leukemia | Republic of Namibia | 0.2 (0.1-0.5) | 0 (0-0.1) | 0.3 (0.1-0.7) | 0 (0-0) | -0.03 (-0.65-0.6) |
| Chronic myeloid leukemia | Republic of Nauru | 0.1 (0.1-0.1) | 1.5 (1-2.1) | 0.1 (0.1-0.2) | 1.7 (1-2.5) | -0.22 (-1.15-0.73) |
| Chronic myeloid leukemia | Republic of Nicaragua | 11.6 (6.9-16.1) | 0.5 (0.3-0.8) | 18.4 (11.4-27.9) | 0.4 (0.2-0.5) | -0.21 (-0.94-0.53) |
| Chronic myeloid leukemia | Republic of Niue | 0 (0-0) | 1 (0.7-1.5) | 0 (0-0) | 1.1 (0.7-1.4) | -0.06 (-0.86-0.74) |
| Chronic myeloid leukemia | Republic of Palau | 0.1 (0.1-0.1) | 0.8 (0.5-1.2) | 0.1 (0.1-0.2) | 0.6 (0.4-0.9) | -0.16 (-0.66-0.36) |
| Chronic myeloid leukemia | Republic of Panama | 10.3 (9.3-11.8) | 0.6 (0.6-0.7) | 15.6 (12.1-19.2) | 0.4 (0.3-0.4) | 0.06 (-0.7-0.83) |
| Chronic myeloid leukemia | Republic of Paraguay | 19 (10.5-27.3) | 0.8 (0.4-1.1) | 33 (18.6-49.3) | 0.6 (0.3-0.8) | 0.24 (-0.47-0.96) |
| Chronic myeloid leukemia | Republic of Peru | 63.3 (40.8-105.7) | 0.4 (0.3-0.7) | 114.3 (56-175.5) | 0.3 (0.2-0.5) | -0.18 (-0.97-0.62) |
| Chronic myeloid leukemia | Republic of Poland | 576.4 (441.6-733.9) | 1.4 (1-1.7) | 190.4 (167.7-213.9) | 0.3 (0.2-0.3) | -0.79 (-1.55--0.02) |
| Chronic myeloid leukemia | Republic of Rwanda | 10.4 (5-20.3) | 0.3 (0.1-0.5) | 10.9 (3.8-27.8) | 0.1 (0.1-0.4) | -0.5 (-1.08-0.08) |
| Chronic myeloid leukemia | Republic of San Marino | 0.2 (0.1-0.4) | 0.7 (0.3-1) | 0.2 (0.1-0.3) | 0.2 (0.1-0.4) | -0.78 (-1.6-0.04) |
| Chronic myeloid leukemia | Republic of Senegal | 2.1 (0.7-5.4) | 0 (0-0.1) | 3 (0.9-8.1) | 0 (0-0.1) | 0.23 (-0.16-0.61) |
| Chronic myeloid leukemia | Republic of Serbia | 38.2 (19.5-54.3) | 0.4 (0.2-0.5) | 29.8 (18.7-44.7) | 0.2 (0.1-0.3) | -0.51 (-1.26-0.25) |
| Chronic myeloid leukemia | Republic of Seychelles | 0.5 (0.3-0.8) | 0.9 (0.5-1.3) | 0.5 (0.3-0.8) | 0.5 (0.3-0.7) | -0.43 (-1.15-0.29) |
| Chronic myeloid leukemia | Republic of Sierra Leone | 1.6 (0.6-4.3) | 0 (0-0.1) | 2.2 (0.9-4.8) | 0 (0-0.1) | 0.26 (-0.11-0.64) |
| Chronic myeloid leukemia | Republic of Singapore | 22.2 (20.5-23.9) | 0.9 (0.8-1) | 12.8 (11.3-14.5) | 0.2 (0.1-0.2) | -0.85 (-1.66--0.04) |
| Chronic myeloid leukemia | Republic of Slovenia | 27.1 (23.2-31.5) | 1.1 (1-1.3) | 21.2 (16.5-26.6) | 0.5 (0.4-0.6) | -0.5 (-1.23-0.23) |
| Chronic myeloid leukemia | Republic of South Africa | 12.5 (5.3-24.8) | 0 (0-0.1) | 14.3 (7.1-26.9) | 0 (0-0.1) | 0.09 (-0.68-0.86) |
| Chronic myeloid leukemia | Republic of South Sudan | 5 (2.3-12) | 0.2 (0.1-0.4) | 8.4 (3.5-19.3) | 0.2 (0.1-0.3) | 0.3 (-0.25-0.86) |
| Chronic myeloid leukemia | Republic of Sudan | 117.4 (39.2-226.4) | 0.8 (0.3-1.7) | 115.7 (55.7-192) | 0.5 (0.2-0.8) | -0.33 (-1.16-0.51) |
| Chronic myeloid leukemia | Republic of Suriname | 1.9 (1.1-2.5) | 0.7 (0.4-0.9) | 2.5 (1.5-3.8) | 0.4 (0.2-0.6) | -0.16 (-0.77-0.45) |
| Chronic myeloid leukemia | Republic of Tajikistan | 13.8 (7.4-22.4) | 0.4 (0.2-0.6) | 11.6 (5.4-21.5) | 0.1 (0.1-0.3) | -0.75 (-1.39--0.1) |
| Chronic myeloid leukemia | Republic of the Congo | 1.4 (0.7-2.9) | 0.1 (0.1-0.2) | 2.3 (1.1-4.9) | 0.1 (0-0.1) | -0.14 (-0.73-0.45) |
| Chronic myeloid leukemia | Republic of the Gambia | 0.1 (0-0.3) | 0 (0-0) | 0.2 (0.1-0.6) | 0 (0-0) | -0.02 (-0.24-0.2) |
| Chronic myeloid leukemia | Republic of the Marshall Islands | 0.3 (0.2-0.4) | 1.2 (0.8-1.7) | 0.5 (0.3-0.8) | 1.2 (0.6-1.8) | -0.05 (-0.88-0.79) |
| Chronic myeloid leukemia | Republic of the Niger | 3.5 (0.8-11.4) | 0 (0-0.1) | 5.1 (1.4-13.2) | 0 (0-0.1) | 0.12 (-0.25-0.5) |
| Chronic myeloid leukemia | Republic of the Philippines | 322.2 (170.4-432.4) | 0.8 (0.4-1.2) | 507.1 (314.6-758.9) | 0.6 (0.4-0.9) | -0.35 (-1.19-0.49) |
| Chronic myeloid leukemia | Republic of the Union of Myanmar | 338.2 (139.6-603.2) | 1.2 (0.5-2) | 239.2 (125.1-408.3) | 0.5 (0.3-0.8) | -0.95 (-1.84--0.07) |
| Chronic myeloid leukemia | Republic of Trinidad and Tobago | 8.3 (7.2-9.1) | 1 (0.8-1.1) | 9.9 (7.5-13) | 0.5 (0.4-0.7) | -0.43 (-1.12-0.27) |
| Chronic myeloid leukemia | Republic of Tunisia | 19.1 (10.5-38) | 0.4 (0.2-0.8) | 26.8 (13.6-67.2) | 0.2 (0.1-0.6) | -0.48 (-1.11-0.16) |
| Chronic myeloid leukemia | Republic of Turkey | 350.3 (182.8-520.7) | 1 (0.5-1.5) | 328.3 (190.2-556.6) | 0.4 (0.2-0.6) | -1.01 (-1.89--0.12) |
| Chronic myeloid leukemia | Republic of Uganda | 9.2 (4.7-20.7) | 0.1 (0.1-0.2) | 22 (8.9-45.5) | 0.1 (0-0.2) | -0.04 (-0.5-0.43) |
| Chronic myeloid leukemia | Republic of Uzbekistan | 58.7 (43.1-82.2) | 0.4 (0.3-0.6) | 52 (35.3-75.8) | 0.2 (0.1-0.2) | -0.68 (-1.33--0.03) |
| Chronic myeloid leukemia | Republic of Vanuatu | 0.8 (0.4-1.3) | 1 (0.6-1.4) | 2.1 (1.2-2.9) | 1 (0.6-1.4) | -0.13 (-0.91-0.65) |
| Chronic myeloid leukemia | Republic of Yemen | 69 (23.3-131.7) | 0.9 (0.4-1.7) | 103 (47.1-158.5) | 0.6 (0.3-1) | -0.28 (-1.07-0.51) |
| Chronic myeloid leukemia | Republic of Zambia | 7.1 (4.1-13.1) | 0.2 (0.1-0.3) | 11 (4.1-23.5) | 0.1 (0-0.2) | -0.05 (-0.63-0.53) |
| Chronic myeloid leukemia | Republic of Zimbabwe | 1.5 (0.4-3.7) | 0 (0-0.1) | 3.5 (1.2-7) | 0 (0-0.1) | 0.62 (-0.21-1.45) |
| Chronic myeloid leukemia | Romania | 111.6 (94.7-134.3) | 0.4 (0.4-0.5) | 57.4 (48.7-67.9) | 0.2 (0.1-0.2) | -0.11 (-0.79-0.57) |
| Chronic myeloid leukemia | Russian Federation | 869.4 (671.3-1069.2) | 0.5 (0.4-0.6) | 696.2 (619.6-794.1) | 0.3 (0.3-0.3) | -0.44 (-1.09-0.21) |
| Chronic myeloid leukemia | Saint Kitts and Nevis | 0.4 (0.4-0.5) | 1.2 (1-1.4) | 0.4 (0.3-0.4) | 0.6 (0.5-0.7) | -0.42 (-1.11-0.28) |
| Chronic myeloid leukemia | Saint Lucia | 1.2 (1-1.3) | 1.3 (1.1-1.5) | 1 (0.8-1.2) | 0.5 (0.4-0.5) | -0.73 (-1.34--0.11) |
| Chronic myeloid leukemia | Saint Vincent and the Grenadines | 0.7 (0.6-0.8) | 0.9 (0.8-1) | 0.7 (0.6-0.8) | 0.5 (0.5-0.6) | -0.29 (-0.95-0.37) |
| Chronic myeloid leukemia | Slovak Republic | 18.5 (10.2-29.4) | 0.3 (0.2-0.5) | 10.4 (5.5-17.9) | 0.1 (0.1-0.2) | -0.52 (-1.32-0.29) |
| Chronic myeloid leukemia | Socialist Republic of Viet Nam | 58.1 (25-94.7) | 0.1 (0.1-0.2) | 95.4 (48.1-173) | 0.1 (0.1-0.2) | -0.39 (-1.08-0.3) |
| Chronic myeloid leukemia | Solomon Islands | 2.2 (0.8-3.8) | 1.2 (0.5-2) | 5.6 (3.1-8.3) | 1.2 (0.7-1.8) | -0.08 (-0.9-0.74) |
| Chronic myeloid leukemia | State of Eritrea | 3.4 (1.6-6.4) | 0.2 (0.1-0.4) | 6 (2.5-12.5) | 0.2 (0.1-0.3) | 0.18 (-0.38-0.75) |
| Chronic myeloid leukemia | State of Israel | 48.3 (40.1-57.1) | 1 (0.9-1.2) | 32.5 (27.3-38.3) | 0.3 (0.2-0.3) | -0.73 (-1.7-0.25) |
| Chronic myeloid leukemia | State of Kuwait | 1.7 (1.3-2.2) | 0.3 (0.2-0.4) | 3.8 (2.9-4.8) | 0.2 (0.1-0.2) | -1.06 (-1.75--0.36) |
| Chronic myeloid leukemia | State of Libya | 14 (7.7-26.7) | 0.6 (0.3-1.3) | 23 (10.6-52.9) | 0.4 (0.2-1) | 0.11 (-0.77-0.99) |
| Chronic myeloid leukemia | State of Qatar | 1.1 (0.4-1.9) | 0.8 (0.4-1.4) | 2.8 (0.9-5.9) | 0.3 (0.1-0.5) | -1.42 (-2.21--0.63) |
| Chronic myeloid leukemia | Sultanate of Oman | 4 (1.8-10.8) | 0.4 (0.2-1.1) | 3 (1.1-10.1) | 0.1 (0-0.5) | -0.44 (-1.2-0.33) |
| Chronic myeloid leukemia | Swiss Confederation | 104 (88.3-128.7) | 1 (0.9-1.3) | 44.4 (35.8-54.8) | 0.2 (0.2-0.3) | -0.68 (-1.44-0.08) |
| Chronic myeloid leukemia | Syrian Arab Republic | 64.1 (37.4-113.2) | 1 (0.5-1.8) | 65.3 (32.5-140) | 0.6 (0.3-1.3) | -0.8 (-1.65-0.06) |
| Chronic myeloid leukemia | Taiwan (Province of China) | 61.1 (36.3-96.2) | 0.4 (0.2-0.6) | 82.3 (67.7-100.3) | 0.2 (0.2-0.3) | 0.19 (-0.59-0.99) |
| Chronic myeloid leukemia | Togolese Republic | 0.9 (0.3-2) | 0 (0-0.1) | 1.8 (0.7-4.1) | 0 (0-0.1) | 0.28 (-0.09-0.66) |
| Chronic myeloid leukemia | Tokelau | 0 (0-0) | 1.2 (0.8-1.7) | 0 (0-0) | 1.1 (0.7-1.5) | -0.17 (-0.97-0.64) |
| Chronic myeloid leukemia | Turkmenistan | 9.5 (7.1-13) | 0.4 (0.3-0.5) | 8.7 (5.6-13.2) | 0.2 (0.1-0.3) | -0.41 (-1.04-0.21) |
| Chronic myeloid leukemia | Tuvalu | 0.1 (0.1-0.2) | 1.6 (1.1-2.3) | 0.1 (0.1-0.2) | 1.1 (0.7-1.5) | -0.34 (-1.16-0.48) |
| Chronic myeloid leukemia | Ukraine | 513.9 (375.1-703) | 0.8 (0.6-1) | 257.7 (183.5-346.2) | 0.4 (0.3-0.5) | -1.13 (-1.8--0.46) |
| Chronic myeloid leukemia | Union of the Comoros | 0.4 (0.2-0.9) | 0.2 (0.1-0.4) | 0.6 (0.2-1.4) | 0.1 (0-0.3) | 0.02 (-0.53-0.57) |
| Chronic myeloid leukemia | United Arab Emirates | 4.7 (2.4-8.2) | 1 (0.5-1.7) | 9.8 (5.2-16.4) | 0.4 (0.2-0.6) | -0.09 (-0.97-0.79) |
| Chronic myeloid leukemia | United Kingdom of Great Britain and Northern Ireland | 720.8 (673.2-762.5) | 0.8 (0.8-0.9) | 256.1 (232.4-274.3) | 0.2 (0.2-0.2) | -0.46 (-1.32-0.41) |
| Chronic myeloid leukemia | United Mexican States | 315.3 (305.9-325.7) | 0.6 (0.6-0.6) | 407.6 (359-453.8) | 0.3 (0.3-0.4) | -0.21 (-1.03-0.62) |
| Chronic myeloid leukemia | United Republic of Tanzania | 24.7 (12.2-52.8) | 0.2 (0.1-0.4) | 40.1 (16-85.8) | 0.1 (0-0.3) | -0.01 (-0.56-0.55) |
| Chronic myeloid leukemia | United States of America | 3632.2 (3433.1-3794.8) | 1.2 (1.1-1.2) | 1845.5 (1645.7-1970.9) | 0.3 (0.3-0.3) | -0.7 (-1.61-0.21) |
| Chronic myeloid leukemia | United States Virgin Islands | 0.4 (0.2-0.5) | 0.4 (0.3-0.6) | 0.3 (0.1-0.4) | 0.2 (0.1-0.3) | -0.54 (-1.26-0.18) |
| Chronic myeloid leukemia | African Union | 782.24 (451.09-1397.7) | 0.2 (0.12-0.38) | 1111.23 (615.4-1968.4) | 0.15 (0.09-0.27) | -0.92 (-1--0.84) |
| Chronic myeloid leukemia | Association of Southeast Asian Nations | 2003.86 (1118.46-2847.2) | 0.67 (0.34-0.96) | 2375.51 (1335.69-3271.38) | 0.38 (0.22-0.52) | -2.03 (-2.15--1.91) |
| Chronic myeloid leukemia | Central Europe, Eastern Europe, and Central Asia | 2959.56 (2527.3-3490.67) | 0.64 (0.55-0.76) | 1745.04 (1564.87-2002.06) | 0.28 (0.25-0.32) | -3.28 (-3.66--2.91) |
| Chronic myeloid leukemia | Commonwealth | 5394.56 (3847.2-7182.7) | 0.58 (0.43-0.75) | 6345.36 (3967.77-8938.72) | 0.31 (0.2-0.44) | -2.14 (-2.3--1.98) |
| Chronic myeloid leukemia | European Union | 6613.3 (6053.17-7432.69) | 1.15 (1.06-1.29) | 3145.39 (2717.14-3554.77) | 0.3 (0.27-0.34) | -5.02 (-5.32--4.73) |
| Chronic myeloid leukemia | Four World Regions | 29144.91 (23453.77-34169.51) | 0.72 (0.59-0.84) | 23099.74 (17455.21-28414.58) | 0.28 (0.21-0.34) | -3.42 (-3.59--3.25) |
| Chronic myeloid leukemia | G20 | 23020.65 (19238.39-26434.5) | 0.76 (0.64-0.86) | 15816.95 (12676.98-19282.11) | 0.26 (0.2-0.31) | -3.96 (-4.18--3.74) |
| Chronic myeloid leukemia | Gulf Cooperation Council | 55.53 (27.79-140.29) | 0.56 (0.28-1.44) | 138.62 (72.61-392.62) | 0.4 (0.22-1.04) | -0.93 (-1.57--0.28) |
| Chronic myeloid leukemia | Health System Grouping Levels | 29180.51 (23490.05-34205.72) | 0.72 (0.59-0.84) | 23128.49 (17479.81-28443.07) | 0.28 (0.21-0.34) | -3.42 (-3.59--3.25) |
| Chronic myeloid leukemia | High SDI | 11180.44 (10407.46-12180.16) | 1.04 (0.97-1.13) | 5730.47 (5049.09-6275.81) | 0.27 (0.24-0.29) | -5 (-5.34--4.67) |
| Chronic myeloid leukemia | High-income | 12586.95 (11808.02-13587.38) | 1.09 (1.02-1.17) | 6302.35 (5534.73-6927.56) | 0.27 (0.24-0.3) | -5.12 (-5.48--4.77) |
| Chronic myeloid leukemia | High-middle SDI | 6691.52 (5384.12-8028.85) | 0.68 (0.55-0.82) | 4100.2 (3295.32-4903.19) | 0.22 (0.18-0.26) | -4.21 (-4.49--3.92) |
| Chronic myeloid leukemia | Latin America and Caribbean | 1950.99 (1828.36-2112.93) | 0.77 (0.72-0.83) | 2269.47 (2025.45-2496.22) | 0.37 (0.33-0.41) | -2.77 (-3--2.53) |
| Chronic myeloid leukemia | Low SDI | 1180.97 (626.34-1837.76) | 0.4 (0.23-0.68) | 1595.51 (909.12-2593.05) | 0.27 (0.16-0.44) | -1.33 (-1.43--1.23) |
| Chronic myeloid leukemia | Low-middle SDI | 4172.27 (2639.32-5816.79) | 0.56 (0.37-0.78) | 5287.36 (3366.17-7635.89) | 0.37 (0.23-0.53) | -1.42 (-1.47--1.37) |
| Chronic myeloid leukemia | Middle SDI | 5955.32 (3914.88-7420.41) | 0.49 (0.33-0.61) | 6414.95 (4227.21-8428.26) | 0.25 (0.17-0.33) | -2.38 (-2.51--2.25) |
| Chronic myeloid leukemia | Nordic Region | 242.71 (223.71-261) | 0.67 (0.62-0.72) | 130.76 (113.36-150.79) | 0.22 (0.19-0.25) | -4.24 (-4.69--3.78) |
| Chronic myeloid leukemia | North Africa and Middle East | 1718.41 (1000.28-2424.86) | 0.86 (0.53-1.29) | 2456.18 (1255.37-3513.73) | 0.54 (0.28-0.78) | -1.32 (-1.4--1.24) |
| Chronic myeloid leukemia | OECD Countries | 13947.61 (13074.36-15078.67) | 1.07 (1-1.15) | 7407.36 (6519-8112.12) | 0.28 (0.26-0.31) | -4.89 (-5.22--4.56) |
| Chronic myeloid leukemia | Organization of Islamic Cooperation | 3658.82 (2227.93-4919.67) | 0.57 (0.36-0.78) | 4777.85 (2675.56-6411.87) | 0.37 (0.21-0.5) | -1.4 (-1.45--1.35) |
| Chronic myeloid leukemia | Sahel Region | 181.03 (67.93-340.97) | 0.17 (0.09-0.36) | 219.67 (106.47-344.92) | 0.11 (0.05-0.19) | -1.69 (-1.8--1.57) |
| Chronic myeloid leukemia | South Asia | 3826.34 (2300.75-5466.91) | 0.56 (0.34-0.81) | 5359.81 (3148.9-7891.74) | 0.37 (0.22-0.55) | -1.36 (-1.45--1.27) |
| Chronic myeloid leukemia | Southeast Asia, East Asia, and Oceania | 5810.61 (3031.27-7885.11) | 0.43 (0.22-0.59) | 4524.44 (2692.68-6391.1) | 0.18 (0.11-0.25) | -3.27 (-3.46--3.08) |
| Chronic myeloid leukemia | Sub-Saharan Africa | 367.56 (181.91-663.34) | 0.12 (0.06-0.23) | 501.84 (232.4-1040.44) | 0.08 (0.04-0.17) | -1.58 (-1.63--1.52) |
| Chronic myeloid leukemia | WHO region | 29083.6 (23389.75-34106.94) | 0.72 (0.59-0.84) | 23017.3 (17372.79-28334.54) | 0.28 (0.21-0.34) | -3.42 (-3.6--3.25) |
| Chronic myeloid leukemia | World Bank Income Levels | 29180.42 (23489.97-34205.6) | 0.72 (0.59-0.84) | 23128.41 (17479.78-28442.98) | 0.28 (0.21-0.34) | -3.42 (-3.59--3.25) |
| Chronic myeloid leukemia | World Bank Regions | 29180.42 (23489.97-34205.6) | 0.72 (0.59-0.84) | 23128.41 (17479.78-28442.98) | 0.28 (0.21-0.34) | -3.42 (-3.59--3.25) |
| Other leukemia | American Samoa | 0.2 (0.2-0.4) | 0.9 (0.6-1.5) | 0.5 (0.3-0.7) | 1.2 (0.7-1.6) | 0.18 (-0.62-1) |
| Other leukemia | Antigua and Barbuda | 0.1 (0.1-0.1) | 0.2 (0.2-0.2) | 0.1 (0.1-0.1) | 0.1 (0.1-0.1) | -0.2 (-0.97-0.58) |
| Other leukemia | Arab Republic of Egypt | 141.9 (56-486.9) | 0.5 (0.2-1.9) | 329.3 (103.5-928.6) | 0.6 (0.2-1.7) | 0.92 (0.11-1.74) |
| Other leukemia | Argentine Republic | 237.2 (200.1-277.1) | 0.8 (0.6-0.9) | 429.2 (348.4-501) | 0.8 (0.6-0.9) | -0.52 (-1.27-0.24) |
| Other leukemia | Australia | 152.6 (109.2-175.1) | 0.8 (0.6-0.9) | 218.6 (176.2-260.5) | 0.4 (0.4-0.5) | -0.68 (-1.46-0.1) |
| Other leukemia | Barbados | 5.1 (4.7-5.5) | 1.7 (1.6-1.9) | 5.3 (4.2-6.5) | 1.1 (0.9-1.3) | -0.03 (-0.83-0.77) |
| Other leukemia | Belize | 0.9 (0.8-1.1) | 0.8 (0.7-1) | 2.7 (2.3-3.1) | 0.8 (0.7-0.9) | -0.03 (-0.62-0.57) |
| Other leukemia | Bermuda | 0.8 (0.7-0.9) | 1.3 (1.2-1.5) | 0.8 (0.7-1) | 0.6 (0.5-0.8) | -0.87 (-1.63--0.11) |
| Other leukemia | Bolivarian Republic of Venezuela | 38.4 (34.8-42.3) | 0.3 (0.3-0.4) | 90.8 (66.6-118.1) | 0.3 (0.2-0.4) | -0.22 (-0.94-0.51) |
| Other leukemia | Bosnia and Herzegovina | 14.5 (4.8-22.3) | 0.4 (0.1-0.6) | 20.1 (6.5-31.9) | 0.3 (0.1-0.5) | -0.03 (-0.73-0.67) |
| Other leukemia | Brunei Darussalam | 1.4 (0.9-2.1) | 1.2 (0.8-1.8) | 3 (1.7-4.1) | 0.9 (0.5-1.2) | -0.39 (-1.21-0.45) |
| Other leukemia | Burkina Faso | 4.7 (1.7-7.3) | 0.1 (0-0.1) | 16.7 (4-30) | 0.1 (0-0.2) | 0.26 (-0.12-0.65) |
| Other leukemia | Canada | 147.2 (133.7-159.6) | 0.4 (0.4-0.5) | 312 (267.7-349.2) | 0.4 (0.3-0.4) | -0.6 (-1.44-0.24) |
| Other leukemia | Central African Republic | 1 (0.4-1.7) | 0.1 (0-0.1) | 2.3 (0.6-4) | 0.1 (0-0.2) | -0.06 (-0.65-0.54) |
| Other leukemia | Commonwealth of Dominica | 0.4 (0.2-0.5) | 0.6 (0.3-0.9) | 0.4 (0.2-0.6) | 0.5 (0.3-0.7) | 0.07 (-0.69-0.83) |
| Other leukemia | Commonwealth of the Bahamas | 0.9 (0.8-1) | 0.5 (0.4-0.5) | 1.4 (1.1-1.7) | 0.3 (0.3-0.4) | -0.14 (-0.76-0.5) |
| Other leukemia | Cook Islands | 0 (0-0.1) | 0.3 (0.2-0.5) | 0 (0-0.1) | 0.2 (0.1-0.3) | -0.42 (-0.88-0.03) |
| Other leukemia | Czech Republic | 203.4 (170.5-228.4) | 1.5 (1.2-1.7) | 246.8 (189.9-306.3) | 1.1 (0.8-1.3) | -0.7 (-1.46-0.08) |
| Other leukemia | Democratic People's Republic of Korea | 102 (58.9-161) | 0.6 (0.3-0.9) | 172.7 (99.3-282.3) | 0.5 (0.3-0.9) | -0.24 (-1.03-0.56) |
| Other leukemia | Democratic Republic of Sao Tome and Principe | 0 (0-0) | 0 (0-0.1) | 0.1 (0-0.1) | 0 (0-0.1) | -0.1 (-0.38-0.18) |
| Other leukemia | Democratic Republic of the Congo | 9 (3.3-15.7) | 0.1 (0-0.1) | 28.6 (7.7-56.3) | 0.1 (0-0.1) | 0.02 (-0.51-0.54) |
| Other leukemia | Democratic Republic of Timor-Leste | 2.2 (1.1-5.3) | 0.6 (0.3-1.5) | 5.1 (2.8-12.3) | 0.6 (0.3-1.4) | -0.3 (-1.08-0.49) |
| Other leukemia | Democratic Socialist Republic of Sri Lanka | 158.7 (85.2-208.8) | 1.4 (0.8-1.9) | 168.2 (94.8-288.1) | 0.6 (0.4-1.1) | -0.77 (-1.55-0.01) |
| Other leukemia | Dominican Republic | 34.6 (19.3-46) | 0.8 (0.4-1.1) | 64.3 (40.8-95.8) | 0.6 (0.4-0.9) | -0.06 (-0.7-0.57) |
| Other leukemia | Eastern Republic of Uruguay | 21.5 (18.3-24.9) | 0.6 (0.5-0.7) | 36.5 (30.8-42.5) | 0.7 (0.6-0.8) | -0.36 (-1.13-0.42) |
| Other leukemia | Federal Democratic Republic of Ethiopia | 590.4 (279.9-957.4) | 2.2 (1.1-3.5) | 683 (292.8-1288.6) | 1.3 (0.6-2.4) | -1.11 (-2.01--0.19) |
| Other leukemia | Federal Democratic Republic of Nepal | 110.2 (65.1-166.4) | 1 (0.6-1.5) | 190.6 (117.9-291) | 0.8 (0.5-1.2) | -0.23 (-0.85-0.4) |
| Other leukemia | Federal Republic of Germany | 367.7 (306.2-444.7) | 0.3 (0.2-0.3) | 778.3 (602.5-945.3) | 0.4 (0.3-0.4) | -0.55 (-1.36-0.27) |
| Other leukemia | Federal Republic of Nigeria | 69.6 (26.1-111.1) | 0.1 (0-0.2) | 122.1 (39.3-193.2) | 0.1 (0-0.2) | -0.02 (-0.38-0.34) |
| Other leukemia | Federal Republic of Somalia | 14.4 (6.1-34.3) | 0.4 (0.2-0.9) | 49 (25.6-81.5) | 0.6 (0.3-0.9) | 0.19 (-0.39-0.77) |
| Other leukemia | Federated States of Micronesia | 0.3 (0.1-0.7) | 0.7 (0.3-1.3) | 0.4 (0.2-0.7) | 0.5 (0.3-1) | -0.28 (-1.15-0.59) |
| Other leukemia | Federative Republic of Brazil | 419.1 (385.4-447) | 0.5 (0.4-0.5) | 1004.2 (894.6-1080.9) | 0.4 (0.4-0.4) | -0.3 (-1.02-0.43) |
| Other leukemia | French Republic | 651.7 (544.1-727.6) | 0.8 (0.6-0.8) | 1012.7 (833.3-1206.7) | 0.6 (0.5-0.7) | -0.61 (-1.42-0.2) |
| Other leukemia | Gabonese Republic | 0.3 (0.1-0.5) | 0.1 (0-0.1) | 0.7 (0.2-1.3) | 0.1 (0-0.1) | -0.02 (-0.6-0.57) |
| Other leukemia | Georgia | 79.7 (67.4-91.9) | 1.3 (1.1-1.5) | 68.6 (58.9-78.9) | 1.2 (1.1-1.4) | -0.01 (-0.71-0.7) |
| Other leukemia | Grand Duchy of Luxembourg | 4.1 (3.8-4.5) | 0.8 (0.7-0.8) | 5.4 (4.5-6.2) | 0.5 (0.4-0.5) | -0.95 (-1.9-0) |
| Other leukemia | Greenland | 0.2 (0.1-0.5) | 0.6 (0.4-1.5) | 0.3 (0.2-0.7) | 0.5 (0.3-1.1) | -0.64 (-1.29-0.03) |
| Other leukemia | Grenada | 1.1 (0.9-1.2) | 1.4 (1.3-1.6) | 1.4 (1.2-1.5) | 1.2 (1.1-1.4) | -0.03 (-0.68-0.62) |
| Other leukemia | Guam | 0.1 (0-0.2) | 0.2 (0-0.4) | 0.1 (0-0.3) | 0 (0-0.1) | -0.36 (-1.11-0.38) |
| Other leukemia | Hashemite Kingdom of Jordan | 9.1 (4.6-16.7) | 0.6 (0.3-1.1) | 32 (13.9-53.7) | 0.5 (0.2-0.8) | -0.79 (-1.83-0.26) |
| Other leukemia | Hellenic Republic | 220.3 (202-237.1) | 1.5 (1.4-1.6) | 316.7 (271.1-351.1) | 1.1 (1-1.2) | -0.69 (-1.52-0.14) |
| Other leukemia | Hungary | 86.5 (77.5-96) | 0.6 (0.5-0.7) | 91.8 (75-110.2) | 0.5 (0.4-0.6) | -0.82 (-1.6--0.03) |
| Other leukemia | Independent State of Papua New Guinea | 8.1 (1.9-18.4) | 0.5 (0.1-1.1) | 15.8 (3.7-34.3) | 0.4 (0.1-0.8) | -0.21 (-0.95-0.53) |
| Other leukemia | Independent State of Samoa | 0.7 (0.3-1.6) | 0.9 (0.4-1.9) | 0.8 (0.4-2) | 0.6 (0.3-1.5) | -0.24 (-1.16-0.68) |
| Other leukemia | Ireland | 21.2 (18.5-23.9) | 0.5 (0.5-0.6) | 23.7 (19.7-28) | 0.3 (0.2-0.3) | -1.01 (-1.81--0.19) |
| Other leukemia | Islamic Republic of Afghanistan | 70.1 (17.5-207.7) | 1 (0.3-3.1) | 99.2 (33.5-280.5) | 0.8 (0.3-2.3) | -0.2 (-1.14-0.75) |
| Other leukemia | Islamic Republic of Iran | 226.9 (53-339.8) | 0.8 (0.2-1.2) | 414.7 (111.5-559.7) | 0.6 (0.2-0.8) | -0.61 (-1.39-0.17) |
| Other leukemia | Islamic Republic of Mauritania | 1 (0.5-1.4) | 0.1 (0-0.1) | 2.8 (0.7-5.5) | 0.1 (0-0.2) | 0.12 (-0.24-0.47) |
| Other leukemia | Islamic Republic of Pakistan | 680.5 (409.3-963.4) | 1.1 (0.6-1.5) | 1317.8 (871.9-1929) | 1 (0.7-1.4) | -0.15 (-0.86-0.56) |
| Other leukemia | Jamaica | 29.8 (26.4-33.2) | 1.6 (1.4-1.8) | 44.8 (33.6-57.2) | 1.4 (1.1-1.8) | 0.12 (-0.59-0.83) |
| Other leukemia | Japan | 899.8 (851.9-932.3) | 0.5 (0.5-0.6) | 3097.9 (2673.8-3383.3) | 0.8 (0.7-0.9) | -0.53 (-1.28-0.23) |
| Other leukemia | Kingdom of Bahrain | 5.9 (3.6-8) | 3.5 (2.1-4.6) | 15 (9.4-21) | 2.1 (1.4-2.9) | -1.23 (-2.22--0.23) |
| Other leukemia | Kingdom of Belgium | 178.5 (149.1-204.2) | 1.1 (1-1.3) | 159.3 (128.7-189) | 0.6 (0.5-0.7) | -0.78 (-1.6-0.05) |
| Other leukemia | Kingdom of Bhutan | 3.1 (1.5-4.9) | 1 (0.6-1.6) | 4.9 (3-7.5) | 0.8 (0.5-1.2) | -0.34 (-0.99-0.32) |
| Other leukemia | Kingdom of Cambodia | 50.8 (23.3-119.7) | 1 (0.5-2.3) | 93.6 (50.5-225.6) | 0.7 (0.4-1.8) | -0.48 (-1.35-0.4) |
| Other leukemia | Kingdom of Denmark | 67.1 (57-76.1) | 0.8 (0.7-0.9) | 78.6 (64.7-93.3) | 0.6 (0.5-0.7) | -0.74 (-1.6-0.13) |
| Other leukemia | Kingdom of Eswatini | 0.2 (0.1-0.4) | 0.1 (0-0.2) | 0.4 (0.1-0.8) | 0.1 (0-0.1) | 0.34 (-0.51-1.21) |
| Other leukemia | Kingdom of Lesotho | 0.5 (0.2-1.2) | 0.1 (0-0.1) | 1 (0.4-1.7) | 0.1 (0-0.2) | 0.94 (0.14-1.74) |
| Other leukemia | Kingdom of Morocco | 19.5 (7.9-60.3) | 0.1 (0-0.4) | 35.8 (11.6-100.9) | 0.1 (0-0.3) | -0.08 (-0.46-0.29) |
| Other leukemia | Kingdom of Norway | 20.4 (18.7-21.6) | 0.3 (0.3-0.3) | 41.4 (36.2-45.4) | 0.4 (0.3-0.4) | -0.33 (-1.13-0.48) |
| Other leukemia | Kingdom of Saudi Arabia | 38.2 (13.7-77.1) | 0.6 (0.2-1.2) | 141.4 (52.2-227.2) | 0.6 (0.2-1) | 0.26 (-0.34-0.87) |
| Other leukemia | Kingdom of Spain | 433.2 (381.4-475.6) | 0.8 (0.7-0.9) | 582.2 (465-693.3) | 0.5 (0.4-0.6) | -0.8 (-1.53--0.07) |
| Other leukemia | Kingdom of Sweden | 23.1 (19-26.8) | 0.1 (0.1-0.2) | 73.3 (58-89.5) | 0.3 (0.2-0.3) | -0.55 (-1.35-0.26) |
| Other leukemia | Kingdom of Thailand | 251.2 (132.5-384.7) | 0.7 (0.4-1) | 373.4 (225-754.7) | 0.4 (0.2-0.8) | -0.59 (-1.45-0.27) |
| Other leukemia | Kingdom of the Netherlands | 77.6 (61.1-88.9) | 0.4 (0.3-0.4) | 177.1 (110.5-224.6) | 0.5 (0.3-0.6) | -0.65 (-1.48-0.19) |
| Other leukemia | Kingdom of Tonga | 0.3 (0.1-0.7) | 0.7 (0.3-1.4) | 0.4 (0.2-0.9) | 0.5 (0.2-1.2) | -0.13 (-0.88-0.62) |
| Other leukemia | Kyrgyz Republic | 35.5 (30.1-42) | 1 (0.9-1.2) | 24 (19.5-30.1) | 0.5 (0.4-0.6) | -0.67 (-1.25--0.1) |
| Other leukemia | Lao People's Democratic Republic | 22 (8.8-54.1) | 0.9 (0.4-2.3) | 30.8 (15.8-76.4) | 0.6 (0.3-1.5) | -0.77 (-1.63-0.1) |
| Other leukemia | Lebanese Republic | 12.7 (6.7-29.9) | 0.6 (0.3-1.4) | 27 (14.5-53.1) | 0.4 (0.2-0.8) | -0.36 (-1.17-0.46) |
| Other leukemia | Malaysia | 140.8 (98.8-264.9) | 1.3 (0.9-2.6) | 326.8 (225.6-644.3) | 1.2 (0.8-2.3) | -0.31 (-1.14-0.53) |
| Other leukemia | Mongolia | 3.9 (2.2-6.2) | 0.3 (0.2-0.5) | 5.5 (2.9-8.5) | 0.2 (0.1-0.3) | -0.43 (-1.11-0.26) |
| Other leukemia | Montenegro | 2.4 (0.8-3.9) | 0.4 (0.1-0.6) | 3.3 (1.3-5) | 0.4 (0.1-0.5) | -0.12 (-0.87-0.64) |
| Other leukemia | New Zealand | 10.8 (8.9-12.9) | 0.3 (0.2-0.3) | 64.1 (51.7-77) | 0.7 (0.6-0.8) | -0.58 (-1.44-0.28) |
| Other leukemia | North Macedonia | 0 (0-0) | 0 (0-0) | 0 (0-0) | 0 (0-0) | -0.23 (-1.25-0.79) |
| Other leukemia | Northern Mariana Islands | 0.2 (0.1-0.3) | 1.1 (0.3-1.8) | 0.7 (0.2-1) | 1.5 (0.4-2.1) | -0.52 (-1.28-0.24) |
| Other leukemia | Palestine | 41.1 (28.1-56.2) | 4.3 (2.9-5.8) | 71.7 (49.3-97.6) | 2.8 (1.9-3.7) | -0.8 (-1.71-0.11) |
| Other leukemia | People's Democratic Republic of Algeria | 184.6 (131.4-244.1) | 1.4 (1-1.8) | 346.8 (241-487.7) | 1.1 (0.7-1.5) | -0.28 (-0.92-0.36) |
| Other leukemia | People's Republic of Bangladesh | 666 (365.4-981.6) | 1.1 (0.6-1.6) | 968.1 (527.9-1543.2) | 0.7 (0.4-1.1) | -0.59 (-1.24-0.06) |
| Other leukemia | People's Republic of China | 10205.2 (6022.1-13818.9) | 1.1 (0.6-1.5) | 12502.7 (6546.7-17075.4) | 0.6 (0.3-0.9) | -1.08 (-1.87--0.29) |
| Other leukemia | Plurinational State of Bolivia | 55.5 (32.4-83.2) | 1.6 (1-2.3) | 106.5 (61.6-156) | 1.2 (0.7-1.7) | -0.42 (-1.3-0.48) |
| Other leukemia | Portuguese Republic | 55.8 (47.9-62.8) | 0.4 (0.4-0.5) | 91 (69.5-112) | 0.3 (0.2-0.4) | -0.79 (-1.59-0.01) |
| Other leukemia | Principality of Andorra | 0.3 (0.1-0.6) | 0.5 (0.2-1.1) | 0.5 (0.3-1.1) | 0.3 (0.2-0.7) | -0.9 (-1.78--0.01) |
| Other leukemia | Principality of Monaco | 0.4 (0.2-0.9) | 0.6 (0.3-1.2) | 0.9 (0.4-1.8) | 0.8 (0.4-1.8) | 0.1 (-0.95-1.17) |
| Other leukemia | Puerto Rico | 9.6 (7.8-11.2) | 0.3 (0.2-0.3) | 26.3 (20-33.2) | 0.4 (0.3-0.5) | -0.7 (-1.46-0.06) |
| Other leukemia | Republic of Albania | 8.5 (3.1-14.1) | 0.5 (0.2-0.7) | 13.4 (5-23.7) | 0.3 (0.1-0.6) | -0.39 (-1.07-0.3) |
| Other leukemia | Republic of Angola | 2.5 (1-4.4) | 0.1 (0-0.1) | 8.6 (2.2-16.6) | 0.1 (0-0.1) | -0.09 (-0.64-0.47) |
| Other leukemia | Republic of Armenia | 18.5 (15-22.3) | 0.6 (0.5-0.8) | 27.5 (22.2-33.4) | 0.7 (0.5-0.8) | -0.62 (-1.3-0.06) |
| Other leukemia | Republic of Austria | 54.3 (48.8-61.4) | 0.5 (0.4-0.5) | 83.4 (66-99.7) | 0.4 (0.3-0.5) | -0.49 (-1.3-0.34) |
| Other leukemia | Republic of Azerbaijan | 49.4 (26.6-72.5) | 0.9 (0.5-1.3) | 57.4 (30.3-95) | 0.6 (0.3-0.9) | -0.64 (-1.38-0.1) |
| Other leukemia | Republic of Belarus | 185.1 (148.5-235.3) | 1.5 (1.2-1.8) | 68 (52.7-85.4) | 0.4 (0.3-0.5) | -0.69 (-1.4-0.03) |
| Other leukemia | Republic of Benin | 2.1 (0.9-3.1) | 0.1 (0-0.1) | 9.2 (2.3-15.8) | 0.1 (0-0.2) | 0.2 (-0.18-0.58) |
| Other leukemia | Republic of Botswana | 0.3 (0.1-0.7) | 0.1 (0-0.1) | 0.7 (0.2-1.6) | 0 (0-0.1) | -0.07 (-0.81-0.67) |
| Other leukemia | Republic of Bulgaria | 105.7 (85.2-126.3) | 1 (0.8-1.2) | 117 (87.5-150) | 0.8 (0.6-1.1) | 0.05 (-0.67-0.78) |
| Other leukemia | Republic of Burundi | 11 (5.4-24.5) | 0.3 (0.2-0.7) | 25.5 (11.6-45.1) | 0.4 (0.2-0.7) | -0.13 (-0.68-0.42) |
| Other leukemia | Republic of Cabo Verde | 0.5 (0.2-0.8) | 0.2 (0.1-0.4) | 2 (0.8-3) | 0.3 (0.1-0.5) | 0.14 (-0.46-0.75) |
| Other leukemia | Republic of Cameroon | 5.6 (2.8-7.6) | 0.1 (0-0.1) | 28.1 (7.5-49.4) | 0.1 (0-0.2) | 0.23 (-0.18-0.64) |
| Other leukemia | Republic of Chad | 2.4 (0.9-4.1) | 0.1 (0-0.1) | 11.8 (2.7-22.1) | 0.1 (0-0.3) | 0.39 (0.02-0.77) |
| Other leukemia | Republic of Chile | 54.2 (47.3-60.7) | 0.5 (0.5-0.6) | 104.5 (85.1-123.4) | 0.4 (0.3-0.5) | -0.41 (-1.09-0.27) |
| Other leukemia | Republic of Colombia | 159 (141.6-174.7) | 0.8 (0.7-0.9) | 258.3 (211.4-311.8) | 0.5 (0.4-0.6) | -0.44 (-1.19-0.31) |
| Other leukemia | Republic of Costa Rica | 7.8 (6.5-9.3) | 0.4 (0.4-0.5) | 20.3 (16.2-24.6) | 0.4 (0.3-0.5) | -0.11 (-0.88-0.68) |
| Other leukemia | République de Côte d’Ivoire | 5.9 (2.2-8.5) | 0.1 (0-0.1) | 15.2 (5.1-25.3) | 0.1 (0-0.2) | -0.02 (-0.32-0.28) |
| Other leukemia | Republic of Croatia | 45.5 (34.4-59.2) | 0.8 (0.6-1.1) | 41.3 (28-57.1) | 0.4 (0.3-0.6) | -0.54 (-1.3-0.23) |
| Other leukemia | Republic of Cuba | 28.1 (25.1-31) | 0.3 (0.2-0.3) | 36.8 (31.5-42.7) | 0.2 (0.2-0.2) | -0.44 (-1.14-0.26) |
| Other leukemia | Republic of Cyprus | 1.7 (1-3.4) | 0.3 (0.1-0.6) | 3.1 (1.6-4.9) | 0.2 (0.1-0.2) | -0.67 (-1.65-0.33) |
| Other leukemia | Republic of Djibouti | 0.5 (0.3-1.3) | 0.3 (0.1-0.6) | 2.7 (1.3-4.6) | 0.4 (0.2-0.6) | 0.21 (-0.35-0.77) |
| Other leukemia | Republic of Ecuador | 63.4 (54.1-73.1) | 1.1 (0.9-1.2) | 98.8 (76.9-126.8) | 0.6 (0.5-0.8) | 0.33 (-0.52-1.18) |
| Other leukemia | Republic of El Salvador | 40.5 (26.6-57.4) | 1.2 (0.8-1.8) | 77.7 (51-104.1) | 1.2 (0.8-1.6) | -0.02 (-0.83-0.79) |
| Other leukemia | Republic of Equatorial Guinea | 0.1 (0.1-0.2) | 0.1 (0-0.1) | 0.4 (0.1-0.8) | 0.1 (0-0.1) | -0.19 (-0.72-0.35) |
| Other leukemia | Republic of Estonia | 16.7 (13.5-21.4) | 0.9 (0.7-1.1) | 11.4 (8.9-14.1) | 0.4 (0.3-0.5) | -1 (-1.8--0.2) |
| Other leukemia | Republic of Fiji | 0.3 (0.1-0.5) | 0.1 (0-0.2) | 0.3 (0.1-0.5) | 0 (0-0.1) | -0.32 (-1.45-0.82) |
| Other leukemia | Republic of Finland | 34 (29.1-38.8) | 0.5 (0.4-0.5) | 56.4 (45.6-68.3) | 0.4 (0.3-0.5) | -0.61 (-1.32-0.11) |
| Other leukemia | Republic of Ghana | 21.3 (6.5-33.6) | 0.2 (0.1-0.4) | 38.1 (19.5-58.1) | 0.1 (0.1-0.2) | -0.75 (-1.24--0.27) |
| Other leukemia | Republic of Guatemala | 35.8 (32.5-39.1) | 0.9 (0.8-1) | 93.2 (79.7-108.7) | 0.8 (0.7-0.9) | -0.01 (-0.78-0.77) |
| Other leukemia | Republic of Guinea | 1.1 (0.4-1.7) | 0 (0-0) | 2.6 (1-3.9) | 0 (0-0.1) | -0.02 (-0.21-0.16) |
| Other leukemia | Republic of Guinea-Bissau | 0.7 (0.3-1.1) | 0.1 (0.1-0.2) | 2.1 (0.6-3.6) | 0.2 (0.1-0.3) | 0.24 (-0.2-0.68) |
| Other leukemia | Republic of Guyana | 1.9 (1.6-2.2) | 0.4 (0.4-0.5) | 2.3 (1.7-3) | 0.3 (0.3-0.4) | 0.13 (-0.52-0.78) |
| Other leukemia | Republic of Haiti | 51.3 (25-85) | 1.4 (0.7-2.1) | 90 (45.7-140.1) | 1.1 (0.6-1.7) | -0.34 (-1.08-0.41) |
| Other leukemia | Republic of Honduras | 14 (8.1-21.4) | 0.6 (0.3-0.9) | 36.2 (22.2-61.2) | 0.6 (0.3-0.9) | -0.13 (-0.92-0.67) |
| Other leukemia | Republic of Iceland | 1.6 (1.3-1.8) | 0.5 (0.4-0.6) | 2.5 (2-3) | 0.4 (0.3-0.5) | -0.35 (-1.12-0.43) |
| Other leukemia | Republic of India | 4474.9 (2873.5-6216.5) | 0.9 (0.6-1.2) | 8678.6 (5840.1-12094.1) | 0.7 (0.5-1) | -0.31 (-0.92-0.29) |
| Other leukemia | Republic of Indonesia | 699.2 (400.1-1664) | 0.6 (0.4-1.5) | 1393 (842.2-3531.8) | 0.6 (0.3-1.5) | -0.16 (-0.99-0.69) |
| Other leukemia | Republic of Iraq | 42.9 (13.1-158.1) | 0.4 (0.1-1.6) | 86.3 (28-296.1) | 0.4 (0.1-1.2) | -0.36 (-1.22-0.5) |
| Other leukemia | Republic of Italy | 779.7 (703.1-834) | 0.9 (0.8-0.9) | 713.4 (582.9-802.8) | 0.4 (0.3-0.5) | -0.84 (-1.62--0.04) |
| Other leukemia | Republic of Kazakhstan | 127.7 (101.7-152.5) | 0.9 (0.7-1.1) | 108.2 (85.3-134.7) | 0.6 (0.5-0.8) | -0.54 (-1.21-0.12) |
| Other leukemia | Republic of Kenya | 38.9 (18.5-69.8) | 0.4 (0.2-0.8) | 111 (49.5-177) | 0.4 (0.2-0.7) | 0.29 (-0.18-0.76) |
| Other leukemia | Republic of Kiribati | 0.1 (0-0.2) | 0.3 (0.1-0.6) | 0.2 (0.1-0.3) | 0.3 (0.1-0.5) | -0.04 (-0.76-0.68) |
| Other leukemia | Republic of Korea | 102.3 (66.1-204.1) | 0.3 (0.2-0.7) | 155.1 (81.6-272.4) | 0.2 (0.1-0.3) | -1 (-1.72--0.27) |
| Other leukemia | Republic of Latvia | 27.4 (22.7-33.2) | 0.8 (0.7-0.9) | 15.6 (11.9-19.2) | 0.4 (0.3-0.5) | -0.81 (-1.58--0.03) |
| Other leukemia | Republic of Liberia | 1.2 (0.5-1.8) | 0.1 (0-0.1) | 4.6 (1.1-8.3) | 0.1 (0-0.2) | 0.23 (-0.14-0.6) |
| Other leukemia | Republic of Lithuania | 33.9 (27.2-43.1) | 0.8 (0.6-1) | 30.9 (24-38.7) | 0.5 (0.4-0.7) | -0.54 (-1.31-0.25) |
| Other leukemia | Republic of Madagascar | 18.3 (11-41) | 0.3 (0.2-0.6) | 46.2 (24.1-75) | 0.3 (0.2-0.5) | 0.01 (-0.48-0.5) |
| Other leukemia | Republic of Malawi | 6.4 (4.1-12.1) | 0.1 (0.1-0.2) | 11.8 (6.1-18.5) | 0.1 (0.1-0.2) | -0.09 (-0.38-0.2) |
| Other leukemia | Republic of Maldives | 0.9 (0.4-1.6) | 0.9 (0.5-1.5) | 1.2 (0.7-1.8) | 0.3 (0.2-0.5) | -1.16 (-1.89--0.42) |
| Other leukemia | Republic of Mali | 2.5 (1-3.7) | 0.1 (0-0.1) | 5.7 (2-8.7) | 0 (0-0.1) | -0.11 (-0.42-0.2) |
| Other leukemia | Republic of Malta | 1.7 (1.4-1.9) | 0.4 (0.3-0.5) | 2.7 (2.1-3.4) | 0.3 (0.2-0.3) | -0.66 (-1.46-0.15) |
| Other leukemia | Republic of Mauritius | 0 (0-0) | 0 (0-0) | 23.9 (22-25.4) | 1.4 (1.3-1.5) | -0.51 (-1.24-0.22) |
| Other leukemia | Republic of Moldova | 28.3 (25.1-31.6) | 0.6 (0.6-0.7) | 19 (16.6-21.9) | 0.3 (0.3-0.4) | -0.74 (-1.42--0.06) |
| Other leukemia | Republic of Mozambique | 42.6 (24.5-86.6) | 0.5 (0.3-1) | 96.6 (54.3-166.1) | 0.6 (0.4-1.1) | 0.14 (-0.52-0.8) |
| Other leukemia | Republic of Namibia | 0.3 (0.1-0.7) | 0 (0-0.1) | 0.5 (0.2-1.1) | 0 (0-0.1) | -0.03 (-0.65-0.6) |
| Other leukemia | Republic of Nauru | 0 (0-0.1) | 0.9 (0.5-1.6) | 0 (0-0.1) | 0.6 (0.3-1.1) | -0.22 (-1.15-0.73) |
| Other leukemia | Republic of Nicaragua | 5.8 (3.3-9.3) | 0.3 (0.2-0.5) | 14.9 (8.7-21.9) | 0.3 (0.2-0.4) | -0.21 (-0.94-0.53) |
| Other leukemia | Republic of Niue | 0 (0-0) | 0.5 (0.3-0.9) | 0 (0-0) | 0.4 (0.2-0.8) | -0.06 (-0.86-0.74) |
| Other leukemia | Republic of Palau | 0.1 (0-0.1) | 0.6 (0.4-1) | 0.1 (0.1-0.2) | 0.5 (0.4-0.9) | -0.16 (-0.66-0.36) |
| Other leukemia | Republic of Panama | 7.2 (6.4-8) | 0.5 (0.4-0.5) | 20.3 (15.9-24.8) | 0.5 (0.4-0.6) | 0.06 (-0.7-0.83) |
| Other leukemia | Republic of Paraguay | 7.6 (3.9-11.8) | 0.3 (0.2-0.5) | 23.3 (12.9-33) | 0.4 (0.2-0.6) | 0.24 (-0.47-0.96) |
| Other leukemia | Republic of Peru | 154.8 (101.4-208.7) | 1.1 (0.7-1.5) | 291.4 (180.6-417.1) | 0.8 (0.5-1.2) | -0.18 (-0.97-0.62) |
| Other leukemia | Republic of Poland | 486.7 (432.2-539.9) | 1.1 (1-1.3) | 498.9 (439.1-555.2) | 0.7 (0.6-0.7) | -0.79 (-1.55--0.02) |
| Other leukemia | Republic of Rwanda | 15.6 (7.9-34.3) | 0.4 (0.2-0.8) | 26.5 (13.2-46.7) | 0.3 (0.2-0.6) | -0.5 (-1.08-0.08) |
| Other leukemia | Republic of San Marino | 1 (0.6-1.5) | 2.6 (1.7-4.1) | 1.2 (0.7-1.7) | 1.3 (0.8-2) | -0.78 (-1.6-0.04) |
| Other leukemia | Republic of Senegal | 3.8 (1.4-5.5) | 0.1 (0-0.1) | 13.7 (3.4-25.1) | 0.1 (0-0.2) | 0.23 (-0.16-0.61) |
| Other leukemia | Republic of Serbia | 82.7 (45.3-108.1) | 0.9 (0.5-1.1) | 94.2 (54.1-132.5) | 0.6 (0.3-0.8) | -0.51 (-1.26-0.25) |
| Other leukemia | Republic of Seychelles | 1.1 (0.8-1.9) | 1.9 (1.3-3.2) | 1.6 (1.1-2.5) | 1.4 (1-2.3) | -0.43 (-1.15-0.29) |
| Other leukemia | Republic of Sierra Leone | 2 (0.7-3) | 0.1 (0-0.1) | 6.4 (1.4-11.7) | 0.1 (0-0.2) | 0.26 (-0.11-0.64) |
| Other leukemia | Republic of Singapore | 1.9 (1.7-2.1) | 0.1 (0.1-0.1) | 7.1 (6.2-8.1) | 0.1 (0.1-0.1) | -0.85 (-1.66--0.04) |
| Other leukemia | Republic of Slovenia | 8.7 (7.2-10.1) | 0.4 (0.3-0.4) | 17.9 (14-22.4) | 0.4 (0.3-0.5) | -0.5 (-1.23-0.23) |
| Other leukemia | Republic of South Africa | 19.7 (6.7-35.8) | 0.1 (0-0.2) | 36.4 (14.8-63.8) | 0.1 (0-0.1) | 0.09 (-0.68-0.86) |
| Other leukemia | Republic of South Sudan | 11.3 (5.4-27.6) | 0.3 (0.2-0.8) | 25.7 (15.2-40) | 0.5 (0.3-0.8) | 0.3 (-0.25-0.86) |
| Other leukemia | Republic of Sudan | 67.2 (23.4-193.6) | 0.6 (0.2-1.8) | 107.8 (38-283.2) | 0.5 (0.2-1.3) | -0.33 (-1.16-0.51) |
| Other leukemia | Republic of Suriname | 2.4 (1.3-3.2) | 0.9 (0.5-1.2) | 4.5 (2.7-6.5) | 0.7 (0.4-1) | -0.16 (-0.77-0.45) |
| Other leukemia | Republic of Tajikistan | 24.6 (11.3-36.2) | 0.7 (0.3-1.1) | 30.3 (13.3-51.1) | 0.4 (0.2-0.7) | -0.75 (-1.39--0.1) |
| Other leukemia | Republic of the Congo | 0.8 (0.4-1.3) | 0.1 (0-0.1) | 2 (0.6-3.7) | 0.1 (0-0.1) | -0.14 (-0.73-0.45) |
| Other leukemia | Republic of the Gambia | 0.3 (0.1-0.4) | 0.1 (0-0.1) | 0.8 (0.3-1.1) | 0.1 (0-0.1) | -0.02 (-0.24-0.2) |
| Other leukemia | Republic of the Marshall Islands | 0.1 (0-0.2) | 0.6 (0.2-1.2) | 0.2 (0.1-0.3) | 0.5 (0.2-1.1) | -0.05 (-0.88-0.79) |
| Other leukemia | Republic of the Niger | 3.5 (1.3-6.2) | 0.1 (0-0.2) | 13.7 (2.6-33.2) | 0.1 (0-0.3) | 0.12 (-0.25-0.5) |
| Other leukemia | Republic of the Philippines | 93.1 (31.4-123.8) | 0.3 (0.1-0.4) | 179.1 (77.1-242.5) | 0.2 (0.1-0.3) | -0.35 (-1.19-0.49) |
| Other leukemia | Republic of the Union of Myanmar | 248.5 (107.9-581.3) | 0.9 (0.4-2.2) | 289 (161.3-724.8) | 0.6 (0.3-1.5) | -0.95 (-1.84--0.07) |
| Other leukemia | Republic of Trinidad and Tobago | 6.8 (6.2-7.4) | 0.8 (0.7-0.8) | 7.6 (5.8-9.7) | 0.4 (0.3-0.5) | -0.43 (-1.12-0.27) |
| Other leukemia | Republic of Tunisia | 14.3 (5.9-46.5) | 0.3 (0.1-0.9) | 25.6 (8-83.7) | 0.2 (0.1-0.7) | -0.48 (-1.11-0.16) |
| Other leukemia | Republic of Turkey | 228.7 (127.2-363.4) | 0.6 (0.4-1.1) | 409.7 (209.3-603.3) | 0.5 (0.2-0.7) | -1.01 (-1.89--0.12) |
| Other leukemia | Republic of Uganda | 38.2 (20.7-58.3) | 0.4 (0.2-0.6) | 87.9 (36.1-148.4) | 0.4 (0.2-0.6) | -0.04 (-0.5-0.43) |
| Other leukemia | Republic of Uzbekistan | 144.5 (117.1-179.6) | 1.1 (0.9-1.3) | 176.3 (128.1-234.6) | 0.6 (0.4-0.8) | -0.68 (-1.33--0.03) |
| Other leukemia | Republic of Vanuatu | 0.4 (0.1-0.8) | 0.7 (0.2-1.3) | 0.8 (0.3-1.7) | 0.5 (0.2-1) | -0.13 (-0.91-0.65) |
| Other leukemia | Republic of Yemen | 37.6 (13.5-108.1) | 0.6 (0.2-1.8) | 77 (29-203.5) | 0.5 (0.2-1.4) | -0.28 (-1.07-0.51) |
| Other leukemia | Republic of Zambia | 14.4 (8.1-32.4) | 0.3 (0.2-0.7) | 36.7 (16.9-67.4) | 0.4 (0.2-0.7) | -0.05 (-0.63-0.53) |
| Other leukemia | Republic of Zimbabwe | 2.6 (0.9-5.9) | 0.1 (0-0.2) | 7 (2.4-12.6) | 0.1 (0-0.2) | 0.62 (-0.21-1.45) |
| Other leukemia | Romania | 241.7 (219.1-267.5) | 0.9 (0.8-1) | 304.5 (260.5-345.3) | 0.8 (0.7-0.9) | -0.11 (-0.79-0.57) |
| Other leukemia | Russian Federation | 1228.6 (1034.9-1473.4) | 0.7 (0.6-0.8) | 1398.3 (1217-1547.5) | 0.6 (0.5-0.7) | -0.44 (-1.09-0.21) |
| Other leukemia | Saint Kitts and Nevis | 0.8 (0.7-0.9) | 2.2 (1.9-2.5) | 0.9 (0.7-1) | 1.4 (1.2-1.7) | -0.42 (-1.11-0.28) |
| Other leukemia | Saint Lucia | 0.8 (0.8-0.9) | 0.9 (0.8-1) | 1.2 (0.9-1.4) | 0.5 (0.4-0.6) | -0.73 (-1.34--0.11) |
| Other leukemia | Saint Vincent and the Grenadines | 1.3 (1.2-1.4) | 1.6 (1.5-1.8) | 1.7 (1.5-2) | 1.3 (1.1-1.5) | -0.29 (-0.95-0.37) |
| Other leukemia | Slovak Republic | 64.5 (33.7-93.6) | 1.1 (0.6-1.6) | 55.9 (32.3-88.2) | 0.6 (0.3-0.9) | -0.52 (-1.32-0.29) |
| Other leukemia | Socialist Republic of Viet Nam | 281.7 (189.4-495.4) | 0.7 (0.5-1.2) | 450.2 (279.7-951.3) | 0.5 (0.3-1) | -0.39 (-1.08-0.3) |
| Other leukemia | Solomon Islands | 1 (0.2-1.9) | 0.8 (0.2-1.5) | 1.9 (0.7-4.1) | 0.6 (0.2-1.1) | -0.08 (-0.9-0.74) |
| Other leukemia | State of Eritrea | 5.5 (2.8-12.9) | 0.3 (0.2-0.7) | 14.9 (8.8-23.3) | 0.4 (0.3-0.6) | 0.18 (-0.38-0.75) |
| Other leukemia | State of Israel | 12.8 (10.3-15.6) | 0.3 (0.2-0.3) | 40 (31.1-48.2) | 0.3 (0.2-0.4) | -0.73 (-1.7-0.25) |
| Other leukemia | State of Kuwait | 11.8 (10-13.6) | 1.7 (1.5-2) | 16.2 (12.6-20.1) | 0.6 (0.5-0.7) | -1.06 (-1.75--0.36) |
| Other leukemia | State of Libya | 11.2 (4.9-35.6) | 0.5 (0.2-1.7) | 23.7 (8.5-72.7) | 0.5 (0.2-1.4) | 0.11 (-0.77-0.99) |
| Other leukemia | State of Qatar | 3.1 (1.4-4.6) | 2.5 (1.3-3.6) | 12.5 (7-19.5) | 1.3 (0.8-2) | -1.42 (-2.21--0.63) |
| Other leukemia | Sultanate of Oman | 9.2 (4.4-14.6) | 1.2 (0.6-1.8) | 14.2 (8-20.6) | 0.8 (0.4-1.1) | -0.44 (-1.2-0.33) |
| Other leukemia | Swiss Confederation | 42.8 (35.7-51.1) | 0.4 (0.3-0.5) | 91.7 (71.3-114.7) | 0.4 (0.3-0.5) | -0.68 (-1.44-0.08) |
| Other leukemia | Syrian Arab Republic | 40.3 (15.3-124.6) | 0.7 (0.2-2.1) | 63.7 (16.6-201.6) | 0.6 (0.2-1.7) | -0.8 (-1.65-0.06) |
| Other leukemia | Taiwan (Province of China) | 33.9 (28-41) | 0.2 (0.2-0.3) | 102.6 (79-122.8) | 0.3 (0.2-0.3) | 0.19 (-0.59-0.99) |
| Other leukemia | Togolese Republic | 1.4 (0.5-2.1) | 0.1 (0-0.1) | 7.5 (1.8-12.9) | 0.1 (0-0.2) | 0.28 (-0.09-0.66) |
| Other leukemia | Tokelau | 0 (0-0) | 0.5 (0.2-1) | 0 (0-0) | 0.4 (0.2-0.7) | -0.17 (-0.97-0.64) |
| Other leukemia | Turkmenistan | 17.4 (14.1-20.9) | 0.7 (0.6-0.9) | 23.6 (16.7-32.3) | 0.5 (0.4-0.7) | -0.41 (-1.04-0.21) |
| Other leukemia | Tuvalu | 0 (0-0.1) | 0.6 (0.2-1) | 0 (0-0.1) | 0.5 (0.2-0.9) | -0.34 (-1.16-0.48) |
| Other leukemia | Ukraine | 571.8 (475.4-697.9) | 0.8 (0.7-1) | 373.1 (265.6-489.3) | 0.5 (0.4-0.7) | -1.13 (-1.8--0.46) |
| Other leukemia | Union of the Comoros | 0.8 (0.5-1.8) | 0.3 (0.2-0.7) | 2 (1-3.3) | 0.4 (0.2-0.6) | 0.02 (-0.53-0.57) |
| Other leukemia | United Arab Emirates | 10.5 (5-15.4) | 1.9 (0.9-2.6) | 40.2 (19.4-64) | 1.1 (0.7-1.6) | -0.09 (-0.97-0.79) |
| Other leukemia | United Kingdom of Great Britain and Northern Ireland | 159.8 (148.5-168) | 0.2 (0.2-0.2) | 503.1 (444-541.6) | 0.3 (0.3-0.4) | -0.46 (-1.32-0.41) |
| Other leukemia | United Mexican States | 254.6 (243.2-262.4) | 0.5 (0.5-0.6) | 416.8 (369.9-463.8) | 0.3 (0.3-0.4) | -0.21 (-1.03-0.62) |
| Other leukemia | United Republic of Tanzania | 47.2 (28.4-108.9) | 0.3 (0.2-0.7) | 113.6 (55.1-193) | 0.3 (0.2-0.6) | -0.01 (-0.56-0.55) |
| Other leukemia | United States of America | 1173.1 (1071.9-1236.2) | 0.4 (0.3-0.4) | 2630.4 (2341.9-2806.1) | 0.4 (0.4-0.5) | -0.7 (-1.61-0.21) |
| Other leukemia | United States Virgin Islands | 0.1 (0.1-0.3) | 0.2 (0.1-0.3) | 0.2 (0.1-0.3) | 0.1 (0.1-0.2) | -0.54 (-1.26-0.18) |
| Other leukemia | African Union | 1462.55 (924.85-2582.65) | 0.4 (0.27-0.74) | 2619.27 (1462.43-4171.94) | 0.36 (0.22-0.59) | -0.31 (-0.35--0.27) |
| Other leukemia | Association of Southeast Asian Nations | 1790.45 (1221.45-3607.36) | 0.67 (0.46-1.37) | 3145.96 (2237.69-6887.25) | 0.5 (0.36-1.11) | -1.05 (-1.21--0.89) |
| Other leukemia | Central Europe, Eastern Europe, and Central Asia | 3965.67 (3534.05-4443.24) | 0.85 (0.76-0.96) | 3965 (3512.29-4295.38) | 0.63 (0.55-0.68) | -0.85 (-1.02--0.69) |
| Other leukemia | Commonwealth | 6983.65 (4679.43-9554.35) | 0.73 (0.5-0.99) | 13407.87 (9422.11-18236.11) | 0.66 (0.47-0.88) | -0.39 (-0.46--0.33) |
| Other leukemia | European Union | 4292.29 (3932.25-4535.98) | 0.72 (0.66-0.76) | 5589.36 (4774.58-6162.22) | 0.52 (0.45-0.57) | -0.76 (-0.87--0.65) |
| Other leukemia | Four World Regions | 31873.43 (24115.41-41142.23) | 0.77 (0.6-0.98) | 50066.97 (36853.38-62756.17) | 0.6 (0.44-0.75) | -0.73 (-0.77--0.69) |
| Other leukemia | G20 | 24732.55 (18593.46-30868.02) | 0.79 (0.61-0.98) | 38916.69 (28972.03-46811.71) | 0.62 (0.46-0.75) | -0.68 (-0.72--0.64) |
| Other leukemia | Gulf Cooperation Council | 78.79 (39.67-125.41) | 0.84 (0.44-1.31) | 239.59 (119.39-340.65) | 0.74 (0.4-1.03) | -0.33 (-0.52--0.13) |
| Other leukemia | Health System Grouping Levels | 31884.51 (24125.94-41154.6) | 0.77 (0.6-0.98) | 50095.6 (36879.65-62781.22) | 0.6 (0.44-0.75) | -0.73 (-0.77--0.69) |
| Other leukemia | High SDI | 5336.54 (4913.61-5585.74) | 0.48 (0.44-0.51) | 11241.82 (9699.6-12212.81) | 0.5 (0.44-0.54) | 0.29 (0.19-0.38) |
| Other leukemia | High-income | 6015.64 (5528.06-6313.23) | 0.5 (0.46-0.53) | 11901.26 (10387.2-12934.18) | 0.49 (0.43-0.53) | 0.26 (0.15-0.37) |
| Other leukemia | High-middle SDI | 9118.22 (7011.08-10963.91) | 0.92 (0.72-1.1) | 11542.72 (8324.53-13802.05) | 0.62 (0.44-0.75) | -1.03 (-1.15--0.91) |
| Other leukemia | Latin America and Caribbean | 1446.4 (1264.59-1570.04) | 0.61 (0.53-0.66) | 2855.49 (2491.68-3184) | 0.47 (0.41-0.52) | -0.85 (-0.94--0.75) |
| Other leukemia | Low SDI | 1962.93 (1202.81-2979.79) | 0.71 (0.45-1.03) | 3263.62 (1904.85-4849.31) | 0.58 (0.34-0.84) | -0.78 (-0.85--0.72) |
| Other leukemia | Low-middle SDI | 5280.94 (3829.93-8095.34) | 0.76 (0.55-1.17) | 9450.5 (6959.69-14576.98) | 0.65 (0.48-1) | -0.56 (-0.61--0.5) |
| Other leukemia | Middle SDI | 10185.87 (6859.35-13791.01) | 0.85 (0.6-1.17) | 14596.93 (9811.93-19572.21) | 0.56 (0.38-0.75) | -1.36 (-1.4--1.33) |
| Other leukemia | Nordic Region | 146.32 (129.22-158.75) | 0.39 (0.34-0.42) | 252.49 (214.95-282.76) | 0.39 (0.34-0.43) | 0.85 (0.65-1.06) |
| Other leukemia | North Africa and Middle East | 1227.55 (809.63-2395.5) | 0.66 (0.43-1.35) | 2391.97 (1423.37-4182.27) | 0.55 (0.34-0.95) | -0.39 (-0.49--0.28) |
| Other leukemia | OECD Countries | 7323.76 (6730-7653.84) | 0.55 (0.5-0.57) | 13486.88 (11740.25-14633.87) | 0.49 (0.43-0.53) | -0.15 (-0.25--0.04) |
| Other leukemia | Organization of Islamic Cooperation | 4030.98 (2920.87-6783.64) | 0.68 (0.5-1.18) | 7336.7 (5331.67-12345.33) | 0.57 (0.42-0.95) | -0.53 (-0.57--0.49) |
| Other leukemia | Sahel Region | 184.72 (101.12-373.71) | 0.22 (0.13-0.44) | 413.31 (214.11-609.05) | 0.22 (0.12-0.35) | 0.02 (-0.06-0.1) |
| Other leukemia | South Asia | 5934.71 (3767.39-8227.66) | 0.92 (0.58-1.27) | 11159.99 (7596.87-15543.78) | 0.76 (0.52-1.06) | -0.74 (-0.82--0.67) |
| Other leukemia | Southeast Asia, East Asia, and Oceania | 12307.01 (8159.51-17761.55) | 0.94 (0.64-1.37) | 16141.85 (10301.19-23281.85) | 0.6 (0.38-0.86) | -1.44 (-1.51--1.38) |
| Other leukemia | Sub-Saharan Africa | 1023.31 (617.54-1606.23) | 0.35 (0.22-0.53) | 1725.97 (783.11-2733.29) | 0.28 (0.13-0.45) | -0.77 (-0.8--0.73) |
| Other leukemia | WHO region | 31839.3 (24080.83-41107.36) | 0.77 (0.6-0.99) | 49964.1 (36756.04-62678.05) | 0.6 (0.44-0.75) | -0.73 (-0.77--0.69) |
| Other leukemia | World Bank Income Levels | 31884.46 (24125.91-41154.5) | 0.77 (0.6-0.98) | 50095.55 (36879.62-62781.13) | 0.6 (0.44-0.75) | -0.73 (-0.77--0.69) |
| Other leukemia | World Bank Regions | 31884.45 (24125.91-41154.5) | 0.77 (0.6-0.98) | 50095.55 (36879.62-62781.13) | 0.6 (0.44-0.75) | -0.73 (-0.77--0.69) |

Table S3. The number of leukemia DALYs and age standardized DALYs rate of leukemia caused by different reasons from 1990 to 2021.

| Cause | Location | 1990 | | 2021 | | 1990-2021 |
| --- | --- | --- | --- | --- | --- | --- |
|  |  | Number of DALYs  No. ×10^3^ (95% UI) | DALYs rate | Number of DALYs  No. ×10^3^ (95% UI) | DALYs rate | EAPC  No. (95% CI) |
| Leukemia | American Samoa | 57.2 (44.9-71.5) | 135.1 (106.9-165.9) | 65.5 (49.3-82.8) | 133.9 (100.9-169.2) | 0.9 (-1.47-3.32) |
| Leukemia | Antigua and Barbuda | 91.1 (84.1-99.1) | 156.6 (144.6-169.7) | 119.1 (111.9-126.4) | 127.7 (120.4-135.8) | -0.85 (-2.55-0.89) |
| Leukemia | Arab Republic of Egypt | 104752 (85848.6-154309) | 196.9 (163.6-287.3) | 194795 (130220.8-249153.1) | 214.9 (148.6-270.8) | 1.06 (-0.43-2.57) |
| Leukemia | Argentine Republic | 75259 (72548.1-77864.3) | 227 (218.8-234.7) | 74069.9 (69500.9-79817.2) | 153.1 (143.2-165.6) | -1.44 (-2.86-0) |
| Leukemia | Australia | 33479.3 (32270.9-34603.2) | 185.8 (179.3-192.1) | 46551.5 (42716.1-50146.4) | 121.7 (113.3-130.5) | -2.02 (-3.27--0.74) |
| Leukemia | Barbados | 517.8 (486.2-548.4) | 199 (185.9-211.2) | 600.6 (467-746.3) | 163.6 (127.3-205.7) | -0.51 (-1.94-0.94) |
| Leukemia | Belize | 278.9 (256.8-304.6) | 135.4 (126.9-146.3) | 428.9 (384.8-479.9) | 107.6 (96.5-120.2) | -0.27 (-1.54-1) |
| Leukemia | Bermuda | 118.1 (109.5-127.8) | 195.8 (180.9-212.5) | 95.8 (80-115) | 109.7 (91.5-132.3) | -1.97 (-3.42--0.5) |
| Leukemia | Bolivarian Republic of Venezuela | 40109.4 (38461.2-41760) | 209.2 (201.4-217) | 48325.2 (35743.8-63674.3) | 182.2 (134.3-240.1) | -0.85 (-2.52-0.86) |
| Leukemia | Bosnia and Herzegovina | 5560.8 (4599.4-6658.5) | 127.7 (106.5-154) | 5081.5 (3739.5-6428.3) | 103.7 (76.8-130) | -0.58 (-2.07-0.92) |
| Leukemia | Brunei Darussalam | 524.8 (422.5-682.8) | 234.1 (190.9-296.1) | 647.7 (494.7-774.6) | 148.6 (112.7-177.3) | -1.16 (-2.76-0.47) |
| Leukemia | Burkina Faso | 6470.3 (4633.9-9090.9) | 53.3 (39.7-68.2) | 15845.4 (7545.3-22661.1) | 61.2 (29.4-85.1) | 0.82 (-0.76-2.43) |
| Leukemia | Canada | 54801.2 (52511.8-56840.2) | 183.7 (176.4-190.5) | 66568.7 (61108.7-71512.4) | 113.1 (104.9-121.6) | -1.73 (-3.16--0.28) |
| Leukemia | Central African Republic | 2782.4 (1722.5-4254) | 103 (73.1-141) | 4638.3 (2913.1-6948.7) | 93.2 (58.7-131.7) | -0.18 (-1.96-1.63) |
| Leukemia | Commonwealth of Dominica | 136.8 (114.8-167.6) | 195.4 (164.8-237.5) | 151.2 (111.9-191) | 225.7 (166-285.3) | 0.33 (-1.16-1.84) |
| Leukemia | Commonwealth of the Bahamas | 352 (323.1-380.4) | 149.6 (138-161.4) | 516.2 (411.2-643.9) | 128.4 (102.6-160.5) | -0.66 (-1.87-0.56) |
| Leukemia | Cook Islands | 10.7 (7.8-14.2) | 62.4 (45.1-81.4) | 7.9 (5.6-10.2) | 40.4 (27.9-52.8) | -1.92 (-3.7--0.11) |
| Leukemia | Czech Republic | 26496.4 (24950.2-28190.3) | 222.6 (209.6-236.6) | 23652.3 (20242.3-27264.4) | 132.9 (113.5-153.1) | -1.87 (-3.1--0.62) |
| Leukemia | Democratic People's Republic of Korea | 50011.6 (34784.7-68858.1) | 239.1 (166.4-328.4) | 50535.6 (36097.7-72235.7) | 187.6 (134.5-265.5) | -0.67 (-2.32-1.01) |
| Leukemia | Democratic Republic of Sao Tome and Principe | 61.8 (42.5-83.8) | 40.4 (28.3-52.3) | 54.6 (30.8-94.1) | 26.9 (16.8-42) | -0.76 (-2.31-0.81) |
| Leukemia | Democratic Republic of the Congo | 31056.2 (21123.1-45911.5) | 80 (58.5-106.3) | 54678.4 (35126.5-76410.5) | 71.3 (44.5-104.3) | -0.08 (-1.89-1.75) |
| Leukemia | Democratic Republic of Timor-Leste | 1936.8 (998.3-2906.4) | 235.8 (144.1-333.2) | 2440.5 (1806.1-3143.3) | 179.2 (133.6-234.6) | -0.82 (-2.66-1.05) |
| Leukemia | Democratic Socialist Republic of Sri Lanka | 34598.3 (27649.6-41381.4) | 212 (172.9-254.6) | 27000 (17979.4-37656.1) | 113.6 (76.6-157.5) | -1.44 (-3.52-0.69) |
| Leukemia | Dominican Republic | 13807.1 (10995.1-17130.1) | 179.2 (147-219) | 14290.7 (10714.7-18514) | 133.3 (99.4-172.7) | -0.39 (-1.99-1.24) |
| Leukemia | Eastern Republic of Uruguay | 7518.8 (7166.9-7917.8) | 224.6 (213.7-237.1) | 7523.6 (6961.9-8139.6) | 180 (166.2-195.1) | -1.09 (-2.34-0.18) |
| Leukemia | Federal Democratic Republic of Ethiopia | 208743.7 (90138.1-341109.3) | 422 (237.4-614.5) | 245373 (171244.8-350377) | 252 (178.5-367.3) | -2.03 (-3.54--0.5) |
| Leukemia | Federal Democratic Republic of Nepal | 31777.9 (18949.1-46037.8) | 154.6 (104.3-207.2) | 31517.8 (22969-43851.2) | 107.7 (79.2-149.8) | -0.85 (-2.73-1.08) |
| Leukemia | Federal Republic of Germany | 186153.6 (178493.5-193125.8) | 188.3 (181.5-194.8) | 184138.2 (168068.5-197790.3) | 121 (113-129) | -1.65 (-3.1--0.18) |
| Leukemia | Federal Republic of Nigeria | 61900.9 (41354.8-89587.6) | 55.5 (39.4-78.3) | 131924.2 (68999.5-183589.9) | 49.2 (27.1-66.5) | -0.26 (-1.92-1.42) |
| Leukemia | Federal Republic of Somalia | 8772.9 (5268.1-13431.3) | 109.4 (72.9-158) | 20489.1 (12884.1-29923.8) | 109.6 (70.9-154.6) | 0.28 (-1.02-1.61) |
| Leukemia | Federated States of Micronesia | 180.6 (128.4-248.9) | 195.9 (140-270.5) | 159.4 (105.3-218.7) | 164.1 (108.2-226.4) | -1.15 (-3.42-1.17) |
| Leukemia | Federative Republic of Brazil | 260529.2 (247796.2-274134.6) | 186 (178.6-194.4) | 310560.2 (297269-322773.4) | 136.6 (129.8-143) | -1.09 (-2.62-0.46) |
| Leukemia | French Republic | 137289.1 (130893.8-143579) | 200.6 (191.9-209.1) | 141056.8 (125999.4-154985.3) | 125.1 (114.4-135.6) | -1.81 (-3.07--0.52) |
| Leukemia | Gabonese Republic | 734.6 (556.2-938.9) | 80.7 (60.9-101.4) | 1206 (699.4-1801.7) | 75.8 (45.3-113.4) | -0.14 (-2-1.75) |
| Leukemia | Georgia | 13061.8 (12153.4-14076.8) | 237.8 (221-256.9) | 7266.2 (6513.4-8071.9) | 165.2 (148.8-182.2) | -1.09 (-2.5-0.35) |
| Leukemia | Grand Duchy of Luxembourg | 1099.2 (1046.6-1153.7) | 248.4 (236.2-261) | 1191 (1070.3-1318.8) | 131.5 (119.2-146.5) | -2.76 (-4.25--1.25) |
| Leukemia | Greenland | 65 (46.3-80.3) | 137.2 (102-166) | 46.2 (35-61.3) | 73.2 (54.9-96.2) | -1.76 (-3.17--0.32) |
| Leukemia | Grenada | 166.2 (148.9-185.1) | 194.6 (175.1-216.9) | 166.3 (145.2-188.6) | 156.7 (137-176.3) | -0.46 (-1.47-0.57) |
| Leukemia | Guam | 172.4 (139.5-210.3) | 141.2 (116.2-173.6) | 185.1 (142.2-222.7) | 108.4 (82.8-131.5) | -1.37 (-3.64-0.96) |
| Leukemia | Hashemite Kingdom of Jordan | 9150 (7398-11497.2) | 289.7 (231.6-357.9) | 18408.2 (13277.4-24193.4) | 173.8 (125.6-228.1) | -1.65 (-3.48-0.21) |
| Leukemia | Hellenic Republic | 26226.2 (25144.3-27157.6) | 207.7 (200.6-214.8) | 29917.3 (27752.6-31884.7) | 166.3 (156.4-175.9) | -1.49 (-2.69--0.26) |
| Leukemia | Hungary | 27647.1 (25785.2-29745.8) | 227.4 (211.6-245.9) | 21392.9 (17896.9-25609.1) | 143.9 (119.2-173.6) | -1.95 (-3.18--0.71) |
| Leukemia | Independent State of Papua New Guinea | 6077.6 (3162.8-8732.1) | 151.6 (76.7-214.2) | 13964.2 (8247.7-19581.8) | 136.4 (78.2-196.4) | -0.84 (-3.11-1.48) |
| Leukemia | Independent State of Samoa | 283 (219.1-383.6) | 196.5 (153.8-263.5) | 323.5 (241.4-449.9) | 171.8 (130.8-236.8) | -1.11 (-3.33-1.17) |
| Leukemia | Ireland | 6847.9 (6515.4-7202.9) | 176.5 (168.2-185.6) | 6104.2 (5578.1-6676.6) | 90.4 (83.3-98.5) | -2.42 (-3.85--0.96) |
| Leukemia | Islamic Republic of Afghanistan | 40757.8 (18952.5-65087.6) | 421 (208.9-661.5) | 95629.4 (55839.8-138477.8) | 373.7 (215.7-553.1) | -0.41 (-1.93-1.13) |
| Leukemia | Islamic Republic of Iran | 176717.3 (110160-227901.3) | 311.6 (193.5-390.2) | 144488.7 (95894.3-168954) | 176.3 (116.4-205.8) | -1.38 (-2.81-0.08) |
| Leukemia | Islamic Republic of Mauritania | 1060.7 (778.6-1424.3) | 46.2 (35.8-58.4) | 2029.4 (1016.3-3161.8) | 46.9 (24.1-70.2) | 0.21 (-1.42-1.87) |
| Leukemia | Islamic Republic of Pakistan | 182249.3 (121414.6-243443.9) | 158.1 (112.5-200) | 339926 (251932.2-467098.5) | 149.8 (112.9-203.5) | -0.36 (-2.31-1.64) |
| Leukemia | Jamaica | 3381.9 (3088.5-3715) | 142.8 (131.6-155.8) | 3737.9 (2863.7-4858.5) | 131.1 (100.7-169.4) | -0.05 (-1.61-1.55) |
| Leukemia | Japan | 224726 (220302.8-228281.9) | 164.9 (162-167.3) | 206496.8 (188223.6-217895.6) | 91.6 (87.1-94.7) | -2.09 (-3.9--0.25) |
| Leukemia | Kingdom of Bahrain | 837.2 (635.7-1007.7) | 247.6 (183.6-296.4) | 1669.8 (1198.9-2260) | 137.5 (100.3-180.8) | -2.38 (-4.47--0.24) |
| Leukemia | Kingdom of Belgium | 24773.4 (23295.9-26213.3) | 202 (191-212.6) | 23702.6 (21232.7-25928.3) | 131 (120.9-142.2) | -2.1 (-3.43--0.76) |
| Leukemia | Kingdom of Bhutan | 923.8 (538.5-1336.2) | 148.6 (93.7-207.5) | 766.5 (487.6-1183.6) | 109.7 (71.2-166.9) | -1.11 (-3.07-0.88) |
| Leukemia | Kingdom of Cambodia | 31724.4 (18305.6-48961.6) | 308.9 (198.3-446.7) | 37060.8 (26250.6-49861.3) | 230.2 (165-305.8) | -1.07 (-2.82-0.71) |
| Leukemia | Kingdom of Denmark | 14674.6 (14077.6-15290.1) | 226.5 (217.2-235.2) | 12837 (11737.7-13894.5) | 130.6 (120.8-141.6) | -2.04 (-3.36--0.7) |
| Leukemia | Kingdom of Eswatini | 737 (537.2-1098.8) | 116 (87.4-164.1) | 1294.4 (817-1938.7) | 136.9 (89.4-204.5) | 0.69 (-1.6-3.03) |
| Leukemia | Kingdom of Lesotho | 1129.1 (805.2-1547.6) | 86.2 (61.8-116.2) | 2321.9 (1646.7-3139) | 147.4 (105.3-199.3) | 2.14 (-0.13-4.46) |
| Leukemia | Kingdom of Morocco | 12744.3 (9291.2-17148.2) | 51.8 (38.9-67.4) | 13623.2 (9478.9-18352.9) | 38 (26.3-51.1) | -0.79 (-2.16-0.61) |
| Leukemia | Kingdom of Norway | 7874.2 (7594.6-8113.5) | 149.7 (145.4-154.1) | 8364.2 (7720.8-8858.1) | 101.6 (95.7-106.8) | -1.34 (-2.89-0.23) |
| Leukemia | Kingdom of Saudi Arabia | 17298.8 (12215.4-26461.2) | 120.6 (82.9-196.9) | 38776.6 (27300.4-60108.6) | 117.1 (86.8-171) | 0.33 (-0.83-1.51) |
| Leukemia | Kingdom of Spain | 86478.3 (83010.3-89562.2) | 198.8 (191.1-206.1) | 78029.6 (70325.4-84542.6) | 108.3 (99.8-116.3) | -2.42 (-3.74--1.09) |
| Leukemia | Kingdom of Sweden | 17537.8 (16655.3-18402.1) | 157.9 (150.6-165.5) | 16239.6 (14382.5-18119.2) | 94.7 (85.4-104.3) | -1.48 (-3.05-0.11) |
| Leukemia | Kingdom of Thailand | 115787.1 (89222.9-138486.2) | 223.9 (170.6-269.1) | 142145.3 (84576.3-187889.3) | 186.3 (115.2-241.9) | -1.39 (-3.05-0.3) |
| Leukemia | Kingdom of the Netherlands | 28570 (27430.7-29734.3) | 167.9 (161.5-174.5) | 28682.3 (26389.5-30880.9) | 101.4 (94.6-109.2) | -1.97 (-3.44--0.48) |
| Leukemia | Kingdom of Tonga | 98.1 (68.7-127.9) | 110.8 (78.6-145.1) | 108.4 (73.9-149.8) | 108.1 (74.5-147.5) | -0.9 (-2.99-1.23) |
| Leukemia | Kyrgyz Republic | 8782.3 (7872.6-9665.3) | 191.6 (172.5-209.8) | 7082.2 (5838.3-8410.7) | 105.9 (87.3-125.6) | -2.2 (-3.56--0.81) |
| Leukemia | Lao People's Democratic Republic | 13256.6 (6519.4-21504.7) | 314.7 (172.7-480.7) | 14564.6 (10265-20192.5) | 209.1 (149.6-283.1) | -1.5 (-3.31-0.33) |
| Leukemia | Lebanese Republic | 5789.4 (3893.2-8777) | 214.1 (145.4-322.3) | 8509.8 (6726.5-10972.6) | 147.7 (116.6-189.8) | -1.09 (-2.51-0.35) |
| Leukemia | Malaysia | 37685.8 (27667.4-45958.2) | 227.2 (176.4-268.3) | 53528.7 (43808.4-64231.7) | 170.5 (139.9-204.9) | -0.88 (-2.74-1.01) |
| Leukemia | Mongolia | 3676.5 (2827.5-4891.9) | 160.5 (125.6-205.7) | 3512.4 (2590.3-4496.3) | 108.1 (80.4-137.4) | -1.31 (-3.11-0.53) |
| Leukemia | Montenegro | 952.9 (763.2-1153.6) | 154 (123.5-186) | 955.7 (766-1210.7) | 116.8 (92.8-145.2) | -0.85 (-2.36-0.68) |
| Leukemia | New Zealand | 6902.8 (6611.4-7172.8) | 189.8 (182.1-197.7) | 8330.8 (7785.5-8808.1) | 119 (112.3-125.1) | -1.41 (-2.94-0.15) |
| Leukemia | North Macedonia | 3444.3 (2878.2-4196.9) | 180.3 (151.4-220.3) | 3405.7 (2407.4-4317.5) | 123.3 (86.4-157.3) | -0.68 (-3.78-2.53) |
| Leukemia | Northern Mariana Islands | 59.8 (38.8-81.2) | 159.8 (106.5-211.6) | 58.6 (44.4-69.8) | 116.5 (87.8-139.2) | -0.81 (-3.09-1.53) |
| Leukemia | Palestine | 5072.3 (3804.5-6822.8) | 286.7 (216.9-377.9) | 7295.9 (5829.3-9665.8) | 178.5 (145-229.9) | -1.48 (-2.88--0.06) |
| Leukemia | People's Democratic Republic of Algeria | 33043.2 (24530.4-41062.3) | 130.6 (98-158.6) | 33885.4 (24898.3-43609.4) | 83.2 (61.4-107.1) | -1.21 (-2.85-0.47) |
| Leukemia | People's Republic of Bangladesh | 218420.3 (125397.8-325533.9) | 184.5 (123.3-254.7) | 174437.6 (123058.1-234851) | 109.8 (77.5-147.6) | -1.53 (-3.42-0.4) |
| Leukemia | People's Republic of China | 3924466.2 (2969934.5-4726962.8) | 343.6 (260.8-414.8) | 2205220.6 (1612838.7-2736625.1) | 151.5 (108.7-185.1) | -2.7 (-4.38--0.99) |
| Leukemia | Plurinational State of Bolivia | 25193.3 (16887-35184.7) | 365.8 (253.2-503.7) | 29471.5 (20510.1-39615.8) | 260.7 (181.1-350) | -1.05 (-2.81-0.75) |
| Leukemia | Portuguese Republic | 22711.9 (21719.5-23613.1) | 208.1 (198.5-216.7) | 20035.7 (17966-21670.6) | 113.8 (104.9-122.9) | -2.28 (-3.79--0.75) |
| Leukemia | Principality of Andorra | 125.2 (84.6-187.7) | 231.2 (156.1-342.7) | 154.3 (100.4-216.7) | 127.2 (83.6-175.4) | -1.82 (-3.32--0.31) |
| Leukemia | Principality of Monaco | 127.3 (97.2-171.3) | 293.9 (228.6-383) | 185.3 (138.2-228.5) | 302.6 (223.3-373.6) | -0.17 (-1.75-1.43) |
| Leukemia | Puerto Rico | 7201.2 (6817.7-7627) | 199.4 (189-211.1) | 6354.5 (5247.1-7456.5) | 135.6 (112.4-158.9) | -1.65 (-3.07--0.22) |
| Leukemia | Republic of Albania | 4925.4 (4003-6146.6) | 157.3 (128.9-200.9) | 3075.4 (2137.9-4473.9) | 97.6 (67.7-137.8) | -1.21 (-2.88-0.49) |
| Leukemia | Republic of Angola | 10306.3 (5690.8-16057.2) | 95.4 (65.2-131.4) | 22494.4 (14220.1-31964.5) | 77.3 (47.7-115.6) | -0.42 (-2.26-1.46) |
| Leukemia | Republic of Armenia | 8501.6 (8031.1-8990.2) | 251.6 (238.5-264.8) | 4547.4 (4034-5137.1) | 132.6 (118.3-148.5) | -1.7 (-3.02--0.37) |
| Leukemia | Republic of Austria | 16544.3 (15816-17324) | 174.8 (167.2-183.1) | 16295 (14802-17616.6) | 110.6 (101.9-119) | -1.66 (-3.05--0.25) |
| Leukemia | Republic of Azerbaijan | 18055.3 (14610.6-21794.8) | 239.7 (195.1-293.2) | 16135.9 (11296.3-22818.6) | 161.6 (113.8-224.7) | -1.65 (-3.34-0.08) |
| Leukemia | Republic of Belarus | 26432.2 (24662.1-27966.4) | 241.3 (224.1-256.2) | 18216.4 (14945.5-22202.3) | 144.2 (119.2-175) | -2.07 (-3.27--0.85) |
| Leukemia | Republic of Benin | 3472.8 (2491.7-4814.6) | 54 (41.5-70.2) | 8930.2 (4161.6-13161.8) | 58.7 (28.3-84.1) | 0.56 (-1.1-2.25) |
| Leukemia | Republic of Botswana | 1092.6 (764.3-1515.8) | 104.4 (72.8-143.9) | 1978.3 (1316.5-2839) | 96.7 (66.5-136) | -0.19 (-2.44-2.12) |
[truncated: 211,836 more chars]
